# Supplementary material for: Chemically Induced Senescence Prompts Functional Changes in Human Microglia-Like Cells
Source: J Immunol Res. 2025 Feb 24;2025:3214633. doi: 10.1155/jimr/3214633 (PMC11876530; doi:10.1155/jimr/3214633)
Supplement: Supporting Information 2 — File 1: RNA-Seq_MultiQC. [file 3214633.f2.pdf]

A modular tool to aggregate results from bioinformatics analyses across many samples into a single report.

General Statistics

Copy table | Configure columns | Scatter plot | Violin plot | Showing 18/18 rows and 6/9 columns. | Export as CSV

Sample Name	% Aligned	M Aligned	% Dropped	% Dups	% GC	M Seqs
SLO_1-2_1	91.1 %	19.8 M				
SLO_1-2_1_S35_R1_001			0.0 %	52.1 %	46 %	21.8 M
SLO_1-2_2	91.1 %	19.8 M				
SLO_1-2_2_S36_R1_001			0.0 %	51.7 %	46 %	21.8 M
SLO_1-2_3	91.2 %	18.6 M				
SLO_1-2_3_S37_R1_001			0.0 %	51.9 %	46 %	20.4 M
SLO_1_1	91.7 %	18.2 M				
SLO_1_1_S38_R1_001			0.0 %	50.3 %	46 %	19.8 M
SLO_1_2	91.9 %	18.8 M				
SLO_1_2_S39_R1_001			0.0 %	49.3 %	46 %	20.4 M
SLO_1_3	91.9 %	16.7 M				
SLO_1_3_S40_R1_001			0.0 %	48.9 %	46 %	18.2 M
VEH_1	90.7 %	18.0 M				
VEH_1_S32_R1_001			0.0 %	53.0 %	46 %	19.8 M
VEH_2	90.7 %	17.7 M				
VEH_2_S33_R1_001			0.0 %	51.9 %	46 %	19.5 M
VEH_3	90.5 %	22.0 M				
VEH_3_S34_R1_001			0.0 %	55.1 %	46 %	24.3 M

RSeQC

RSeQC package provides a number of useful modules that can comprehensively evaluate high throughput RNA-seq data. *DOI: 10.1093/bioinformatics/bts356.*

Read Distribution

Read Distribution calculates how mapped reads are distributed over genome features.

Percentages | Export Plot

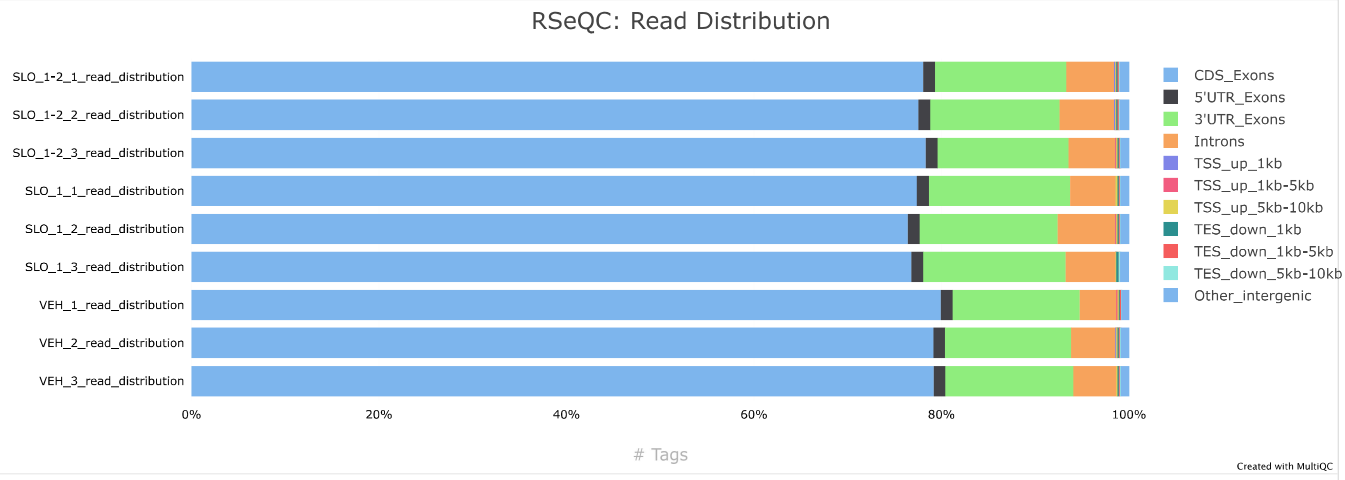

STAR

STAR is an ultrafast universal RNA-seq aligner. *DOI: 10.1093/bioinformatics/bts635.*

Alignment Scores

Percentages | Export Plot

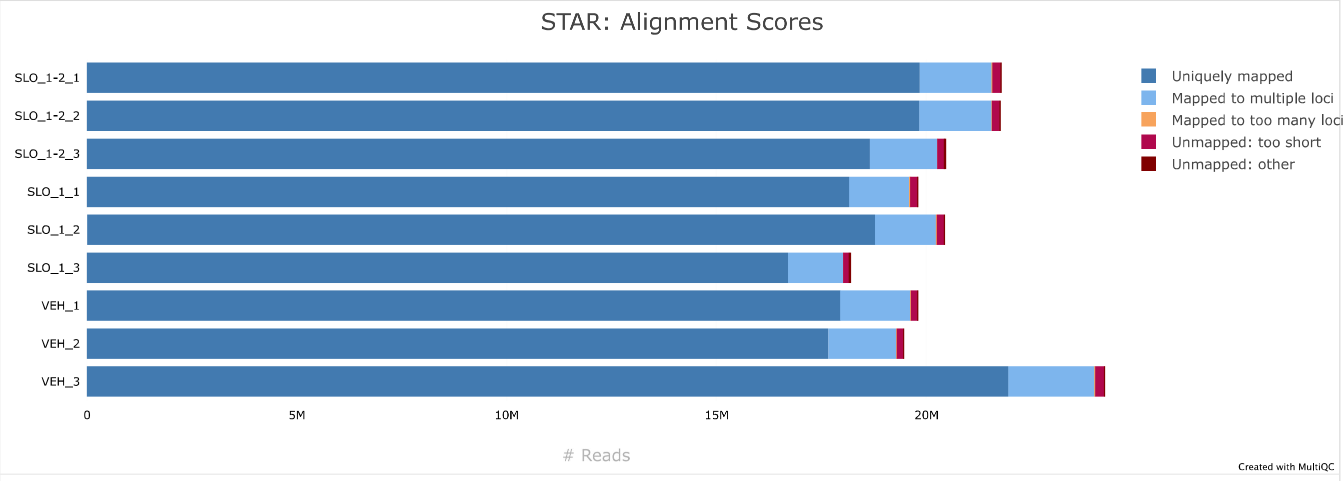

Gene Counts

Statistics from results generated using `--quantMode GeneCounts`. The three tabs show counts for unstranded RNA-seq, counts for the 1st read strand aligned with RNA and counts for the 2nd read strand aligned with RNA.

Percentages | Unstranded | Same Stranded | Reverse Stranded | Export Plot

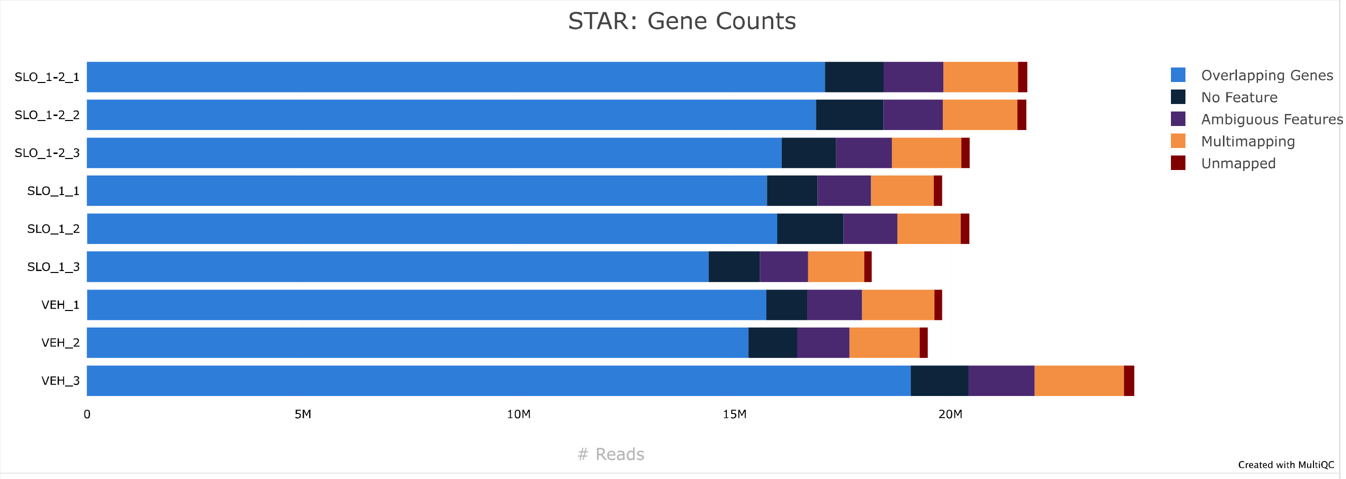

Trimmomatic

Trimmomatic is a flexible read trimming tool for Illumina NGS data. *DOI: 10.1093/bioinformatics/btu170.*

Percentages | Export Plot

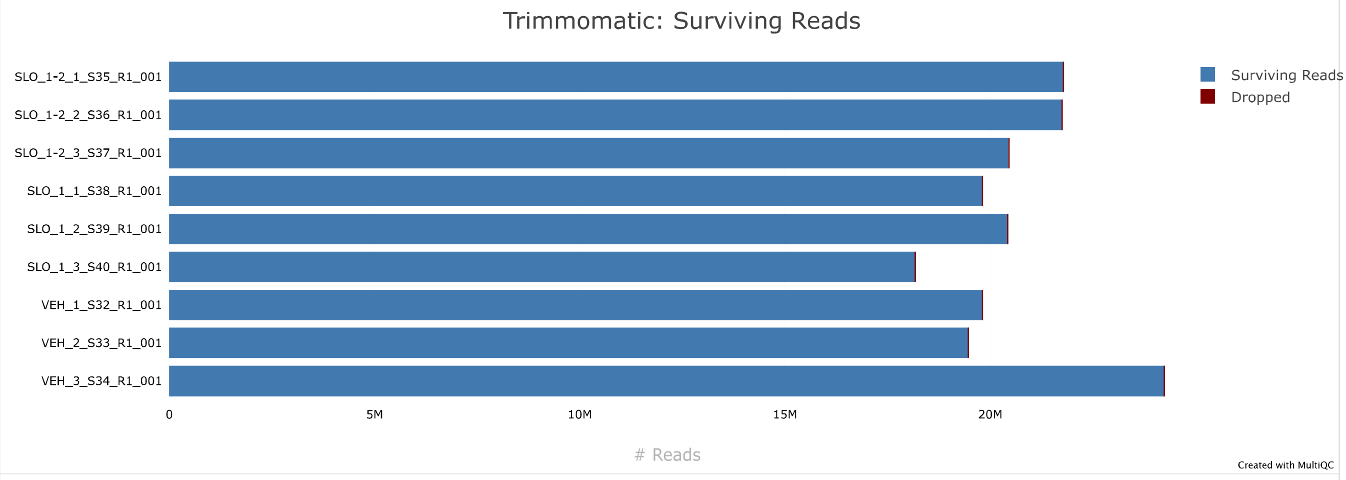

FastQ Screen *Version:* `0.14.0`

FastQ Screen allows you to screen a library of sequences in FastQ format against a set of sequence databases so you can see if the composition of the library matches with what you expect. *DOI: 10.12688/f1000research.15931.2.*

Mapped Reads

Percentages

Export Plot

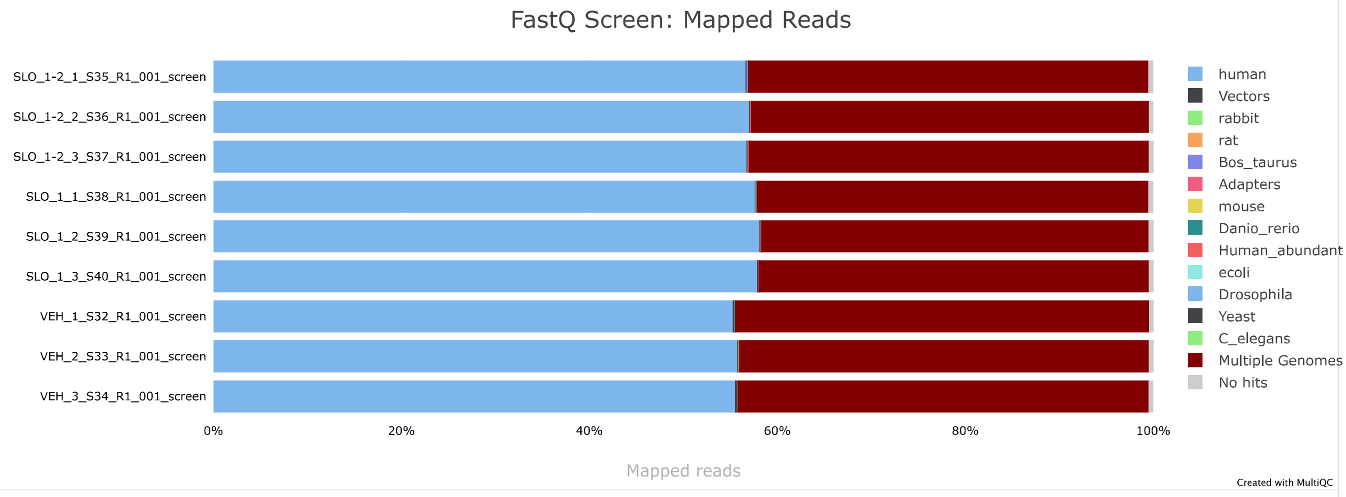

FastQC *Version:* `0.11.9`

FastQC is a quality control tool for high throughput sequence data, written by Simon Andrews at the Babraham Institute in Cambridge.

Sequence Counts

Sequence counts for each sample. Duplicate read counts are an estimate only.

Percentages

Export Plot

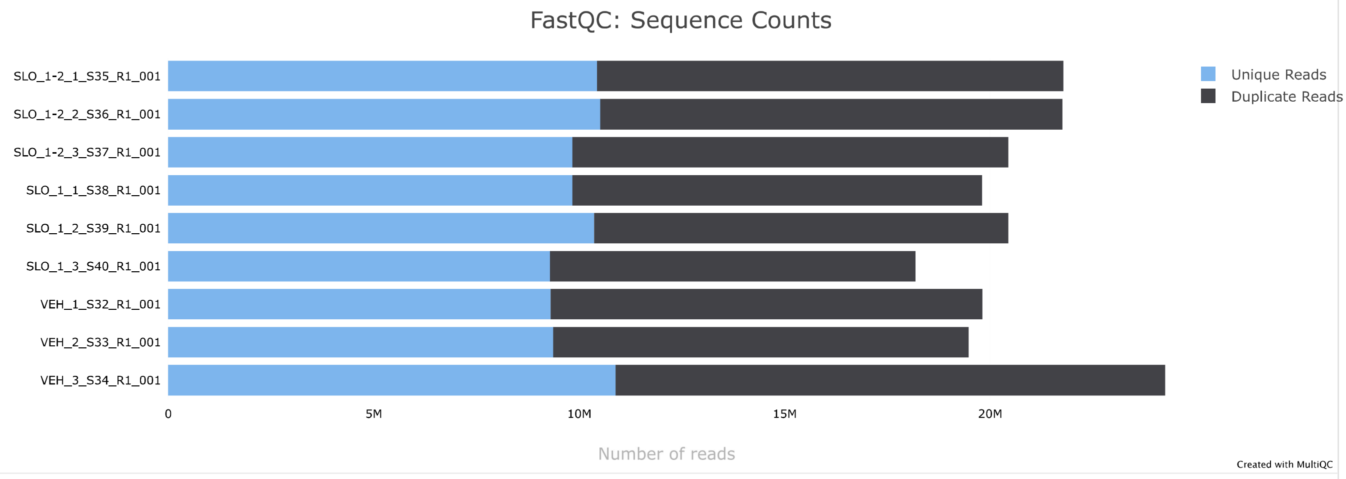

Sequence Quality Histograms

9

The mean quality value across each base position in the read.

Export Plot

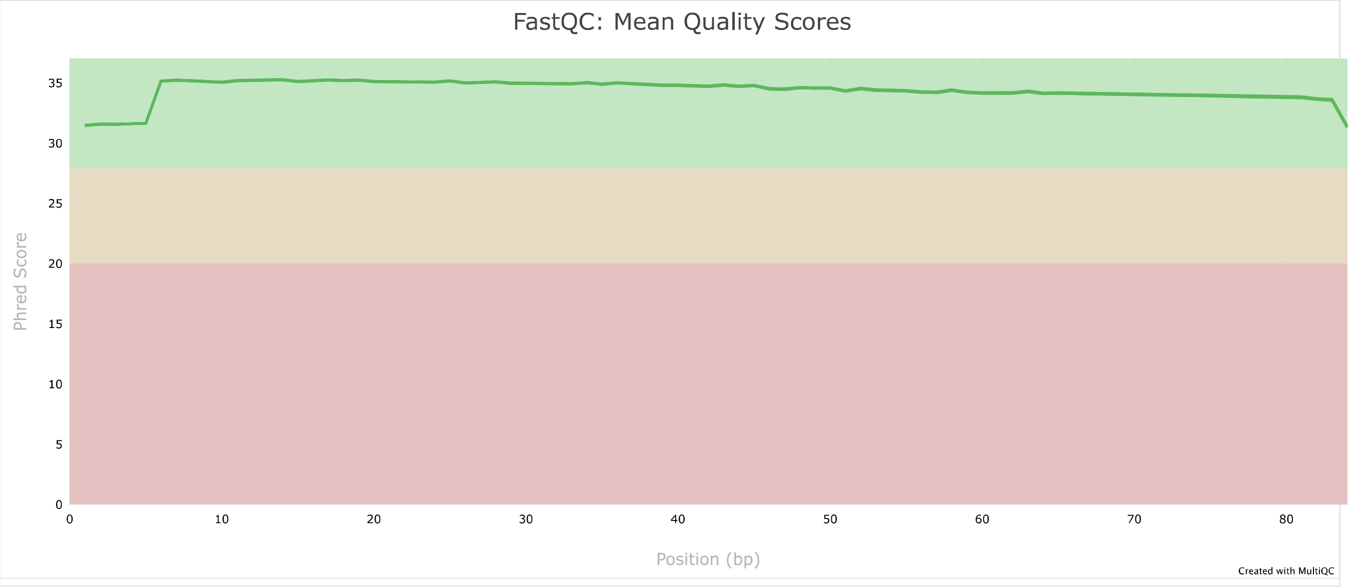

Per Sequence Quality Scores

9

The number of reads with average quality scores. Shows if a subset of reads has poor quality.

Export Plot

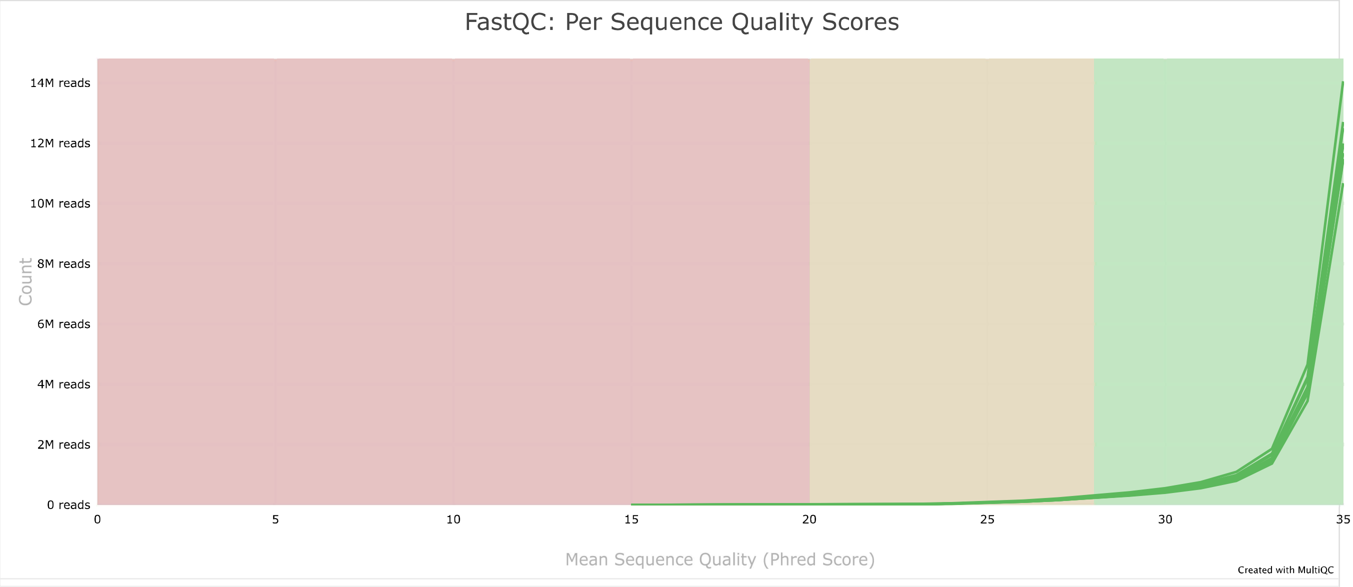

Per Base Sequence Content 9

The proportion of each base position for which each of the four normal DNA bases has been called.

Click a sample row to see a line plot for that dataset.

Rollover for sample name

Position: - %T: - %C: - %A: - %G: -

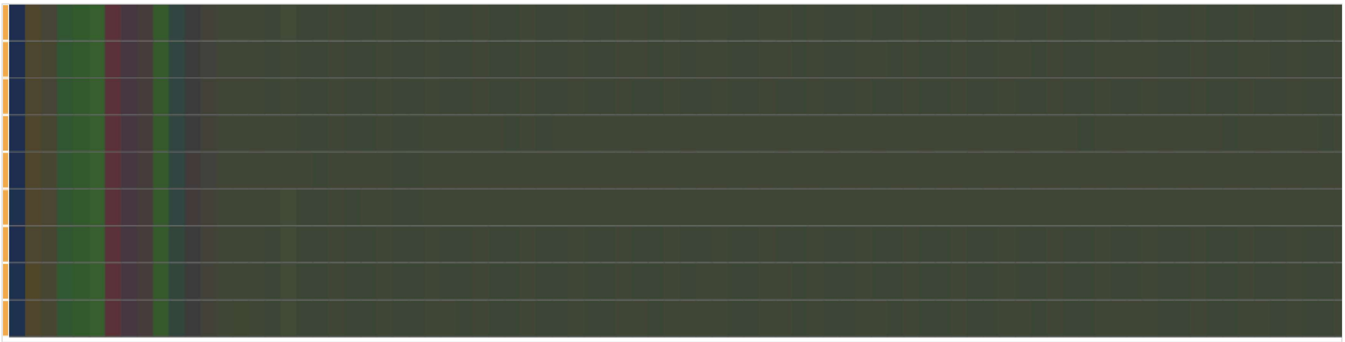

Per Sequence GC Content 9

The average GC content of reads. Normal random library typically have a roughly normal distribution of GC content.

Percentages Counts Export Plot

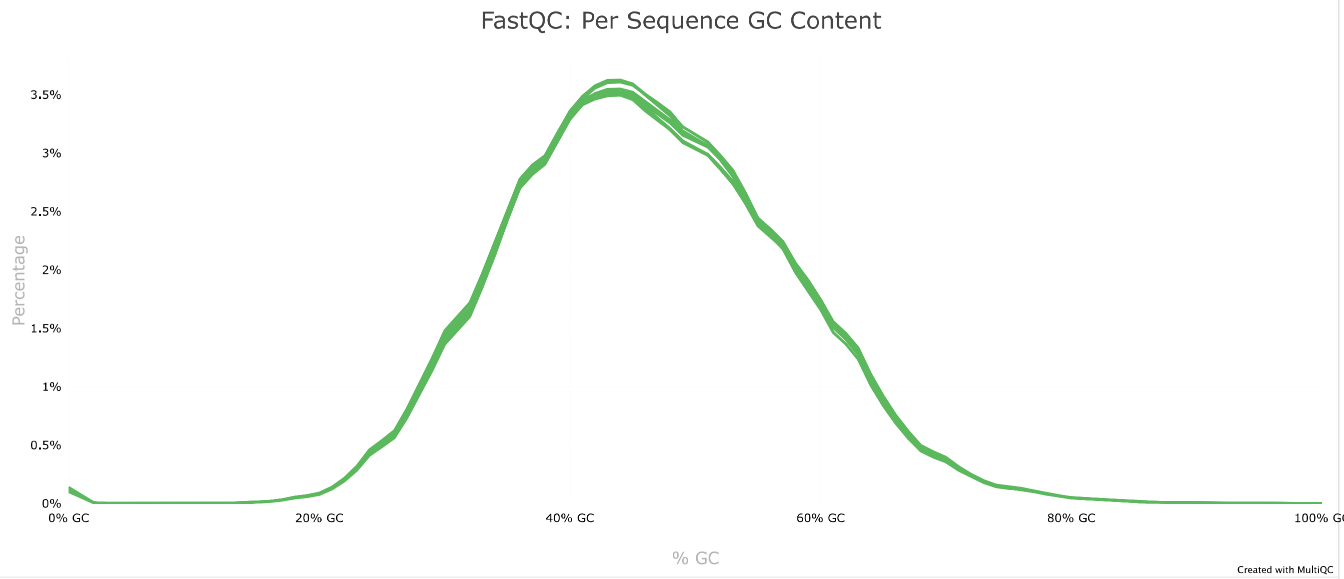

Per Base N Content 9

The percentage of base calls at each position for which an `N` was called.

Export Plot

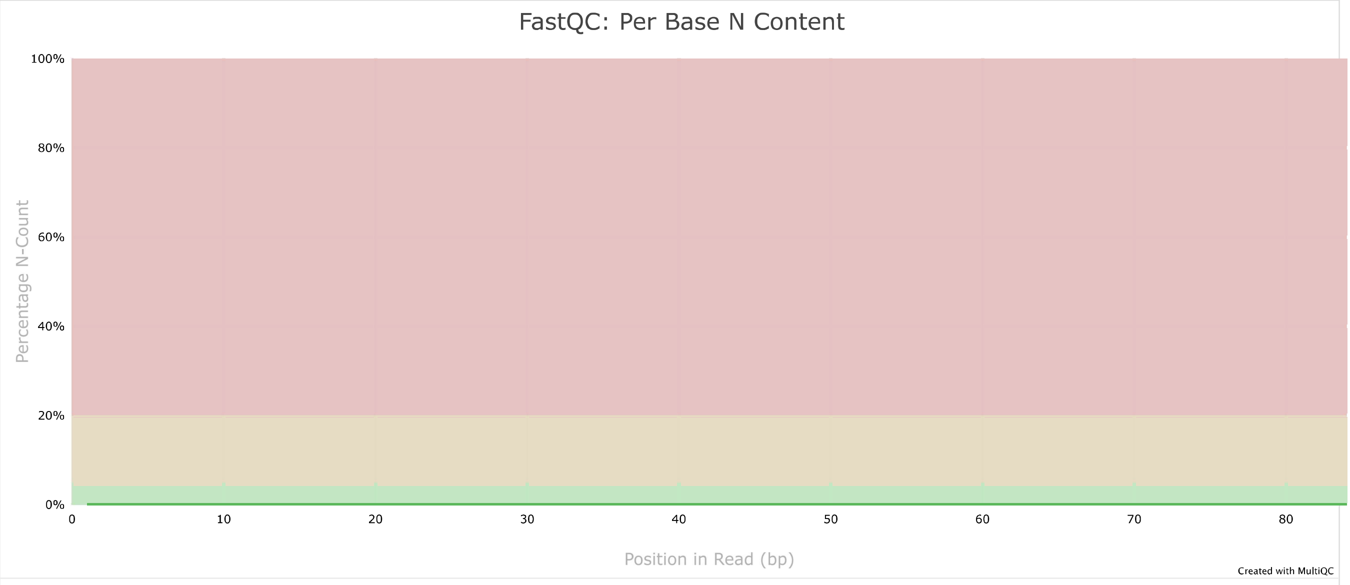

Sequence Length Distribution 9

All samples have sequences of a single length (84bp).

Sequence Duplication Levels 2

The relative level of duplication found for every sequence.

Export Plot

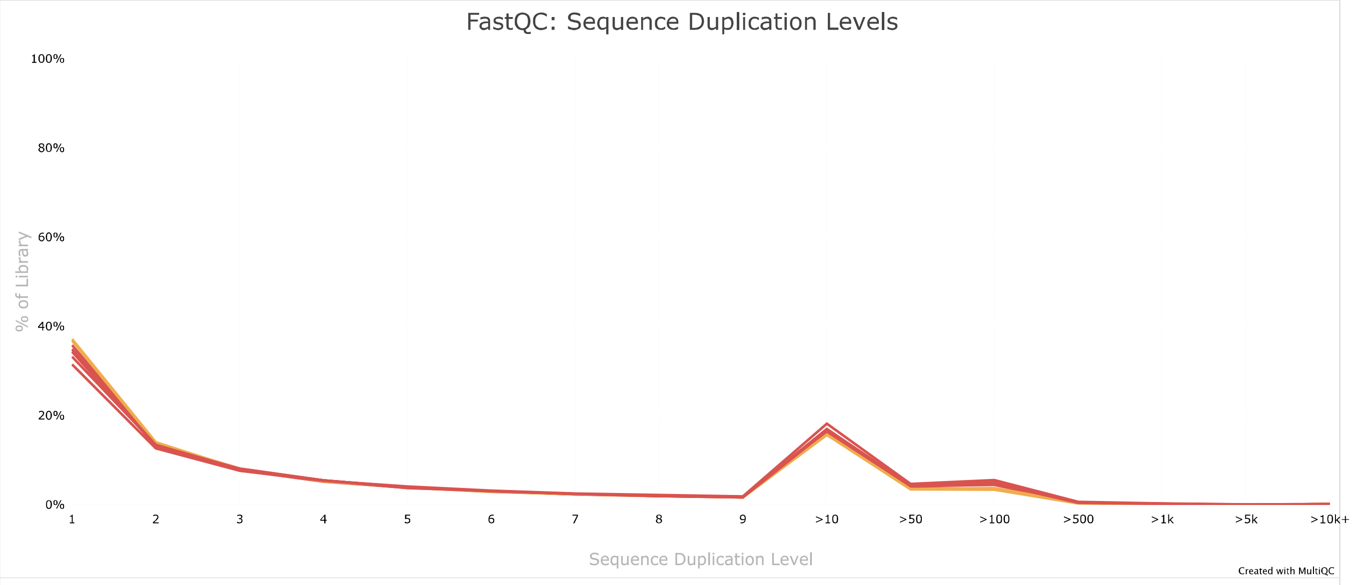

Overrepresented sequences by sample 9

The total amount of overrepresented sequences found in each library.

9 samples had less than 1% of reads made up of overrepresented sequences

Top overrepresented sequences

Top overrepresented sequences across all samples. The table shows 20 most overrepresented sequences across all samples, ranked by the number of samples they occur in.

Copy table | Configure columns | Scatter plot | Violin plot | Showing 1/1 rows and 3/3 columns. | Export as CSV

Overrepresented sequence	Samples	Occurrences	% of all reads
TTTTTTTTTTTTTTTTTTTTTTTTTTTTTT	9	226 467	0.1218 %

Adapter Content 9

The cumulative percentage count of the proportion of your library which has seen each of the adapter sequences at each position.

Export Plot

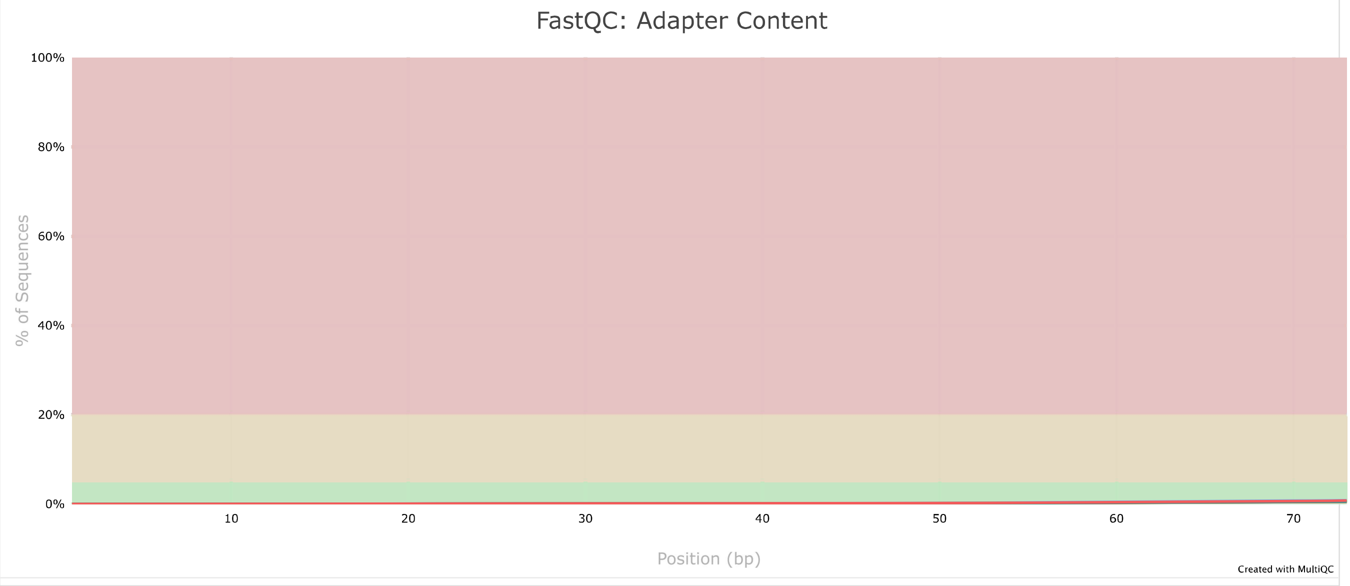

Status Checks

Status for each FastQC section showing whether results seem entirely normal (green), slightly abnormal (orange) or very unusual (red).

Min: 0 **Max:** 1 Export Plot

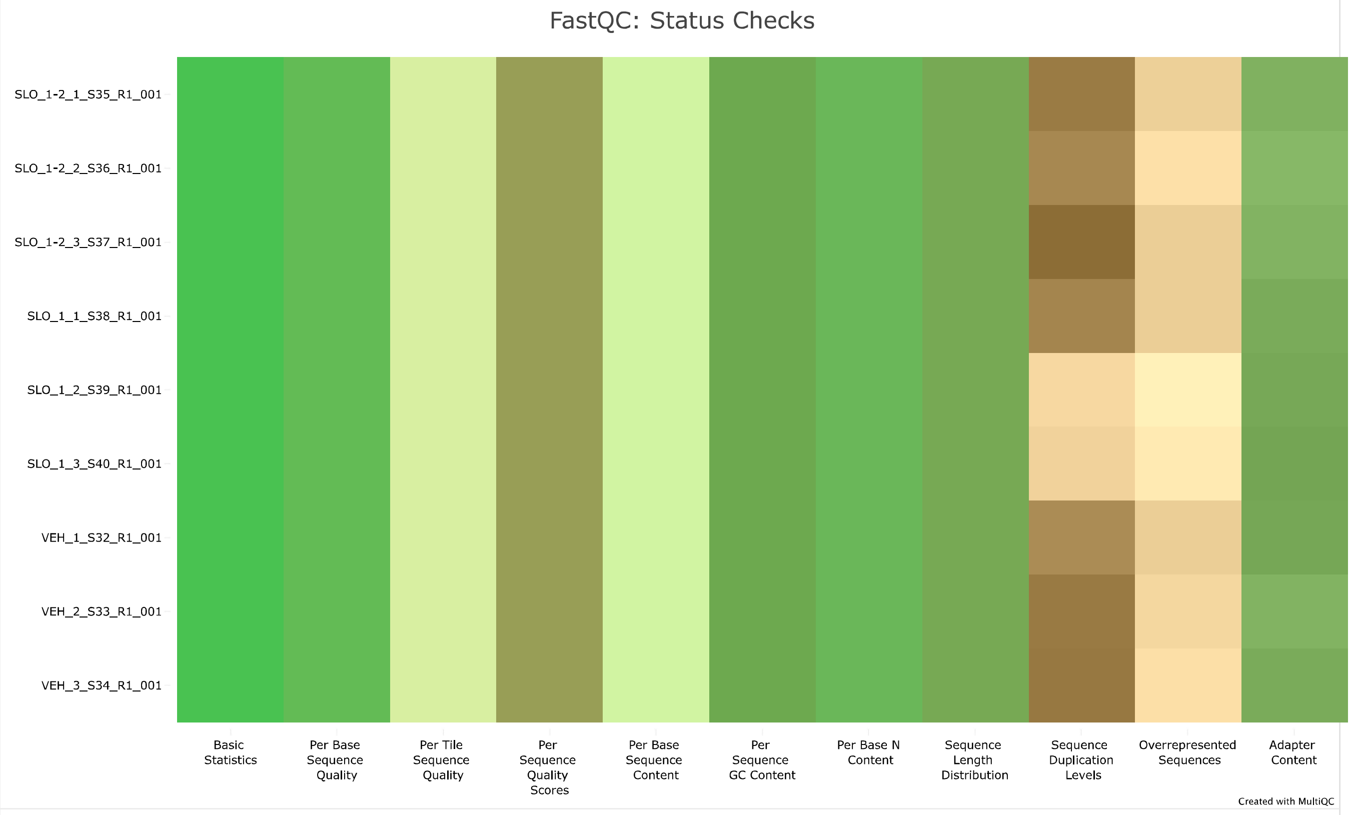

Software Versions

Software Versions lists versions of software tools extracted from file contents.

Copy table

Software	Version
FastQ Screen	0.14.0
FastQC	0.11.9

MultiQC v1.21 - Written by Phil Ewels, available on GitHub.
This report uses HighCharts, jQuery, jQuery UI, Bootstrap, FileSaver.js and clipboard.js.

seqera
