# Supplementary material for: Chemically Induced Senescence Prompts Functional Changes in Human Microglia-Like Cells
Source: J Immunol Res. 2025 Feb 24;2025:3214633. doi: 10.1155/jimr/3214633 (PMC11876530; doi:10.1155/jimr/3214633)
Supplement: Supporting Information 3 — File 2: RNA-Seq_Raw reads and mapping statistics for RNA-seq libraries. [file 3214633.f3.pdf]

Library	Reads	Filtered	%	Mapped	%	rRNA	%	Coverage (X)†
VEH_1	19,802,471	19,801,544	100	19,626,716	99.1	146,277	0.7	29.46
KAPA RNA HyperPrep PolyA								
VEH_2	19,470,155	19,469,188	100	19,289,257	99.1	165,012	0.8	28.6
KAPA RNA HyperPrep PolyA								
VEH_3	24,250,908	24,249,174	100	24,018,005	99	182,154	0.8	35.54
KAPA RNA HyperPrep PolyA								
SLO_1-2_1	21,779,590	21,777,880	100	21,563,493	99	225,098	1	30.88
KAPA RNA HyperPrep PolyA								
SLO_1-2_2	21,754,903	21,754,000	100	21,549,169	99.1	237,685	1.1	30.53
KAPA RNA HyperPrep PolyA								
SLO_1-2_3	20,442,181	20,441,141	100	20,249,462	99.1	190,983	0.9	29.2
KAPA RNA HyperPrep PolyA								
SLO_1_1	19,801,262	19,800,237	100	19,607,734	99	199,372	1	27.67
KAPA RNA HyperPrep PolyA								
SLO_1_2	20,434,850	20,433,874	100	20,237,521	99	200,762	1	27.92
KAPA RNA HyperPrep PolyA								
SLO_1_3	18,177,919	18,177,439	100	18,009,810	99.1	183,790	1	25.07
KAPA RNA HyperPrep PolyA								
