# Supplementary material for: Chemically Induced Senescence Prompts Functional Changes in Human Microglia-Like Cells
Source: J Immunol Res. 2025 Feb 24;2025:3214633. doi: 10.1155/jimr/3214633 (PMC11876530; doi:10.1155/jimr/3214633)
Supplement: Supporting Information 4 — File 3: RNA-Seq_list of differentially expressed genes. File 4: RNA-Seq_gProfiler-GSEA. [file 3214633.f4.pdf]

baseMean	log2FoldChang	lfcSE	stat	pvalue	padj	gene.symbol
1079.163143	0.749606754	0.13665669	5.485328	4.13E-08	3.49E-07	MTND2P28
142.8925854	0.587397347	0.17459547	3.3643333	0.0007673	0.003044294	MTCO1P12
3626.786177	0.910524428	0.15804023	5.761346	8.34E-09	7.78E-08	MTATP6P1
235.6722005	0.373702048	0.12609153	2.9637363	0.0030393	0.010430863	LINC01128
282.5757691	2.58516302	0.13058546	19.796714	3.18E-87	1.49E-84	ISG15
1602.175127	0.140059591	0.05616092	2.4938979	0.0126349	0.036571361	SDF4
375.1180266	0.530849075	0.11001785	4.8251175	1.40E-06	9.35E-06	ACAP3
480.2336829	0.201273065	0.08330037	2.4162324	0.015682	0.043964341	MXRA8
531.8039034	-0.23288863	0.08373101	-2.7813903	0.0054127	0.017385703	AURKAIP1
296.0022034	0.481973179	0.11017859	4.3744723	1.22E-05	6.93E-05	ATAD3B
279.4889661	0.619712937	0.12099141	5.1219581	3.02E-07	2.25E-06	MIB2
502.2775033	-0.37468563	0.07999473	-4.6838787	2.81E-06	1.79E-05	SLC35E2B
579.4654931	0.1863476	0.07850688	2.3736467	0.0176134	0.048510762	NADK
7800.258292	-0.13223472	0.03979291	-3.3230723	0.0008903	0.003482374	GNB1
4.489368519	3.196180421	1.25555142	2.5456388	0.0109078	0.032226745	CALML6
1878.469409	0.12983454	0.0542129	2.3949014	0.0166248	0.046249544	SKI
362.1884196	-0.25945922	0.10000406	-2.5944868	0.0094732	0.02844257	PANK4
26.92168331	1.501101662	0.36804247	4.0786099	4.53E-05	0.000231114	TNFRSF14
760.866646	1.193787668	0.14998949	7.959142	1.73E-15	3.37E-14	MEGF6
72.63687958	-0.86517888	0.20064329	-4.312025	1.62E-05	8.96E-05	TP73
297.237952	0.554690353	0.11579898	4.7901142	1.67E-06	1.10E-05	AJAP1
35.78624308	0.971472226	0.36234913	2.6810392	0.0073394	0.022734761	CHD5
1037.405365	-0.51255597	0.06016631	-8.5189857	1.61E-17	3.79E-16	RPL22
678.6389729	0.921677182	0.08188748	11.255411	2.18E-29	1.31E-27	KLHL21
904.731433	-0.35713601	0.06668832	-5.3553006	8.54E-08	6.90E-07	PHF13
284.8968458	-1.00712398	0.11816793	-8.5228197	1.56E-17	3.67E-16	PER3
38.04164737	1.483069819	0.31378406	4.7264027	2.29E-06	1.47E-05	TNFRSF9
2357.026414	-0.17701267	0.05034207	-3.5161979	0.0004378	0.001838421	PARK7
1132.321994	0.31131488	0.06371995	4.8856737	1.03E-06	7.01E-06	ERRFI1
32442.61562	-0.4806064	0.03134757	-15.331538	4.71E-53	7.72E-51	ENO1
42.39313676	2.336497682	0.39257254	5.9517604	2.65E-09	2.64E-08	MIR34AHG
9.830873312	2.185154519	0.69250346	3.155442	0.0016026	0.005903064	LNCTAM34A
4539.724166	0.234164585	0.04517768	5.1831916	2.18E-07	1.66E-06	CLSTN1
731.1299387	-0.36357909	0.07492789	-4.8523865	1.22E-06	8.22E-06	LZIC
1236.462784	-0.57656054	0.06049531	-9.5306654	1.56E-21	5.22E-20	UBE4B
3037.884917	-0.12683387	0.04678117	-2.7112166	0.0067037	0.021046586	KIF1B
4767.916497	-0.23031248	0.04046225	-5.6920332	1.26E-08	1.14E-07	TARDBP
1978.070229	-0.11721945	0.04718026	-2.4845021	0.0129733	0.037411491	EXOSC10
9.45016379	1.766223434	0.7384933	2.391658	0.0167725	0.046585707	DISP3
454.7297622	-0.35446609	0.08595922	-4.1236539	3.73E-05	0.00019327	MAD2L2
104.9208248	1.742585003	0.21010219	8.2939878	1.10E-16	2.38E-15	DRAXIN
356.6048312	-0.3147936	0.10559852	-2.9810418	0.0028727	0.009919813	CLCN6
687.55639	1.301955198	0.08232629	15.814573	2.47E-56	4.52E-54	NPPB
2096.004778	0.530837471	0.05416566	9.8002593	1.12E-22	4.16E-21	PLOD1
19.02135005	1.197472172	0.44357425	2.6995981	0.0069423	0.021701799	TNFRSF1B
1238.820119	-0.59396171	0.07734368	-7.6795122	1.60E-14	2.82E-13	VPS13D
381.8155156	0.660212196	0.09559546	6.9063132	4.97E-12	6.74E-11	TMEM51

1248.487516	0.794548327	0.05827113	13.635369	2.47E-42	2.73E-40	FBLIM1
2123.778431	-0.40308354	0.05679053	-7.0977243	1.27E-12	1.82E-11	SPEN
2051.136515	0.331455757	0.05721114	5.7935532	6.89E-09	6.49E-08	EPHA2
962.7652806	0.350018893	0.06418306	5.453447	4.94E-08	4.14E-07	NECAP2
13.29090432	1.40537461	0.53595007	2.6222118	0.0087361	0.026467069	LINC01772
926.1369402	-0.16142882	0.06421483	-2.5138872	0.0119409	0.034846721	SDHB
31.40972682	1.615341291	0.40908384	3.9486802	7.86E-05	0.000382898	PADI2
21.23139765	1.655836982	0.40604335	4.0779808	4.54E-05	0.000231672	PADI3
3080.239686	0.105322759	0.04178285	2.5207174	0.0117116	0.034252354	RCC2
10.56467117	1.808287381	0.60166467	3.0054738	0.0026517	0.009228062	KLHDC7A
506.5389761	0.774645224	0.09033278	8.5754605	9.87E-18	2.37E-16	IFFO2
5132.4668	-0.39608608	0.13073464	-3.029695	0.002448	0.008598437	UBR4
146.6045503	0.502304188	0.15479696	3.2449228	0.0011748	0.004457822	SLC66A1
4437.943276	0.18132068	0.04113432	4.4080148	1.04E-05	6.01E-05	CAPZB
339.0747532	0.617385337	0.10080744	6.1244027	9.10E-10	9.62E-09	NBL1
945.6200876	0.345091122	0.06614206	5.2174231	1.81E-07	1.40E-06	CAMK2N1
513.3781673	0.385572547	0.08433698	4.5718087	4.84E-06	2.95E-05	MUL1
1002.925752	0.261568465	0.0647023	4.0426453	5.29E-05	0.000265326	PINK1
156.6662634	-0.32989135	0.13723706	-2.4038065	0.0162254	0.045263996	SH2D5
6933.543505	-0.18705794	0.03783991	-4.9434036	7.68E-07	5.34E-06	HP1BP3
3350.757332	-0.20793483	0.04506258	-4.6143567	3.94E-06	2.45E-05	EIF4G3
2953.4831	0.280255502	0.04482088	6.2527885	4.03E-10	4.47E-09	ECE1
2224.462569	-0.24028821	0.04837027	-4.9676843	6.78E-07	4.76E-06	USP48
2741.639368	0.301049497	0.06581777	4.5739854	4.79E-06	2.92E-05	HSPG2
2423.15694	-0.30985461	0.04771874	-6.4933529	8.39E-11	1.00E-09	KDM1A
2840.990474	0.300520683	0.04716155	6.3721549	1.86E-10	2.15E-09	LUZP1
5634.721696	-0.56033771	0.03775941	-14.839683	8.11E-50	1.18E-47	HNRNPR
125.6823647	-0.71021396	0.16219245	-4.3788348	1.19E-05	6.79E-05	E2F2
1134.103764	0.558204109	0.07245312	7.7043492	1.32E-14	2.35E-13	ID3
9887.766175	-0.3328759	0.03656875	-9.1027422	8.81E-20	2.56E-18	RPL11
855.9974126	0.276757311	0.06601805	4.192146	2.76E-05	0.000146639	PITHD1
353.8719913	0.288535705	0.10186904	2.8324179	0.0046197	0.015098161	FUCA1
17.30014638	2.452602587	0.5323718	4.6069356	4.09E-06	2.53E-05	GRHL3
451.3507544	-0.33352528	0.09395706	-3.5497628	0.0003856	0.001636614	RCAN3
702.7075623	-0.23147531	0.07472815	-3.0975652	0.0019512	0.007036692	SYF2
1177.266675	0.446397351	0.06123804	7.2895438	3.11E-13	4.79E-12	RSRP1
2504.966216	0.273348265	0.04904455	5.5734688	2.50E-08	2.18E-07	TMEM50A
751.676662	0.286734992	0.07337935	3.9075704	9.32E-05	0.000448107	MACO1
509.7407693	0.502229457	0.08441925	5.9492292	2.69E-09	2.68E-08	LDLRAP1
885.4290076	-0.39070735	0.06479315	-6.0300716	1.64E-09	1.68E-08	SELENON
8077.253882	-0.30134648	0.03845641	-7.8360541	4.65E-15	8.60E-14	STMN1
18.33904558	1.492758741	0.45737521	3.2637509	0.0010995	0.00419849	EXTL1
155.0625633	-0.34424417	0.14131144	-2.4360672	0.0148479	0.041984621	PDIK1L
4963.933924	-0.41335921	0.0393844	-10.495507	9.06E-26	4.26E-24	HMGN2
2512.191353	-0.4096114	0.05079008	-8.0647915	7.34E-16	1.49E-14	ARID1A
134.7132036	0.409917753	0.15434424	2.6558669	0.0079105	0.024240229	PIGV
23.15802461	1.142055453	0.36739899	3.1084883	0.0018805	0.006812789	SFN
503.3725566	0.293597692	0.08217477	3.5728447	0.0003531	0.001510659	GPN2

2502.55416	-0.19587016	0.04696985	-4.1701251	3.04E-05	0.000160281	NUDC
592.208791	0.23397336	0.0807674	2.8968785	0.003769	0.012609111	TRNP1
683.4979156	0.474706031	0.07262197	6.536672	6.29E-11	7.61E-10	SLC9A1
569.8466269	-0.33616825	0.08007078	-4.1983883	2.69E-05	0.000142873	WDTC1
2276.535868	-0.44679142	0.04552121	-9.815017	9.70E-23	3.64E-21	WASF2
5.808562827	4.085327506	1.1948298	3.4191711	0.0006281	0.002544385	LINC02574
743.7024326	3.148858919	0.41355098	7.6141977	2.65E-14	4.59E-13	IFI6
909.4454538	0.347595016	0.06690855	5.195076	2.05E-07	1.56E-06	STX12
42.98501886	0.713698403	0.26557106	2.6874103	0.0072008	0.022361189	XKR8
17.12173958	1.442967521	0.45354964	3.1814985	0.0014652	0.005434941	PTAFR
344.4973569	0.67576217	0.1031283	6.5526354	5.65E-11	6.85E-10	SESN2
1387.717198	-0.33503943	0.05248989	-6.3829326	1.74E-10	2.02E-09	RCC1
672.7270348	-0.35236458	0.07644724	-4.6092518	4.04E-06	2.50E-05	SNHG3
55.14082669	-0.76639424	0.23850573	-3.2133159	0.0013121	0.004919739	SNORA73B
271.8479012	-0.29926552	0.11102394	-2.6955044	0.0070282	0.021911198	TRNAU1AP
288.8350478	0.633998896	0.10830014	5.854091	4.80E-09	4.60E-08	SNHG12
414.9482006	-1.07224835	0.09185329	-11.673488	1.74E-31	1.18E-29	EPB41
1984.253367	-0.39278475	0.05509587	-7.1291144	1.01E-12	1.47E-11	SRSF4
149.4454621	0.947958948	0.17175791	5.5191576	3.41E-08	2.91E-07	PTPRU
9.705661383	1.995399066	0.64603081	3.0887057	0.0020103	0.007219991	LAPTM5
1435.476603	0.189137039	0.06769205	2.7940806	0.0052048	0.016786094	SDC3
2709.199443	-0.13214986	0.0461911	-2.8609374	0.0042239	0.013935438	PUM1
1090.579439	-0.37358279	0.05939514	-6.2897875	3.18E-10	3.57E-09	SNRNP40
299.465851	1.081082638	0.11020659	9.8096006	1.02E-22	3.83E-21	SERINC2
246.1815859	0.354281347	0.12773881	2.7734823	0.005546	0.017774545	TINAGL1
648.3157938	-0.20043655	0.07833335	-2.558764	0.0105045	0.031162283	PEF1
113.4016295	0.87232294	0.16829945	5.1831598	2.18E-07	1.66E-06	COL16A1
173.5823794	0.531743355	0.15306376	3.473999	0.0005128	0.002121567	ADGRB2
432.3698585	1.068502449	0.09158758	11.666455	1.89E-31	1.28E-29	SPOCD1
3194.888373	-0.17206443	0.04862538	-3.5385724	0.0004023	0.001703834	PTP4A2
4586.08906	-0.37940381	0.04128644	-9.1895508	3.94E-20	1.17E-18	KHDRBS1
2609.387717	-0.24360275	0.05000817	-4.8712588	1.11E-06	7.52E-06	KPNA6
2729.727308	-0.22926309	0.04280775	-5.3556442	8.53E-08	6.89E-07	EIF3I
1619.906197	0.432926662	0.05624246	7.6975059	1.39E-14	2.47E-13	MARCKSL1
185.1744534	-0.35096506	0.13686089	-2.5643927	0.0103357	0.030713776	ZBTB8A
4170.43077	-0.23030729	0.04080459	-5.6441513	1.66E-08	1.48E-07	RBBP4
609.0207854	-0.21240748	0.07394371	-2.8725565	0.0040717	0.013502296	SYNC
687.4887148	-0.29704229	0.0780749	-3.8045813	0.000142	0.000658706	KIAA1522
2384.202446	-0.26308246	0.04464233	-5.8931166	3.79E-09	3.68E-08	YARS1
591.4798717	-0.26362199	0.08336703	-3.1621852	0.0015659	0.005777846	S100PBP
429.4542456	0.804746888	0.10172577	7.9109438	2.55E-15	4.87E-14	RNF19B
3046.643047	-0.43667761	0.04169982	-10.471931	1.16E-25	5.43E-24	AK2
817.7042206	0.193825171	0.06567939	2.9510806	0.0031666	0.010806106	PHC2
167.0547331	-0.34267293	0.13450542	-2.5476515	0.0108451	0.032068668	ZSCAN20
226.5637588	-0.3110185	0.11690309	-2.6604813	0.0078029	0.02396971	ZMYM6
943.6274748	-0.27647337	0.07585122	-3.644943	0.0002675	0.001174192	ZMYM1
6003.683425	-0.15077381	0.05601861	-2.6914948	0.0071133	0.022130748	SFPQ
1777.487861	-0.48376219	0.05282812	-9.1572852	5.32E-20	1.57E-18	ZMYM4

451.8633061	0.312413247	0.08743631	3.5730381	0.0003529	0.001510287	NCDN
3597.24499	0.153208638	0.06004816	2.5514295	0.0107282	0.031760886	CLSPN
207.079425	-0.61368374	0.12425597	-4.9388672	7.86E-07	5.46E-06	AGO4
1252.607079	-0.3437642	0.05716247	-6.0138093	1.81E-09	1.85E-08	AGO1
607.0765813	-0.39472482	0.08327894	-4.7397917	2.14E-06	1.38E-05	AGO3
4277.219844	-0.3043696	0.04028631	-7.5551623	4.18E-14	7.10E-13	THRAP3
532.9473771	-0.2842691	0.08584528	-3.3114122	0.0009283	0.003614533	STK40
906.9328025	-0.25188087	0.06387777	-3.9431693	8.04E-05	0.000390822	MEAF6
1717.641198	-0.23550471	0.04801318	-4.905001	9.34E-07	6.40E-06	CDCA8
258.1605067	0.2827938	0.11219276	2.5206064	0.0117153	0.034257394	YRDC
408.6798528	-0.28093368	0.09008036	-3.1187008	0.0018165	0.006604429	INPP5B
3147.412992	-0.19789209	0.04164238	-4.75218	2.01E-06	1.31E-05	SF3A3
2184.842243	0.218786992	0.06031069	3.6276654	0.000286	0.001245545	AKIRIN1
8387.253201	-0.62337858	0.08421034	-7.4026371	1.34E-13	2.15E-12	MACF1
3156.510024	-0.30006302	0.04238177	-7.0800018	1.44E-12	2.05E-11	PABPC4
21.9689066	1.60292419	0.48778499	3.2861286	0.0010157	0.003916592	HPCAL4
85.39680653	1.116344402	0.20831485	5.3589286	8.37E-08	6.78E-07	MFSD2A
8719.933378	0.225796023	0.04148451	5.4428992	5.24E-08	4.37E-07	CAP1
2249.902921	-0.20623536	0.04741351	-4.3497173	1.36E-05	7.66E-05	PPT1
1241.765107	-0.32243392	0.05876996	-5.4863725	4.10E-08	3.47E-07	RLF
2031.397846	0.147669532	0.06215003	2.3760169	0.0175007	0.048276724	ZMPSTE24
129.2065004	0.436624529	0.15797076	2.763958	0.0057105	0.018250325	ZNF684
27.73562183	0.890777398	0.35981719	2.4756388	0.0132998	0.038235014	CITED4
3697.12206	0.361810572	0.04686772	7.719824	1.16E-14	2.09E-13	CTPS1
69.97472493	1.310101042	0.22705378	5.7700032	7.93E-09	7.42E-08	SLFNL1-AS1
253.062639	-0.57007969	0.11226787	-5.0778527	3.82E-07	2.79E-06	SCMH1
11436.81309	-0.2048585	0.04171782	-4.9105749	9.08E-07	6.24E-06	YBX1
1031.965711	0.522097954	0.06427674	8.1226577	4.56E-16	9.44E-15	P3H1
115.4198183	0.433242994	0.18106921	2.3926928	0.0167252	0.046491671	ERMAP
2188.010836	-0.19343977	0.04989114	-3.8772371	0.0001056	0.000501705	SLC2A1
1955.601509	-0.2345797	0.05673042	-4.13499	3.55E-05	0.000184652	EBNA1BP2
30.06147685	0.752178111	0.30338407	2.4792934	0.0131643	0.037895568	TIE1
2246.099106	-0.23184017	0.0491972	-4.7124663	2.45E-06	1.57E-05	CDC20
283.3587863	0.301645862	0.11614393	2.5971729	0.0093995	0.02825036	HYI
3709.24017	-0.50590538	0.04438996	-11.396842	4.34E-30	2.71E-28	PTPRF
361.9237269	0.252623525	0.09846657	2.5655766	0.0103004	0.030624852	IPO13
277.2008363	0.282323251	0.11152808	2.5314096	0.0113605	0.033388418	DPH2
505.0296103	0.315730043	0.08947344	3.5287571	0.0004175	0.001762283	ATP6V0B
422.1998214	-0.31450021	0.09257582	-3.3972177	0.0006807	0.002733996	ERI3
3358.823385	-0.10660694	0.04242788	-2.5126623	0.0119824	0.034950338	KIF2C
10907.03665	-0.33905831	0.03951794	-8.5798588	9.50E-18	2.28E-16	RPS8
154.6744751	0.960102289	0.1643517	5.8417544	5.17E-09	4.93E-08	PLK3
60.79464482	1.187810216	0.24640007	4.820657	1.43E-06	9.54E-06	BTBD19
448.6222135	-0.21408668	0.08379425	-2.5549089	0.0106216	0.03147196	AKR1A1
1565.164289	-0.33500496	0.05620528	-5.9603818	2.52E-09	2.52E-08	GPBP1L1
303.7645999	-0.49118506	0.10554742	-4.653691	3.26E-06	2.05E-05	IPP
1503.305315	0.143595767	0.0556695	2.5794335	0.0098963	0.029579893	MAST2
465.9234925	0.83836003	0.096304	8.7053503	3.17E-18	7.98E-17	PIK3R3

721.3746099	-0.27568805	0.07114629	-3.8749461	0.0001066	0.000506034	RAD54L
505.2555165	0.417331536	0.08645822	4.8269736	1.39E-06	9.26E-06	MKNK1
693.8444921	-0.37936	0.070805	-5.3578136	8.42E-08	6.82E-07	ATPAF1
1387.887214	-0.52322859	0.05795725	-9.0278368	1.75E-19	4.95E-18	FAF1
846.6731342	-0.73963161	0.07450998	-9.9266116	3.19E-23	1.22E-21	CDKN2C
1298.715506	-0.32749696	0.05435212	-6.0254675	1.69E-09	1.72E-08	EPS15
1708.901204	-0.24275754	0.05457752	-4.4479403	8.67E-06	5.06E-05	OSBPL9
2248.95208	-0.59215617	0.04469167	-13.249812	4.52E-40	4.57E-38	NRDC
6328.342537	-0.85846865	0.04273903	-20.086291	9.73E-90	4.97E-87	RAB3B
914.8495845	-0.48091704	0.06425171	-7.484891	7.16E-14	1.18E-12	BTF3L4
813.6474466	-0.51960361	0.06481798	-8.0163495	1.09E-15	2.18E-14	ZFYVE9
561.630695	0.197959104	0.08164341	2.4246797	0.0153219	0.043121516	CC2D1B
588.369679	-0.34671075	0.07433239	-4.6643295	3.10E-06	1.96E-05	ORC1
1137.805348	-0.23797893	0.05909578	-4.0270041	5.65E-05	0.00028246	PRPF38A
1326.595516	-0.56164507	0.05572813	-10.078305	6.89E-24	2.81E-22	TUT4
1308.135696	-0.35021423	0.05971014	-5.8652388	4.48E-09	4.31E-08	ZYG11B
1343.027219	-0.14574705	0.0539242	-2.7028137	0.0068755	0.021510641	SCP2
725.7427036	-0.17655311	0.07323895	-2.4106452	0.0159243	0.044550217	MAGOH
291.7073978	-0.71490962	0.10887658	-6.566239	5.16E-11	6.29E-10	LRP8
1844.346313	-0.62965072	0.05278987	-11.927491	8.51E-33	6.30E-31	NDC1
779.5361001	-0.18662134	0.07221565	-2.5842229	0.0097599	0.02922605	LRRC42
2388.280796	0.17819683	0.05215751	3.416513	0.0006343	0.002566365	TMEM59
377.8699269	-0.24128301	0.09206288	-2.6208502	0.0087711	0.026545258	TCEANC2
200.6743396	-0.48255936	0.12903741	-3.7396857	0.0001843	0.000839028	CYB5RL
1349.738481	-0.38438694	0.05309929	-7.2390228	4.52E-13	6.84E-12	MRPL37
1719.1225	-0.57304299	0.06549185	-8.749837	2.14E-18	5.42E-17	USP24
54.09917028	1.434191499	0.2854381	5.0245272	5.05E-07	3.63E-06	PLPP3
132.1080231	-0.72972033	0.15584121	-4.6824606	2.83E-06	1.80E-05	OMA1
3467.628346	-0.37666182	0.04632335	-8.1311447	4.25E-16	8.82E-15	JUN
17.07876259	-1.03250455	0.42206036	-2.4463434	0.0144313	0.041006725	NFIA
131.262581	-1.06078283	0.15385965	-6.8944834	5.41E-12	7.28E-11	PATJ
3975.12402	-0.27288176	0.04444402	-6.1398981	8.26E-10	8.77E-09	USP1
1775.634594	-0.49957408	0.0520726	-9.5937992	8.49E-22	2.88E-20	DOCK7
279.9886389	-0.64723656	0.10457834	-6.1890115	6.05E-10	6.56E-09	ATG4C
210.4879643	-0.43130618	0.12716126	-3.3918048	0.0006943	0.002782796	ALG6
533.3581764	-0.58557018	0.0903775	-6.4791586	9.22E-11	1.09E-09	ITGB3BP
133.7527752	-0.61220327	0.15021794	-4.0754337	4.59E-05	0.000234085	EFCAB7
268.3572819	1.008718084	0.12532069	8.0490949	8.34E-16	1.69E-14	ROR1
252.9687837	0.714660225	0.12593002	5.6750586	1.39E-08	1.25E-07	CACHD1
2435.317374	-0.14464565	0.05268956	-2.7452433	0.0060466	0.019216091	AK4
1505.230853	-0.26401397	0.05464443	-4.8314894	1.36E-06	9.07E-06	DNAJC6
1710.013925	0.179012713	0.05465038	3.2755987	0.0010544	0.004044048	LEPROT
425.244907	-0.52682685	0.10067527	-5.2329324	1.67E-07	1.29E-06	LEPR
34.60321341	0.917417712	0.31266864	2.9341533	0.0033446	0.011342125	PDE4B
7.201536301	1.938062017	0.72276154	2.6814681	0.00733	0.022713564	SGIP1
2094.936203	-0.18626784	0.05363418	-3.4729319	0.0005148	0.002129514	MIER1
8539.736046	-0.30412718	0.0360036	-8.4471316	2.99E-17	6.82E-16	SERBP1
1665.730296	0.497237679	0.05466282	9.0964517	9.33E-20	2.70E-18	GADD45A

11471.24693	-0.27415505	0.04404857	-6.2239269	4.85E-10	5.31E-09	GNG12
3483.146721	-0.22458424	0.0495888	-4.5289307	5.93E-06	3.54E-05	DEPDC1
47.43655564	1.42807982	0.28545044	5.0028993	5.65E-07	4.01E-06	LRRC7
970.1279656	-0.2359407	0.0620798	-3.8006034	0.0001443	0.000668125	LRRC40
4459.10483	-0.12029084	0.04997872	-2.4068409	0.0160912	0.044959121	SRSF11
4.781128756	3.726352376	1.36133437	2.7372793	0.006195	0.019615893	PTGER3
571.5057116	-0.71740003	0.08279502	-8.6647727	4.52E-18	1.12E-16	NEGR1
177.0758975	-0.34748275	0.13934831	-2.4936273	0.0126445	0.03659315	FPGT
610.1119097	-0.38430071	0.08091284	-4.7495639	2.04E-06	1.32E-05	CRYZ
596.3844883	-0.35831442	0.07959018	-4.501993	6.73E-06	3.99E-05	TYW3
1386.9008	-0.35579703	0.05890841	-6.039834	1.54E-09	1.59E-08	PIGK
1398.967131	-0.29022662	0.05265084	-5.5122889	3.54E-08	3.02E-07	ZZZ3
1498.030047	-0.29558782	0.05553565	-5.322488	1.02E-07	8.19E-07	USP33
2400.844446	0.482961765	0.06204369	7.7842208	7.01E-15	1.28E-13	NEXN
3333.978532	-0.27673452	0.04256656	-6.5012183	7.97E-11	9.52E-10	FUBP1
153.1566374	1.0504583	0.14696673	7.1475924	8.83E-13	1.29E-11	PTGFR
47.33251021	4.115429278	0.71350504	5.7679049	8.03E-09	7.50E-08	IFI44L
204.8384763	3.47154343	0.33870634	10.24942	1.19E-24	5.14E-23	IFI44
784.6244126	0.213947255	0.0762936	2.8042621	0.0050432	0.016325587	PRKACB
4379.677544	-0.1286458	0.04759273	-2.7030559	0.0068705	0.021500507	DDAH1
544.6818537	0.647780192	0.09663909	6.7030869	2.04E-11	2.57E-10	ENSG00000282057
18309.10701	-0.39516458	0.04028091	-9.8102205	1.02E-22	3.81E-21	CCN1
30.27427319	1.661759332	0.36235413	4.5860091	4.52E-06	2.77E-05	CLCA2
1441.402258	-0.14728674	0.05758458	-2.5577463	0.0105353	0.031248291	HS2ST1
1997.290049	-0.43233777	0.05056393	-8.5503204	1.23E-17	2.91E-16	PKN2
398.7227602	0.307279003	0.09301263	3.3036268	0.0009544	0.003708111	GTF2B
311.8542634	-0.84567637	0.10003687	-8.4536471	2.82E-17	6.46E-16	KYAT3
446.8123263	0.574727295	0.08383848	6.855173	7.12E-12	9.41E-11	GBP3
841.4645771	2.110211308	0.08388629	25.155617	1.23E-139	1.94E-136	GBP1
12.05348755	2.021369702	0.64752663	3.1216781	0.0017982	0.006544851	GBP2
470.1914437	-0.32032996	0.0949761	-3.3727428	0.0007442	0.002960932	LRRC8C
1446.993582	-0.1881664	0.05782887	-3.2538486	0.0011385	0.004334287	ZNF326
56.95945117	-0.68507085	0.22661891	-3.0230083	0.0025028	0.008773008	BTBD8
536.1670397	-0.3966008	0.08259941	-4.8014965	1.57E-06	1.04E-05	RPAP2
11729.2255	-0.40307604	0.03463665	-11.63727	2.66E-31	1.79E-29	RPL5
1282.928577	0.142541669	0.06013953	2.3701827	0.0177793	0.048890261	FNBP1L
8292.016765	-0.16161621	0.04217344	-3.8321798	0.000127	0.00059407	CNN3
93.9149635	-0.59416551	0.17657985	-3.3648546	0.0007658	0.003039242	ALG14
1008.980387	-0.29793208	0.06869234	-4.3371952	1.44E-05	8.07E-05	TLCD4
412.1265445	-0.2294413	0.09124005	-2.5146995	0.0119134	0.034784057	DPYD
708.666406	0.193771156	0.07297543	2.6552931	0.007924	0.024277227	SNX7
7.779601792	1.934256454	0.76836421	2.5173693	0.0118235	0.034550561	PLPPR4
13.11720966	2.082537123	0.6022245	3.4580744	0.0005441	0.002238772	PALMD
783.0873136	-0.49152804	0.07490096	-6.5623733	5.30E-11	6.44E-10	AGL
357.7310882	-0.37197226	0.09362077	-3.9731812	7.09E-05	0.000348831	TRMT13
883.3905817	-0.39306468	0.07295689	-5.3876291	7.14E-08	5.83E-07	DBT
86.31763952	0.796685966	0.2176454	3.660477	0.0002517	0.001111698	RTCA-AS1
471.324178	-0.45859348	0.08430198	-5.4398898	5.33E-08	4.43E-07	EXTL2

312.8083547	-0.50803133	0.10083935	-5.0380268	4.70E-07	3.39E-06	DPH5
9466.482419	0.886592915	0.09210237	9.6261686	6.20E-22	2.15E-20	COL11A1
291.874319	-0.32485013	0.10643495	-3.0521	0.0022725	0.008056706	PRMT6
91.57136457	0.696926141	0.19617579	3.5525593	0.0003815	0.001619711	HENMT1
1751.412716	0.15386081	0.05035213	3.0556959	0.0022454	0.007976977	PRPF38B
1032.419483	-0.54731006	0.0638127	-8.5768208	9.75E-18	2.34E-16	GPSM2
708.4914828	0.352113611	0.07451785	4.7252251	2.30E-06	1.48E-05	WDR47
1095.691383	0.364089586	0.06975746	5.2193639	1.80E-07	1.38E-06	TAF13
800.8875585	-0.19468141	0.06702587	-2.904571	0.0036776	0.012338976	TMEM167B
129.3935115	1.563852637	0.17703817	8.8334206	1.02E-18	2.69E-17	ELAPOR1
752.8371977	-0.45899819	0.07703433	-5.9583587	2.55E-09	2.55E-08	CELSR2
607.5626659	-0.29032749	0.08059076	-3.6024909	0.0003152	0.001358706	PSRC1
3562.266409	0.178320799	0.05293211	3.3688586	0.0007548	0.003000227	SORT1
15.54543163	1.323540382	0.54977721	2.4074122	0.016066	0.044896057	SYPL2
129.1897335	0.516786565	0.16057825	3.218285	0.0012896	0.004845738	AMIGO1
3126.667163	-0.26706937	0.04514331	-5.9160337	3.30E-09	3.22E-08	GNAI3
507.1332153	-0.22629159	0.07873308	-2.8741616	0.004051	0.013445436	AMPD2
398.2938986	0.622024315	0.10180512	6.1099513	9.97E-10	1.05E-08	GSTM3
165.8531849	1.309775341	0.14772526	8.8662921	7.56E-19	2.02E-17	CSF1
2799.076729	-0.45808382	0.04965488	-9.225353	2.83E-20	8.55E-19	AHCYL1
18.47095151	2.207590705	0.47613933	4.6364384	3.54E-06	2.21E-05	SLC6A17
322.707415	-0.42164293	0.10822586	-3.8959537	9.78E-05	0.0004682	RBM15
743.1748314	-0.25792178	0.07011473	-3.6785674	0.0002345	0.001042368	CEPT1
48.01132779	0.829626817	0.2656979	3.122444	0.0017936	0.00653058	DENND2D
1947.422518	-0.17526944	0.04974712	-3.523208	0.0004264	0.001796545	ATP5PB
626.4180704	0.706641147	0.0789539	8.9500473	3.55E-19	9.79E-18	INKA2
102.527293	0.442007413	0.18513345	2.387507	0.0169631	0.047017528	INKA2-AS1
1036.193084	0.242679167	0.06770433	3.5843963	0.0003379	0.001449633	DDX20
1500.63197	-0.15639015	0.06019061	-2.5982481	0.0093701	0.028171792	CTTNBP2NL
17.34822029	1.503590838	0.4632091	3.2460304	0.0011703	0.004442453	WNT2B
1269.993609	0.199903084	0.06543991	3.0547581	0.0022524	0.007998694	MOV10
930.3698057	0.625335056	0.06734586	9.2854271	1.61E-20	4.96E-19	RHOC
3415.348587	0.32617556	0.04614412	7.0686271	1.56E-12	2.21E-11	SLC16A1
365.4302688	0.234633203	0.09709282	2.4165866	0.0156668	0.043935758	SLC16A1-AS1
560.000815	-0.25738109	0.07866326	-3.2719351	0.0010681	0.004089601	MAGI3
480.8976234	-0.57045831	0.08164524	-6.9870371	2.81E-12	3.87E-11	RSBN1
16.56407706	1.138489322	0.46406003	2.4533234	0.0141543	0.040371222	PTPN22
2096.307125	-0.21805031	0.05038934	-4.3273102	1.51E-05	8.40E-05	HIPK1
2087.817762	-0.16201127	0.05546075	-2.9211878	0.003487	0.011763116	TRIM33
157.3272833	0.439162707	0.14653014	2.9970811	0.0027258	0.009463235	DENND2C
2710.570907	-0.23186079	0.04862792	-4.7680592	1.86E-06	1.22E-05	NRAS
16624.87834	-0.39408604	0.03706639	-10.631898	2.12E-26	1.05E-24	CSDE1
1497.820649	-0.19498851	0.05620188	-3.4694302	0.0005216	0.002155416	SIKE1
338.6498236	0.761713332	0.11298148	6.741931	1.56E-11	1.99E-10	TSPAN2
189.826396	0.407044956	0.13074382	3.1133016	0.0018501	0.006712433	NGF
477.9395857	-0.26795045	0.08678356	-3.0875716	0.002018	0.007246103	VANGL1
8.474226613	1.666884694	0.64227568	2.5952792	0.0094514	0.028386882	SLC22A15
4750.077001	-0.11995316	0.03980569	-3.0134678	0.0025828	0.00901726	ATP1A1

216.3607501	0.30070612	0.12532653	2.3993811	0.0164228	0.045782733	CD58
46.52710293	0.769139386	0.26770285	2.8731087	0.0040645	0.01348386	NAP1L4P1
363.4485751	0.491091913	0.09958688	4.9312911	8.17E-07	5.65E-06	IGSF3
602.0581513	-0.37458697	0.08260274	-4.5348011	5.77E-06	3.46E-05	PTGFRN
1554.685703	-0.26030511	0.05239142	-4.9684682	6.75E-07	4.74E-06	TTF2
2108.8039	-0.2145745	0.05701564	-3.7634322	0.0001676	0.000768191	MAN1A2
28.12888357	1.546703262	0.42460817	3.6426602	0.0002698	0.001182866	TENT5C
1123.155138	-0.24478463	0.06873408	-3.5613283	0.000369	0.001573087	WDR3
287.7377966	-0.2858369	0.10907514	-2.6205504	0.0087788	0.026563992	WARS2
121.9589487	0.464829016	0.16881845	2.7534255	0.0058975	0.018773178	ZNF697
1188.380368	-0.58606356	0.05703096	-10.276235	9.02E-25	3.93E-23	PHGDH
99.2675477	0.510667016	0.18304171	2.7898942	0.0052725	0.016982632	PDE4DIPP2
38.72526756	0.840726603	0.2770521	3.0345434	0.002409	0.008475122	PDZK1
1138.463489	-0.33630153	0.05860718	-5.7382313	9.57E-09	8.84E-08	RNF115
932.9831311	-0.27325828	0.0661023	-4.1338697	3.57E-05	0.000185444	PIAS3
1072.493216	-0.20607488	0.0622441	-3.3107534	0.0009305	0.003622242	RBM8A
952.7188223	-0.52383282	0.06555664	-7.9905381	1.34E-15	2.65E-14	LIX1L
287.8119126	-0.53877145	0.10897159	-4.9441461	7.65E-07	5.33E-06	BCL9
102.3563204	1.077511224	0.18552391	5.8079372	6.32E-09	5.98E-08	GJA5
373.2471925	1.043866218	0.11294092	9.2425862	2.41E-20	7.33E-19	H2BC21
449.0162444	-0.25089251	0.09200957	-2.7268089	0.006395	0.02017949	SV2A
907.7582669	-0.20814269	0.06374023	-3.265484	0.0010928	0.004179305	VPS45
3235.357451	-0.8478065	0.04310643	-19.667748	4.07E-86	1.82E-83	ANP32E
1012.963117	-0.35482501	0.06282175	-5.648124	1.62E-08	1.45E-07	APH1A
1246.584773	-0.15238767	0.05915863	-2.5759164	0.0099975	0.029820961	RPRD2
465.1938018	-0.32979922	0.08745651	-3.7710081	0.0001626	0.000746615	TARS2
391.9010921	0.496087662	0.09703853	5.1122752	3.18E-07	2.35E-06	ECM1
792.0602518	0.407772736	0.07176794	5.6818232	1.33E-08	1.21E-07	ADAMTSL4
2449.731428	-0.24295841	0.04468297	-5.437383	5.41E-08	4.49E-07	ENSA
717.1226539	-0.51422453	0.07601087	-6.7651449	1.33E-11	1.71E-10	GOLPH3L
4.861385342	2.746199312	0.96207702	2.8544485	0.0043112	0.014182957	CTSS
853.2877557	-0.42180414	0.06977548	-6.0451632	1.49E-09	1.54E-08	ARNT
451.3155569	-0.43917668	0.09568049	-4.5900337	4.43E-06	2.73E-05	SETDB1
1357.572962	0.198489726	0.05750354	3.4517828	0.0005569	0.0022846	CDC42SE1
556.1492537	-0.31283046	0.08365604	-3.7394844	0.0001844	0.000839446	VPS72
3468.793749	-0.1995328	0.04356983	-4.5796095	4.66E-06	2.85E-05	PIP5K1A
1741.020235	-0.24000668	0.05324823	-4.507318	6.57E-06	3.90E-05	PSMD4
462.4020092	-0.27092423	0.0889339	-3.0463549	0.0023163	0.008188898	ZNF687
956.9677558	0.154999805	0.06331051	2.4482478	0.0143553	0.04081728	RFX5
1492.969946	-0.41098765	0.06182846	-6.6472244	2.99E-11	3.71E-10	POGZ
2027.273465	0.534516991	0.05313599	10.059415	8.35E-24	3.37E-22	TUFT1
860.1286936	-0.45959225	0.07206377	-6.377577	1.80E-10	2.08E-09	SNX27
854.3291987	-0.18452196	0.06636033	-2.7806064	0.0054257	0.017421299	MRPL9
464.2150845	-0.32652245	0.0873977	-3.736053	0.0001869	0.000849865	TDRKH
4030.307999	0.314714022	0.04267835	7.3740915	1.65E-13	2.64E-12	S100A10
1577.189156	0.462039707	0.0527107	8.7655775	1.86E-18	4.75E-17	S100A11
81.25293987	-0.67419868	0.20522388	-3.2851863	0.0010191	0.003925362	FLG-AS1
1670.050293	0.512103174	0.05774299	8.8686647	7.40E-19	1.98E-17	S100A6

912.3530565	-0.22125913	0.0634096	-3.4893633	0.0004842	0.002018159	CHTOP
373.7482502	-0.30123952	0.09427013	-3.1954928	0.0013959	0.005202549	SNAPIN
4047.991871	-0.28024092	0.04421864	-6.3376201	2.33E-10	2.67E-09	ILF2
1052.454511	-0.28404612	0.06359117	-4.4667544	7.94E-06	4.67E-05	INTS3
1165.789585	-0.35171096	0.06143054	-5.7253435	1.03E-08	9.48E-08	GATAD2B
547.001209	-0.32181196	0.08322161	-3.866928	0.0001102	0.000521392	DENND4B
1684.44911	-0.16070064	0.05642923	-2.8478264	0.0044019	0.01446776	C1orf43
4060.080178	-0.29566038	0.04357327	-6.7853616	1.16E-11	1.50E-10	UBAP2L
1054.684688	-0.15249238	0.05895979	-2.5863793	0.009699	0.029060275	HAX1
930.9758085	-0.19018779	0.06255918	-3.0401259	0.0023648	0.008331392	ATP8B2
27.43305224	2.599236504	0.45714336	5.6858235	1.30E-08	1.18E-07	ENSG00000287064
3526.903878	-0.11342073	0.04399928	-2.5777859	0.0099436	0.02969577	SHC1
57.46390534	0.583052725	0.24448506	2.3848194	0.0170875	0.047264475	MUC1
281.1696103	0.468631195	0.11805337	3.9696553	7.20E-05	0.00035358	THBS3
744.7997974	0.478704722	0.07557098	6.3345044	2.38E-10	2.71E-09	GBA
246.107129	-0.62599804	0.1241161	-5.0436488	4.57E-07	3.30E-06	FAM189B
673.8134393	-0.41309191	0.08300315	-4.976822	6.46E-07	4.55E-06	CLK2
1727.872946	-0.41162835	0.05357401	-7.6833588	1.55E-14	2.74E-13	FDPS
1903.163722	-0.54003904	0.05830805	-9.2618258	2.01E-20	6.16E-19	ASH1L
259.8340986	0.78599214	0.12602376	6.2368569	4.46E-10	4.92E-09	SYT11
730.4024072	0.327106004	0.07239519	4.518339	6.23E-06	3.71E-05	RIT1
1346.02922	-0.17247571	0.05647815	-3.0538484	0.0022593	0.008018074	ARHGEF2
128.2737097	-0.88621631	0.17250203	-5.1374255	2.79E-07	2.08E-06	MEX3A
4626.039602	0.150941944	0.0491896	3.0685742	0.0021508	0.007673967	LMNA
100.9085043	-0.48183824	0.17704184	-2.7216065	0.0064965	0.020466468	PMF1
1858.866318	0.170337195	0.04974884	3.4239434	0.0006172	0.002506684	SMG5
5380.838795	-0.16301523	0.04344612	-3.752124	0.0001753	0.000801369	CCT3
2198.8	-0.1902105	0.06060354	-3.1386039	0.0016975	0.006217428	IQGAP3
131.4038464	0.860551692	0.17738345	4.8513639	1.23E-06	8.26E-06	CRABP2
559.0316182	-0.33275502	0.08179162	-4.0683266	4.74E-05	0.000240986	MRPL24
4469.945033	-0.37385925	0.03997688	-9.3518861	8.61E-21	2.72E-19	HDGF
189.1593296	0.395489503	0.1283957	3.0802395	0.0020683	0.007408541	PEAR1
2592.038435	-0.25172242	0.05278382	-4.7689313	1.85E-06	1.21E-05	KIRREL1
638.3738679	0.782576746	0.1537086	5.091301	3.56E-07	2.61E-06	IFI16
19.03646946	1.609790622	0.46435867	3.4666966	0.0005269	0.002174354	CADM3
6.123000034	2.06273639	0.76013687	2.7136381	0.0066549	0.020904696	SLAMF8
19.97155146	1.048195199	0.41940666	2.4992335	0.0124462	0.036115445	ENSG00000272668
6643.25429	0.297620485	0.03802933	7.8260765	5.03E-15	9.29E-14	TAGLN2
3.461689752	4.063660666	1.39560905	2.9117471	0.0035941	0.012088286	LINC01133
9422.175738	0.099673742	0.03564653	2.7961697	0.0051712	0.016690344	PEA15
923.9771053	-0.19357028	0.06335983	-3.0550949	0.0022499	0.00799135	PEX19
5814.342828	-0.17986158	0.03659657	-4.9147109	8.89E-07	6.11E-06	COPA
158.8817042	-0.49806158	0.14974249	-3.3261205	0.0008806	0.003448391	VANGL2
7.770278225	2.942977767	0.90721346	3.243975	0.0011787	0.004470732	ARHGAP30
57.40818903	1.876823801	0.28862166	6.502713	7.89E-11	9.43E-10	NECTIN4
299.9958491	-0.31568587	0.11388341	-2.7720093	0.0055711	0.017845311	NIT1
346.0443974	-0.31400191	0.09875218	-3.1796959	0.0014743	0.005460707	USP21
513.3363409	-0.19266844	0.07899816	-2.4388978	0.0147321	0.041738641	B4GALT3

3.168143643	3.726717266	1.41617301	2.631541	0.0084999	0.025842757	ADAMTS4
1044.248504	-0.39339089	0.06642575	-5.9222653	3.18E-09	3.12E-08	NDUFS2
20.39133992	2.935467965	0.74408703	3.94506	7.98E-05	0.000388511	PCP4L1
784.8332821	-0.24378054	0.07467709	-3.2644624	0.0010967	0.004191279	SDHC
383.7138011	-0.29500953	0.09571303	-3.0822297	0.0020546	0.007365252	DUSP12
1279.775362	-0.28823955	0.06337999	-4.5478006	5.42E-06	3.26E-05	ATF6
7367.5638	-0.19684809	0.04955753	-3.9721126	7.12E-05	0.000350249	UHMK1
5705.323774	-0.28479579	0.04290486	-6.6378448	3.18E-11	3.94E-10	UAP1
62.78863353	0.783275275	0.23576817	3.3222266	0.000893	0.003490592	DDR2
46.1103742	1.58147702	0.28584353	5.5326669	3.15E-08	2.72E-07	CCDC190
85.98862333	1.158632487	0.20870826	5.5514452	2.83E-08	2.46E-07	RGS5
1454.080335	-0.36102094	0.05713673	-6.3185435	2.64E-10	2.99E-09	NUF2
1252.270749	-0.22561581	0.05876025	-3.8395988	0.0001232	0.000577801	UCK2
1431.675624	-0.18152597	0.05371726	-3.3792857	0.0007267	0.002895323	POGK
209.8418986	-0.44058008	0.12191614	-3.6137963	0.0003017	0.001307592	TADA1
531.6741036	-0.58014477	0.08485253	-6.8370945	8.08E-12	1.06E-10	POU2F1
1032.171511	0.21186042	0.0676057	3.1337656	0.0017258	0.00631288	CREG1
2282.219924	0.301101911	0.05483032	5.4915221	3.98E-08	3.38E-07	MPZL1
964.0121531	0.234086757	0.06847774	3.4184357	0.0006298	0.002550677	MPC2
2233.094472	-0.25086293	0.04807651	-5.2179936	1.81E-07	1.39E-06	DCAF6
1328.247954	-0.28801447	0.05715147	-5.0394935	4.67E-07	3.37E-06	TIPRL
1276.909444	-0.15052565	0.05838443	-2.5781815	0.0099322	0.029671988	ATP1B1
240.5484158	-0.70335582	0.11921068	-5.9001075	3.63E-09	3.53E-08	NME7
527.0467883	-0.30062541	0.08098154	-3.7122707	0.0002054	0.00092564	C1orf112
122.4340035	-0.66728568	0.16087855	-4.1477604	3.36E-05	0.000175649	METTL18
390.3578935	-0.61021173	0.09581386	-6.3687206	1.91E-10	2.19E-09	KIFAP3
7855.421199	-0.39703978	0.04632005	-8.5716608	1.02E-17	2.44E-16	PRRC2C
240.751021	-0.62713742	0.11635691	-5.3897738	7.05E-08	5.77E-07	VAMP4
117.2669313	0.534305176	0.19198443	2.7830652	0.0053848	0.017305793	DNM3
332.944401	-0.32345729	0.09690787	-3.3377813	0.0008445	0.003320345	PIGC
1417.912423	-0.54208441	0.05570767	-9.7308753	2.23E-22	8.05E-21	DARS2
9.17316606	-1.73460908	0.60617629	-2.8615588	0.0042156	0.013916071	GAS5-AS1
343.7225416	-0.60045641	0.11765447	-5.1035579	3.33E-07	2.46E-06	ZBTB37
384.7264431	-0.33394461	0.09483701	-3.5212478	0.0004295	0.001807252	RABGAP1L
1807.1135	-0.19146649	0.05502871	-3.4793924	0.0005026	0.002083782	CACYBP
656.3611322	-0.2074642	0.08259474	-2.511833	0.0120106	0.03502082	COP1
1433.634203	-0.16247602	0.05881445	-2.7625185	0.0057357	0.018317348	RASAL2
264.4371208	-0.34526543	0.10757991	-3.2093858	0.0013302	0.004979974	ENSG00000213058
2145.408146	-0.59868804	0.04556711	-13.138601	1.98E-39	1.92E-37	RALGPS2
1655.033968	-0.46092127	0.05085878	-9.0627665	1.27E-19	3.63E-18	FAM20B
1915.265736	0.178133353	0.06113585	2.91373	0.0035714	0.012017494	SOAT1
2836.159162	-0.20049625	0.05212244	-3.84664	0.0001197	0.000562211	TOR1AIP2
2068.438828	-0.33987086	0.11760852	-2.8898488	0.0038543	0.012864832	CEP350
118.4676794	0.686373504	0.16890763	4.0636027	4.83E-05	0.000245201	MR1
7488.906117	-0.32975314	0.04082949	-8.0763463	6.67E-16	1.36E-14	DHX9
9194.892879	0.386048667	0.03824701	10.093564	5.90E-24	2.41E-22	LAMC1
47.36769098	1.203548843	0.26530792	4.5364225	5.72E-06	3.43E-05	LAMC2
110.8343705	0.978068031	0.17676723	5.5330844	3.15E-08	2.71E-07	NMNAT2

1641.623557	-0.21548427	0.05228051	-4.1216945	3.76E-05	0.000194652	SMG7
85.26721279	1.517699989	0.22002067	6.8979882	5.27E-12	7.11E-11	NCF2
626.4448708	0.890631851	0.08889484	10.018937	1.26E-23	4.96E-22	RGL1
58.28482302	0.740799526	0.24736095	2.994812	0.0027461	0.009524408	COLGALT2
935.6713079	-0.59401495	0.06256508	-9.4943532	2.22E-21	7.33E-20	TSEN15
1399.438006	-0.51870875	0.06537462	-7.934406	2.12E-15	4.06E-14	NIBAN1
659.1872905	-0.41367076	0.07964869	-5.1936921	2.06E-07	1.57E-06	RNF2
615.1090372	-0.44841586	0.08084735	-5.5464508	2.92E-08	2.52E-07	TRMT1L
88.37450898	-0.96256604	0.18930152	-5.0848299	3.68E-07	2.69E-06	SWT1
12.58240963	1.81815266	0.59994774	3.0305184	0.0024413	0.008578081	ENSG00000279838
3728.036578	0.299112833	0.04321184	6.9220115	4.45E-12	6.06E-11	IVNS1ABP
46.01462394	0.900803545	0.32067901	2.8090505	0.0049688	0.016111724	HMCN1
6401.079227	-0.38110947	0.04026708	-9.4645411	2.95E-21	9.69E-20	TPR
1280.886565	-0.22482684	0.05677583	-3.9599042	7.50E-05	0.000366882	UCHL5
1952.786054	-0.18341295	0.05943584	-3.0858981	0.0020294	0.007284001	RO60
1823.846891	-0.22755237	0.0515215	-4.4166485	1.00E-05	5.79E-05	CDC73
6018.745085	-0.74081439	0.05003335	-14.806412	1.33E-49	1.91E-47	ASPM
660.1357689	-0.52251058	0.08035558	-6.5024806	7.90E-11	9.44E-10	ZBTB41
3713.452019	0.261511436	0.04844591	5.3980089	6.74E-08	5.53E-07	NEK7
1500.892567	-0.14620642	0.05788653	-2.5257415	0.0115454	0.033842371	ZNF281
2730.712738	-0.40973552	0.04476053	-9.1539473	5.49E-20	1.62E-18	KIF14
2483.932789	-0.14929278	0.05336046	-2.7978166	0.0051449	0.016617822	CAMSAP2
17.45770771	1.5774332	0.46592648	3.385584	0.0007103	0.002836838	INAVA
608.2480536	-0.21676096	0.07498084	-2.8908845	0.0038416	0.012829903	TMEM9
286.1724346	0.842701586	0.1135569	7.4209634	1.16E-13	1.88E-12	PHLDA3
7094.525407	0.344618055	0.03906664	8.8212875	1.13E-18	2.98E-17	CSRP1
2573.373308	-0.42370553	0.05839819	-7.2554571	4.00E-13	6.10E-12	NAV1
3219.269064	-0.35354636	0.04420575	-7.9977452	1.27E-15	2.52E-14	IPO9
205.0233961	1.197642741	0.17409511	6.8792441	6.02E-12	8.04E-11	LMOD1
1307.457278	-0.17906719	0.0550386	-3.2534839	0.00114	0.004338906	RNPEP
485.146051	0.318418458	0.0927579	3.4327906	0.0005974	0.002436408	ARL8A
980.9988986	-0.31567879	0.06696845	-4.7138438	2.43E-06	1.56E-05	UBE2T
456.6423598	0.412469715	0.08900795	4.6340771	3.59E-06	2.24E-05	RABIF
912.6804597	-0.20423864	0.07626525	-2.6780041	0.0074062	0.02291771	KLHL12
461.319941	-0.28446603	0.10118038	-2.8114743	0.0049315	0.015999782	PPFIA4
370.5284387	1.213448355	0.11491954	10.559112	4.61E-26	2.21E-24	BTG2
2255.043958	-0.37215726	0.05137053	-7.2445673	4.34E-13	6.58E-12	ATP2B4
791.1034496	-0.27542174	0.07108149	-3.8747324	0.0001067	0.00050634	SNRPE
480.1478705	0.477345814	0.08590123	5.5569148	2.75E-08	2.39E-07	SOX13
351.5827254	-0.50398777	0.10074573	-5.0025719	5.66E-07	4.02E-06	ETNK2
46.79666737	0.731073617	0.26668639	2.7413233	0.0061192	0.01940464	PLEKHA6
607.0476329	-0.51760081	0.07837606	-6.604068	4.00E-11	4.92E-10	MDM4
1164.010509	-0.23312747	0.06035812	-3.8624046	0.0001123	0.000529847	RBBP5
851.0557019	-0.20798714	0.06738587	-3.0865096	0.0020252	0.007270535	DSTYK
1544.308498	-0.59377197	0.06161626	-9.6366116	5.60E-22	1.94E-20	ELK4
14911.92539	-0.57525812	0.04760011	-12.085227	1.26E-33	9.73E-32	NUCKS1
84.12707986	0.584508299	0.19714739	2.964829	0.0030285	0.010399836	ENSG00000285417
9.887271046	1.858354275	0.64099717	2.8991614	0.0037416	0.01252489	RHEX

665.6371011	-0.37670058	0.07843534	-4.8026898	1.57E-06	1.04E-05	EIF2D
211.0387661	-0.41860283	0.12920923	-3.2397285	0.0011964	0.004528954	PFKFB2
21.25805458	2.268648388	0.448208	5.0615973	4.16E-07	3.03E-06	C4BPB
571.8309243	0.691065383	0.07789397	8.8718723	7.19E-19	1.93E-17	CD55
4038.335078	0.167242601	0.04606722	3.6304033	0.000283	0.001233024	CD46
52.60895596	1.177336748	0.25711222	4.5790773	4.67E-06	2.85E-05	CD34
267.7940492	0.940459995	0.12063693	7.7957886	6.40E-15	1.17E-13	PLXNA2
74.98994196	0.732378364	0.2058706	3.5574694	0.0003744	0.001594416	LAMB3
1421.196756	0.289808805	0.06043371	4.7954824	1.62E-06	1.07E-05	SERTAD4
249.9108827	0.357866996	0.11753851	3.0446788	0.0023293	0.008231324	HHAT
73.7854591	0.571474975	0.23231969	2.4598645	0.0138989	0.039753731	KCNH1
807.7088973	1.000034524	0.09336249	10.711309	9.01E-27	4.61E-25	SLC30A1
1690.544099	-0.32565284	0.04995293	-6.5191946	7.07E-11	8.51E-10	NEK2
1779.736353	-0.64096914	0.05843324	-10.969255	5.37E-28	3.01E-26	LPGAT1
2547.164715	-0.24724463	0.04753956	-5.2008188	1.98E-07	1.52E-06	DTL
821.9823033	-0.38689787	0.0724071	-5.3433696	9.12E-08	7.36E-07	PPP2R5A
429.8679831	1.068907611	0.10725579	9.9659666	2.15E-23	8.30E-22	ATF3
824.5963955	-0.29774639	0.06518301	-4.567853	4.93E-06	3.00E-05	NSL1
166.7117889	-0.78208134	0.13370628	-5.8492489	4.94E-09	4.73E-08	TATDN3
252.8529081	-0.36132927	0.11317583	-3.1926365	0.0014098	0.005248657	FLVCR1
104.0975264	-0.83809378	0.18287891	-4.5827798	4.59E-06	2.81E-05	VASH2
700.6371821	-0.23703962	0.07722729	-3.0693765	0.0021451	0.007655737	RPS6KC1
5023.105105	-0.40644289	0.05481649	-7.4146095	1.22E-13	1.97E-12	PTPN14
11978.1852	-0.53679621	0.07813714	-6.8699235	6.42E-12	8.53E-11	CENPF
975.0339381	-0.355013	0.06676923	-5.3170147	1.05E-07	8.41E-07	KCTD3
476.8979627	-0.26897735	0.08431613	-3.1901056	0.0014222	0.005288048	GPATCH2
1126.044749	-0.29023644	0.06380608	-4.5487267	5.40E-06	3.25E-05	RRP15
4457.174758	-0.23419636	0.05405152	-4.3328356	1.47E-05	8.22E-05	TGFB2
5312.795296	-0.42789702	0.04111236	-10.407989	2.28E-25	1.05E-23	EPRS1
483.353872	-0.19882502	0.08366567	-2.3764231	0.0174814	0.048231261	BPNT1
2456.088573	-0.58382241	0.04487288	-13.010585	1.07E-38	1.01E-36	IARS2
1590.736715	-0.34374492	0.05745587	-5.9827641	2.19E-09	2.21E-08	RAB3GAP2
306.176622	-0.27444943	0.09984647	-2.7487143	0.005983	0.019024229	MORF4L1P1
149.4974446	-0.64437702	0.15214524	-4.2352757	2.28E-05	0.000122709	C1orf115
165.4623125	1.270755221	0.14995288	8.4743638	2.36E-17	5.49E-16	DUSP10
198.1035642	-0.47139657	0.12884869	-3.6585283	0.0002537	0.001118763	TAF1A
2284.520896	-0.21631966	0.0526688	-4.1071691	4.01E-05	0.000206137	MIA3
314.3615907	-0.28160984	0.10049577	-2.8022059	0.0050754	0.016420843	AIDA
2163.376241	-0.28008308	0.05052099	-5.5438956	2.96E-08	2.56E-07	BROX
4591.282862	-0.12287796	0.04264301	-2.8815499	0.0039572	0.013178187	CAPN2
1143.227652	-0.22576351	0.06413148	-3.5203226	0.000431	0.001811814	FBXO28
2395.175338	0.18638069	0.0598334	3.1149944	0.0018395	0.006676817	DEGS1
670.2839948	-0.32678399	0.07368882	-4.434648	9.22E-06	5.37E-05	NVL
6035.950532	-0.179994	0.04019942	-4.4775277	7.55E-06	4.45E-05	WDR26
2303.467541	-0.435472	0.05205773	-8.3651749	6.00E-17	1.34E-15	LBR
48.93016196	1.306058336	0.31790134	4.1083763	3.98E-05	0.000205244	ENSG00000227496
2928.851189	-0.25083539	0.05023697	-4.993044	5.94E-07	4.21E-06	SRP9
776.2910268	0.391585945	0.07617625	5.1405257	2.74E-07	2.05E-06	EPHX1

317.1996034	0.350323372	0.10408694	3.3656805	0.0007636	0.003030849	TMEM63A
502.6620493	-0.2807438	0.08123509	-3.4559423	0.0005484	0.002253888	LIN9
5750.530524	-0.39607886	0.03869001	-10.237239	1.35E-24	5.77E-23	PARP1
68.19479577	-0.66628185	0.21371954	-3.1175523	0.0018236	0.006627446	ITPKB
360.7220638	-0.48681683	0.12021109	-4.0496832	5.13E-05	0.000258591	ZNF678
497.6989197	-0.24333552	0.08668157	-2.8072347	0.0049969	0.016187739	SNAP47
26.14979535	1.124862131	0.38531638	2.9193208	0.0035079	0.011824627	WNT9A
577.2417232	0.285845617	0.08119241	3.5205952	0.0004306	0.001810818	GUK1
169.7103332	0.361518568	0.15151891	2.3859634	0.0170344	0.047140196	IBA57
432.0109699	0.233620039	0.09115882	2.5627804	0.0103838	0.030835686	TRIM11
39.01612642	0.864085624	0.30472617	2.8356134	0.0045738	0.014956637	H2AW
6.762057443	1.857851265	0.72596041	2.5591633	0.0104924	0.031131817	RHOU
47.51709386	1.358294437	0.28822638	4.7125958	2.45E-06	1.57E-05	ACTA1
1492.717305	-0.30847954	0.05283961	-5.8380356	5.28E-09	5.03E-08	NUP133
556.1309461	-0.22969878	0.08271168	-2.7771022	0.0054846	0.017590754	ABCB10
829.8258946	-0.21538233	0.06943489	-3.1019322	0.0019226	0.006942349	TAF5L
2082.286669	0.338084156	0.05607935	6.028675	1.65E-09	1.69E-08	C1orf198
282.4058639	0.330900643	0.12183218	2.7160364	0.0066069	0.020765116	TTC13
34.99838009	2.278579987	0.41946322	5.432133	5.57E-08	4.62E-07	TRIM67
755.5799932	-0.47095723	0.06743615	-6.9837497	2.87E-12	3.96E-11	GNPAT
291.7778801	-0.33398309	0.10796943	-3.0933117	0.0019794	0.007123571	EGLN1
884.5901305	-0.19109561	0.06402433	-2.9847342	0.0028382	0.009812552	TSNAX
915.220531	1.013230871	0.07945883	12.751646	3.05E-37	2.78E-35	SIPA1L2
179.8912835	-0.63951952	0.12951463	-4.9378167	7.90E-07	5.48E-06	MAP3K21
162.9733752	-0.41314578	0.13924719	-2.9669955	0.0030073	0.010333165	COA6
3680.618265	-0.25124636	0.04431991	-5.668928	1.44E-08	1.30E-07	TOMM20
81.53967725	-0.50769773	0.18839774	-2.6948186	0.0070427	0.021952411	TBCE
33.2048163	-0.76321971	0.30257611	-2.5224057	0.0116555	0.034128552	GNG4
446.3533705	-0.52195932	0.09613957	-5.429183	5.66E-08	4.69E-07	LYST
226.8936346	-0.31849975	0.12119724	-2.6279456	0.0085902	0.026079611	GPR137B
1582.701053	-0.26473741	0.05393873	-4.908113	9.20E-07	6.31E-06	LGALS8
1845.103709	-0.53413406	0.06022991	-8.868253	7.43E-19	1.99E-17	HEATR1
14.58934671	1.4310927	0.48842561	2.9300116	0.0033895	0.011465336	ACTN2
1719.704067	-0.48579049	0.05905607	-8.2259203	1.94E-16	4.11E-15	MTR
150.3682433	-0.60061651	0.14269336	-4.2091414	2.56E-05	0.000136575	CHRM3
1417.755175	-0.64458019	0.06025841	-10.696933	1.05E-26	5.35E-25	FMN2
9.105480563	1.967283441	0.75243822	2.6145448	0.0089346	0.026993305	GREM2
7.463800025	1.6843958	0.70956784	2.3738334	0.0176045	0.048501608	ENSG00000286496
1603.445519	-0.25737362	0.05600317	-4.5956976	4.31E-06	2.66E-05	FH
108.1475103	-0.79963003	0.17501596	-4.5688977	4.90E-06	2.98E-05	OPN3
3945.112195	-0.62496743	0.04694874	-13.311697	1.98E-40	2.01E-38	CHML
1676.877871	-0.31774961	0.05173231	-6.1421891	8.14E-10	8.66E-09	EXO1
2377.579958	-0.28686379	0.0494957	-5.7957312	6.80E-09	6.41E-08	CEP170
2478.258517	-0.6664749	0.05259694	-12.671364	8.52E-37	7.56E-35	AKT3
1078.025748	-0.26104518	0.06111736	-4.2712113	1.94E-05	0.000106149	ZBTB18
12.51401066	1.379713534	0.50372654	2.739013	0.0061624	0.019523416	CATSPERE
444.7014161	-0.74428989	0.09031735	-8.2408298	1.71E-16	3.64E-15	COX20
14941.60583	-0.39685271	0.03192586	-12.430446	1.79E-35	1.49E-33	HNRNPU

333.4069263	-0.45735127	0.10925748	-4.1859951	2.84E-05	0.0001503	SMYD3
2181.883821	-0.17046747	0.04970648	-3.429482	0.0006047	0.002463996	AHCTF1
723.345484	0.269140244	0.07641426	3.5221207	0.0004281	0.001801747	ZNF731P
682.8304559	-0.51034234	0.07058805	-7.2298685	4.83E-13	7.29E-12	ZNF496
63.42569853	0.828964322	0.22224295	3.7299916	0.0001915	0.000868077	NLRP3
2246.700364	0.410237877	0.05298512	7.7425108	9.75E-15	1.76E-13	TRIM58
1751.22756	-0.22993207	0.05154372	-4.460913	8.16E-06	4.79E-05	ACP1
9733.509626	0.34304967	0.03818758	8.983279	2.63E-19	7.32E-18	PXDN
347.8637054	-0.32433159	0.10034361	-3.2322095	0.0012284	0.004640744	EIPR1
398.5704611	-0.3169705	0.0912082	-3.4752412	0.0005104	0.002113685	TRAPPC12
2665.24187	-0.13148722	0.05544807	-2.3713578	0.0177229	0.048765921	ADI1
4786.717004	-0.44043858	0.042556	-10.349624	4.20E-25	1.88E-23	RPS7
15.72375146	3.606496591	0.65581725	5.4992402	3.81E-08	3.24E-07	RSAD2
1120.625936	-0.27523402	0.06188992	-4.4471544	8.70E-06	5.08E-05	ID2
3052.376077	-0.16466083	0.04691511	-3.5097614	0.0004485	0.001878962	KIDINS220
3596.587864	-0.33483271	0.04879725	-6.8617128	6.80E-12	9.00E-11	MBOAT2
1702.981262	-0.24157761	0.05502369	-4.3904295	1.13E-05	6.48E-05	ASAP2
1133.091024	-0.17473864	0.06739022	-2.5929378	0.009516	0.028566063	ITGB1BP1
1418.892483	-0.34213913	0.05715471	-5.9861933	2.15E-09	2.16E-08	CPSF3
1536.276127	-0.16352206	0.05499978	-2.9731405	0.0029477	0.010152588	ADAM17
8598.699675	-0.29676182	0.04063389	-7.3033074	2.81E-13	4.35E-12	YWHAQ
812.0981582	-0.250942	0.06888927	-3.6426864	0.0002698	0.001182866	TAF1B
7803.163573	-0.26593282	0.03781057	-7.0332923	2.02E-12	2.82E-11	RRM2
692.0868232	0.265981304	0.0756226	3.5172199	0.0004361	0.001831798	HPCAL1
1328.774806	-0.15384209	0.05869888	-2.6208691	0.0087706	0.026545258	NOL10
6485.516279	-0.23472985	0.039008	-6.0174803	1.77E-09	1.81E-08	PDIA6
3742.073906	-0.2571097	0.05292791	-4.8577341	1.19E-06	8.01E-06	ROCK2
25.16013032	1.625747164	0.42235327	3.8492591	0.0001185	0.000556836	GREB1
779.4718958	-0.48738919	0.06663864	-7.313913	2.59E-13	4.04E-12	LPIN1
922.2133371	-0.67072796	0.06471057	-10.365045	3.58E-25	1.62E-23	NBAS
3487.538223	-0.21238342	0.04441841	-4.7814283	1.74E-06	1.15E-05	DDX1
192.5958755	0.601228319	0.14178647	4.2403787	2.23E-05	0.000120175	CYRIA
1932.76775	-0.12913824	0.04909188	-2.6305421	0.0085249	0.025903844	SMC6
38.67664726	0.939064138	0.30390326	3.0900101	0.0020015	0.007192814	OSR1
421.1556693	-0.41635653	0.09034724	-4.6084035	4.06E-06	2.51E-05	WDR35
413.3910953	0.765059723	0.09593767	7.9745498	1.53E-15	3.00E-14	MATN3
4978.51902	0.175930765	0.04584289	3.8376891	0.0001242	0.000581685	LAPTM4A
3139.379904	-0.42548993	0.04140768	-10.275629	9.08E-25	3.95E-23	PUM2
2764.840354	0.118400579	0.04548875	2.6028541	0.0092451	0.027829805	RHOB
484.2591593	-0.44650619	0.0865964	-5.1561748	2.52E-07	1.90E-06	LDAH
286.0198977	-0.28163076	0.10949067	-2.5721896	0.0101058	0.030123251	ATAD2B
2095.549955	-0.23877138	0.05762947	-4.1432165	3.42E-05	0.00017863	SF3B6
76.52051741	-0.74833051	0.20015025	-3.7388437	0.0001849	0.000841148	FAM228B
999.6612123	1.390063893	0.07190706	19.331396	2.92E-83	1.18E-80	TP53I3
1324.489367	-0.32531218	0.05722159	-5.6851298	1.31E-08	1.19E-07	ITSN2
753.4677044	-0.37358232	0.08015925	-4.6605015	3.15E-06	1.99E-05	NCOA1
1577.661473	-0.18748879	0.05193714	-3.6099178	0.0003063	0.001324659	CENPO
497.9566353	-0.74904466	0.08549094	-8.7616844	1.92E-18	4.90E-17	ADCY3

99.75812091	-0.67134785	0.1762909	-3.808182	0.00014	0.00064971	DNAJC27
75.59782394	0.943322886	0.26873139	3.5102818	0.0004476	0.00187574	EFR3B
528.7716965	-0.41825683	0.08234575	-5.0792766	3.79E-07	2.77E-06	DNMT3A
1779.875222	-0.50694892	0.05175181	-9.7957712	1.17E-22	4.33E-21	ASXL2
3411.643682	-0.19657561	0.04333941	-4.5357246	5.74E-06	3.44E-05	HADHA
1705.961214	-0.20679632	0.05519826	-3.7464284	0.0001794	0.00081913	HADHB
55.00013513	1.241244249	0.25180506	4.9293857	8.25E-07	5.70E-06	KCNK3
764.6424433	-0.31503804	0.07405991	-4.2538271	2.10E-05	0.000113811	CENPA
774.3716971	-0.40413864	0.07108937	-5.684938	1.31E-08	1.19E-07	AGBL5
21.94694896	3.362487248	0.80485266	4.1777674	2.94E-05	0.000155414	CGREF1
395.8018852	-0.28520603	0.09337188	-3.0545173	0.0022542	0.008001851	ATRAID
1939.738055	-0.28755962	0.04775938	-6.0210086	1.73E-09	1.77E-08	CAD
569.0300695	-0.29556804	0.07838839	-3.7705589	0.0001629	0.000747762	EIF2B4
4204.199713	-0.10761571	0.04309533	-2.4971548	0.0125194	0.036285478	PPM1G
1882.032449	-0.282857	0.05070516	-5.5784659	2.43E-08	2.13E-07	NRBP1
516.5106675	-0.55674631	0.08360466	-6.6592735	2.75E-11	3.43E-10	IFT172
708.9707262	-0.30557912	0.07082848	-4.3143538	1.60E-05	8.87E-05	GPN1
964.1914296	-0.2088086	0.06207362	-3.3638863	0.0007685	0.003046729	SLC4A1AP
212.2114352	-0.46141517	0.12102663	-3.8125094	0.0001376	0.000639973	BABAM2
14.43141972	1.592314141	0.5525016	2.8820082	0.0039515	0.013164079	PLB1
4424.497985	-0.25447424	0.04945053	-5.146037	2.66E-07	2.00E-06	PPP1CB
268.4563294	-0.36379878	0.11226063	-3.2406622	0.0011925	0.004517097	TRMT61B
1726.093373	1.531102561	0.06505757	23.534579	1.81E-122	1.74E-119	LBH
773.6524782	-0.41405101	0.07477721	-5.5371281	3.07E-08	2.65E-07	LCLAT1
483.2110178	0.430891156	0.08548818	5.0403594	4.65E-07	3.35E-06	EHD3
69.40357599	1.471516479	0.22698991	6.4827396	9.01E-11	1.07E-09	XDH
725.7140599	-0.36356573	0.08187251	-4.4406327	8.97E-06	5.23E-05	SPAST
1357.543458	-0.16223331	0.06448797	-2.5157144	0.0118791	0.03469571	SLC30A6
1300.06159	-0.30991506	0.05648664	-5.4865201	4.10E-08	3.47E-07	YIPF4
2615.426628	-0.56087621	0.13174744	-4.2572075	2.07E-05	0.000112419	BIRC6
732.8890302	-0.3288149	0.06887968	-4.7737578	1.81E-06	1.19E-05	TTC27
220.3626825	0.59140594	0.13408243	4.410764	1.03E-05	5.94E-05	LTBP1
99.16312977	0.577585424	0.19402552	2.9768529	0.0029122	0.010040404	RASGRP3
14021.20061	-0.28568153	0.03576379	-7.9880115	1.37E-15	2.71E-14	CRIM1
1808.893418	-0.44933866	0.05342259	-8.4110238	4.06E-17	9.19E-16	STRN
713.8415847	-0.24774733	0.07533801	-3.2884771	0.0010073	0.003889235	HEATR5B
1189.914577	-0.21982832	0.05914309	-3.7168892	0.0002017	0.000911014	GPATCH11
3206.219436	0.288587877	0.06081776	4.7451254	2.08E-06	1.35E-05	EIF2AK2
2052.723648	-0.37026392	0.04646883	-7.968006	1.61E-15	3.15E-14	PRKD3
51.88304681	1.267851706	0.28373161	4.4684895	7.88E-06	4.63E-05	QPCT
1152.703594	1.096323563	0.08014496	13.679258	1.35E-42	1.52E-40	CYP1B1
1354.011562	-0.28135916	0.05826341	-4.8290885	1.37E-06	9.18E-06	ATL2
913.9806828	-0.34127658	0.06561021	-5.201577	1.98E-07	1.52E-06	DHX57
185.3195093	-0.3876712	0.13553765	-2.8602474	0.0042331	0.013963148	MORN2
1861.026427	-0.5801053	0.04932563	-11.760727	6.22E-32	4.40E-30	SOS1
108.741032	0.432966929	0.17787645	2.434088	0.0149294	0.042180607	MAP4K3-DT
621.3280488	-0.34590854	0.07694496	-4.4955323	6.94E-06	4.10E-05	SLC8A1
3215.741104	-0.293188	0.04010191	-7.311073	2.65E-13	4.12E-12	EML4

1463.06465	-0.22761888	0.05618114	-4.0515179	5.09E-05	0.00025672	COX7A2L
734.4280399	-0.45128256	0.07324198	-6.1615283	7.20E-10	7.73E-09	MTA3
822.4166626	-0.41430008	0.07281218	-5.6899835	1.27E-08	1.16E-07	THADA
193.7167058	-0.32507145	0.12888991	-2.522086	0.0116661	0.034151056	DYNC2LI1
4547.703026	-0.23733933	0.0419217	-5.6614915	1.50E-08	1.35E-07	LRPPRC
2562.19862	-0.46923879	0.04962875	-9.4549788	3.23E-21	1.06E-19	PREPL
668.2333356	-0.46380572	0.0744454	-6.2301465	4.66E-10	5.12E-09	SRBD1
382.9860615	0.25598789	0.09373486	2.7309784	0.0063147	0.019958551	PRKCE
5865.32271	1.099280182	0.04420254	24.869165	1.60E-136	2.33E-133	EPAS1
845.6732196	-0.20056056	0.06930786	-2.8937636	0.0038065	0.012720184	RHOQ
775.5857334	-0.37220389	0.07353833	-5.061359	4.16E-07	3.03E-06	SOCS5
3164.994591	-0.326208	0.05124592	-6.3655415	1.95E-10	2.24E-09	MSH2
4066.447853	-0.55945991	0.03918455	-14.277564	3.02E-46	3.75E-44	MSH6
1516.285575	-0.30038068	0.05437116	-5.5246321	3.30E-08	2.84E-07	FBXO11
464.2333912	-0.46290128	0.08500954	-5.4452863	5.17E-08	4.32E-07	FOXN2
60.6069032	-1.14353971	0.22340414	-5.1187043	3.08E-07	2.28E-06	STON1
3449.811355	-0.24962374	0.05254958	-4.7502521	2.03E-06	1.32E-05	PSME4
4542.896681	-0.31400608	0.04755266	-6.6033342	4.02E-11	4.94E-10	RPS27A
1016.957128	-0.42154088	0.05939477	-7.0972733	1.27E-12	1.83E-11	MTIF2
521.8642653	-0.42800445	0.08149183	-5.2521148	1.50E-07	1.17E-06	CFAP36
2023.248092	-0.42233822	0.04779511	-8.8364307	9.88E-19	2.62E-17	PPP4R3B
593.00825	0.642849829	0.08531922	7.5346424	4.90E-14	8.25E-13	EFEMP1
355.5275875	-0.30777623	0.09874854	-3.1167676	0.0018285	0.006643723	VRK2
778.2812875	-0.23478259	0.08085866	-2.903617	0.0036888	0.012371857	FANCL
188.0105588	-0.43884896	0.14620042	-3.0016943	0.0026848	0.009335924	REL
159.6769381	-0.36811002	0.13703592	-2.6862302	0.0072263	0.022432317	PUS10
334.957831	-0.36556414	0.10367944	-3.5259079	0.000422	0.001780055	SANBR
3694.095208	-0.55948882	0.06017163	-9.2982153	1.43E-20	4.43E-19	USP34
11321.63851	-0.49940551	0.03286987	-15.193414	3.91E-52	6.13E-50	XPO1
293.4366797	-0.42000394	0.11211317	-3.7462499	0.0001795	0.000819498	FAM161A
6779.73689	-0.35767113	0.04205735	-8.5043666	1.83E-17	4.27E-16	CCT4
727.9318015	0.356680402	0.07088718	5.0316628	4.86E-07	3.50E-06	B3GNT2
33.95107573	-0.7299326	0.29965681	-2.4358953	0.014855	0.041997753	TMEM17
1884.565104	-0.51785599	0.05168093	-10.020254	1.24E-23	4.91E-22	EHBP1
2811.717676	-0.10782056	0.04538352	-2.3757644	0.0175126	0.048279147	MDH1
656.9917266	-0.65996592	0.07502145	-8.7970293	1.40E-18	3.64E-17	VPS54
437.0156768	0.352334246	0.09005114	3.9126019	9.13E-05	0.00043972	PELI1
246.433186	-0.29044248	0.11636587	-2.4959422	0.0125623	0.036387433	LGALSL
2529.727007	-0.40961764	0.04920343	-8.3249821	8.43E-17	1.85E-15	SERTAD2
864.2715219	0.424114829	0.07690282	5.514945	3.49E-08	2.98E-07	SLC1A4
340.8193538	-0.32675669	0.0974434	-3.3532974	0.0007985	0.003154648	CEP68
709.612277	-0.2641414	0.07045583	-3.7490356	0.0001775	0.000810875	SPRED2
23.32921307	2.445809093	0.48344715	5.0591034	4.21E-07	3.06E-06	LINC01828
755.6476747	-0.66198894	0.07723092	-8.5715537	1.02E-17	2.44E-16	ETAA1
279.1038063	-0.27335146	0.10778249	-2.5361399	0.0112082	0.032980576	C1D
538.5563524	-0.28224997	0.08060798	-3.5015138	0.0004626	0.001934363	CNRIP1
3087.441419	-0.1753609	0.04146917	-4.2287053	2.35E-05	0.000125997	GFPT1
554.7205404	0.54547788	0.08506454	6.4125179	1.43E-10	1.67E-09	ANXA4

641.4237845	-0.36713469	0.0805235	-4.5593484	5.13E-06	3.11E-05	GMCL1
310.8776495	0.264052861	0.11174881	2.3629143	0.0181319	0.04978104	MXD1
2949.090812	-0.45800637	0.04388826	-10.435739	1.70E-25	7.90E-24	PCBP1
1313.588503	-0.30667096	0.05640434	-5.4370103	5.42E-08	4.50E-07	TIA1
271.4811237	1.259955065	0.1208036	10.42978	1.81E-25	8.39E-24	TGFA
1724.232156	-0.13298719	0.05315159	-2.5020359	0.0123481	0.035878718	TEX261
989.075145	0.446634604	0.06710289	6.6559664	2.81E-11	3.50E-10	NAGK
175.8387863	-0.54728149	0.14323016	-3.8209934	0.0001329	0.000619345	PAIP2B
2897.597538	-0.23494005	0.04575954	-5.1342304	2.83E-07	2.12E-06	ZNF638
907.6187501	0.266359628	0.07265355	3.666161	0.0002462	0.00108978	DYSF
19.12634149	1.099724352	0.42897008	2.5636388	0.0103581	0.030764786	CYP26B1
233.5894908	0.335526398	0.12049884	2.7844782	0.0053614	0.01724332	SFXN5
839.3057286	0.178940305	0.06924265	2.5842498	0.0097591	0.02922605	RAB11FIP5
367.820787	-0.28218767	0.09475243	-2.9781577	0.0028999	0.010001725	SMYD5
6861.319906	-0.2636956	0.04482795	-5.8823928	4.04E-09	3.91E-08	CCT7
1011.539301	-0.76264199	0.0667829	-11.41972	3.33E-30	2.11E-28	ALMS1
588.9718386	-0.26881679	0.07611642	-3.531653	0.000413	0.001746489	TPRKB
1282.461245	-0.18116883	0.05538254	-3.2712263	0.0010708	0.004098964	STAMBP
527.6708692	2.0907715	0.10353581	20.193703	1.11E-90	6.04E-88	ACTG2
593.4851631	-0.23936277	0.08298164	-2.8845268	0.00392	0.013064242	TET3
83.53378115	-0.46536086	0.19403866	-2.3982894	0.0164718	0.045890005	BOLA3-DT
6011.480557	-0.30190473	0.04347222	-6.9447736	3.79E-12	5.18E-11	MOB1A
4962.571459	-0.11291054	0.0445245	-2.5359194	0.0112153	0.03299497	MTHFD2
3140.004571	-0.11822415	0.04392142	-2.6917197	0.0071085	0.022125691	DCTN1
413.2191539	-0.34540932	0.09088872	-3.8003541	0.0001445	0.000668619	WDR54
610.0073261	-0.22243295	0.0782912	-2.8410979	0.0044959	0.014744353	MOGS
60.5997942	0.74686438	0.25341131	2.9472417	0.0032062	0.010921891	LBX2-AS1
147.5489195	-0.68375115	0.14325272	-4.7730412	1.81E-06	1.19E-05	PCGF1
16.35043638	1.157858926	0.45101754	2.5672148	0.0102519	0.030490963	DQX1
403.1236183	-0.24524684	0.09362003	-2.619598	0.0088033	0.026631169	SEMA4F
3193.83493	-0.49714358	0.0424615	-11.708103	1.16E-31	7.90E-30	HK2
480.9326281	0.263524185	0.08314942	3.1692847	0.0015281	0.005644533	POLE4
1347.901561	-0.46198238	0.05651236	-8.1748912	2.96E-16	6.20E-15	MRPL19
1054.379124	-0.16826178	0.0655499	-2.5669265	0.0102604	0.030511114	SUCLG1
768.1191689	0.233897015	0.07514751	3.1125052	0.0018551	0.006724957	TRABD2A
1648.370875	-0.17366844	0.05412938	-3.2083953	0.0013348	0.004995009	KCMF1
329.1306122	0.354893387	0.1009614	3.5151392	0.0004395	0.001843984	TCF7L1
10.13402434	1.900490846	0.66639316	2.8519063	0.0043458	0.014288283	ENSG00000246575
790.7243675	0.528052837	0.0716749	7.367333	1.74E-13	2.76E-12	RETSAT
5654.814616	0.180156014	0.04516214	3.9890936	6.63E-05	0.000327861	MAT2A
1430.549024	-0.25892933	0.05268517	-4.9146526	8.89E-07	6.11E-06	USP39
473.9171651	-0.49648155	0.08792588	-5.6465918	1.64E-08	1.46E-07	C2orf68
114.1581962	1.494628593	0.1953195	7.6522242	1.98E-14	3.46E-13	ATOH8
1833.063815	-0.17113189	0.0629852	-2.7170176	0.0065873	0.020714885	POLR1A
1726.811953	-0.18988617	0.0514443	-3.6911024	0.0002233	0.000997409	PTCD3
2958.863467	-0.20360207	0.04567424	-4.4577006	8.28E-06	4.85E-05	IMMT
117.2434075	0.847785191	0.19442736	4.3604212	1.30E-05	7.33E-05	REEP1
2635.265748	-0.21725508	0.04475073	-4.8547832	1.21E-06	8.13E-06	KDM3A

1232.94949	-0.15225702	0.05974015	-2.5486548	0.0108139	0.031992887	CHMP3
546.1549901	0.257030392	0.08195499	3.1362385	0.0017113	0.006265159	RNF103
1279.602995	-0.24297791	0.06583724	-3.6905849	0.0002237	0.000999184	RMND5A
176.0272989	0.548375204	0.13823771	3.9669001	7.28E-05	0.000357086	KRCC1
217.5246378	-0.55308279	0.11941733	-4.6315118	3.63E-06	2.27E-05	RPIA
222.6933233	0.716158658	0.13151285	5.4455412	5.16E-08	4.31E-07	MRPS5
184.2939904	-0.40818659	0.1374244	-2.9702628	0.0029755	0.010238046	ZNF514
375.0098419	-0.33736316	0.12410666	-2.7183324	0.0065612	0.020655152	LINC00342
1090.050695	0.39342314	0.06630778	5.9332874	2.97E-09	2.93E-08	TMEM127
7201.755299	-0.42993036	0.0372096	-11.554287	7.02E-31	4.63E-29	SNRNP200
178.6324796	-0.81290562	0.12971206	-6.2670009	3.68E-10	4.10E-09	ITPRIPL1
2105.034715	-0.21711311	0.05041975	-4.3061121	1.66E-05	9.18E-05	NCAPH
144.9559231	0.638421372	0.15898216	4.0156793	5.93E-05	0.000295516	ARID5A
452.601552	0.270344451	0.09365407	2.8866279	0.0038939	0.012992268	CNNM4
242.0805661	-0.29707711	0.11169527	-2.6597107	0.0078208	0.024016135	SEMA4C
348.9282898	-0.38116957	0.09733468	-3.9160716	9.00E-05	0.000433923	ACTR1B
620.240154	-0.21087005	0.07637934	-2.7608256	0.0057655	0.018401436	INPP4A
137.6904194	0.540768666	0.16626042	3.25254	0.0011438	0.004351437	TSGA10
827.9316003	-0.30214033	0.0690979	-4.3726412	1.23E-05	6.99E-05	MRPL30
248.7163947	-0.41675468	0.12206593	-3.414177	0.0006397	0.002585464	AFF3
6340.307319	-0.20821993	0.04577503	-4.5487669	5.40E-06	3.25E-05	RPL31
173.678427	1.709873235	0.17796786	9.6077644	7.41E-22	2.54E-20	TBC1D8
962.7597352	-0.26940658	0.06554161	-4.1104661	3.95E-05	0.000203516	CNOT11
20.24421641	1.405933097	0.39916157	3.5222156	0.000428	0.001801538	LINC01127
368.6482908	0.335245498	0.09969852	3.3625925	0.0007721	0.003060063	MFSD9
205.4615657	-0.96139603	0.1262186	-7.6169122	2.60E-14	4.50E-13	POU3F3
1032.456455	-0.22856024	0.05930027	-3.8542864	0.0001161	0.000546256	TGFBRAP1
3302.596816	0.1398094	0.04517391	3.0949149	0.0019687	0.007086645	FHL2
1142.024392	0.282711541	0.06138101	4.6058473	4.11E-06	2.54E-05	UXS1
497.3017862	-0.98398353	0.09240236	-10.6489	1.76E-26	8.77E-25	ST6GAL2
1596.890398	-0.19365337	0.05062702	-3.8250994	0.0001307	0.000610094	GCC2
4110.761579	-0.23294646	0.0952127	-2.4465903	0.0144215	0.040985339	RANBP2
197.9160637	-0.31579073	0.12872643	-2.4531927	0.0141594	0.040373925	SH3RF3
2484.725224	-0.5297507	0.05293397	-10.007765	1.41E-23	5.51E-22	SEPTIN10
126.0257479	-0.50174286	0.1674141	-2.9970167	0.0027264	0.009463346	NPHP1
4264.462631	-0.34742807	0.04488701	-7.7400575	9.94E-15	1.79E-13	BUB1
564.4138588	-0.64994409	0.07556043	-8.601646	7.86E-18	1.91E-16	MIR4435-2HG
384.4600353	0.741214565	0.10346089	7.1642005	7.82E-13	1.15E-11	BCL2L11
18.21880738	-1.58132088	0.40590407	-3.8957995	9.79E-05	0.000468369	ENSG00000227992
894.276085	-0.42619581	0.06812235	-6.2563283	3.94E-10	4.39E-09	ANAPC1
303.5024806	0.387331165	0.11360857	3.4093482	0.0006512	0.002624351	MERTK
997.7918761	0.517063577	0.06628529	7.8005779	6.16E-15	1.13E-13	TMEM87B
2188.796312	-0.26641341	0.05175763	-5.1473261	2.64E-07	1.98E-06	TTL
2647.478907	-0.19981528	0.04572893	-4.369559	1.24E-05	7.08E-05	SLC20A1
3088.593131	-0.24800632	0.04387043	-5.6531544	1.58E-08	1.41E-07	CKAP2L
38.53556898	2.380356217	0.32235183	7.3843422	1.53E-13	2.45E-12	IL1B
328.3500116	-0.26377517	0.09950361	-2.6509106	0.0080275	0.02455988	CBWD2
19.93733381	1.095715947	0.42790406	2.560658	0.0104474	0.031008796	DDX11L2

3064.669246	-0.14847909	0.04336513	-3.4239281	0.0006172	0.002506684	SLC35F5
3738.943833	-0.17997309	0.04585613	-3.9247333	8.68E-05	0.000419767	DDX18
71.29207496	0.60933987	0.23045184	2.6441094	0.0081906	0.025014899	ENSG00000236255
1874.04035	0.209025894	0.04997657	4.1824778	2.88E-05	0.000152413	CCDC93
767.2920571	-0.44750006	0.08452292	-5.2944227	1.19E-07	9.45E-07	INSIG2
1161.208214	0.448264601	0.06120614	7.3238497	2.41E-13	3.77E-12	EPB41L5
1198.201703	-0.19745488	0.05682179	-3.4749851	0.0005109	0.00211479	TMEM185B
9.364857548	3.265828391	1.16663297	2.7993623	0.0051204	0.016544619	ENSG00000289091
1225.698882	-0.70756654	0.06985351	-10.129292	4.10E-24	1.68E-22	INHBB
343.1273316	-0.84299561	0.09583569	-8.7962593	1.41E-18	3.65E-17	GLI2
1935.399174	-0.14052223	0.05126083	-2.7413181	0.0061193	0.01940464	CLASP1
2843.178709	-0.31651364	0.04588248	-6.8983558	5.26E-12	7.10E-11	TSN
1741.025008	-0.15833458	0.06450997	-2.4544201	0.0141112	0.040268099	MAP3K2
2104.403899	-0.20920448	0.04916668	-4.2550052	2.09E-05	0.000113319	IWS1
416.5105641	0.738468328	0.10546928	7.0017385	2.53E-12	3.50E-11	LIMS2
108.3796798	-0.66466416	0.1722832	-3.8579742	0.0001143	0.000538957	SFT2D3
1267.49527	-0.26640505	0.05539682	-4.8090316	1.52E-06	1.01E-05	SAP130
4812.557381	-0.25554015	0.04789879	-5.3350023	9.55E-08	7.69E-07	UGGT1
1271.861679	-0.20158879	0.05422535	-3.7176117	0.0002011	0.000908649	SMPD4
415.9013959	-0.22237192	0.09301176	-2.3907935	0.016812	0.046673172	PTPN18
4229.418486	-0.18004524	0.04109249	-4.3814634	1.18E-05	6.72E-05	FAM168B
3744.914502	0.198680407	0.04922045	4.0365417	5.42E-05	0.000271928	PLEKHB2
112.075786	-0.39507856	0.16473001	-2.3983399	0.0164696	0.045890005	MZT2A
199.3567054	0.335117028	0.13461212	2.4895011	0.0127923	0.0369899	C2orf27A
3100.451287	-0.12595834	0.0443125	-2.8425012	0.0044761	0.014695012	RAB3GAP1
238.4209058	-0.8620348	0.12640872	-6.8194249	9.14E-12	1.19E-10	ZRANB3
1571.870426	-0.45063221	0.0530141	-8.5002325	1.89E-17	4.42E-16	R3HDM1
3489.256313	-0.35164819	0.04424648	-7.9474835	1.90E-15	3.67E-14	UBXN4
2550.763285	-0.95476602	0.04764347	-20.03981	2.48E-89	1.23E-86	MCM6
2994.294845	-0.45585486	0.04426049	-10.299364	7.09E-25	3.13E-23	DARS1
22.31990932	1.783432682	0.43303577	4.118442	3.81E-05	0.000197302	CXCR4
868.0877364	-0.33493498	0.06721875	-4.9827609	6.27E-07	4.43E-06	SPOPL
231.9574877	-0.42597264	0.11769076	-3.6194229	0.0002953	0.001282364	GTDC1
1409.413932	-0.24321021	0.05510587	-4.4135081	1.02E-05	5.87E-05	ZEB2
22.39358071	0.987799293	0.37189274	2.6561403	0.0079041	0.024224852	TEX41
273.8233568	0.369425324	0.11710559	3.1546344	0.001607	0.005916921	ACVR2A
973.4123833	-0.20370107	0.06278699	-3.2443195	0.0011773	0.004466302	ORC4
270.198213	-0.39572152	0.11122298	-3.5579115	0.0003738	0.001592125	MBD5
408.9973965	0.327365088	0.10986957	2.9795793	0.0028864	0.009963334	KIF5C
105.1074186	1.093741274	0.19571273	5.5885034	2.29E-08	2.01E-07	LYPD6B
343.4862089	0.261687365	0.10156062	2.5766618	0.0099759	0.029776092	LYPD6
2262.636785	-0.14395792	0.05282419	-2.7252274	0.0064257	0.020261706	MMADHC
4874.793986	0.925104346	0.04581722	20.191192	1.17E-90	6.17E-88	RND3
4631.164253	-0.48696335	0.05924407	-8.2196128	2.04E-16	4.31E-15	RIF1
601.0451419	-0.42850646	0.08375166	-5.1163935	3.11E-07	2.31E-06	ARL5A
24.07275224	1.39729962	0.36842603	3.7926192	0.0001491	0.00068885	CACNB4
1227.05477	-0.16633469	0.05976618	-2.7830904	0.0053844	0.017305793	STAM2
1340.85495	0.528466207	0.05812514	9.0918702	9.73E-20	2.80E-18	FMNL2

5701.85184	-0.24455704	0.0374956	-6.5222868	6.92E-11	8.34E-10	PRPF40A
67.59502804	-0.56861381	0.22109983	-2.5717515	0.0101185	0.030140729	GALNT13
17.69889404	1.319413217	0.45300498	2.91258	0.0035846	0.012059503	NR4A2
3378.534548	-0.69598231	0.04298245	-16.192244	5.72E-59	1.13E-56	PKP4
25.72138247	1.937628754	0.44232574	4.3805471	1.18E-05	6.75E-05	ENSG00000289488
19.23080503	1.667811671	0.46516435	3.5854245	0.0003365	0.001444496	RPL7AP22
1171.053651	0.399517096	0.06023292	6.6328691	3.29E-11	4.07E-10	TANC1
155.602681	-0.55730117	0.13948326	-3.9954699	6.46E-05	0.000319614	WDSUB1
258.9768359	-0.78235508	0.11442677	-6.8371681	8.08E-12	1.06E-10	CD302
1588.448789	0.294785115	0.05418433	5.440413	5.32E-08	4.42E-07	RBMS1
1987.916217	-0.18190719	0.05161541	-3.5242809	0.0004246	0.001790153	PSMD14
130.5561437	1.988170872	0.17813103	11.161283	6.31E-29	3.72E-27	DPP4
223.4568201	1.691441757	0.27777299	6.0892952	1.13E-09	1.19E-08	IFIH1
332.4869679	-0.38200961	0.10749768	-3.5536543	0.0003799	0.001614954	KCNH7
524.1733754	-0.46112823	0.09022203	-5.1110383	3.20E-07	2.36E-06	FIGN
829.1339289	0.483679818	0.06648613	7.2748979	3.47E-13	5.32E-12	COBLL1
7.844476752	2.103595369	0.69142313	3.042414	0.0023469	0.008281737	SCN3A
56.96473834	1.870342535	0.30410229	6.1503732	7.73E-10	8.24E-09	SCN2A
38.2609353	-1.75334364	0.29652421	-5.9129864	3.36E-09	3.28E-08	CSRNP3
1429.629405	-0.41235063	0.07167964	-5.7526886	8.78E-09	8.16E-08	GALNT3
885.9751379	-0.25397204	0.06265835	-4.0532835	5.05E-05	0.000254938	TTC21B
936.0159509	0.36173115	0.06970096	5.1897587	2.11E-07	1.60E-06	STK39
2238.392123	-0.25630452	0.05590197	-4.5848927	4.54E-06	2.79E-05	CERS6
667.8845566	-0.47570814	0.07301423	-6.5152794	7.26E-11	8.71E-10	SPC25
482.0266043	-0.30871457	0.09116936	-3.3861659	0.0007088	0.00283213	FASTKD1
3242.495221	-0.28766901	0.04407775	-6.5263996	6.74E-11	8.12E-10	PPIG
942.0024414	-0.46383791	0.06823501	-6.7976531	1.06E-11	1.38E-10	KLHL23
4618.155476	-0.14716261	0.0456813	-3.2215069	0.0012752	0.004798842	SSB
552.7501545	-0.44848375	0.07921505	-5.6615976	1.50E-08	1.35E-07	METTL5
1653.006871	-0.14411349	0.05887483	-2.4477948	0.0143733	0.040855266	UBR3
95.77151866	0.569114202	0.17874815	3.1838887	0.0014531	0.005393717	GAD1
1670.387151	-0.39896233	0.05576204	-7.1547303	8.38E-13	1.23E-11	TLK1
324.7997567	-0.25547297	0.10102449	-2.5288223	0.0114446	0.033601475	METTL8
3266.817106	0.123286912	0.04909013	2.5114402	0.012024	0.035053945	CYBRD1
2003.685026	-0.18891592	0.05672176	-3.3305721	0.0008667	0.003399846	DYNC1I2
3093.654916	-0.24573949	0.04768741	-5.1531311	2.56E-07	1.93E-06	HAT1
172.7793002	-0.66517944	0.142132	-4.6800117	2.87E-06	1.82E-05	METAP1D
146.0369027	0.938114546	0.16681661	5.6236278	1.87E-08	1.66E-07	DLX2
2048.558521	0.141896184	0.05808284	2.4429968	0.0145659	0.041314694	ITGA6
1623.577693	-0.34119672	0.05696771	-5.9893	2.11E-09	2.12E-08	PDK1
8170.589344	-0.33999898	0.04487432	-7.5766944	3.54E-14	6.06E-13	MAP3K20
1531.396622	-0.68871203	0.05393418	-12.769491	2.43E-37	2.22E-35	CDCA7
2889.999068	-0.36668305	0.04362919	-8.4045349	4.30E-17	9.69E-16	SP3
2651.104999	-0.3531344	0.04818513	-7.3287001	2.32E-13	3.64E-12	OLA1
424.9555654	-0.37397556	0.08883531	-4.2097628	2.56E-05	0.000136242	SCRN3
153.2425768	-0.62843122	0.15763594	-3.9865986	6.70E-05	0.000331138	WIPF1
1414.848069	-0.38539495	0.05631799	-6.8431946	7.74E-12	1.02E-10	ATF2
1379.881948	-0.13777074	0.0569974	-2.4171409	0.015643	0.043883058	ATP5MC3

2135.035284	-0.50707643	0.0524646	-9.6651159	4.24E-22	1.49E-20	LNPK
447.2918156	-0.42980758	0.08996511	-4.7774917	1.77E-06	1.17E-05	MTX2
5405.295402	-0.45386625	0.03937706	-11.526159	9.74E-31	6.37E-29	HNRNPA3
2479.590724	-0.14725497	0.04925493	-2.9896495	0.002793	0.009675303	AGPS
178.8971068	-0.80717574	0.13056728	-6.1820675	6.33E-10	6.83E-09	TTC30A
216.4884342	0.468050432	0.12241854	3.8233623	0.0001316	0.000613916	CHROMR
950.1344573	-0.29556204	0.06351978	-4.6530709	3.27E-06	2.05E-05	PRKRA
17.82704295	1.33297807	0.4735036	2.8151382	0.0048756	0.015848063	ENSG00000270277
862.9371504	-0.29956046	0.06389244	-4.6885119	2.75E-06	1.75E-05	CWC22
483.765838	-0.42882538	0.08591039	-4.9915426	5.99E-07	4.24E-06	UBE2E3
3044.481402	-0.18391168	0.04313995	-4.2631411	2.02E-05	0.000109714	ITPRID2
6845.625603	-0.25607485	0.03519975	-7.2749063	3.47E-13	5.32E-12	NCKAP1
6365.666119	0.454608998	0.04636504	9.804995	1.07E-22	3.99E-21	ITGAV
475.3917191	0.345167684	0.09368556	3.6843211	0.0002293	0.001021453	FAM171B
540.157848	-0.88693079	0.09133286	-9.710971	2.71E-22	9.69E-21	TFPI
383.7871427	0.255933128	0.09215289	2.7772663	0.0054818	0.01758512	GULP1
7947.773023	0.789095333	0.09957418	7.9246982	2.29E-15	4.38E-14	COL5A2
1441.393722	-0.21220982	0.05971366	-3.5537901	0.0003797	0.001614515	WDR75
233.3024392	-0.48575585	0.11378937	-4.2689036	1.96E-05	0.00010722	OSGEPL1
612.9708595	-0.54434302	0.07380714	-7.3752079	1.64E-13	2.62E-12	PMS1
20.40238694	1.244013075	0.42382054	2.9352354	0.0033329	0.011311465	C2orf88
521.0048524	-0.3612118	0.08481857	-4.2586406	2.06E-05	0.000111771	HIBCH
227.3670598	0.472133403	0.12775007	3.6957584	0.0002192	0.00098248	INPP1
421.6878514	-0.34207351	0.08922049	-3.834024	0.0001261	0.000589949	MFSD6
236.7256697	-0.40247158	0.12101042	-3.3259249	0.0008813	0.003450034	NEMP2
642.1414731	-0.43745882	0.0737303	-5.9332295	2.97E-09	2.93E-08	NAB1
29.60931702	2.482095822	0.49470702	5.0173047	5.24E-07	3.75E-06	ENSG00000228509
4463.675192	1.184253	0.10020302	11.818536	3.13E-32	2.24E-30	STAT1
10.7956196	2.188797995	0.64443582	3.3964561	0.0006826	0.002738454	STAT4
6662.080564	-0.09506292	0.03967244	-2.3961953	0.0165663	0.04611918	MYO1B
2060.428481	0.840343988	0.11636849	7.2214051	5.15E-13	7.72E-12	NABP1
17.50654631	2.72222553	0.52804614	5.1552797	2.53E-07	1.91E-06	CAVIN2
60.34232914	1.364039093	0.26651774	5.118005	3.09E-07	2.29E-06	TMEFF2
1139.602773	-0.5416617	0.0648877	-8.347679	6.96E-17	1.54E-15	SLC39A10
1824.766363	0.222110002	0.06049517	3.6715327	0.0002411	0.001069302	STK17B
144.5065215	1.085345161	0.15529804	6.9887883	2.77E-12	3.83E-11	HECW2
319.1016679	0.635495253	0.10683242	5.9485245	2.71E-09	2.69E-08	CCDC150
1153.308754	-0.34349304	0.06404575	-5.3632453	8.17E-08	6.63E-07	GTF3C3
202.4586923	-0.64337549	0.12890745	-4.9909875	6.01E-07	4.25E-06	ANKRD44
8300.726807	-0.26057783	0.03464946	-7.5203998	5.46E-14	9.15E-13	SF3B1
875.5837926	0.191033967	0.07189993	2.6569423	0.0078853	0.024175824	COQ10B
12774.46692	-0.37773634	0.04283316	-8.8187833	1.16E-18	3.04E-17	HSPD1
147.151732	0.667598061	0.15949136	4.1857945	2.84E-05	0.000150387	MARS2
794.6805584	-0.19453304	0.07038313	-2.7639157	0.0057112	0.018250325	C2orf69
1823.450126	0.296728628	0.05335301	5.5616095	2.67E-08	2.33E-07	SPATS2L
196.0017858	-0.36718519	0.1284986	-2.8575034	0.0042699	0.014065773	KCTD18
2954.755314	-0.37554073	0.04682847	-8.0194956	1.06E-15	2.13E-14	SGO2
2536.375417	-0.23730165	0.04759122	-4.9862482	6.16E-07	4.35E-06	BZW1

371.6197015	-0.49709462	0.09185305	-5.4118469	6.24E-08	5.15E-07	PPIL3
619.6645825	-0.24799303	0.07472606	-3.3186952	0.0009044	0.003528687	NIF3L1
928.0513464	-0.52593292	0.0738488	-7.1217528	1.07E-12	1.55E-11	ORC2
923.8553296	0.485172629	0.06520615	7.4405957	1.00E-13	1.63E-12	CFLAR
1958.563419	0.120673389	0.04966192	2.4298977	0.0151031	0.042596571	TRAK2
879.3846107	-0.30517592	0.07024388	-4.3445197	1.40E-05	7.83E-05	TMEM237
42.30501467	-0.69614942	0.28097374	-2.4776316	0.0132258	0.038047949	MPP4
163.0757992	-0.35421658	0.14454982	-2.450481	0.0142665	0.04062474	CDK15
2935.177349	-0.16172128	0.04881964	-3.3126276	0.0009242	0.003600476	SUMO1
2963.162062	-0.37264499	0.06256912	-5.9557336	2.59E-09	2.58E-08	BMPR2
724.5889196	-0.39014434	0.07040365	-5.5415361	3.00E-08	2.59E-07	WDR12
1012.109927	-0.6921094	0.08047074	-8.6007589	7.92E-18	1.92E-16	NBEAL1
2486.801022	-0.6079728	0.04722152	-12.874909	6.23E-38	5.83E-36	ABI2
93.36576267	-0.73493262	0.19965802	-3.6809571	0.0002324	0.001033702	PARD3B
88.9231977	0.669198966	0.18826884	3.5544861	0.0003787	0.00161143	NRP2
2402.985163	-0.38030131	0.04953389	-7.6775989	1.62E-14	2.86E-13	NDUFS1
2554.346844	-0.17611229	0.04560435	-3.8617431	0.0001126	0.00053114	EEF1B2
63.78350543	1.126155417	0.24693332	4.5605648	5.10E-06	3.09E-05	CMKLR2
1417.760761	-0.33012865	0.062978	-5.2419681	1.59E-07	1.23E-06	ZDBF2
83.0495313	1.130818328	0.2165749	5.221373	1.78E-07	1.37E-06	ADAM23
755.364069	-0.25426247	0.07033471	-3.6150353	0.0003003	0.001301678	FASTKD2
99.94072908	0.821532586	0.18248046	4.5020306	6.73E-06	3.99E-05	MYOSLID
1740.277817	-0.4059631	0.05740108	-7.0723949	1.52E-12	2.16E-11	CREB1
1115.580749	0.434667306	0.06178115	7.0355976	1.98E-12	2.78E-11	CCNYL1
348.5183376	-0.79426379	0.09935048	-7.994564	1.30E-15	2.57E-14	PLEKHM3
1100.393784	-0.41730406	0.06597861	-6.3248384	2.53E-10	2.88E-09	IDH1
1463.071672	-0.35492825	0.06584252	-5.3905628	7.02E-08	5.75E-07	PIKFYVE
21.36925728	2.842381419	0.68601206	4.1433403	3.42E-05	0.00017863	MAP2
2049.061621	-0.26064393	0.05092496	-5.1181959	3.08E-07	2.29E-06	RPE
156.5995073	-0.4707908	0.14514774	-3.2435283	0.0011806	0.004475981	KANSL1L
1631.193873	-0.56060098	0.05472304	-10.244332	1.25E-24	5.40E-23	LANCL1
152.3231516	-0.44370092	0.15475164	-2.8671808	0.0041415	0.01369984	IKZF2
231.1344252	-0.34718366	0.11575829	-2.9992121	0.0027068	0.009406682	SPAG16
21.87129854	1.436844317	0.44218211	3.2494402	0.0011563	0.004394841	ABCA12
2003.580691	-0.26225286	0.04880719	-5.3732422	7.73E-08	6.29E-07	ATIC
280958.7838	-0.4626661	0.04616694	-10.021589	1.23E-23	4.85E-22	FN1
45.8457668	1.103221656	0.32509936	3.3934907	0.0006901	0.002767637	TMEM169
10910.84344	-0.47445192	0.03594012	-13.201179	8.64E-40	8.58E-38	XRCC5
770.194302	0.277024573	0.07503887	3.6917478	0.0002227	0.000995136	MARCHF4
438.8282673	-0.41946191	0.09225993	-4.546523	5.45E-06	3.28E-05	SMARCAL1
7978.698019	-0.36120767	0.03981749	-9.0715833	1.17E-19	3.37E-18	RPL37A
248.0459409	0.764114594	0.12735486	5.9998857	1.97E-09	2.00E-08	IGFBP2
307.2157868	1.384540006	0.11427699	12.115649	8.73E-34	6.84E-32	IGFBP5
20.19580863	1.336648073	0.38932078	3.4332821	0.0005963	0.002433136	DIRC3-AS1
6767.835943	0.188788941	0.03991559	4.7297042	2.25E-06	1.45E-05	ARPC2
1505.321845	-0.26131158	0.05537505	-4.7189409	2.37E-06	1.53E-05	AAMP
576.7033387	-0.53259976	0.07624634	-6.9852502	2.84E-12	3.92E-11	CTDSP1
1473.39268	-0.4663546	0.05807089	-8.0307804	9.69E-16	1.96E-14	USP37

1382.30842	-0.19365111	0.05710056	-3.3914045	0.0006954	0.002785476	CNOT9
391.5309384	0.31199692	0.09139934	3.4135575	0.0006412	0.002590145	RNF25
1324.035622	-0.18843148	0.05798949	-3.2494074	0.0011565	0.004394841	RETREG2
559.171444	-0.32968937	0.08930366	-3.6917788	0.0002227	0.000995136	ANKZF1
445.7474047	0.768444357	0.09726933	7.9001714	2.79E-15	5.29E-14	TUBA4A
723.8494951	0.37670243	0.07495882	5.0254585	5.02E-07	3.61E-06	DNAJB2
52.43515944	0.649916051	0.25558413	2.5428654	0.0109948	0.032445073	PTPRN
16.47224908	2.095738421	0.51545975	4.0657654	4.79E-05	0.000243435	DES
497.7607599	-0.24471566	0.09103338	-2.6881971	0.0071839	0.022316539	SPEG
5.217331302	2.723285135	1.04693769	2.6011912	0.0092901	0.027955396	ASIC4
420.4238685	0.721341005	0.09644826	7.479046	7.49E-14	1.23E-12	CHPF
31.04327526	1.007693196	0.3516003	2.8660192	0.0041567	0.013742366	TMEM198
924.6139433	-0.21847312	0.07463475	-2.9272304	0.00342	0.011561626	OBSL1
6.914304223	1.868913319	0.69732468	2.6801193	0.0073596	0.022793112	INHA
223.5134992	1.694778085	0.14717414	11.515461	1.10E-30	7.16E-29	EPHA4
1857.797976	-0.46525613	0.05648612	-8.236645	1.77E-16	3.77E-15	FARSB
4444.544514	-0.24580939	0.04081197	-6.0229727	1.71E-09	1.75E-08	ACSL3
38.13282676	0.938364513	0.28430936	3.3005051	0.0009651	0.003746262	SCG2
184.4685512	0.48911853	0.13857641	3.5295944	0.0004162	0.001757996	AP1S3
3060.627501	1.317109132	0.05162148	25.514747	1.35E-143	2.35E-140	SERPINE2
2864.236479	-0.34400683	0.04729405	-7.2737867	3.50E-13	5.35E-12	CUL3
560.661862	-0.39499316	0.08163549	-4.8384981	1.31E-06	8.77E-06	DOCK10
1347.993826	-0.57520875	0.05376921	-10.697735	1.04E-26	5.32E-25	IRS1
402.6787024	-0.50759493	0.09144436	-5.5508611	2.84E-08	2.47E-07	RHBDD1
1210.720937	-0.37854474	0.05613672	-6.7432641	1.55E-11	1.97E-10	MFF
2318.632242	-0.31975329	0.04407618	-7.2545606	4.03E-13	6.13E-12	AGFG1
256.0939967	0.944293238	0.12654971	7.4618363	8.53E-14	1.39E-12	PID1
568.7970821	1.204208821	0.08535817	14.107716	3.40E-45	4.00E-43	DNER
7699.226985	-0.20372958	0.03790502	-5.374739	7.67E-08	6.24E-07	TRIP12
769.983537	1.240534718	0.13888791	8.9319132	4.19E-19	1.15E-17	SP110
542.8232862	0.331413976	0.08195828	4.0436913	5.26E-05	0.000264297	SP140L
1845.720079	0.807286304	0.06178088	13.066927	5.09E-39	4.92E-37	SP100
2194.96094	0.402282649	0.05088568	7.9056167	2.67E-15	5.07E-14	ITM2C
3951.749276	-0.2986373	0.04155101	-7.187246	6.61E-13	9.80E-12	PSMD1
313.4840394	-0.38946099	0.10565108	-3.6862945	0.0002275	0.001014609	ARMC9
22276.14094	-0.18334715	0.03743685	-4.8975047	9.71E-07	6.63E-06	NCL
10728.07856	-0.42329028	0.04488037	-9.4315234	4.04E-21	1.31E-19	PTMA
239.9707982	-0.3310071	0.11824318	-2.7993758	0.0051202	0.016544619	DIS3L2
6.601940341	3.630502443	1.33435879	2.7207843	0.0065127	0.020513725	ALPG
315.6048803	1.007663433	0.13143308	7.6667415	1.76E-14	3.11E-13	EFHD1
1995.41254	-0.25486459	0.04792626	-5.3178491	1.05E-07	8.38E-07	GIGYF2
60.16689526	0.913961437	0.26573443	3.4393791	0.0005831	0.002384021	INPP5D
645.1141063	-0.25162688	0.07840841	-3.2091821	0.0013311	0.004982432	DGKD
769.5285061	-0.23723104	0.07137773	-3.3236005	0.0008886	0.003476813	USP40
699.2079485	-0.45516456	0.07059154	-6.4478629	1.13E-10	1.34E-09	AGAP1
196.361865	0.935550292	0.1361586	6.8710335	6.37E-12	8.47E-11	GBX2
8.084344978	2.428417828	1.00756151	2.4101931	0.0159441	0.044598283	ACKR3
135.0154436	-0.61271334	0.14682209	-4.1731685	3.00E-05	0.000158346	MLPH

3183.751738	-0.16355238	0.04360187	-3.7510406	0.0001761	0.000804629	LRRFIP1
12.92309874	2.114338284	0.57109692	3.7022407	0.0002137	0.000960038	KLHL30
234.3619924	0.366525447	0.12816488	2.8597963	0.0042391	0.013980372	ERFE
82.79764161	1.154603156	0.22306768	5.1760217	2.27E-07	1.72E-06	HES6
442.4722128	-0.31254135	0.08663455	-3.6075831	0.0003091	0.001335304	TRAF3IP1
1068.123131	-0.17709912	0.06109618	-2.8986939	0.0037472	0.012538753	ASB1
54.68006789	-0.64509239	0.23627686	-2.7302394	0.0063288	0.019996081	HDAC4
1391.532176	-0.15294362	0.05342106	-2.8629836	0.0041967	0.013865594	NDUFA10
903.7716447	0.393896726	0.06823412	5.772724	7.80E-09	7.30E-08	GPC1
72.893374	0.665423154	0.21415271	3.1072366	0.0018885	0.006833162	CAPN10-DT
254.4431375	-0.55130084	0.12373454	-4.4555128	8.37E-06	4.90E-05	PASK
981.2660643	-0.15694535	0.06605794	-2.3758741	0.0175074	0.048279147	PPP1R7
18927.2309	-0.14135946	0.03269633	-4.3234039	1.54E-05	8.54E-05	HDLBP
480.1580708	-0.20298469	0.08495298	-2.3893771	0.016877	0.046808696	THAP4
964.4738576	0.233967733	0.06475226	3.6132751	0.0003024	0.001309898	ATG4B
753.5488184	-0.37145234	0.06876428	-5.4018212	6.60E-08	5.42E-07	DTYMK
538.229217	-0.3163607	0.08073611	-3.9184536	8.91E-05	0.000429777	ING5
276.1551695	0.951229558	0.12784927	7.4402423	1.01E-13	1.63E-12	ITPR1
808.9139212	-0.40736869	0.09287434	-4.386235	1.15E-05	6.59E-05	BHLHE40
2138.438967	0.120115701	0.05066281	2.3708852	0.0177455	0.048814095	ARL8B
1241.739106	-0.24857413	0.06030454	-4.1219805	3.76E-05	0.000194468	EDEM1
1361.443935	0.90667867	0.06997632	12.956935	2.15E-38	2.03E-36	LMCD1
4213.740693	-0.51723839	0.05381612	-9.6112175	7.17E-22	2.47E-20	OXTR
1479.996966	-0.3698016	0.05439024	-6.799043	1.05E-11	1.37E-10	RAD18
19.6428263	1.871709312	0.48363068	3.8701211	0.0001088	0.000515311	SRGAP3
678.1089548	0.72303228	0.09061292	7.9793508	1.47E-15	2.90E-14	THUMPD3-AS1
836.9176309	-0.17989862	0.06676364	-2.6945596	0.0070482	0.02196297	THUMPD3
2650.150487	-0.36595919	0.05315683	-6.8845185	5.80E-12	7.77E-11	SETD5
479.8393663	0.280352855	0.08603299	3.2586667	0.0011194	0.004269765	ARPC4
232.276679	0.298983236	0.1263585	2.3661505	0.0179741	0.049371386	TTLL3
882.4206037	0.239997496	0.06919984	3.4681798	0.000524	0.002164442	EMC3
124.9655368	0.783306259	0.16766226	4.6719296	2.98E-06	1.89E-05	CIDECP1
1110.39749	-0.49012506	0.06195733	-7.9106872	2.56E-15	4.88E-14	FANCD2
147.6525626	0.674244729	0.15285009	4.4111504	1.03E-05	5.93E-05	IRAK2
17.79808146	1.515887742	0.46679432	3.2474425	0.0011645	0.004423365	GHRLOS
594.7063263	-0.22556524	0.07866705	-2.8673408	0.0041394	0.01369552	HRH1
651.4270004	0.48564304	0.07665712	6.3352632	2.37E-10	2.70E-09	VGLL4
81.73094026	-0.90456747	0.19963955	-4.5310034	5.87E-06	3.51E-05	PPARG
428.8087773	0.392168067	0.09807401	3.9986953	6.37E-05	0.000315738	TMEM40
7326.692968	-0.30146728	0.03949554	-7.6329457	2.29E-14	3.99E-13	RPL32
1238.792698	-0.14573239	0.05826183	-2.5013356	0.0123726	0.035931743	TMEM43
810.9534915	0.164803927	0.06910442	2.3848536	0.0170859	0.047264475	XPC
1079.088412	-0.73871796	0.0664088	-11.123795	9.61E-29	5.63E-27	SLC6A6
2936.712303	-0.250576	0.04588618	-5.4608164	4.74E-08	3.98E-07	FGD5-AS1
1514.224184	-0.40797391	0.05389696	-7.5695162	3.75E-14	6.40E-13	NR2C2
708.0714224	-0.21600532	0.06975889	-3.096456	0.0019585	0.007054516	RBSN
154.4938147	1.146353135	0.2932448	3.9092019	9.26E-05	0.000445338	SH3BP5-AS1
730.0411836	-0.34344813	0.07190188	-4.7766221	1.78E-06	1.17E-05	SH3BP5

2252.877395	-0.49318417	0.04512225	-10.929954	8.29E-28	4.53E-26	ANKRD28
701.2510685	0.198949526	0.07446241	2.6718115	0.0075443	0.023288212	DPH3
204.5225426	-0.51620631	0.11938424	-4.3239068	1.53E-05	8.52E-05	OXNAD1
603.9790196	0.262241409	0.07651352	3.4273866	0.0006094	0.002478445	RFTN1
831.4149084	-0.80024445	0.06673693	-11.991029	3.96E-33	2.97E-31	TBC1D5
763.8668052	-0.33126858	0.06805061	-4.8679736	1.13E-06	7.63E-06	SGO1
100.7531911	-0.4447822	0.16567211	-2.6847137	0.0072592	0.022522284	HMGB1P5
356.8141172	0.237353645	0.09837185	2.4128207	0.0158296	0.044320851	NKIRAS1
5453.805959	-0.17751147	0.04499878	-3.9448058	7.99E-05	0.000388706	RPL15
1813.31648	-0.44225746	0.05815308	-7.6050569	2.85E-14	4.92E-13	NR1D2
6.202798537	3.218076832	1.0278835	3.1307797	0.0017434	0.0063667	RARB
2938.667485	-0.75154428	0.04548652	-16.522351	2.53E-61	5.44E-59	TOP2B
48.93391271	0.866807436	0.26631684	3.2547977	0.0011347	0.004321722	NEK10
485.9814722	-1.04947944	0.08445925	-12.425868	1.89E-35	1.57E-33	RBMS3
564.209871	0.468322033	0.07973879	5.8732023	4.27E-09	4.12E-08	OSBPL10
3079.375741	0.268922085	0.05130775	5.2413542	1.59E-07	1.24E-06	CMTM6
7.285545473	3.453740797	1.18143349	2.9233476	0.0034629	0.01169173	CCR4
748.7294658	0.352417229	0.0773413	4.55665	5.20E-06	3.14E-05	GLB1
2916.612609	-0.11597589	0.04632002	-2.5037961	0.0122869	0.035724608	CRTAP
461.2104356	0.342818235	0.08891761	3.8554596	0.0001155	0.000543937	SUSD5
232.9367416	0.682449872	0.12000756	5.6867242	1.29E-08	1.18E-07	FBXL2
2026.761801	0.124730824	0.04892584	2.5493852	0.0107913	0.031936822	UBP1
319.5662905	0.567587403	0.11015073	5.1528249	2.57E-07	1.93E-06	PDCD6IP-DT
3405.457376	-0.17386595	0.04061315	-4.281026	1.86E-05	0.000101798	PDCD6IP
17.83189436	1.60336295	0.43178016	3.713378	0.0002045	0.000922315	TRANK1
1557.18162	0.293761914	0.05396095	5.4439721	5.21E-08	4.34E-07	LRRFIP2
31.17797978	1.514899355	0.35159424	4.3086581	1.64E-05	9.08E-05	UBE2FP1
4445.201722	-0.23675823	0.04051248	-5.8440818	5.09E-09	4.86E-08	GOLGA4
380.601259	0.416761945	0.09496255	4.3886981	1.14E-05	6.53E-05	ACAA1
159.2043492	0.871141526	0.14967956	5.8200434	5.88E-09	5.58E-08	MYD88
2300.590742	-0.18378177	0.04898017	-3.7521669	0.0001753	0.000801369	OXSR1
122.3083762	-0.70136627	0.15505297	-4.523398	6.09E-06	3.63E-05	XYLB
106.4823412	-1.09833696	0.17414921	-6.3068731	2.85E-10	3.22E-09	ACVR2B
1415.305907	-0.36031627	0.05560229	-6.4802413	9.16E-11	1.09E-09	WDR48
429.0267751	0.403372183	0.09229835	4.3703075	1.24E-05	7.06E-05	SLC25A38
6011.10621	-0.35563356	0.0375997	-9.4584156	3.13E-21	1.02E-19	RPSA
34.95718337	1.08007423	0.30095425	3.5888319	0.0003322	0.001426955	EIF1B-AS1
2898.133709	-0.3450148	0.04614235	-7.4771835	7.59E-14	1.25E-12	RPL14
963.1042546	-0.24888183	0.06436642	-3.8666412	0.0001103	0.000521864	ZNF621
944.0384741	-0.29925823	0.06425979	-4.657006	3.21E-06	2.02E-05	SEC22C
90.74451221	0.520164656	0.20146355	2.5819294	0.009825	0.029392084	ZBTB47
14.34399781	2.467069239	0.78376071	3.1477327	0.0016454	0.006045609	CCDC13-AS2
504.6013316	0.271125931	0.08731686	3.1050811	0.0019023	0.006876002	HIGD1A
479.1816974	0.200736394	0.08381025	2.3951295	0.0166145	0.04622817	SNRK
4.115397799	2.397619205	1.00461802	2.3865979	0.0170051	0.047081414	SNRK-AS1
118.1323001	0.795157364	0.19571162	4.0629031	4.85E-05	0.000245865	ENSG00000261786
435.8928639	-0.22045564	0.09164431	-2.4055572	0.0161478	0.045095655	TCAIM
168.9607521	-0.51960722	0.14173539	-3.6660372	0.0002463	0.00109003	ZNF660

485.8548196	-0.31057284	0.08411033	-3.692446	0.0002221	0.000993684	ZNF197
710.5674839	-0.3846868	0.07223439	-5.3255358	1.01E-07	8.07E-07	KIAA1143
1386.796354	-0.70743293	0.05822458	-12.150073	5.73E-34	4.51E-32	KIF15
421.0924665	-0.60964994	0.08678451	-7.0248704	2.14E-12	2.99E-11	LZTFL1
642.8545904	-0.46131698	0.07534722	-6.1225479	9.21E-10	9.72E-09	FYCO1
108.3310827	1.555454313	0.20027214	7.7667035	8.06E-15	1.46E-13	ALS2CL
2763.065432	-0.1914165	0.05127305	-3.7332774	0.000189	0.00085772	SETD2
453.2969513	0.243076106	0.0871864	2.7880048	0.0053034	0.017072512	KLHL18
3874.552829	-0.33635967	0.0428532	-7.8491146	4.19E-15	7.82E-14	SMARCC1
901.2115223	0.559429342	0.0701458	7.9752368	1.52E-15	2.99E-14	SHISA5
374.3420172	-0.42583217	0.09450292	-4.5060215	6.61E-06	3.92E-05	PFKFB4
37.46502112	2.051659564	0.36971923	5.5492368	2.87E-08	2.49E-07	UCN2
1517.792608	1.582129951	0.14735975	10.736514	6.86E-27	3.55E-25	COL7A1
473.2692298	0.754295591	0.09655569	7.8120264	5.63E-15	1.04E-13	SLC26A6
367.4236344	0.345765969	0.10563675	3.27316	0.0010635	0.004075509	CELSR3
1069.528347	0.30793479	0.0606627	5.0761805	3.85E-07	2.81E-06	IP6K2
1739.729697	-0.24711694	0.05950268	-4.1530389	3.28E-05	0.000171851	PRKAR2A
186.0775894	0.636272226	0.13651878	4.6606936	3.15E-06	1.99E-05	SLC25A20
1683.68883	-0.26567098	0.05255941	-5.0546795	4.31E-07	3.13E-06	WDR6
2466.974243	-0.31003829	0.04605828	-6.731435	1.68E-11	2.13E-10	IMPDH2
1724.661671	-0.1800198	0.04893584	-3.67869	0.0002344	0.001042133	QRICH1
1827.968301	-0.22672882	0.0494355	-4.5863563	4.51E-06	2.77E-05	QARS1
1970.114963	0.481230367	0.05541029	8.6848549	3.79E-18	9.46E-17	LAMB2
569.1779659	0.72503285	0.08379104	8.6528684	5.02E-18	1.23E-16	TCTA
927.4569383	-0.29235985	0.06270296	-4.6626163	3.12E-06	1.97E-05	APEH
389.7096035	0.317628075	0.09733438	3.2632669	0.0011014	0.004204743	GMPPB
59.40109093	1.158456712	0.2434747	4.7580168	1.96E-06	1.28E-05	UBA7
342.8294484	0.352278542	0.10074679	3.4966727	0.0004711	0.001967913	TRAIP
1446.869736	0.318600784	0.05877164	5.4209954	5.93E-08	4.90E-07	RBM5
349.3131953	0.405126467	0.10206901	3.9691425	7.21E-05	0.000354242	SEMA3F
5905.673473	0.20099494	0.04622981	4.3477346	1.38E-05	7.72E-05	GNAI2
169.4260494	0.443338037	0.13823357	3.2071661	0.0013405	0.005014243	SEMA3B
420.4451192	0.726356521	0.09302299	7.808355	5.79E-15	1.07E-13	RASSF1
209.4923328	-0.29694223	0.12181275	-2.4376942	0.0147813	0.041850596	MAPKAPK3
1039.322722	-0.29681219	0.06011906	-4.937073	7.93E-07	5.50E-06	DCAF1
231.0314348	0.395680593	0.12022536	3.2911574	0.0009978	0.003856648	TEX264
43.66645245	1.736142269	0.31133541	5.576437	2.45E-08	2.15E-07	GRM2
249.2060767	0.3988568	0.11558046	3.4509016	0.0005587	0.002291532	PCBP4
290.1326487	0.278361324	0.10851651	2.5651519	0.0103131	0.030657123	ABHD14B
2782.698827	-0.17160449	0.05298009	-3.2390374	0.0011993	0.004538955	RPL29
791.5333247	0.234624405	0.06826195	3.4371184	0.0005879	0.002400622	ALAS1
290.3835879	0.365398757	0.11160329	3.2740858	0.00106	0.00406397	TWF2
30.39039138	3.303569599	0.5543091	5.9597968	2.53E-09	2.53E-08	SEMA3G
12.02220744	2.009516275	0.656752	3.0597794	0.002215	0.007878678	TNNC1
1246.071036	-0.21817288	0.05637631	-3.8699389	0.0001089	0.000515556	NT5DC2
86.96771322	0.558934669	0.19041474	2.9353539	0.0033317	0.01130948	SMIM4
2608.479765	-0.36283195	0.05253181	-6.9068992	4.95E-12	6.72E-11	PBRM1
2267.305829	0.172496244	0.05252055	3.2843573	0.0010222	0.003935185	GNL3

723.1497711	-0.24221014	0.0723259	-3.3488716	0.0008114	0.003204016	GLT8D1
638.5119267	-0.25581218	0.07262451	-3.5223947	0.0004277	0.001800758	NEK4
45.5404574	2.553059093	0.34888572	7.3177518	2.52E-13	3.93E-12	ITIH3
493.104029	-0.24693312	0.08187007	-3.0161585	0.00256	0.008950199	SFMBT1
3749.49145	-0.38490281	0.04118249	-9.3462723	9.08E-21	2.86E-19	TKT
792.7622793	-0.39327944	0.06747264	-5.8287245	5.59E-09	5.31E-08	DCP1A
1710.444597	-0.59054216	0.05381626	-10.973303	5.14E-28	2.89E-26	TASOR
131.9333087	0.602181836	0.16340117	3.685297	0.0002284	0.00101833	ARHGEF3
1819.119912	-0.49036861	0.05050044	-9.7101849	2.73E-22	9.74E-21	APPL1
925.1883914	0.248553334	0.07859066	3.1626318	0.0015635	0.005770215	PDE12
5769.6094	0.242031967	0.03917702	6.1779063	6.50E-10	7.00E-09	ARF4
11058.42609	0.12715271	0.0376617	3.3761805	0.000735	0.002925519	FLNB
1571.661439	-0.46646004	0.11224513	-4.1557264	3.24E-05	0.0001701	PTPRG
110.4435377	0.429177281	0.17248928	2.4881388	0.0128414	0.037107219	PTPRG-AS1
277.1657089	0.855266669	0.12062916	7.0900492	1.34E-12	1.92E-11	PRICKLE2
354.4484203	-0.25760059	0.09247433	-2.7856444	0.0053421	0.01718777	LRIG1
103.6259043	1.436877029	0.20248718	7.0961384	1.28E-12	1.84E-11	KBTBD8
772.7720264	-0.67757465	0.07117379	-9.5200029	1.73E-21	5.76E-20	SUCLG2
2067.58943	-0.24378766	0.04792373	-5.0869926	3.64E-07	2.66E-06	TMF1
1399.381738	-0.1810001	0.05411354	-3.3448209	0.0008234	0.003246017	UBA3
1147.920077	0.335316071	0.06451391	5.1975781	2.02E-07	1.55E-06	ARL6IP5
492.5357094	0.409403438	0.08525172	4.8022892	1.57E-06	1.04E-05	MITF
18.85730059	1.108399749	0.44851306	2.4712764	0.0134632	0.038634416	ENSG00000270562
602.0192058	0.298410876	0.07927826	3.7640948	0.0001672	0.000766561	SHQ1
2339.153265	-0.14990179	0.05094414	-2.9424736	0.003256	0.011074148	PPP4R2
201.3154498	1.259958215	0.14903122	8.454324	2.81E-17	6.43E-16	PDZRN3
328.856819	0.463068006	0.10833024	4.2745958	1.91E-05	0.000104616	ROBO1
2307.675964	-0.38257719	0.05332677	-7.1742046	7.27E-13	1.08E-11	GBE1
2731.340077	-0.31830695	0.04726455	-6.7345815	1.64E-11	2.08E-10	CGGBP1
135.3034199	-0.41555136	0.16381117	-2.5367706	0.011188	0.032942727	PROS1
183.1898858	-0.59930857	0.13740095	-4.3617498	1.29E-05	7.29E-05	DHFR2
1927.694258	-0.27514154	0.06550682	-4.2001971	2.67E-05	0.000141866	CRYBG3
886.5026632	-0.20723604	0.06798095	-3.0484427	0.0023003	0.008142143	CPOX
121.5016748	0.576898483	0.1565442	3.6852116	0.0002285	0.00101841	ST3GAL6
11223.25846	-0.49299562	0.04460115	-11.053428	2.11E-28	1.21E-26	DCBLD2
137.1581459	0.482822273	0.15150393	3.186863	0.0014382	0.005341979	LINC00973
148.254912	1.037766066	0.15187957	6.8328219	8.33E-12	1.09E-10	ENSG00000243089
5332.160194	-1.46740148	0.05618694	-26.116417	2.37E-150	5.90E-147	COL8A1
849.695494	-0.29658097	0.06668967	-4.44718	8.70E-06	5.08E-05	CMSS1
1994.925033	-0.2366572	0.05336643	-4.4345704	9.23E-06	5.37E-05	TBC1D23
3156.152244	-0.21514623	0.04041203	-5.3238162	1.02E-07	8.14E-07	TOMM70
3170.941398	-0.18029322	0.04414849	-4.0837913	4.43E-05	0.000226483	TFG
124.9588196	-1.53607317	0.16909582	-9.0840396	1.05E-19	3.01E-18	ABI3BP
568.4995435	-0.64275741	0.0885554	-7.2582523	3.92E-13	5.98E-12	SENP7
3678.058524	-0.37710844	0.04135222	-9.1194253	7.55E-20	2.20E-18	PCNP
1856.759918	-0.1163747	0.04856709	-2.3961641	0.0165677	0.04611918	ZBTB11
6110.09698	-0.27966611	0.0432691	-6.4634142	1.02E-10	1.21E-09	RPL24
1318.902549	-0.54717443	0.05929981	-9.2272537	2.78E-20	8.41E-19	CEP97

770.8828466	-0.18993875	0.06725298	-2.8242427	0.0047392	0.015462575	NXPE3
624.0597187	0.403824051	0.09956734	4.0557883	5.00E-05	0.000252661	NFKBIZ
15795.16187	-0.10359589	0.04231285	-2.4483316	0.0143519	0.040814455	ALCAM
372.6256662	0.50730102	0.10046499	5.0495305	4.43E-07	3.21E-06	DUBR
4006.535069	-0.51748842	0.04367372	-11.848966	2.18E-32	1.58E-30	BBX
1336.365251	0.330548637	0.06133454	5.3892742	7.07E-08	5.78E-07	CD47
418.8716081	-0.43021024	0.08983869	-4.7886967	1.68E-06	1.11E-05	IFT57
2322.643347	-0.13860897	0.04924781	-2.8145207	0.004885	0.015872611	CIP2A
1318.701528	0.169366039	0.06028984	2.809197	0.0049665	0.016107399	DZIP3
173.734409	1.49647755	0.14949309	10.010346	1.37E-23	5.38E-22	NECTIN3-AS1
18.56340556	1.445041269	0.45844797	3.1520289	0.0016214	0.005967446	ENSG00000289069
727.0164079	0.733953414	0.08278986	8.8652571	7.63E-19	2.04E-17	PLCXD2
370.9241503	0.824807427	0.10094824	8.1705978	3.07E-16	6.41E-15	C3orf52
48.59544078	1.401617866	0.27719846	5.0563696	4.27E-07	3.10E-06	TBILA
1530.855299	-0.27418629	0.05731024	-4.7842464	1.72E-06	1.13E-05	ATG3
966.7869718	-0.24837363	0.06915375	-3.5916147	0.0003286	0.001413897	SLC35A5
3664.412649	-1.05809282	0.05758775	-18.373576	2.14E-75	6.64E-73	CCDC80
1083.467731	-0.18534341	0.06323908	-2.9308365	0.0033805	0.011441613	NEPRO
569.5377112	-0.85464361	0.08640022	-9.8916831	4.52E-23	1.73E-21	USF3
5261.540966	-0.23023743	0.04100012	-5.6155301	1.96E-08	1.74E-07	NAA50
2287.505087	-0.13168547	0.05292869	-2.4879789	0.0128471	0.037111579	ATP6V1A
154.1342599	-0.45164449	0.15255926	-2.9604528	0.0030719	0.010528153	GRAMD1C
825.3402031	-0.21182138	0.06728137	-3.1482915	0.0016423	0.006035338	QTRT2
128.181523	-0.82720484	0.16638105	-4.9717491	6.64E-07	4.66E-06	ZBTB20
1234.964446	0.297905397	0.06298771	4.7295794	2.25E-06	1.45E-05	B4GALT4
320.4503284	0.650053868	0.10721185	6.0632652	1.33E-09	1.39E-08	ARHGAP31
877.8810084	-0.28137311	0.06435117	-4.3724631	1.23E-05	6.99E-05	TMEM39A
3167.70966	-0.133121	0.04408812	-3.0194302	0.0025325	0.008864792	GSK3B
879.6117871	-0.37835346	0.0714751	-5.2935001	1.20E-07	9.49E-07	NDUFB4
648.1976679	-0.44359349	0.07415909	-5.981647	2.21E-09	2.22E-08	RABL3
6.344562834	2.213580376	0.82251662	2.6912287	0.0071189	0.022142426	HCLS1
4145.999671	-0.33908989	0.10069804	-3.3673932	0.0007588	0.003013463	GOLGB1
501.1716094	-0.31803636	0.08264775	-3.8480944	0.000119	0.000559036	MIX23
1097.644688	-0.2882798	0.07124603	-4.0462576	5.20E-05	0.000261947	FAM162A
537.7061117	1.674562359	0.22343888	7.4944985	6.66E-14	1.10E-12	PARP9
1061.395724	0.832715416	0.13566742	6.1379172	8.36E-10	8.87E-09	DTX3L
1314.353423	0.863803793	0.12530211	6.8937687	5.43E-12	7.31E-11	PARP14
303.9369874	0.320873192	0.10895671	2.9449603	0.00323	0.010994134	PDIA5
4191.31418	-0.22918238	0.04363399	-5.2523816	1.50E-07	1.17E-06	HACD2
804.5934158	0.345170928	0.07022652	4.915108	8.87E-07	6.10E-06	ITGB5
2356.310119	-0.38337261	0.05427853	-7.0630612	1.63E-12	2.30E-11	ZNF148
1062.811814	0.218118232	0.06195346	3.5206786	0.0004304	0.001810697	OSBPL11
138.0972553	-0.54719024	0.14730781	-3.7146043	0.0002035	0.000918331	CHCHD6
1405.852306	0.246837948	0.05973053	4.1325256	3.59E-05	0.000186365	PLXNA1
2703.43242	-0.43595491	0.04607402	-9.4620542	3.02E-21	9.91E-20	MCM2
105.8087466	0.530343499	0.18343071	2.8912471	0.0038372	0.012817568	PODXL2
543.9254908	0.323132143	0.08857494	3.6481215	0.0002642	0.001161817	MGLL
6631.59076	0.142072516	0.03540795	4.0124462	6.01E-05	0.000299002	SEC61A1

1621.641698	-0.35428518	0.05744981	-6.1668646	6.97E-10	7.48E-09	RUVBL1
143.5095205	-0.6835763	0.14729133	-4.6409813	3.47E-06	2.17E-05	EEFSEC
10.05568768	2.478539861	0.69763832	3.5527576	0.0003812	0.001619163	ENSG00000231305
16.94229613	1.385941812	0.48175601	2.8768542	0.0040166	0.013347767	ENSG00000287110
215.1474433	-0.29587309	0.12109232	-2.4433679	0.0145509	0.041278973	ISY1
5313.825192	-0.1480945	0.04103092	-3.6093392	0.000307	0.001327287	CNBP
3596.94763	0.160524435	0.0472608	3.3965661	0.0006824	0.002737985	COPG1
1124.51613	-0.66410535	0.06912791	-9.6069063	7.48E-22	2.56E-20	H1-10
418.854402	-0.2500098	0.09997441	-2.5007378	0.0123935	0.035980432	IFT122
820.0826696	0.309781354	0.07313618	4.2356785	2.28E-05	0.000122527	PLXND1
89.08164418	0.57654635	0.19489168	2.9582912	0.0030935	0.010589746	TMCC1-DT
38.05827684	1.014861092	0.30127185	3.3685891	0.0007555	0.003002476	FAM86HP
1409.738343	-0.39967297	0.05182167	-7.7124682	1.23E-14	2.22E-13	PIK3R4
799.4565568	-0.41033325	0.07200669	-5.6985434	1.21E-08	1.11E-07	NUDT16
27.51857018	-1.01178624	0.33074392	-3.0591227	0.0022199	0.007894356	ENSG00000261167
2119.756349	-0.41943431	0.04893477	-8.5712938	1.02E-17	2.44E-16	MRPL3
2691.81037	-0.39823677	0.05002444	-7.9608438	1.71E-15	3.33E-14	DNAJC13
3612.072184	-0.31764951	0.04803049	-6.6134977	3.75E-11	4.62E-10	TOPBP1
1183.686197	0.342031432	0.06597735	5.1840738	2.17E-07	1.65E-06	SRPRB
22.28697767	0.998039083	0.42160663	2.3672282	0.0179219	0.049243404	ENSG00000260633
948.5539403	-0.77596048	0.06462844	-12.006487	3.29E-33	2.48E-31	PPP2R3A
746.2800656	-0.28021096	0.06912338	-4.0537797	5.04E-05	0.000254471	MSL2
729.5656971	-0.18105984	0.06930017	-2.6126899	0.0089833	0.027116681	PCCB
1786.608856	-0.71526492	0.04964508	-14.407568	4.64E-47	6.06E-45	STAG1
266.9421236	0.345929637	0.11822689	2.925981	0.0034337	0.011605902	SLC35G2
327.6963553	0.627863798	0.11662727	5.3835077	7.30E-08	5.96E-07	MRAS
564.6138897	-0.57002873	0.07839572	-7.2711718	3.56E-13	5.45E-12	CEP70
1396.046068	-0.43917674	0.0551777	-7.9593153	1.73E-15	3.37E-14	PIK3CB
1093.845079	-0.24686346	0.05804308	-4.2531074	2.11E-05	0.000114106	MRPS22
6000.344981	-0.18147619	0.03741216	-4.8507275	1.23E-06	8.28E-06	COPB2
319.7026788	1.112691689	0.12145322	9.161484	5.12E-20	1.52E-18	CLSTN2
3070.455674	-0.29446857	0.04773788	-6.1684473	6.90E-10	7.42E-09	SLC25A36
247.1120044	-0.32086406	0.11982055	-2.6778717	0.0074092	0.022918053	PXYLP1
3738.09704	-0.32534027	0.04213395	-7.7215704	1.15E-14	2.07E-13	ZBTB38
694.4448908	-0.45329777	0.07255625	-6.2475358	4.17E-10	4.62E-09	RASA2
3571.908407	0.36505704	0.05121272	7.128249	1.02E-12	1.48E-11	ATP1B3
1097.535546	-0.35898452	0.06079188	-5.905139	3.52E-09	3.43E-08	TFDP2
1335.624392	-0.72867336	0.06073262	-11.998055	3.64E-33	2.74E-31	XRN1
1263.50056	-0.4156982	0.05781818	-7.1897491	6.49E-13	9.64E-12	ATR
27.48199105	0.812435244	0.32666801	2.4870364	0.0128812	0.037191504	PAQR9
241.0417372	1.127252663	0.13089776	8.6117033	7.20E-18	1.75E-16	CHST2
98.91574897	-0.87359585	0.19341085	-4.5167882	6.28E-06	3.73E-05	ENSG00000261051
28915.43033	-0.14283128	0.03750282	-3.8085476	0.0001398	0.000649097	PLOD2
160.6675073	0.455396927	0.15039538	3.0279982	0.0024618	0.008641628	PLSCR4
745.1204172	1.142393249	0.09453178	12.084753	1.27E-33	9.74E-32	PLSCR1
1153.473723	0.177613098	0.06140356	2.8925537	0.0038212	0.012766829	GYG1
4735.905014	-0.46709433	0.04509878	-10.357139	3.88E-25	1.75E-23	HLTF
930.1966673	-0.25465235	0.06564436	-3.8792722	0.0001048	0.00049807	HPS3

14.0146756	5.279898952	1.25095303	4.2207012	2.44E-05	0.000130274	ENSG00000244468
914.1652706	0.553402738	0.07775066	7.1176594	1.10E-12	1.59E-11	TM4SF1
2460.476706	0.356219582	0.05017341	7.0997685	1.25E-12	1.80E-11	WWTR1
1146.10364	-0.24337644	0.05852298	-4.1586473	3.20E-05	0.000168193	COMMD2
3105.650939	-0.13364502	0.05182895	-2.5785786	0.0099208	0.029642999	PFN2
2218.842305	-0.45859378	0.04678038	-9.8031214	1.09E-22	4.06E-21	EIF2A
23.11646849	2.327263022	0.43861771	5.305903	1.12E-07	8.91E-07	GPR87
7170.87	-0.24953211	0.04434955	-5.626486	1.84E-08	1.64E-07	MBNL1
140.093016	0.742320848	0.16257398	4.5660497	4.97E-06	3.02E-05	MBNL1-AS1
728.1694897	0.31138737	0.07812479	3.9857689	6.73E-05	0.000332108	RAP2B
2258.956892	-0.14765983	0.04845675	-3.0472497	0.0023095	0.008169543	DHX36
3657.173978	-0.489043	0.03921029	-12.472312	1.06E-35	8.92E-34	GMPS
2143.423515	0.454093749	0.06181678	7.345801	2.05E-13	3.22E-12	TIPARP
47.9679308	0.998829325	0.27825014	3.5896813	0.0003311	0.001423368	LINC00886
9.169420146	1.67979896	0.69332149	2.4228284	0.0154002	0.04330684	LEKR1
618.0723215	1.526857006	0.08967556	17.026457	5.23E-65	1.21E-62	PTX3
883.6778912	-0.36690714	0.06377803	-5.7528768	8.77E-09	8.15E-08	RSRC1
512.7766886	-0.2680679	0.08551732	-3.1346619	0.0017205	0.006294948	MLF1
164.3043926	0.455694089	0.13915596	3.2747004	0.0010577	0.004056034	IQCJ-SCHIP1
74.43972377	0.657360771	0.22218429	2.9586284	0.0030901	0.010584868	SCHIP1
587.7818068	-0.40742859	0.07520717	-5.4174171	6.05E-08	4.99E-07	IFT80
13953.69105	-0.53832141	0.03122771	-17.238579	1.36E-66	3.39E-64	SMC4
1627.871028	-0.47581913	0.06633065	-7.1734429	7.31E-13	1.08E-11	TRIM59
68.67369742	0.608192593	0.21407155	2.8410716	0.0044962	0.014744353	KRT8P12
115.2771866	0.757199175	0.1905689	3.9733617	7.09E-05	0.000348712	PPM1L
124.4709457	0.542572222	0.16860778	3.2179549	0.0012911	0.004849223	B3GALNT1
1776.136287	-0.20466502	0.0532642	-3.8424499	0.0001218	0.000571283	NMD3
146.7361017	0.810246999	0.15784063	5.1333235	2.85E-07	2.13E-06	LINC01322
39.17440252	1.751581588	0.36168378	4.8428536	1.28E-06	8.59E-06	SERPINI1
3.426820874	3.628177819	1.41286224	2.5679629	0.0102298	0.030435655	LRRC31
2091.101906	-0.55982906	0.05328436	-10.506442	8.07E-26	3.82E-24	PHC3
3921.619419	-0.2042341	0.04475181	-4.5637056	5.03E-06	3.05E-05	PRKCI
419.0807458	-0.59748779	0.08794204	-6.794109	1.09E-11	1.41E-10	CLDN11
411.74217	-0.22380206	0.09116465	-2.4549216	0.0140915	0.040225168	RPL22L1
1231.816139	0.288205943	0.06059828	4.7560084	1.97E-06	1.29E-05	EIF5A2
4641.219213	0.186235633	0.05096331	3.6543082	0.0002579	0.001135596	NCEH1
5755.883055	-0.30042914	0.03680157	-8.1634879	3.25E-16	6.79E-15	ECT2
1894.923836	-0.67851317	0.05602847	-12.11015	9.33E-34	7.28E-32	TBL1XR1
1453.994195	0.593896669	0.06661211	8.9157468	4.85E-19	1.32E-17	ZMAT3
1310.165313	-0.33526129	0.06214131	-5.3951436	6.85E-08	5.60E-07	PIK3CA
1811.991234	-0.43981994	0.05137742	-8.5605686	1.12E-17	2.67E-16	MFN1
1929.680674	-0.29151877	0.05022829	-5.8038755	6.48E-09	6.12E-08	ACTL6A
766.1691416	-0.50174295	0.07039449	-7.1275886	1.02E-12	1.49E-11	USP13
1800.700539	0.454229692	0.05288374	8.5892127	8.76E-18	2.11E-16	TTC14
7462.3162	-0.29668533	0.04607789	-6.4387787	1.20E-10	1.42E-09	FXR1
2501.134255	-0.34534418	0.04872421	-7.0877326	1.36E-12	1.95E-11	ATP11B
20.26815392	1.292308272	0.4100538	3.1515579	0.001624	0.005974586	LAMP3
87.48090501	0.722853166	0.21387091	3.3798574	0.0007252	0.002891295	LINC00888

2898.833171	-0.29366754	0.05101467	-5.7565315	8.59E-09	8.00E-08	YEATS2
1022.989023	0.192660027	0.06904484	2.7903612	0.0052649	0.016961299	ABCC5
12909.27865	-0.09320736	0.03607817	-2.5834836	0.0097808	0.029275095	EIF4G1
120.1424377	0.528874058	0.16927262	3.1243923	0.0017817	0.006492925	CLCN2
47.41564057	1.294217577	0.32378954	3.997095	6.41E-05	0.000317519	EPHB3
14.14230348	2.517466322	0.628574	4.0050437	6.20E-05	0.00030797	C3orf70
490.711263	-0.48916594	0.08287316	-5.9025855	3.58E-09	3.48E-08	MAP3K13
25.0137353	0.956715728	0.37298665	2.5650133	0.0103172	0.030664133	LIPH
1707.25512	-0.15931303	0.05471451	-2.9117145	0.0035945	0.012088286	SENP2
3635.77361	-0.54699186	0.04047118	-13.515588	1.27E-41	1.37E-39	IGF2BP2
4406.291039	-0.12328132	0.04547367	-2.7110485	0.0067071	0.021053457	TRA2B
1176.059388	0.241793805	0.06936243	3.485948	0.0004904	0.002041655	ETV5
362.472677	-0.24712986	0.0966665	-2.5565202	0.0105725	0.031344604	TBCCD1
1661.500054	0.220756392	0.05322113	4.1479084	3.36E-05	0.000175589	DNAJB11
9392.087626	-0.23099749	0.04671812	-4.9444944	7.63E-07	5.32E-06	EIF4A2
1520.52239	-0.26452023	0.0565257	-4.6796456	2.87E-06	1.82E-05	RFC4
55.87203176	0.696202514	0.24791357	2.8082469	0.0049812	0.016144206	LPP-AS2
5603.847945	-0.51037611	0.10729	-4.7569773	1.97E-06	1.28E-05	LPP
548.2805314	0.428685061	0.08821101	4.859768	1.18E-06	7.93E-06	P3H2
2673.889024	1.068053297	0.04983496	21.431809	6.75E-102	4.35E-99	CLDN1
576.1593788	0.501045062	0.0828442	6.04804	1.47E-09	1.51E-08	IL1RAP
2196.731053	-0.21646545	0.04805109	-4.5049022	6.64E-06	3.94E-05	CCDC50
3321.408858	-0.3119002	0.04931863	-6.3241865	2.55E-10	2.89E-09	OPA1
49.76615718	1.924763769	0.33184427	5.800202	6.62E-09	6.25E-08	LRRC15
7084.401431	0.148699162	0.04926736	3.0182086	0.0025427	0.008898815	ATP13A3
14.17830302	1.640979147	0.54842701	2.992156	0.0027701	0.009603838	ATP13A3-DT
1594.925758	0.230987482	0.05893515	3.9193501	8.88E-05	0.00042842	LSG1
21.82941641	2.019644769	0.51041881	3.9568384	7.59E-05	0.000371096	FAM43A
192.7843347	0.333948771	0.13203281	2.5292863	0.0114295	0.033568412	XXYLT1
2720.231706	-0.32546806	0.05064627	-6.4262992	1.31E-10	1.53E-09	ACAP2
1354.755074	0.288632757	0.0633411	4.5568005	5.19E-06	3.14E-05	PPP1R2
8412.750602	0.281579894	0.05612344	5.0171532	5.24E-07	3.75E-06	TFRC
2884.456597	-0.54029356	0.05094869	-10.60466	2.83E-26	1.38E-24	UBXN7
6521.574086	-0.14187527	0.03803875	-3.7297564	0.0001917	0.000868435	PAK2
2344.223345	-0.2736588	0.04597818	-5.9519271	2.65E-09	2.64E-08	NCBP2
252.1203569	0.96543018	0.12374175	7.8019759	6.09E-15	1.12E-13	MELTF
3322.106799	-0.4713275	0.04067081	-11.588839	4.69E-31	3.12E-29	DLG1
1354.888311	-0.23691076	0.05569308	-4.2538636	2.10E-05	0.000113811	LRCH3
176.0435766	-0.40683305	0.13906326	-2.925525	0.0034388	0.011620667	IQCG
5868.55164	-0.32640945	0.03902437	-8.3642474	6.05E-17	1.35E-15	RPL35A
431.939298	-0.46214984	0.09792168	-4.7195864	2.36E-06	1.52E-05	LMLN
367.2855156	-0.26437328	0.09535403	-2.7725443	0.005562	0.017822571	ZNF718
642.3434106	-0.35654436	0.07785878	-4.5793726	4.66E-06	2.85E-05	ZNF141
707.5957378	-0.35182746	0.07267467	-4.8411292	1.29E-06	8.66E-06	ZNF721
153.7137991	0.582070415	0.15425447	3.7734428	0.000161	0.000739558	TMEM175
226.205946	0.598740336	0.11799985	5.0740772	3.89E-07	2.84E-06	DGKQ
101.505071	0.909308199	0.19533224	4.6551875	3.24E-06	2.04E-05	IDUA
958.0177619	-0.38076722	0.06514589	-5.8448387	5.07E-09	4.84E-08	FGFRL1

351.4942267	0.781178872	0.10916061	7.1562341	8.29E-13	1.22E-11	FGFR3
6068.435838	-0.46404755	0.03756362	-12.353642	4.66E-35	3.78E-33	NSD2
427.4015755	-0.29070387	0.08567996	-3.3929039	0.0006916	0.002772294	NELFA
1082.09316	0.219416001	0.06217498	3.5290078	0.0004171	0.001761469	HAUS3
643.0585644	0.408079251	0.08015509	5.0911207	3.56E-07	2.61E-06	MXD4
1237.345879	-0.36345874	0.05934755	-6.1242418	9.11E-10	9.62E-09	SH3BP2
4341.986248	-0.14606281	0.03958678	-3.6896866	0.0002245	0.001001946	ADD1
1598.123634	0.525724362	0.05517702	9.527958	1.60E-21	5.34E-20	LRPAP1
27.58148329	1.352840435	0.37303291	3.6265981	0.0002872	0.001249807	NSG1
69.48550668	0.641762298	0.21996377	2.9175818	0.0035276	0.011880602	MSX1
56.21313013	1.284379986	0.27523183	4.6665387	3.06E-06	1.94E-05	CYTL1
770.2321026	0.33452432	0.06903402	4.8457891	1.26E-06	8.47E-06	MAN2B2
222.0329714	0.589734209	0.12632472	4.6683992	3.04E-06	1.92E-05	ENSG00000170846
1228.761287	-0.25851896	0.0599594	-4.3115665	1.62E-05	8.97E-05	TBC1D14
4144.096371	-0.22952038	0.04046399	-5.6722135	1.41E-08	1.27E-07	AFAP1
26.32380335	1.73352109	0.41871153	4.1401322	3.47E-05	0.000180832	HTRA3
5.282528766	4.204847561	1.37939254	3.0483328	0.0023011	0.008143464	SLC2A9
40.09471835	1.206818201	0.29399672	4.1048696	4.05E-05	0.00020789	ENSG00000261490
15166.14429	0.264239313	0.03541158	7.4619474	8.53E-14	1.39E-12	WDR1
1701.04048	-0.20385187	0.05582471	-3.6516423	0.0002606	0.001146871	ZNF518B
312.1396991	-0.50540613	0.11255082	-4.4904704	7.11E-06	4.20E-05	RAB28
3752.409049	-0.34702116	0.04589611	-7.561015	4.00E-14	6.82E-13	BOD1L1
501.5388276	0.54655265	0.08963883	6.0972757	1.08E-09	1.13E-08	CPEB2
292.5547761	-0.3908861	0.1008503	-3.8759044	0.0001062	0.000504184	CC2D2A
314.4674704	0.5165705	0.11007934	4.6927107	2.70E-06	1.72E-05	FAM200B
410.0617868	0.291933541	0.09339595	3.1257622	0.0017734	0.006464987	QDPR
1432.455786	0.44516214	0.05976631	7.4483798	9.45E-14	1.54E-12	LAP3
1636.590587	-0.20869523	0.05111845	-4.0825811	4.45E-05	0.000227532	DCAF16
4518.712649	-0.16211643	0.04179162	-3.879161	0.0001048	0.000498162	NCAPG
487.2433523	-0.54939165	0.084272	-6.5192667	7.07E-11	8.51E-10	LCORL
420.0925215	0.928522982	0.09697983	9.5743925	1.02E-21	3.44E-20	SLIT2
19.35371953	1.585183433	0.50887861	3.1150522	0.0018391	0.006676817	PPARGC1A
5288.865406	-0.2250507	0.03803138	-5.9174997	3.27E-09	3.20E-08	DHX15
115.9605849	-0.42982175	0.17369088	-2.4746362	0.0133372	0.03832398	CCDC149
338.7746305	-0.44209535	0.10640453	-4.1548548	3.25E-05	0.000170698	ZCCHC4
4241.427267	-0.47882493	0.04559728	-10.501174	8.53E-26	4.02E-24	RBPJ
977.9927946	0.577756453	0.07004938	8.2478454	1.61E-16	3.45E-15	STIM2
14.13080799	2.533598511	0.59455071	4.2613666	2.03E-05	0.000110554	ENSG00000251410
153.8558228	2.350135924	0.19651164	11.959271	5.81E-33	4.33E-31	PCDH7
292.8231744	-0.44653646	0.11023677	-4.0507035	5.11E-05	0.000257541	ARAP2
108.7646367	1.43288161	0.19884373	7.2060687	5.76E-13	8.60E-12	C4orf19
864.5216916	0.285503423	0.07391773	3.8624486	0.0001123	0.000529847	RELL1
1334.55681	-0.14846401	0.0574771	-2.5830115	0.0097942	0.029310141	TBC1D1
2311.342132	-0.32947186	0.05712069	-5.767995	8.02E-09	7.50E-08	FAM114A1
26.17865404	2.071307681	0.43733107	4.7362464	2.18E-06	1.41E-05	TMEM156
2957.36221	0.356659606	0.04691025	7.60302	2.89E-14	4.99E-13	KLHL5
532.9793084	-0.5465971	0.07985071	-6.8452375	7.63E-12	1.00E-10	WDR19
4128.918271	-0.24215734	0.04155262	-5.827728	5.62E-09	5.34E-08	RFC1

2227.39915	-0.25028666	0.05733553	-4.3652976	1.27E-05	7.19E-05	RPL9
621.3601332	0.376351124	0.08787326	4.2828855	1.84E-05	0.000101077	SMIM14
3344.15052	-0.15705447	0.04532227	-3.4652827	0.0005297	0.002183788	UBE2K
4919.373304	-0.37773179	0.04283226	-8.8188624	1.16E-18	3.04E-17	PDS5A
5176.557839	0.251544362	0.04436536	5.6698375	1.43E-08	1.29E-07	UCHL1
3207.03229	-0.20511363	0.05525683	-3.7120048	0.0002056	0.000926374	LIMCH1
1766.801918	-0.15201246	0.05429104	-2.799955	0.005111	0.016520411	SLC30A9
1257.029947	-0.17377045	0.06330734	-2.7448703	0.0060535	0.01923443	GUF1
573.7441755	-0.24631237	0.07871485	-3.129173	0.001753	0.006397589	GNPDA2
789.0455252	0.366057813	0.07001394	5.2283559	1.71E-07	1.32E-06	ATP10D
174.6876142	0.325853586	0.13705654	2.377512	0.0174299	0.04810435	TEC
2597.880475	-0.5090665	0.06704819	-7.5925463	3.14E-14	5.40E-13	FRYL
350.6538239	0.524934195	0.11000988	4.7717004	1.83E-06	1.20E-05	OCIAD2
2085.173817	0.24598294	0.05361535	4.5879197	4.48E-06	2.75E-05	SGCB
767.7182111	-0.26588939	0.07105972	-3.7417737	0.0001827	0.000832924	USP46
333.6287538	-0.32489623	0.10767081	-3.0174961	0.0025487	0.008914381	DANCR
185.628709	-0.66929083	0.13036236	-5.1340804	2.84E-07	2.12E-06	SCFD2
403.9353525	0.229098299	0.0957937	2.3915799	0.016776	0.04658818	CHIC2
2003.240822	1.022634764	0.05582032	18.320116	5.72E-75	1.71E-72	KIT
46.16919982	2.206252762	0.36291019	6.0793354	1.21E-09	1.26E-08	KDR
1386.687831	-0.41756225	0.05774731	-7.2308519	4.80E-13	7.25E-12	CLOCK
295.9627147	-0.51682453	0.10539088	-4.9038827	9.40E-07	6.43E-06	AASDH
7032.546592	-0.39872047	0.04072372	-9.7908661	1.23E-22	4.53E-21	PAICS
3938.557919	-0.32157858	0.04675615	-6.8777812	6.08E-12	8.11E-11	SRP72
2050.657791	-0.2809544	0.05658619	-4.96507	6.87E-07	4.81E-06	REST
2757.282945	1.699011542	0.05996081	28.335367	1.27E-176	5.51E-173	IGFBP7
50.07651872	0.685143425	0.25716635	2.6642032	0.0077171	0.02374385	ADGRL3
2822.242067	-0.41373978	0.0489065	-8.4598119	2.68E-17	6.17E-16	UBA6
2561.999533	0.125324675	0.04285622	2.9243052	0.0034523	0.011661774	YTHDC1
373.9523959	-0.38418777	0.09272956	-4.1430992	3.43E-05	0.000178668	RUFY3
237.9212192	0.626566001	0.12351305	5.0728731	3.92E-07	2.86E-06	SLC4A4
500.9114043	0.31026504	0.09730391	3.1886183	0.0014295	0.005313056	ADAMTS3
146.0179535	-0.39622002	0.14910421	-2.6573363	0.0078761	0.02416036	COX18
3306.24282	-0.4123248	0.05037818	-8.1845917	2.73E-16	5.75E-15	ANKRD17
175.3137252	1.676406063	0.15368317	10.908195	1.05E-27	5.72E-26	CXCL8
75.15318528	2.928724142	0.26910735	10.883107	1.39E-27	7.49E-26	CXCL1
47.48914205	2.120547191	0.28760257	7.3731857	1.67E-13	2.65E-12	CXCL5
389.0064577	-0.27320193	0.09480781	-2.8816393	0.0039561	0.013176972	RCHY1
5398.891681	-0.20211301	0.04317003	-4.6817902	2.84E-06	1.81E-05	G3BP2
3381.029793	-0.25374362	0.04416798	-5.7449681	9.19E-09	8.53E-08	USO1
135.6268438	0.388424997	0.15628733	2.4853261	0.0129433	0.037341442	NAAA
1329.670104	-0.29637107	0.05392997	-5.4954796	3.90E-08	3.31E-07	NUP54
2635.397737	0.134268741	0.05105728	2.6297671	0.0085443	0.025958431	SCARB2
245.1096162	0.532293483	0.12656493	4.2056948	2.60E-05	0.000138546	SHROOM3
5184.503254	-0.67301989	0.04283758	-15.71097	1.27E-55	2.28E-53	CCNI
1929.661125	-0.23411767	0.05444912	-4.2997512	1.71E-05	9.43E-05	CCNG2
620.5269993	-0.50827572	0.07916897	-6.4201385	1.36E-10	1.59E-09	CNOT6L
461.0261172	-0.30593418	0.09174553	-3.3345948	0.0008542	0.003354074	MRPL1

13.4922394	1.801878684	0.65332685	2.7580049	0.0058155	0.018535943	FRAS1
685.3552476	0.380447405	0.07198296	5.2852423	1.26E-07	9.90E-07	ANXA3
816.8145304	0.356049015	0.06816892	5.2230406	1.76E-07	1.36E-06	BMP2K
547.3734806	0.982116961	0.08948129	10.975669	5.00E-28	2.82E-26	ANTXR2
366.3886801	-0.34218083	0.10411097	-3.2866934	0.0010137	0.003909612	PRDM8
6846.542146	-0.29259391	0.05509024	-5.3111752	1.09E-07	8.67E-07	FGF5
7099.152927	-0.44868778	0.03645238	-12.308875	8.11E-35	6.53E-33	HNRNPD
4961.644094	-0.21835254	0.03847142	-5.6757082	1.38E-08	1.25E-07	HNRNPDL
1053.805615	-0.36523536	0.05829466	-6.2653318	3.72E-10	4.14E-09	COPS4
336.2546228	-0.23968996	0.09881776	-2.4255758	0.0152841	0.043036098	PLAC8
282.4024176	-0.31058525	0.11069751	-2.8057112	0.0050206	0.016261438	COQ2
394.4095538	-0.31614258	0.09268967	-3.4107641	0.0006478	0.002613184	HPSE
161.681164	-0.61628762	0.14049432	-4.386566	1.15E-05	6.58E-05	ABRAXAS1
1398.594652	-0.56760818	0.07725737	-7.3469779	2.03E-13	3.20E-12	WDFY3
1559.58221	0.21852006	0.06009887	3.6360094	0.0002769	0.001209239	PTPN13
977.4288744	-0.17756803	0.06107245	-2.907498	0.0036433	0.012231169	KLHL8
6.834089391	2.936351825	1.01234788	2.9005364	0.0037252	0.012479682	SPP1
16.30569021	1.310735803	0.4906595	2.6713756	0.0075541	0.02330836	ENSG00000289034
2058.925682	0.816486343	0.05207431	15.679255	2.10E-55	3.65E-53	PKD2
268.6991206	2.245114396	0.2354047	9.5372541	1.47E-21	4.90E-20	HERC6
346.365608	1.346725111	0.1056304	12.749409	3.14E-37	2.85E-35	HERC5
81.61794976	-0.55214885	0.20128633	-2.7431015	0.0060862	0.019320698	FAM13A
490.8597384	0.248673239	0.08769858	2.8355446	0.0045748	0.014956781	TIGD2
1295.518829	-0.17088349	0.05702475	-2.9966548	0.0027296	0.009472696	SMARCAD1
4803.406488	0.528902986	0.04478109	11.810856	3.43E-32	2.44E-30	PDLIM5
1371.773734	-0.30734112	0.05845227	-5.2579845	1.46E-07	1.14E-06	TSPAN5
728.5503235	-0.18898441	0.07004715	-2.6979599	0.0069766	0.021785385	EIF4E
1934.72981	-0.3575549	0.05501944	-6.4987016	8.10E-11	9.67E-10	METAP1
1868.576405	-0.39447497	0.04928395	-8.0041268	1.20E-15	2.40E-14	PPP3CA
57.36453582	1.251065673	0.26331567	4.751201	2.02E-06	1.31E-05	BANK1
150.3788445	0.81654612	0.1554883	5.2514956	1.51E-07	1.17E-06	SLC39A8
701.8855535	0.218482138	0.07101252	3.0766706	0.0020933	0.007488556	NFKB1
3322.782317	-0.22639701	0.04892111	-4.6277974	3.70E-06	2.30E-05	UBE2D3
69.6595784	-0.61367745	0.21475214	-2.8576081	0.0042685	0.014064737	SLC9B2
723.0526467	-0.23576364	0.08429776	-2.796796	0.0051612	0.016664213	TET2
380.3826123	-0.57996068	0.09973494	-5.8150204	6.06E-09	5.74E-08	PPA2
727.7687235	-0.25973275	0.0707717	-3.6700088	0.0002425	0.001074327	GSTCD
54.27251211	2.010270585	0.35144639	5.7199921	1.07E-08	9.78E-08	NPNT
297.5913855	-0.31259756	0.10223087	-3.0577608	0.00223	0.007927078	TBCK
1477.442718	-0.15839004	0.05202519	-3.0444874	0.0023308	0.008234889	AIMP1
857.7884954	-0.40966754	0.07299374	-5.6123655	2.00E-08	1.77E-07	SGMS2
490.1229909	0.94389503	0.09241178	10.214012	1.72E-24	7.31E-23	CYP2U1
349.7842545	-0.33696851	0.10130623	-3.3262368	0.0008803	0.003447729	HADH
2930.424285	-0.15514968	0.0488413	-3.1766081	0.0014901	0.00551362	RPL34
39.68563014	1.852126398	0.31527799	5.874582	4.24E-09	4.09E-08	COL25A1
1155.19549	-0.25232526	0.05997952	-4.2068566	2.59E-05	0.000137878	SEC24B
292.3764608	-0.36877683	0.10207855	-3.612677	0.0003031	0.001312597	MCUB
415.715206	-0.46316243	0.08782359	-5.2737813	1.34E-07	1.05E-06	PLA2G12A

204.193779	-0.60398318	0.12411259	-4.8664136	1.14E-06	7.69E-06	ELOVL6
232.2064291	0.31517619	0.11862188	2.6569819	0.0078844	0.024175824	PITX2
180.4915132	0.613992508	0.1480847	4.1462252	3.38E-05	0.000176671	FAM241A
661.4458056	-0.27141508	0.08434335	-3.2179784	0.001291	0.004849223	ZGRF1
1628.166198	-0.18376129	0.05879002	-3.1257224	0.0017737	0.006464987	ARSJ
85.32919552	0.563028942	0.19926029	2.8255954	0.0047193	0.015403218	SNHG8
519.3760979	0.368645634	0.08446914	4.3642639	1.28E-05	7.22E-05	PRSS12
782.5482889	-0.3259591	0.06802906	-4.7914684	1.66E-06	1.09E-05	METTL14
1914.142719	-0.12895798	0.04957043	-2.6015102	0.0092814	0.02793425	SEC24D
150.0664811	2.887692592	0.20122586	14.350504	1.06E-46	1.36E-44	SYNPO2
1589.834512	0.427094509	0.05652534	7.5558057	4.16E-14	7.07E-13	USP53
1810.947068	-0.18972186	0.05713673	-3.3204885	0.0008986	0.003509245	C4orf3
51.15591237	0.71987104	0.25916498	2.7776556	0.0054753	0.017570551	PDE5A
11720.61991	0.210311342	0.03701418	5.6819131	1.33E-08	1.21E-07	ANXA5
1344.588524	-0.14055232	0.05580758	-2.5185166	0.011785	0.034452431	EXOSC9
3208.459452	-0.27027179	0.04463964	-6.0545249	1.41E-09	1.46E-08	CCNA2
497.9991296	-0.30626722	0.08361105	-3.6629991	0.0002493	0.001101364	BBS7
1491.187197	-0.7399703	0.14100247	-5.2479242	1.54E-07	1.20E-06	KIAA1109
4088.461606	0.256359065	0.04651681	5.5111063	3.57E-08	3.04E-07	FGF2
475.8805593	-0.2254352	0.08915621	-2.5285417	0.0114537	0.033622669	SPATA5
1206.004236	-0.14729666	0.05928707	-2.4844651	0.0129746	0.037411491	ANKRD50
984.3066593	-0.54102461	0.1453721	-3.7216536	0.0001979	0.000895621	FAT4
1026.702827	0.350578746	0.06313562	5.5527887	2.81E-08	2.44E-07	HSPA4L
1551.115741	-0.2816614	0.05294371	-5.320016	1.04E-07	8.29E-07	PLK4
2550.089544	-0.19884445	0.05002869	-3.9746086	7.05E-05	0.000347087	PGRMC2
1451.057168	-0.46644227	0.05253307	-8.8790221	6.75E-19	1.82E-17	JADE1
568.560495	-0.22739327	0.07857617	-2.8939214	0.0038046	0.012716237	SCLT1
8.530423506	3.891497617	1.13755433	3.4209334	0.0006241	0.002530902	PCDH10-DT
235.2422926	2.072963469	0.14874805	13.936072	3.82E-44	4.43E-42	PCDH10
99.62513332	1.299653078	0.206107	6.3057204	2.87E-10	3.24E-09	PCDH18
573.6797971	-0.30576514	0.10535151	-2.9023328	0.0037039	0.012417901	SLC7A11
504.3923764	0.487355236	0.09187715	5.3044226	1.13E-07	8.97E-07	NOCT
648.8359289	-0.31973624	0.07776487	-4.1115768	3.93E-05	0.00020266	ELF2
177.6758546	1.082976293	0.15051299	7.1952348	6.24E-13	9.28E-12	MGARP
322.1305623	-0.30568937	0.09846446	-3.1045654	0.0019056	0.006885138	NDUFC1
3296.581748	-0.24184621	0.05347254	-4.5228112	6.10E-06	3.63E-05	NAA15
52.83764988	1.169738733	0.26599626	4.3975758	1.09E-05	6.29E-05	MGST2
381.0208413	0.857279696	0.10686385	8.0221671	1.04E-15	2.09E-14	CLGN
733.9520048	-0.27302477	0.0700088	-3.8998636	9.62E-05	0.000461338	ELMOD2
138.2770009	0.853070399	0.1690358	5.0466849	4.50E-07	3.25E-06	RNF150
23.80457258	1.618642435	0.41895312	3.8635407	0.0001118	0.000527818	LINC02432
724.0083361	-0.54093534	0.068057	-7.9482695	1.89E-15	3.66E-14	INPP4B
719.6587729	0.200942229	0.07283034	2.7590456	0.005797	0.018490571	USP38
4257.134606	-0.14363664	0.04091345	-3.5107439	0.0004469	0.001872934	SMARCA5
3839.217787	-0.1944242	0.04815993	-4.0370529	5.41E-05	0.000271415	ABCE1
1463.664044	-0.30801092	0.05374065	-5.7314325	9.96E-09	9.18E-08	OTUD4
222.3694096	-0.44502284	0.11925199	-3.7317856	0.0001901	0.000862367	LSM6
106.2135771	-0.40121588	0.16124352	-2.4882606	0.012837	0.037100683	SLC10A7

47.22421946	1.284193596	0.27722606	4.6322975	3.62E-06	2.26E-05	EDNRA
22.67092749	1.154992158	0.41060121	2.8129293	0.0049092	0.015939464	ENSG00000280219
1663.483556	-0.56936091	0.06278253	-9.0687795	1.20E-19	3.45E-18	LRBA
1867.954759	-0.36328861	0.05279591	-6.8809995	5.94E-12	7.94E-11	RPS3A
142.0926161	-0.93266966	0.15589736	-5.9825878	2.20E-09	2.21E-08	FHIP1A
322.1561453	-0.34673283	0.10283621	-3.3716997	0.0007471	0.002970809	GATB
606.9329964	0.207222159	0.07848832	2.6401657	0.0082866	0.025285684	FBXW7
894.0781826	-0.18596678	0.06738379	-2.7598147	0.0057834	0.018450484	ARFIP1
230.0424267	-0.6496916	0.1263923	-5.1402784	2.74E-07	2.05E-06	FHDC1
766.0114825	0.598620788	0.07958744	7.5215482	5.41E-14	9.09E-13	TMEM131L
10.62160794	2.282575301	0.6582232	3.4677831	0.0005248	0.002167125	TLR2
1773.560212	-0.14711504	0.05422594	-2.7130014	0.0066677	0.020941125	PLRG1
390.2465876	-0.38049726	0.09898883	-3.8438403	0.0001211	0.00056836	MAP9
60.70614697	-0.97707466	0.23507538	-4.1564313	3.23E-05	0.000169627	GUCY1B1
925.394481	-0.61640132	0.06863984	-8.9802268	2.70E-19	7.52E-18	PDGFC
20.10880315	2.490816092	0.53148104	4.6865568	2.78E-06	1.77E-05	GASK1B
431.5107527	-0.42486015	0.08678992	-4.8952708	9.82E-07	6.70E-06	ETFDH
836.8675535	-0.20971501	0.06884626	-3.0461349	0.002318	0.008193228	PPID
638.5354459	0.327093169	0.07556994	4.3283503	1.50E-05	8.37E-05	FNIP2
20.85896664	1.533459961	0.413601	3.7075828	0.0002092	0.000941964	FSTL5
295.4048418	-0.47673364	0.10239923	-4.6556371	3.23E-06	2.03E-05	NAF1
644.7645299	-0.25915855	0.07135826	-3.6317947	0.0002815	0.001227008	TMA16
1255.583705	-0.5505543	0.06507963	-8.4597017	2.68E-17	6.17E-16	MSMO1
1359.075821	0.765113262	0.07642811	10.010888	1.37E-23	5.36E-22	CPE
237.4900634	0.809869474	0.1286152	6.2968411	3.04E-10	3.41E-09	TLL1
295.8353635	2.024151241	0.13358377	15.152673	7.27E-52	1.13E-49	DDX60
312.780709	1.076072524	0.11154949	9.6465934	5.08E-22	1.77E-20	DDX60L
9.704846597	1.61338971	0.64366337	2.5065737	0.0121908	0.035462925	ENSG00000279384
305.0747314	0.373549234	0.10934029	3.4163915	0.0006346	0.002566914	CBR4
731.3401084	-0.34767108	0.07062605	-4.9227028	8.54E-07	5.88E-06	NEK1
1576.535948	-0.20361681	0.05904916	-3.448259	0.0005642	0.002311345	CLCN3
534.5089018	-0.36029193	0.08008181	-4.4990483	6.83E-06	4.04E-05	HPF1
40.61238453	0.783002176	0.29609275	2.644449	0.0081824	0.024994205	MFAP3L
1171.935641	-0.59933014	0.06176944	-9.7026972	2.94E-22	1.04E-20	GALNT7
6737.89388	-0.69730536	0.04991838	-13.968909	2.41E-44	2.82E-42	HMGB2
58.17627877	-0.75235455	0.23219141	-3.2402342	0.0011943	0.004522274	SAP30-DT
448.7572587	-0.55265217	0.0886832	-6.2317572	4.61E-10	5.07E-09	SAP30
255.9780117	-0.38034216	0.11067476	-3.4365754	0.0005891	0.002404875	FBXO8
591.7916404	-0.56874622	0.08253819	-6.8907037	5.55E-12	7.46E-11	CEP44
93.61345363	-0.76387174	0.18451455	-4.1398997	3.47E-05	0.000180961	WDR17
5090.882519	-0.15200498	0.04695658	-3.2371392	0.0012073	0.004567267	SPCS3
686.3980156	0.504768241	0.07299098	6.9154876	4.66E-12	6.33E-11	VEGFC
212.9099368	-0.3728795	0.12981208	-2.872456	0.0040729	0.013504015	TENM3-AS1
1388.505792	-0.53621614	0.07714043	-6.9511688	3.62E-12	4.96E-11	TENM3
1217.732895	-0.1416682	0.05634488	-2.5143046	0.0119267	0.034817181	DCTD
3546.548997	-0.18345139	0.04719305	-3.8872546	0.0001014	0.00048383	WWC2
914.9394695	0.184884151	0.06854367	2.6973192	0.00699	0.021823432	CDKN2AIP
1063.326062	-0.3155758	0.05919354	-5.3312536	9.75E-08	7.83E-07	TRAPPC11

7.741945894	4.726392231	1.29581958	3.6474153	0.0002649	0.00116413	ENSG00000287349
13.88868253	3.095258095	0.75746202	4.0863542	4.38E-05	0.000224327	ENSG00000248206
1893.864135	0.734511931	0.05805479	12.652047	1.09E-36	9.52E-35	CASP3
280.7787184	-0.26539316	0.10514657	-2.5240306	0.0116018	0.033988378	PRIMPOL
2774.445142	-0.11889368	0.04366528	-2.7228426	0.0064723	0.020395196	CENPU
1024.378797	0.344453079	0.06520794	5.2823796	1.28E-07	1.01E-06	ACSL1
726.5187178	0.495870463	0.07492823	6.6179389	3.64E-11	4.49E-10	SLC25A4
1241.480954	-0.57952156	0.0700366	-8.2745532	1.29E-16	2.78E-15	CFAP97
433.7675188	-0.64058182	0.08582416	-7.4638867	8.40E-14	1.38E-12	UFSP2
133.8202693	0.669983181	0.1556658	4.3039844	1.68E-05	9.27E-05	PLEKHG4B
25.50741017	1.034770698	0.34443578	3.0042486	0.0026624	0.0092616	ENSG00000260774
151.4272594	0.464201515	0.16223512	2.8612887	0.0042192	0.013922646	PDCD6
136.2377601	0.725373434	0.17268115	4.2006521	2.66E-05	0.000141625	SLC9A3-AS1
1212.911424	0.186464177	0.06410838	2.9085775	0.0036308	0.012193729	LPCAT1
703.9057379	-0.18410879	0.07467831	-2.4653583	0.0136876	0.039177208	NDUFS6
145.3298819	0.786261489	0.15318762	5.13267	2.86E-07	2.13E-06	ADAMTS16
2744.731512	-0.34623344	0.04935459	-7.0152227	2.30E-12	3.19E-11	ICE1
3401.581617	0.145872327	0.04924895	2.9619376	0.0030571	0.010483716	NSUN2
1338.614377	-0.21873959	0.05530934	-3.9548398	7.66E-05	0.000373899	TENT4A
470.2463613	0.867454101	0.10715058	8.0956549	5.70E-16	1.17E-14	SEMA5A
265.4046692	-0.3106224	0.11475746	-2.706773	0.0067941	0.02130344	SNHG18
10055.19015	-0.1749895	0.03705207	-4.7227999	2.33E-06	1.50E-05	CCT5
19.13515891	1.687083885	0.46284435	3.6450351	0.0002674	0.001174192	CMBL
4816.612435	-0.32428984	0.04704919	-6.8925699	5.48E-12	7.36E-11	MARCHF6
493.9455745	0.206116662	0.08688595	2.3722669	0.0176793	0.048661494	ANKRD33B
5789.372713	-0.14760051	0.05324299	-2.7722053	0.0055678	0.017837854	TRIO
582.1469345	0.27772379	0.08812275	3.1515562	0.001624	0.005974586	OTULINL
355.8679576	-0.46152507	0.09860073	-4.6807471	2.86E-06	1.82E-05	ANKH
686.201769	0.25915855	0.07255416	3.5719322	0.0003544	0.001515561	ZNF622
3587.42419	-0.12545985	0.04219063	-2.9736424	0.0029429	0.010138004	MYO10
111.440757	1.975959308	0.20173129	9.7950066	1.18E-22	4.36E-21	CDH10
56.94842153	1.472405805	0.25931061	5.6781549	1.36E-08	1.23E-07	PURPL
240.405202	2.313109545	0.1725028	13.409113	5.35E-41	5.57E-39	CDH6
2821.855841	-0.16568973	0.04749454	-3.4886056	0.0004855	0.002022434	DROSHA
1073.11966	0.162567687	0.06186496	2.6277832	0.0085943	0.026087513	C5orf22
1318.582288	-0.17838441	0.05467114	-3.2628622	0.0011029	0.004209827	MTMR12
4114.61254	-0.1645857	0.04585063	-3.5896061	0.0003312	0.001423427	ZFR
2466.356003	0.464360589	0.05964594	7.7852845	6.96E-15	1.27E-13	NPR3
1509.431297	0.551531865	0.05894945	9.3560135	8.28E-21	2.62E-19	ADAMTS12
2012.597186	-0.23763552	0.05058851	-4.697421	2.63E-06	1.68E-05	DNAJC21
107.5903069	-0.54998662	0.16945785	-3.2455659	0.0011722	0.004448737	SPEF2
1603.428621	0.470207245	0.05490053	8.564713	1.08E-17	2.58E-16	IL7R
1049.318029	-0.16084143	0.06742666	-2.3854278	0.0170593	0.047201385	LMBRD2
4037.577041	-0.20293703	0.03968877	-5.1132104	3.17E-07	2.34E-06	SKP2
620.7284349	-0.22072945	0.07777662	-2.8379925	0.0045398	0.014856523	NADK2
429.8876433	0.845123794	0.10463723	8.0767023	6.65E-16	1.36E-14	SLC1A3
498.7758889	-0.29371242	0.08324778	-3.5281712	0.0004184	0.00176576	NIPBL-DT
3306.881269	-0.39945644	0.05786397	-6.9033713	5.08E-12	6.88E-11	NIPBL

1340.170892	-0.24891205	0.06517405	-3.8191897	0.0001339	0.000623724	CPLANE1
3135.262149	-0.33301247	0.04850173	-6.8659911	6.60E-12	8.75E-11	NUP155
868.1099537	-0.22858167	0.06441225	-3.5487299	0.0003871	0.001642646	WDR70
398.8810756	0.256107141	0.09676731	2.6466288	0.0081299	0.024855514	GDNF
12.05038144	1.308375766	0.51855811	2.5231034	0.0116324	0.034066645	ENSG00000251257
1010.881395	-0.4273373	0.06684594	-6.3928682	1.63E-10	1.89E-09	LIFR
3115.783301	-0.49837483	0.04727626	-10.541756	5.55E-26	2.65E-24	OSMR
1336.076621	-0.28176364	0.06021614	-4.6792045	2.88E-06	1.83E-05	RICTOR
3121.886031	0.320770461	0.04316714	7.4308945	1.08E-13	1.75E-12	DAB2
517.7740962	-0.40361338	0.08045739	-5.0164862	5.26E-07	3.77E-06	TTC33
127.8861073	0.94400638	0.17608663	5.3610338	8.27E-08	6.71E-07	PTGER4
3911.936042	-0.11098217	0.04066525	-2.7291645	0.0063495	0.020054093	PRKAA1
7910.495398	-0.28612921	0.04120932	-6.9433127	3.83E-12	5.23E-11	RPL37
18.66593246	1.18456579	0.41710534	2.839968	0.0045118	0.014778737	CARD6
1203.690883	-0.51324648	0.06088852	-8.4292818	3.48E-17	7.91E-16	C5orf51
462.7018279	-0.20971661	0.08663799	-2.420608	0.0154946	0.043527928	ENSG00000287263
1488.506533	-0.24152559	0.05679869	-4.2523091	2.12E-05	0.000114407	HMGCS1
936.0560776	-0.26906427	0.07291308	-3.6902056	0.0002241	0.001000161	PAIP1
1794.000177	-0.49199936	0.05495294	-8.9531028	3.46E-19	9.54E-18	NNT
744.1161563	-0.26098369	0.06815521	-3.8292551	0.0001285	0.00060069	MRPS30
50.72004216	-0.83694055	0.25138184	-3.3293597	0.0008705	0.003413912	ENSG00000272335
394.1872703	0.257399108	0.09052998	2.843247	0.0044656	0.014663436	PARP8
625.3154611	0.219917251	0.07664245	2.8693921	0.0041126	0.013613041	PELO
525.9754782	1.630628341	0.10256842	15.897957	6.55E-57	1.24E-54	ITGA1
79.84911351	0.56247793	0.20612231	2.7288551	0.0063555	0.020065627	ITGA2
571.0614516	0.274526943	0.08214176	3.3421116	0.0008314	0.00327564	FST
586.4984236	-0.40735572	0.07574406	-5.3780549	7.53E-08	6.14E-07	NDUFS4
564.362042	0.315125764	0.08865786	3.5544032	0.0003788	0.001611544	SNX18
1588.030925	-0.37091163	0.05245221	-7.07142	1.53E-12	2.17E-11	GPX8
2665.189421	-0.46846183	0.04535965	-10.327721	5.28E-25	2.35E-23	MTREX
199.5154999	0.406257149	0.13222731	3.0724149	0.0021233	0.007586799	PLPP1
8692.015055	0.236884151	0.04571596	5.1816508	2.20E-07	1.67E-06	IL6ST
2859.879761	-0.33763398	0.04396505	-7.6795992	1.60E-14	2.82E-13	GPBP1
69.78838256	1.366344601	0.25197611	5.4225165	5.88E-08	4.86E-07	ACTBL2
10974.65839	-0.55937883	0.03826218	-14.619629	2.11E-48	2.93E-46	PLK2
983.1564009	-0.49607838	0.06361685	-7.797909	6.29E-15	1.15E-13	DEPDC1B
51.35289619	0.661866593	0.26243142	2.5220554	0.0116671	0.034151056	ELOVL7
426.9961953	-0.40170244	0.09157845	-4.3864297	1.15E-05	6.58E-05	ERCC8
1557.332315	0.272117956	0.0534054	5.0953267	3.48E-07	2.56E-06	ZSWIM6
2868.651671	-0.48409194	0.04769234	-10.150307	3.30E-24	1.36E-22	KIF2A
1454.354615	-0.48349974	0.06060782	-7.9775136	1.49E-15	2.93E-14	IPO11
1208.527912	-0.36753465	0.05628948	-6.5293664	6.60E-11	7.98E-10	CWC27
194.6296892	-0.71369278	0.12348681	-5.7795061	7.49E-09	7.03E-08	ADAMTS6
1948.266145	-0.20563847	0.05034336	-4.0847188	4.41E-05	0.000225647	CENPK
1138.90326	-0.32211772	0.06110116	-5.2718754	1.35E-07	1.06E-06	PPWD1
1181.050053	0.288851711	0.05983468	4.8274966	1.38E-06	9.24E-06	SGTB
1416.707936	-0.31393806	0.05992419	-5.2389207	1.62E-07	1.25E-06	NLN
9053.641093	-0.66211904	0.04240952	-15.612511	5.98E-55	1.01E-52	ERBIN

44.05146149	0.957577841	0.29018189	3.2999228	0.0009671	0.003753206	MAST4
727.4878852	0.659766487	0.0824204	8.0048927	1.20E-15	2.39E-14	PIK3R1
1459.024932	-0.19612946	0.05747989	-3.4121405	0.0006445	0.002602439	SLC30A5
6882.813936	-0.32463267	0.04070639	-7.9749798	1.52E-15	2.99E-14	CCNB1
908.6129405	-0.31406232	0.06528695	-4.8104916	1.51E-06	1.00E-05	CENPH
1108.665726	-0.25334276	0.06167081	-4.1079851	3.99E-05	0.000205531	RAD17
3005.606852	-0.25376449	0.05722836	-4.4342437	9.24E-06	5.37E-05	BDP1
1893.244208	-0.51027021	0.05169389	-9.8709974	5.56E-23	2.12E-21	MRPS27
7411.443909	-0.23954014	0.04334038	-5.5269511	3.26E-08	2.80E-07	TNPO1
107.4443001	0.96162161	0.18395512	5.2274793	1.72E-07	1.33E-06	TMEM171
2223.692892	-0.28051998	0.05227421	-5.3663167	8.04E-08	6.52E-07	FOXD1
9181.842353	-0.27708513	0.04029122	-6.8770603	6.11E-12	8.15E-11	BTF3
6135.678227	0.820801109	0.04731633	17.347102	2.07E-67	5.38E-65	ENC1
2914.319929	0.26359195	0.05513145	4.7811538	1.74E-06	1.15E-05	HEXB
1597.704768	-0.24045411	0.0526348	-4.5683484	4.92E-06	2.99E-05	GFM2
2513.456344	-0.27097981	0.0513977	-5.2722168	1.35E-07	1.06E-06	NSA2
344.7014536	0.649911562	0.11257875	5.7729508	7.79E-09	7.30E-08	GCNT4
2013.642358	0.238309675	0.04763573	5.0027506	5.65E-07	4.01E-06	CERT1
1472.928092	-0.42125068	0.05841787	-7.2109902	5.55E-13	8.31E-12	POLK
3.181379447	3.077689825	1.28654696	2.3922095	0.0167473	0.046545505	ANKDD1B
313.4101969	1.01532386	0.10999287	9.2308154	2.69E-20	8.15E-19	F2RL2
1889.125006	-0.43782185	0.04833923	-9.0572782	1.34E-19	3.81E-18	AP3B1
1675.365708	-0.3557336	0.05977265	-5.9514443	2.66E-09	2.64E-08	SCAMP1
980.4278601	-0.17515503	0.06906305	-2.5361614	0.0112075	0.032980576	JMY
628.0520391	0.466984318	0.09087189	5.1389305	2.76E-07	2.07E-06	HOMER1
1031.586795	-0.43608824	0.06181369	-7.0548808	1.73E-12	2.43E-11	MTX3
1760.589123	-0.12578324	0.05173632	-2.4312367	0.0150474	0.042476222	ZFYVE16
19.84154782	1.167183299	0.47181245	2.473829	0.0133674	0.038391168	ANKRD34B
1677.389619	-0.42185868	0.05378229	-7.8438219	4.37E-15	8.12E-14	DHFR
1007.009425	-0.39470909	0.06870668	-5.7448431	9.20E-09	8.53E-08	MSH3
40.832226	1.618419826	0.3274729	4.9421489	7.73E-07	5.37E-06	RASGRF2
9019.734824	-0.44616382	0.03797856	-11.747781	7.25E-32	5.06E-30	RPS23
2724.392896	0.208326756	0.04775026	4.3628406	1.28E-05	7.26E-05	TMEM167A
2954.328099	0.247462248	0.09931022	2.4918106	0.0127094	0.036762491	VCAN
1929.098698	-0.22014533	0.05585896	-3.9410923	8.11E-05	0.000393782	COX7C
1822.714284	-0.36970156	0.05377353	-6.8751593	6.19E-12	8.25E-11	RASA1
534.4862765	-0.47054209	0.07796729	-6.035122	1.59E-09	1.63E-08	CETN3
402.7629196	0.315862311	0.10126343	3.119214	0.0018133	0.006594316	POLR3G
98.63988907	0.544597331	0.17848799	3.0511707	0.0022795	0.008075101	NR2F1
591.4366789	-0.85419778	0.08011601	-10.662012	1.53E-26	7.68E-25	FAM172A
1055.972387	-0.17372809	0.05921808	-2.9337003	0.0033495	0.011356338	SLF1
37.66706075	3.747507316	0.52815968	7.0954059	1.29E-12	1.85E-11	MCTP1
3619.223071	-0.32006726	0.03947843	-8.1073961	5.17E-16	1.07E-14	TTC37
621.864795	0.279990029	0.08715207	3.2126607	0.0013151	0.004929911	ARSK
342.9677302	0.386006223	0.1049674	3.6773913	0.0002356	0.001046916	GLRX
55.77935791	0.835097147	0.26316991	3.1732243	0.0015076	0.005574413	HSPD1P11
61.90346195	3.096555858	0.37782479	8.1957456	2.49E-16	5.25E-15	LINC01554
5012.513138	0.268434077	0.03997283	6.7154126	1.88E-11	2.37E-10	ELL2

8861.353491	0.303184888	0.03825186	7.9260164	2.26E-15	4.34E-14	CAST
751.4426022	0.284162521	0.07596061	3.7409194	0.0001833	0.000835323	ERAP1
1446.271303	0.707973943	0.05879253	12.041904	2.14E-33	1.62E-31	ERAP2
2045.364796	0.265851128	0.05745576	4.6270576	3.71E-06	2.31E-05	LNPEP
1006.414141	0.220170413	0.0643895	3.4193527	0.0006277	0.002543279	RIOK2
1840.475697	-0.25222711	0.05350854	-4.7137729	2.43E-06	1.56E-05	RGMB
5715.855634	-0.31507753	0.04472783	-7.0443277	1.86E-12	2.61E-11	PAM
135.673354	-0.51248948	0.1605476	-3.1921341	0.0014123	0.005255544	GIN1
1800.293494	-0.42496013	0.05056605	-8.4040605	4.31E-17	9.70E-16	PPIP5K2
1117.574716	0.202697202	0.06653448	3.0464985	0.0023152	0.008186653	MACIR
322.295333	-0.50081962	0.09961609	-5.0274973	4.97E-07	3.58E-06	NUDT12
554.4185767	0.299870315	0.07883989	3.8035354	0.0001426	0.000661319	EFNA5
196.7942356	-0.64556445	0.13055284	-4.9448519	7.62E-07	5.31E-06	FBXL17
1122.325931	-0.85256135	0.07563332	-11.272298	1.80E-29	1.09E-27	FER
5122.779632	-0.38929546	0.04369296	-8.9097977	5.11E-19	1.39E-17	PJA2
3317.457744	0.124908356	0.0456965	2.7334339	0.0062678	0.019824758	MAN2A1
2248.646379	-0.20072731	0.05099381	-3.9363074	8.27E-05	0.000401153	WDR36
327.5156241	-0.54734062	0.10307617	-5.3100598	1.10E-07	8.72E-07	CAMK4
246.006985	0.446502967	0.12239958	3.6479124	0.0002644	0.001162469	EPB41L4A-AS1
1646.80146	-0.148432	0.06215716	-2.3880112	0.0169398	0.046960562	APC
402.0357238	0.314119784	0.09162778	3.4282157	0.0006076	0.002472622	SRP19
1205.13607	-0.27810004	0.06425835	-4.3278429	1.51E-05	8.39E-05	YTHDC2
243.278524	1.306060238	0.14057419	9.2908961	1.53E-20	4.73E-19	TRIM36
4754.238347	-0.1730574	0.05336225	-3.243068	0.0011825	0.004482055	TMED7
348.0695235	-0.36801998	0.09628703	-3.8221139	0.0001323	0.000616702	COMMD10
928.7996431	-0.42464804	0.07128604	-5.9569595	2.57E-09	2.57E-08	DMXL1
601.8508009	0.243460397	0.07729075	3.1499291	0.0016331	0.006004149	TNFAIP8
2543.656994	-0.46574285	0.04630875	-10.05734	8.53E-24	3.43E-22	HSD17B4
9357.312734	0.601529507	0.04746709	12.672558	8.39E-37	7.49E-35	LOX
511.4662593	0.429774367	0.08973735	4.7892474	1.67E-06	1.11E-05	PPIC
1069.977758	-0.31661698	0.05990284	-5.2855086	1.25E-07	9.89E-07	CEP120
1651.963633	-0.28042925	0.05823724	-4.8152908	1.47E-06	9.79E-06	CSNK1G3
740.4229862	0.294416605	0.07429986	3.962546	7.42E-05	0.000362947	GRAMD2B
41.22605393	1.326760895	0.30381857	4.3669513	1.26E-05	7.15E-05	ENSG00000279118
4010.208378	-0.76173813	0.03914422	-19.459787	2.41E-84	1.02E-81	LMNB1
569.4313511	0.553544787	0.08494527	6.5164876	7.20E-11	8.65E-10	MARCHF3
3284.910763	-0.17609257	0.04162018	-4.2309418	2.33E-05	0.000124905	PRRC1
1433.918931	0.183329125	0.06866025	2.670091	0.0075831	0.023385278	SLC12A2
6305.273715	0.770070504	0.09820315	7.8416068	4.45E-15	8.25E-14	FBN2
1089.225368	-0.38959649	0.06957059	-5.6000172	2.14E-08	1.89E-07	LYRM7
418.8067811	-0.33281646	0.08911627	-3.7346318	0.000188	0.000854232	RAPGEF6
2189.454875	0.508800348	0.04619053	11.015251	3.23E-28	1.85E-26	P4HA2
41.559702	1.405645068	0.30949156	4.541788	5.58E-06	3.35E-05	SLC22A4
303.4355745	0.849904353	0.1160823	7.3215673	2.45E-13	3.83E-12	IRF1
3243.873185	0.204345726	0.04217936	4.8446854	1.27E-06	8.52E-06	SEPTIN8
7397.387046	-0.20996221	0.04856617	-4.3232193	1.54E-05	8.54E-05	AFF4
5398.508481	-0.23575342	0.04281589	-5.5062131	3.67E-08	3.12E-07	VDAC1
99.61690816	-0.52987177	0.16859728	-3.1428252	0.0016733	0.006134924	TCF7

367.0432311	-0.23673434	0.09476116	-2.4982213	0.0124818	0.036200623	CDKN2AIPNL
2531.999654	-0.11496109	0.04717041	-2.4371441	0.0148038	0.041900671	SEC24A
4704.296429	-0.41256213	0.04005344	-10.300292	7.02E-25	3.11E-23	DDX46
686.4326499	-0.30546922	0.07431443	-4.1104966	3.95E-05	0.000203516	TXNDC15
4414.684192	-0.18954672	0.0439749	-4.3103387	1.63E-05	9.02E-05	MACROH2A1
8506.295686	0.8173701	0.11445104	7.1416574	9.22E-13	1.35E-11	TGFBI
3301.945008	-0.36371236	0.05038448	-7.2187382	5.25E-13	7.86E-12	SMAD5
1379.057139	0.960177172	0.06053208	15.862287	1.16E-56	2.14E-54	SPOCK1
2835.959769	-0.26330857	0.04523191	-5.8213006	5.84E-09	5.54E-08	HNRNPA0
2123.280904	0.74030331	0.06536328	11.325982	9.76E-30	5.98E-28	FAM13B
2250.403547	-0.12474261	0.04615345	-2.7027797	0.0068762	0.021510641	BRD8
5085.498424	-0.32129208	0.04655853	-6.9008208	5.17E-12	7.00E-11	KIF20A
1829.025134	-0.20014747	0.04834305	-4.1401502	3.47E-05	0.000180832	CDC23
617.3867667	-0.48007858	0.08179097	-5.869579	4.37E-09	4.20E-08	CDC25C
1736.389993	0.261606294	0.05280999	4.9537276	7.28E-07	5.08E-06	FAM53C
2587.426047	-0.32791161	0.04376634	-7.4923247	6.77E-14	1.12E-12	KDM3B
1176.880331	-2.12661437	0.27678069	-7.6833913	1.55E-14	2.74E-13	EGR1
7347.404177	-0.3283746	0.03890591	-8.4402238	3.17E-17	7.23E-16	HSPA9
19.72319783	1.011009237	0.41614298	2.4294757	0.0151207	0.042638012	LRRTM2
444.096858	0.347846947	0.09319822	3.7323347	0.0001897	0.000860713	SIL1
482.8298203	0.51482196	0.10119882	5.0872328	3.63E-07	2.66E-06	SNHG4
2605.58281	-0.25907764	0.04852603	-5.3389414	9.35E-08	7.53E-07	PAIP2
471.7278832	1.320496338	0.09357322	14.111904	3.21E-45	3.80E-43	STING1
28.47990969	4.398132435	0.78654129	5.5917375	2.25E-08	1.97E-07	ENSG00000286403
2007.801059	-0.20177388	0.05484107	-3.6792477	0.0002339	0.001040389	UBE2D2
828.0948071	-0.47470916	0.06990303	-6.7909666	1.11E-11	1.44E-10	CXXC5
58.40192733	0.667486606	0.24822095	2.6890825	0.0071649	0.02227335	MALINC1
1635.164938	-0.1746361	0.05324081	-3.2801175	0.0010376	0.003988624	PURA
2814.522606	0.519646896	0.06538072	7.9480147	1.90E-15	3.66E-14	HBEGF
711.6442783	0.202724442	0.0717958	2.8236253	0.0047484	0.015483672	SRA1
105.1456427	-0.51208713	0.18696806	-2.7389017	0.0061645	0.019526471	TMCO6
2556.261645	-0.17043076	0.04340834	-3.9262212	8.63E-05	0.000417412	IK
2457.713551	0.188365831	0.04800073	3.9242288	8.70E-05	0.000420531	HARS1
39.34039229	-0.89383389	0.28389954	-3.1484161	0.0016416	0.006034041	PCDHB5
29.27694938	-0.87952223	0.33548182	-2.6216688	0.00875	0.026495422	PCDHB13
3399.913286	-0.19732659	0.04533128	-4.3529899	1.34E-05	7.57E-05	TAF7
4.417556254	-2.89323882	1.00784319	-2.8707232	0.0040953	0.013565325	PCDHGA5
11.24898561	-1.19616294	0.5004004	-2.3904117	0.0168295	0.046706824	PCDHGA8
134.6110268	-0.3706565	0.14772326	-2.5091276	0.012103	0.035231122	PCDHGA11
1588.772442	0.321067103	0.06320801	5.0795321	3.78E-07	2.77E-06	PCDHGC3
6721.751742	-0.22138555	0.03470006	-6.3799762	1.77E-10	2.05E-09	DIAPH1
1450.33019	-0.15436412	0.0563277	-2.7404657	0.0061352	0.019444403	HDAC3
31.01591131	1.626892006	0.37592066	4.3277536	1.51E-05	8.39E-05	PCDH1
1228.071652	-0.20590174	0.05931835	-3.4711304	0.0005183	0.002142832	GNPDA1
223.5373714	0.91506838	0.12197596	7.5020386	6.28E-14	1.05E-12	SPRY4
889.9306695	0.806796253	0.07057201	11.432241	2.89E-30	1.83E-28	FGF1
549.2702864	0.663617068	0.08726298	7.6047949	2.85E-14	4.92E-13	ARHGAP26
3437.785249	-0.44014475	0.04583482	-9.6028468	7.78E-22	2.65E-20	NR3C1

64.37851569	1.205470511	0.24704421	4.8795741	1.06E-06	7.22E-06	KCTD16
166.3828148	0.981353061	0.14865821	6.6014049	4.07E-11	4.99E-10	SH3RF2
4715.430167	-0.53129684	0.04039408	-13.152839	1.64E-39	1.60E-37	LARS1
1064.374139	-0.35392156	0.06049155	-5.8507605	4.89E-09	4.69E-08	RBM27
2661.840144	-0.43825722	0.05003541	-8.7589408	1.97E-18	5.01E-17	TCERG1
2064.388448	0.317696445	0.05037508	6.3066196	2.85E-10	3.22E-09	DPYSL3
9.192782793	4.985230679	1.27603538	3.9068123	9.35E-05	0.000449266	C5orf46
1181.695238	-0.27134147	0.05687036	-4.7712282	1.83E-06	1.20E-05	FBXO38
364.7483164	0.802689984	0.10416892	7.705657	1.30E-14	2.33E-13	ADRB2
134.077384	0.496464303	0.16174921	3.0693461	0.0021453	0.007655737	SH3TC2
376.6760693	0.294364159	0.10585788	2.7807486	0.0054234	0.017416886	ABLIM3
691.4916367	-0.27750494	0.07584529	-3.6588288	0.0002534	0.001117736	GRPEL2
304.7161511	0.479590392	0.11058231	4.336954	1.44E-05	8.08E-05	PCYOX1L
57.14472564	1.607549396	0.24993938	6.4317571	1.26E-10	1.48E-09	CARMN
3354.933673	-0.19723517	0.04768614	-4.1361111	3.53E-05	0.000183808	CSNK1A1
1002.158807	-0.21904464	0.07700558	-2.8445294	0.0044477	0.014607299	SLC26A2
8.518801861	1.666911314	0.68763743	2.4241137	0.0153458	0.043167821	CSF1R
1275.06274	-0.52971934	0.06307423	-8.3983477	4.53E-17	1.02E-15	PDGFRB
14.56392932	3.390350794	0.93859404	3.6121589	0.0003037	0.001314895	CD74
6994.971017	-0.28783314	0.04270483	-6.7400607	1.58E-11	2.01E-10	RPS14
392.3750409	1.046884737	0.10279934	10.183769	2.34E-24	9.84E-23	SYNPO
2849.937166	-0.25353222	0.04512872	-5.6179792	1.93E-08	1.71E-07	DCTN4
149.9317462	-0.44484796	0.14706504	-3.0248382	0.0024877	0.00872538	SMIM3
8.596621891	3.754058354	1.14919565	3.2666834	0.0010882	0.004162556	IRGM
398.4335881	-0.48346472	0.09750533	-4.9583415	7.11E-07	4.97E-06	ZNF300
551.8264457	0.639407417	0.08301506	7.7023061	1.34E-14	2.39E-13	GPX3
2349.638648	0.234259798	0.04709857	4.9738197	6.56E-07	4.61E-06	TNIP1
3068.489287	0.252266634	0.04209565	5.9927006	2.06E-09	2.09E-08	ANXA6
59.9292189	0.930158932	0.24938167	3.7298609	0.0001916	0.000868301	CCDC69
786.8196673	0.23343727	0.06923385	3.3717218	0.000747	0.002970809	GM2A
23035.74549	0.547655473	0.0441374	12.407967	2.37E-35	1.95E-33	SPARC
408.5487422	0.287979447	0.10241775	2.8118119	0.0049263	0.015988967	ATOX1
7613.267478	-0.13423285	0.03885065	-3.4550991	0.0005501	0.002259347	G3BP1
790.5259759	-0.38902682	0.06783554	-5.7348526	9.76E-09	9.00E-08	FAM114A2
1674.115865	0.197837391	0.0574602	3.4430336	0.0005752	0.002354805	GALNT10
7348.509648	-0.37527988	0.04110219	-9.1304105	6.82E-20	1.99E-18	LARP1
1716.301026	-0.19716655	0.0537149	-3.670612	0.000242	0.001072614	CNOT8
1389.455774	-0.27242586	0.05430861	-5.0162555	5.27E-07	3.77E-06	GEMIN5
773.5294554	-0.40160246	0.06966451	-5.7648068	8.18E-09	7.64E-08	MRPL22
1007.429298	0.285607929	0.06538866	4.3678513	1.25E-05	7.12E-05	CYFIP2
2921.551091	1.006537475	0.0531242	18.946874	4.69E-80	1.70E-77	ADAM19
132.1609368	0.813907814	0.16129966	5.0459362	4.51E-07	3.26E-06	NIPAL4
498.2111059	-0.24908769	0.07918441	-3.1456659	0.0016571	0.006082066	THG1L
433.0810671	-0.23559175	0.09838788	-2.3945201	0.0166421	0.046290257	LSM11
34.02756165	1.137632549	0.33252991	3.4211435	0.0006236	0.002529538	EBF1
2724.658473	0.121300487	0.04397253	2.7585514	0.0058058	0.018508373	RNF145
1181.344302	0.15085863	0.06340562	2.3792627	0.0173473	0.047906887	UBLCP1
209.0911116	0.500690647	0.12856707	3.8943926	9.84E-05	0.000470836	CCNJL

219.2240791	-0.41371385	0.12481719	-3.3145584	0.0009179	0.003578107	ZBED8
2833.635659	-0.15638076	0.05140225	-3.0422942	0.0023478	0.008283351	PTTG1
3.582426176	3.105442962	1.26734639	2.4503506	0.0142717	0.040632805	MIR3142HG
4712.669352	-0.16131966	0.04132836	-3.9033643	9.49E-05	0.000455089	CCNG1
1470.793568	-0.2062361	0.05673275	-3.6352215	0.0002777	0.001212028	MAT2B
1971.420794	-1.31092752	0.10490117	-12.496787	7.77E-36	6.63E-34	TENM2
1066.029299	0.292968101	0.06047252	4.8446489	1.27E-06	8.52E-06	WWC1
3623.77647	-0.17798805	0.04390182	-4.0542297	5.03E-05	0.000254056	RARS1
4431.596671	-0.3273222	0.04604729	-7.1083925	1.17E-12	1.70E-11	PANK3
17.03276519	-1.37605732	0.43405326	-3.1702499	0.0015231	0.005628206	SLIT3
734.8272607	-0.7665667	0.07181828	-10.673699	1.35E-26	6.83E-25	INSYN2B
276.1386336	-0.2877704	0.11021318	-2.6110344	0.0090269	0.027238849	RANBP17
13676.91577	-0.33804038	0.0350353	-9.6485645	4.98E-22	1.74E-20	NPM1
19.28352323	1.573380018	0.50926628	3.0895036	0.0020049	0.007203601	FGF18
1424.834716	0.340791166	0.05350094	6.3698164	1.89E-10	2.18E-09	STK10
352.6295461	-0.56590645	0.10270728	-5.5098961	3.59E-08	3.06E-07	NEURL1B
7.341184138	2.32799125	0.81999272	2.8390389	0.004525	0.014813464	ENSG00000253736
2744.930429	0.12550291	0.05079335	2.470853	0.0134791	0.038667419	DUSP1
2037.686006	-0.24002874	0.0504257	-4.7600475	1.94E-06	1.26E-05	ERGIC1
1882.561256	0.396213403	0.05847571	6.7756924	1.24E-11	1.60E-10	ATP6V0E1
243.496351	-0.41401105	0.11246617	-3.6812052	0.0002321	0.001032961	BNIP1
10587.43831	0.646268793	0.04060162	15.917315	4.81E-57	9.18E-55	STC2
903.1144515	0.59085887	0.07915903	7.4642002	8.38E-14	1.37E-12	CPEB4
2646.644095	-0.15149517	0.0503283	-3.0101386	0.0026113	0.009102073	SFXN1
214.6664505	-0.31298339	0.12094436	-2.5878296	0.0096583	0.02894819	SIMC1
1000.007608	-0.26454477	0.06558889	-4.0333774	5.50E-05	0.000275379	ARL10
866.0781723	0.296753594	0.06763812	4.3873721	1.15E-05	6.56E-05	CLTB
3061.771894	-0.13731189	0.04782507	-2.8711282	0.0040901	0.013550538	FAF2
767.3903569	-0.29304928	0.06820875	-4.2963587	1.74E-05	9.56E-05	UIMC1
184.9662886	-0.60888667	0.13042515	-4.668476	3.03E-06	1.92E-05	FGFR4
2951.303326	-0.32454123	0.05200011	-6.2411642	4.34E-10	4.80E-09	NSD1
186.3739054	-0.36687996	0.12814747	-2.8629511	0.0041972	0.013865594	MXD3
148.0883185	0.359303118	0.15093541	2.3805092	0.0172887	0.047783062	ENSG00000279821
1824.517559	0.252993485	0.05532179	4.5731251	4.81E-06	2.93E-05	PDLIM7
159.2168538	0.598802893	0.15069344	3.9736493	7.08E-05	0.000348389	DOK3
3336.077545	0.114291586	0.04823058	2.3696914	0.0178029	0.04893979	TMED9
263.8033692	0.358609456	0.1154613	3.1058844	0.0018971	0.006861629	B4GALT7
795.1050878	-0.31642131	0.06799033	-4.6539166	3.26E-06	2.05E-05	NHP2
5838.758435	-0.19034701	0.04203442	-4.5283606	5.94E-06	3.55E-05	HNRNPAB
474.3425838	0.466979924	0.08908099	5.2421949	1.59E-07	1.23E-06	CLK4
251.935532	-0.31018734	0.11366291	-2.7290111	0.0063525	0.02005978	ZNF354B
417.4050238	-0.39406478	0.0889777	-4.4288039	9.48E-06	5.50E-05	ZNF354C
654.7594418	-0.21260759	0.07371344	-2.8842445	0.0039235	0.013073455	RUFY1
11301.5644	-0.2547288	0.04127602	-6.1713514	6.77E-10	7.29E-09	HNRNPH1
2781.205964	0.364822745	0.04380192	8.3289219	8.16E-17	1.80E-15	SQSTM1
558.0643379	0.331768138	0.08983435	3.6931101	0.0002215	0.000991347	MRNIP
816.0345311	-0.19737562	0.07019737	-2.8117237	0.0049277	0.015990367	RNF130
1561.133595	-0.20325335	0.05637591	-3.6053225	0.0003118	0.001346313	MAPK9

500.0253556	0.341003984	0.0954324	3.5732518	0.0003526	0.001509426	GFPT2
1595.077427	-0.25818616	0.05114645	-5.0479777	4.47E-07	3.23E-06	CNOT6
289.0327696	-0.41361826	0.10201229	-4.0545923	5.02E-05	0.000253811	FLT4
2013.220727	0.299471812	0.04991565	5.999557	1.98E-09	2.01E-08	MGAT1
680.7929405	-0.29861314	0.07533003	-3.9640653	7.37E-05	0.000360948	ZFP62
14612.26849	-0.31521337	0.03568226	-8.8338968	1.01E-18	2.68E-17	RACK1
117.9511807	0.865289333	0.19190072	4.5090467	6.51E-06	3.87E-05	CTC-338M12.4
99.52670748	-0.46387465	0.17714582	-2.6186035	0.0088291	0.026692838	TRIM52-AS1
5.732840783	4.209934292	1.32609778	3.1746786	0.0015	0.005548913	IRF4
1142.494015	-0.2311175	0.05973924	-3.8687722	0.0001094	0.000517606	EXOC2
660.0950195	0.888922091	0.08108805	10.96243	5.79E-28	3.23E-26	FOXC1
566.2564307	-0.21832802	0.07784468	-2.8046619	0.0050369	0.016308391	GMDS
2259.869028	0.124285965	0.0478221	2.5989233	0.0093517	0.028121302	SERPINB6
1704.336725	0.235649263	0.0534666	4.407411	1.05E-05	6.03E-05	RIPK1
802.0095104	0.600209241	0.07573919	7.9246856	2.29E-15	4.38E-14	TUBB2A
48.33172007	1.300274318	0.30551177	4.2560532	2.08E-05	0.00011293	ENSG00000228793
169.8908599	0.731510922	0.1469436	4.9781746	6.42E-07	4.52E-06	ENSG00000260604
4087.051718	-0.1188345	0.04255975	-2.7921807	0.0052354	0.016872458	PRPF4B
1700.553045	-0.37354178	0.05513956	-6.7744784	1.25E-11	1.61E-10	ECI2
16.19766843	1.251810263	0.48370211	2.5879777	0.0096541	0.028944839	ECI2-DT
1171.440669	-0.19864977	0.06341222	-3.1326734	0.0017322	0.006333752	CDYL
37.30690108	0.888687276	0.33598402	2.6450284	0.0081684	0.024960213	ENSG00000272142
476.3280004	-0.37917616	0.08656784	-4.3801044	1.19E-05	6.76E-05	LYRM4
159.1489495	-0.40482174	0.13660755	-2.963392	0.0030427	0.010440478	FARS2
665.0902278	-0.40790778	0.07026481	-5.8052927	6.43E-09	6.07E-08	RREB1
5632.284746	-0.13912511	0.04453269	-3.124112	0.0017834	0.006497752	SSR1
1411.403135	-0.17060139	0.05295167	-3.221832	0.0012737	0.004794437	SNRNP48
507.4277466	-0.42210397	0.08054132	-5.2408376	1.60E-07	1.24E-06	BLOC1S5
537.0161019	0.248616597	0.08652012	2.873512	0.0040594	0.013469225	SLC35B3
1147.813585	0.171403183	0.06851163	2.5018115	0.012356	0.035895473	TFAP2A
7.972960268	3.125286045	1.00789755	3.1007973	0.00193	0.006967565	LINC02522
47.91127822	0.593824023	0.24491484	2.4246143	0.0153247	0.043122295	GCNT2
16.09597791	1.418090995	0.48486095	2.9247375	0.0034475	0.011647851	MAK
987.4403501	-0.60621806	0.06285045	-9.6454048	5.14E-22	1.79E-20	ELOVL2
1652.358709	0.380079762	0.05520404	6.8849985	5.78E-12	7.75E-11	SMIM13
1771.323662	0.545041735	0.0547695	9.9515552	2.48E-23	9.55E-22	NEDD9
561.1081107	-0.61436571	0.08044088	-7.6374811	2.22E-14	3.86E-13	TMEM170B
1260.899699	1.759616184	0.07464967	23.571653	7.53E-123	7.70E-120	EDN1
199.1553675	0.873073487	0.13725447	6.3609841	2.00E-10	2.30E-09	GFOD1
276.7272657	-0.31179338	0.10535485	-2.9594591	0.0030818	0.010560086	SIRT5
228.7658094	0.352790213	0.1220472	2.8906046	0.003845	0.012836402	CD83
1014.372842	0.19799015	0.06698551	2.955716	0.0031194	0.010665965	JARID2
378.6734797	-0.26269938	0.08996078	-2.9201546	0.0034986	0.011799896	DTNBP1
300.806107	0.616503634	0.11289798	5.4607143	4.74E-08	3.98E-07	MYLIP
76.40886399	1.057748102	0.21926863	4.8239829	1.41E-06	9.39E-06	GMPR
81.60640438	0.86305165	0.21015298	4.106778	4.01E-05	0.000206425	ENSG00000272341
2247.572003	-0.39777893	0.0528302	-7.5293852	5.10E-14	8.57E-13	CAP2
1365.585465	-0.3801176	0.05975733	-6.3610201	2.00E-10	2.30E-09	FAM8A1

4779.906313	-0.2469677	0.04226099	-5.8438695	5.10E-09	4.87E-08	NUP153
79.96894871	0.57385039	0.19503213	2.9423377	0.0032574	0.011076843	NUP153-AS1
1927.881126	-0.36301298	0.05381933	-6.7450298	1.53E-11	1.95E-10	KIF13A
1212.426673	-0.2294289	0.05692166	-4.0306081	5.56E-05	0.000278404	TPMT
25689.75553	-0.49021163	0.03644719	-13.449915	3.08E-41	3.23E-39	DEK
1151.832347	-0.17669807	0.05685983	-3.1076085	0.0018861	0.006827412	RNF144B
96.58333595	0.889653708	0.20734124	4.2907706	1.78E-05	9.78E-05	ID4
989.4553624	0.414126656	0.0690424	5.9981499	2.00E-09	2.02E-08	MBOAT1
1549.690032	0.179245505	0.05195455	3.4500443	0.0005605	0.002298279	E2F3
535.8246977	-0.46014319	0.07867199	-5.8488819	4.95E-09	4.73E-08	CDKAL1
903.6382469	1.214829405	0.08044059	15.102194	1.57E-51	2.37E-49	SOX4
422.8294666	-0.50036471	0.0899443	-5.563051	2.65E-08	2.31E-07	ALDH5A1
1199.85187	-0.25844812	0.05707147	-4.5284994	5.94E-06	3.55E-05	TDP2
255.6277959	-0.63483796	0.11088094	-5.7254021	1.03E-08	9.48E-08	ACOT13
6132.823541	-0.22635468	0.04040321	-5.6023932	2.11E-08	1.87E-07	C6orf62
5.72215406	3.494213742	1.03069362	3.3901575	0.0006985	0.00279635	CMAHP
686.7760187	0.188446845	0.07836017	2.4048806	0.0161778	0.045171978	CARMIL1
292.8246728	0.610563359	0.10737319	5.6863668	1.30E-08	1.18E-07	TRIM38
24.64361181	1.403555457	0.43474963	3.2284224	0.0012448	0.004695489	H3C2
1020.678234	1.31985166	0.08133566	16.22722	3.24E-59	6.55E-57	H1-2
230.942309	-0.71637137	0.11576458	-6.1881742	6.09E-10	6.59E-09	HFE
331.1746514	1.654984741	0.12112868	13.66303	1.69E-42	1.88E-40	H2BC4
850.4927437	1.237858521	0.084526	14.644706	1.46E-48	2.04E-46	H2AC6
661.488909	1.587110605	0.09192509	17.26526	8.59E-67	2.17E-64	H2BC5
48.63731262	1.347086838	0.32237212	4.1786704	2.93E-05	0.000154893	H2BC6
5.844604621	2.868852655	1.04367602	2.7487962	0.0059815	0.019022958	H4C4
69.3636406	1.587720585	0.25900898	6.1299828	8.79E-10	9.29E-09	ENSG00000282988
15.72564983	1.401564849	0.53261481	2.6314793	0.0085014	0.025842757	H2BC7
8.573554408	2.930749791	0.87843261	3.3363399	0.0008489	0.003335348	ENSG00000217275
23.19126661	1.132222348	0.39052696	2.8992169	0.003741	0.01252489	H4C5
66.38140553	1.837111726	0.26883977	6.8334821	8.29E-12	1.09E-10	H2BC8
28.70696377	1.39559268	0.39032055	3.575504	0.0003496	0.001497596	H2AC8
169.3935747	1.006844869	0.15052816	6.6887477	2.25E-11	2.82E-10	H3C6
37.94118114	1.212959362	0.32776291	3.7007219	0.000215	0.000965055	H2BC9
12.68054809	2.852904899	0.72613962	3.9288655	8.53E-05	0.000413079	H3C8
513.8144801	2.981921276	0.13095909	22.769869	9.12E-115	8.35E-112	H4C8
150.0831612	-0.49060474	0.14442111	-3.3970431	0.0006812	0.002734629	BTN3A2
184.9867165	0.394581194	0.1427252	2.7646218	0.0056989	0.01821757	BTN2A3P
1893.174682	-0.3133535	0.058066	-5.3965052	6.80E-08	5.56E-07	HMGN4
28.40862516	1.035972268	0.34030878	3.0442126	0.0023329	0.008240742	LINC00240
89.41858004	1.650627292	0.22744613	7.2572229	3.95E-13	6.02E-12	ENSG00000272468
394.2841121	1.389963943	0.10564505	13.156924	1.55E-39	1.53E-37	H2BC11
83.66068186	1.213222398	0.23325531	5.2012638	1.98E-07	1.52E-06	H2AC11
1362.078646	1.38304631	0.06387785	21.651423	5.89E-104	4.10E-101	H2BC12
144.2475727	-1.12938611	0.14591193	-7.7401901	9.93E-15	1.79E-13	ZNF204P
250.5910276	-0.34465638	0.11096795	-3.1059093	0.0018969	0.006861629	ZNF391
545.9495954	-0.31535303	0.09132894	-3.4529367	0.0005545	0.002276461	ZNF184
4.05821316	2.768877823	1.09121811	2.5374192	0.0111673	0.032904028	H2BC13

49.66640169	1.780648117	0.34302492	5.1910168	2.09E-07	1.60E-06	H2AC13
154.0427381	1.695218263	0.18492376	9.1671198	4.86E-20	1.44E-18	H3C10
115.1241575	1.771646444	0.21365592	8.292054	1.11E-16	2.41E-15	H2BC15
13.69280045	1.483384679	0.55276568	2.6835687	0.0072841	0.022591494	OR2B6
1940.635571	-0.40028286	0.05533201	-7.234201	4.68E-13	7.08E-12	ZKSCAN8
321.3533017	-0.3536141	0.10104119	-3.4997023	0.0004658	0.001946618	ZSCAN26
647.2052533	-0.26500566	0.07276693	-3.6418418	0.0002707	0.001185737	ZSCAN12
108.0382082	-0.58270125	0.17459275	-3.3374882	0.0008454	0.003323098	ZSCAN23
7.32886967	3.510549361	1.16100895	3.0237057	0.002497	0.008756341	ZFP57
63.67832822	0.918172076	0.23677931	3.8777547	0.0001054	0.000500776	HLA-G
1075.851651	0.685051899	0.07250957	9.4477444	3.46E-21	1.13E-19	HLA-A
181.5394985	-0.42669725	0.12932498	-3.2994187	0.0009689	0.003759114	POLR1H
1030.117499	-0.24762035	0.06120242	-4.0459241	5.21E-05	0.000262244	PPP1R11
1374.935075	0.529126951	0.05942124	8.9046774	5.35E-19	1.45E-17	TRIM26
187.0976615	0.495858986	0.13746583	3.6071435	0.0003096	0.001337235	HLA-L
3432.390476	0.748612656	0.05079266	14.7386	3.64E-49	5.15E-47	HLA-E
3.280974827	3.809120452	1.44327564	2.639219	0.0083097	0.02534307	LINC02569
1295.218635	-0.13996138	0.05799451	-2.4133557	0.0158064	0.044270103	GNL1
2323.220432	0.363943707	0.0487955	7.4585507	8.75E-14	1.43E-12	PPP1R10
2687.73155	0.143654093	0.04676138	3.0720671	0.0021258	0.007592523	PPP1R18
362.5597358	-0.54456459	0.09873974	-5.5151508	3.48E-08	2.98E-07	NRM
39739.98509	-0.27596221	0.03091133	-8.9275432	4.36E-19	1.19E-17	TUBB
1863.05774	-0.27344982	0.06198813	-4.4113256	1.03E-05	5.93E-05	FLOT1
3712.149056	0.197906527	0.05025997	3.937657	8.23E-05	0.000399015	IER3
878.4875565	0.169957075	0.06718212	2.5297961	0.0114129	0.03353101	DDR1
33.98124706	1.050762721	0.30649607	3.4283074	0.0006074	0.002472365	PSORS1C1
33.93507733	0.801966184	0.3155291	2.5416552	0.0110329	0.032546572	PSORS1C3
1278.022381	0.752164302	0.082102	9.161339	5.13E-20	1.52E-18	HLA-C
1935.801678	1.330148962	0.07031006	18.918331	8.06E-80	2.86E-77	HLA-B
1019.814559	0.263731696	0.06347723	4.1547451	3.26E-05	0.000170728	MICA
14.46005106	1.456473412	0.54276049	2.6834551	0.0072866	0.02259514	HCP5
1455.513689	0.511995682	0.06212131	8.2418689	1.70E-16	3.61E-15	MICB
2553.730787	0.277441279	0.10979085	2.5269981	0.0115042	0.03375941	DDX39B
8946.561635	-0.32889544	0.03540884	-9.288513	1.56E-20	4.82E-19	PRRC2A
4146.58212	-0.39823551	0.04467895	-8.91327	4.95E-19	1.35E-17	BAG6
219.2079448	0.315458196	0.13308797	2.3702984	0.0177737	0.048882684	CSNK2B
100.6183143	0.638369787	0.1962584	3.2527004	0.0011431	0.004349934	LY6G5B
451.6399927	-0.24462255	0.08609655	-2.8412585	0.0044936	0.014741272	DDAH2
5439.223388	-0.21808158	0.04391007	-4.9665502	6.82E-07	4.78E-06	CLIC1
1586.904369	-0.22649916	0.05086898	-4.4525985	8.48E-06	4.96E-05	VARS1
566.5962277	-0.40496512	0.08430716	-4.8034485	1.56E-06	1.04E-05	LSM2
1662.657105	0.918282666	0.05850396	15.696077	1.61E-55	2.85E-53	NEU1
1103.555989	-0.38924555	0.05895006	-6.6029715	4.03E-11	4.95E-10	EHMT2
67.25377255	-1.02977778	0.20746942	-4.9635159	6.92E-07	4.84E-06	ZBTB12
1441.35335	-0.20753514	0.05693736	-3.6449731	0.0002674	0.001174192	NELFE
61.40162874	0.653631492	0.23374464	2.7963486	0.0051684	0.016684206	TNXB
41.83801801	0.711734434	0.2853329	2.4944001	0.012617	0.036531806	EGFL8
591.9238498	-0.3274216	0.07508236	-4.3608329	1.30E-05	7.32E-05	PBX2

105.93531	0.815101593	0.17483322	4.6621666	3.13E-06	1.98E-05	GPSM3
676.5783512	0.350914793	0.07074708	4.9601312	7.04E-07	4.93E-06	TAP2
171.8425068	0.77841329	0.13474232	5.7770514	7.60E-09	7.13E-08	PSMB8
88.54789103	1.02744485	0.19508617	5.2666206	1.39E-07	1.09E-06	PSMB9
520.1652205	1.161172804	0.08819682	13.1657	1.38E-39	1.37E-37	TAP1
649.420276	0.242175234	0.07604939	3.1844467	0.0014503	0.005384478	ENSG00000289047
11.40602255	1.540799509	0.64097304	2.4038445	0.0162237	0.045263996	HLA-DOA
56.22351133	0.699099071	0.24965612	2.800248	0.0051063	0.016508491	HLA-DPA1
58.31508504	1.504150707	0.26885333	5.5946889	2.21E-08	1.94E-07	COL11A2
919.7629722	-0.30087567	0.06612994	-4.5497648	5.37E-06	3.24E-05	VPS52
17938.36919	-0.31251831	0.04269345	-7.320053	2.48E-13	3.87E-12	RPS18
1492.146432	-0.16977481	0.05405125	-3.1409967	0.0016837	0.006168153	WDR46
1026.684813	-0.1761605	0.07357711	-2.3942297	0.0166553	0.04631883	PFDN6
2504.478397	0.286120195	0.04971035	5.7557471	8.63E-09	8.03E-08	TAPBP
2623.553904	-0.23979624	0.04346585	-5.5168882	3.45E-08	2.95E-07	KIFC1
617.7911364	-0.42058545	0.09157351	-4.5928726	4.37E-06	2.69E-05	PHF1
1589.971536	-0.19848812	0.05559867	-3.5700158	0.000357	0.001525941	CUTA
371.1976366	-0.44130683	0.09992936	-4.4161879	1.00E-05	5.80E-05	SYNGAP1
386.1025346	0.343371706	0.09478394	3.6226783	0.0002916	0.00126696	BAK1
50.1773641	1.252114028	0.26619069	4.7038235	2.55E-06	1.64E-05	IP6K3
6302.328297	-0.53796964	0.05242909	-10.260901	1.06E-24	4.57E-23	HMGA1
44.03747272	0.964258891	0.29465014	3.2725554	0.0010658	0.004081537	ENSG00000225339
799.2036501	-0.81811967	0.07470013	-10.952052	6.50E-28	3.60E-26	NUDT3
2107.827364	-0.1705408	0.04891557	-3.4864318	0.0004895	0.002038454	SNRPC
1551.200135	-0.49321284	0.05698985	-8.6543982	4.96E-18	1.22E-16	UHRF1BP1
573.7269343	-0.42333694	0.07998344	-5.2928071	1.20E-07	9.53E-07	ANKS1A
129.8227528	0.8021509	0.177019	4.5314394	5.86E-06	3.50E-05	SCUBE3
386.639018	0.388702845	0.09618529	4.0411878	5.32E-05	0.000266903	ZNF76
1077.585136	-0.17351901	0.0633746	-2.7379897	0.0061816	0.019577132	PPARD
5649.101434	-0.34356682	0.04085245	-8.409944	4.10E-17	9.26E-16	RPL10A
4026.886941	-0.16095517	0.03969769	-4.0545225	5.02E-05	0.000253811	SRPK1
87.13266578	0.449943542	0.18149894	2.4790423	0.0131736	0.037915981	MAPK13
2300.917131	-0.16390527	0.04474126	-3.6634027	0.0002489	0.001100188	BRPF3
56.60878345	1.95261953	0.29242853	6.6772538	2.43E-11	3.04E-10	ETV7
8092.437209	-0.13634127	0.04586091	-2.97293	0.0029497	0.010157541	KCTD20
7442.804978	-0.10535756	0.04041283	-2.6070323	0.0091331	0.027503376	SRSF3
4219.995145	1.360482341	0.11453217	11.878604	1.53E-32	1.12E-30	CDKN1A
1310.270664	-0.2468403	0.062791	-3.9311414	8.45E-05	0.000409302	PPIL1
4829.691748	0.121075839	0.03929452	3.0812402	0.0020614	0.007388252	MTCH1
748.3101393	-0.59076749	0.0823735	-7.1718147	7.40E-13	1.09E-11	PIM1
56.63107177	1.081534582	0.25501974	4.2409838	2.23E-05	0.000119889	TMEM217
526.0040778	-0.27529128	0.08454787	-3.2560402	0.0011298	0.004305683	TBC1D22B
195.0444643	-0.50885717	0.13213297	-3.8510993	0.0001176	0.000552966	CCDC167
2323.856145	0.264186116	0.05967273	4.4272505	9.54E-06	5.54E-05	MDGA1
10948.40992	-0.19456038	0.03877356	-5.0178624	5.22E-07	3.75E-06	GLO1
24.41575073	0.923983854	0.3824748	2.4158032	0.0157005	0.043994918	DNAH8
63.87459782	0.788152861	0.23276859	3.3859931	0.0007092	0.002833262	DAAM2
329.1164582	-0.34547058	0.10107722	-3.4178875	0.0006311	0.002554629	OARD1

1244.838002	-0.27406978	0.05751647	-4.765066	1.89E-06	1.23E-05	NFYA
7.02988998	3.032813014	0.90371303	3.355947	0.0007909	0.003126711	TREML1
11.34122471	1.459841408	0.56058234	2.6041516	0.0092102	0.027729463	TREM2
863.8398704	0.273254773	0.06626592	4.1236094	3.73E-05	0.00019327	FOXP4
83.89300136	0.537097127	0.19081896	2.8146948	0.0048824	0.015866977	TFEB
287.8456764	-0.46862501	0.11385629	-4.1159343	3.86E-05	0.000199223	USP49
1136.616276	0.34694985	0.06732928	5.1530304	2.56E-07	1.93E-06	CCND3
990.5553108	-0.22441732	0.06335881	-3.5420064	0.0003971	0.001683446	TAF8
816.9393712	0.494065019	0.07140014	6.9196644	4.53E-12	6.16E-11	C6orf132
1672.444339	-0.27382998	0.05355643	-5.1129246	3.17E-07	2.34E-06	MRPS10
1866.81461	-0.18429437	0.05288156	-3.4850402	0.0004921	0.002046634	UBR2
370.3087807	-0.58429966	0.10819035	-5.4006633	6.64E-08	5.45E-07	BICRAL
4070.478852	-0.27246439	0.04329014	-6.2939136	3.10E-10	3.48E-09	RPL7L1
2886.691595	-0.3249685	0.04942992	-6.5743274	4.89E-11	5.97E-10	PPP2R5D
1374.178883	-0.29110511	0.05807239	-5.0127973	5.36E-07	3.83E-06	KLHDC3
615.9756357	-0.21281793	0.08423158	-2.5265811	0.0115179	0.03378456	CUL7
3278.568641	0.301350466	0.05312613	5.6723589	1.41E-08	1.27E-07	PTK7
75.12627472	-0.81821108	0.19363352	-4.2255652	2.38E-05	0.000127728	DNPH1
5.469854934	3.251692043	1.21806998	2.6695445	0.0075954	0.023415303	TTBK1
1369.563383	-0.49532659	0.05531948	-8.9539268	3.43E-19	9.48E-18	ZNF318
273.5543917	0.833153889	0.11442825	7.2810157	3.31E-13	5.09E-12	ABCC10
82.5579152	-0.54858258	0.20455292	-2.6818614	0.0073214	0.022690926	DLK2
424.325748	0.343633091	0.09500365	3.6170513	0.000298	0.00129223	POLR1C
1556.241072	0.129123836	0.05464022	2.3631648	0.0181196	0.049755248	VEGFA
36.99888766	3.374703313	0.52763668	6.3958846	1.60E-10	1.86E-09	ENSG00000289609
904.0678937	0.500515197	0.06645897	7.5311908	5.03E-14	8.46E-13	SLC29A1
40785.39231	-0.1112174	0.03725025	-2.9856817	0.0028295	0.009788042	HSP90AB1
1191.967412	-0.14085563	0.05796664	-2.429943	0.0151012	0.042596571	SLC35B2
160.6800088	0.872752266	0.146519	5.9565809	2.58E-09	2.57E-08	NFKBIE
620.3447897	-0.27425746	0.0766405	-3.5784929	0.0003456	0.001481671	AARS2
4716.205169	-0.2588988	0.04177335	-6.1977031	5.73E-10	6.24E-09	CDC5L
230.3714411	-0.5351796	0.12647668	-4.2314488	2.32E-05	0.000124662	SUPT3H
509.7397516	-0.86777135	0.0827492	-10.486764	9.94E-26	4.66E-24	RUNX2
18.91986713	1.066319312	0.43883495	2.4298869	0.0151035	0.042596571	CLIC5
30.8088797	0.878664481	0.3466286	2.5348874	0.0112483	0.033081133	ENPP5
61.02125115	2.600269141	0.30692686	8.4719505	2.41E-17	5.58E-16	ADGRF5
630.9801706	2.209505643	0.09759073	22.640529	1.73E-113	1.50E-110	TNFRSF21
4881.28143	-0.14353672	0.0442184	-3.2460861	0.00117	0.004442453	CD2AP
984.1444021	-0.60771932	0.07073431	-8.5915783	8.58E-18	2.07E-16	MMUT
1185.83391	-0.26111171	0.05594103	-4.6676245	3.05E-06	1.93E-05	CENPQ
5112.746066	-0.28202758	0.03758971	-7.5027877	6.25E-14	1.04E-12	MCM3
162.3910228	-0.53385474	0.1607543	-3.320936	0.0008972	0.00350441	PAQR8
538.3860642	-0.32329142	0.07870476	-4.1076475	4.00E-05	0.000205771	EFHC1
1169.995814	0.310168932	0.05653325	5.4864868	4.10E-08	3.47E-07	ENSG00000216775
755.6746115	-0.60847219	0.07195486	-8.4563045	2.76E-17	6.34E-16	CILK1
5747.805744	-0.21896585	0.04232118	-5.1739064	2.29E-07	1.73E-06	ELOVL5
1124.985307	0.614962741	0.06369199	9.6552606	4.67E-22	1.63E-20	GCLC
228.8242007	0.315518354	0.12061304	2.6159557	0.0088978	0.026891369	LRRC1

9867.257792	-0.61930398	0.19195513	-3.2262956	0.001254	0.004723352	DST
2432.9923	-0.18090936	0.0456539	-3.9626262	7.41E-05	0.000362927	ZNF451
927.0459816	-0.21206711	0.06372175	-3.3280175	0.0008747	0.003428855	BAG2
1133.767155	-0.25889738	0.07108335	-3.6421665	0.0002704	0.00118454	PRIM2
28.33882855	-0.80161805	0.32400269	-2.4741092	0.0133569	0.0383674	ENSG00000272316
236.2379271	0.371866803	0.11724299	3.1717616	0.0015152	0.005600184	LINC00680
1669.912882	-0.55855452	0.05209743	-10.721346	8.08E-27	4.16E-25	PHF3
5.669905337	2.473299946	0.8899519	2.7791389	0.0054503	0.017496971	ENSG00000227706
519.6459263	-0.35150602	0.08077477	-4.3516806	1.35E-05	7.60E-05	SMAP1
1743.170002	0.508391133	0.05649917	8.9982057	2.29E-19	6.42E-18	OGFRL1
371.4761554	0.296090335	0.10896595	2.717274	0.0065822	0.020707681	LINC00472
249.7608436	0.359535523	0.11469485	3.1347137	0.0017202	0.006294948	CGAS
10776.40044	-0.54570008	0.03824049	-14.270216	3.36E-46	4.14E-44	EEF1A1
4.004808052	3.526354549	1.40478024	2.5102535	0.0120645	0.035143245	ENSG00000289286
5.739621756	2.738946728	0.95619507	2.8644226	0.0041777	0.01380921	FILIP1
1138.605478	-0.19623468	0.05705888	-3.4391612	0.0005835	0.00238538	SENP6
45.78055337	-0.80994307	0.25857934	-3.1322806	0.0017345	0.006338233	IRAK1BP1
1889.107054	-0.50365668	0.06277516	-8.0231841	1.03E-15	2.08E-14	PHIP
359.260596	0.236293065	0.09664932	2.4448498	0.0144912	0.041143315	HMGN3
101.8319985	-0.46430148	0.17034551	-2.7256455	0.0064176	0.020243402	LCA5
151.1042097	0.973269618	0.1633683	5.9575182	2.56E-09	2.56E-08	ELOVL4
303.612762	1.112236182	0.11372079	9.7804117	1.37E-22	5.01E-21	TENT5A
104.7712606	0.834144813	0.18275959	4.5641644	5.01E-06	3.04E-05	TPBG
34.4843795	1.111362063	0.3589153	3.0964466	0.0019586	0.007054516	PRSS35
243.5815697	-0.40044339	0.11753292	-3.4070744	0.0006566	0.002645086	CEP162
615.5807758	1.628904296	0.08933066	18.234549	2.75E-74	7.70E-72	NT5E
1793.254328	-0.34331014	0.05596788	-6.1340565	8.57E-10	9.07E-09	SYNCRIP
1368.507252	-0.53796349	0.05903438	-9.1127149	8.03E-20	2.34E-18	ZNF292
807.978583	-0.16655272	0.0666814	-2.497739	0.0124988	0.036231781	ORC3
842.4134736	-0.23622314	0.06809035	-3.4692601	0.0005219	0.002156269	RNGTT
831.9639421	-0.27403058	0.07469141	-3.6688364	0.0002437	0.00107899	PM20D2
1664.247801	0.397111618	0.05539222	7.1690863	7.55E-13	1.11E-11	UBE2J1
782.2741291	-0.79208057	0.16215204	-4.8848017	1.04E-06	7.04E-06	MDN1
1087.952827	-0.27653887	0.05976827	-4.6268508	3.71E-06	2.31E-05	CASP8AP2
463.1140486	-0.66407688	0.08499694	-7.8129502	5.59E-15	1.03E-13	MANEA
479.9459859	-0.42346638	0.08511053	-4.9754877	6.51E-07	4.58E-06	UFL1
1141.685149	-0.27968201	0.06474399	-4.3198143	1.56E-05	8.67E-05	MMS22L
280.8691169	-0.37552994	0.10836945	-3.465275	0.0005297	0.002183788	FBXL4
138.3712778	-0.86691703	0.14754653	-5.8755502	4.21E-09	4.06E-08	COQ3
208.2251432	-0.7123642	0.12510374	-5.6941877	1.24E-08	1.13E-07	SIM1
13.2036313	2.320472443	0.79182549	2.9305352	0.0033838	0.011450487	GRIK2
198.9717883	0.438627896	0.14193837	3.09027	0.0019997	0.007188009	BVES
551.6371301	-0.30739389	0.08593585	-3.5770157	0.0003475	0.001489699	PREP
84.3644307	-0.6817076	0.19288025	-3.5343567	0.0004088	0.00172956	RTN4IP1
257.6920801	-0.27100391	0.1079591	-2.5102462	0.0120647	0.035143245	QRSL1
305.4865187	1.353213989	0.13512547	10.0145	1.32E-23	5.18E-22	CD24
110.5056361	-0.59677444	0.16492358	-3.6184907	0.0002963	0.001285755	PDSS2
898.8810786	0.573751279	0.06970859	8.2307113	1.86E-16	3.95E-15	OSTM1

1752.816656	-0.13560364	0.05513147	-2.4596414	0.0139076	0.03977191	SNX3
796.0026768	-0.336579	0.06786802	-4.9593164	7.07E-07	4.94E-06	FOXO3
239.7451916	0.734975767	0.12257292	5.9962327	2.02E-09	2.04E-08	SESN1
553.2557859	-0.55740376	0.0800844	-6.960204	3.40E-12	4.66E-11	CEP57L1
235.8518989	0.53699884	0.1197696	4.4835988	7.34E-06	4.33E-05	MICAL1
51.44070236	-0.8353449	0.25289523	-3.3031263	0.0009561	0.003713906	AK9
544.660411	-0.46632082	0.08448101	-5.5198299	3.39E-08	2.90E-07	CDK19
602.7335599	-0.29577561	0.07989287	-3.7021527	0.0002138	0.000960123	RPF2
623.0489262	-0.24936114	0.08453565	-2.949775	0.0031801	0.010841236	REV3L
890.9845367	0.199827188	0.07045952	2.8360565	0.0045674	0.014941257	FYN
9.295579416	1.488884696	0.60691996	2.4531813	0.0141599	0.040373925	LAMA4
2352.539852	-0.49081123	0.05316564	-9.2317365	2.66E-20	8.10E-19	MARCKS
2163.463358	-0.28399106	0.05194913	-5.4667148	4.58E-08	3.85E-07	HDAC2
800.5623335	-0.17534896	0.06968407	-2.5163421	0.011858	0.034645605	TSPYL4
1057.416529	-0.14536755	0.05929176	-2.451733	0.014217	0.040503581	DSE
568.5750936	0.572925099	0.08331992	6.8762083	6.15E-12	8.19E-11	DCBLD1
172.0337688	-0.52032645	0.13913679	-3.7396754	0.0001843	0.000839028	MCM9
31.44346297	1.437650944	0.44369047	3.2402115	0.0011944	0.004522274	MAN1A1
4708.515616	0.57451433	0.04746291	12.104489	1.00E-33	7.76E-32	GJA1
3949.321881	0.158218757	0.04440878	3.562781	0.0003669	0.001565555	SERINC1
5.36327379	2.232624876	0.84812008	2.6324396	0.0084774	0.025804683	RNF217-AS1
81.29422598	0.837367002	0.20941705	3.9985617	6.37E-05	0.000315738	TPD52L1
508.3561586	0.503293303	0.09498828	5.298478	1.17E-07	9.25E-07	NCOA7
275.634121	-0.53803609	0.11431341	-4.7066752	2.52E-06	1.61E-05	HINT3
271.5260019	0.422924097	0.11127307	3.8007768	0.0001442	0.000667835	RNF146
697.6253046	-0.2858231	0.07344012	-3.8919204	9.95E-05	0.000475399	ECHDC1
941.2930074	0.728626273	0.06975899	10.444909	1.55E-25	7.19E-24	PTPRK
24.06455824	0.94864367	0.36388111	2.6070154	0.0091335	0.027503376	LAMA2
1028.158993	-0.417545	0.07246297	-5.7621844	8.30E-09	7.75E-08	ARHGAP18
79.94243522	0.520012539	0.19576622	2.6562935	0.0079005	0.024218115	TMEM200A
674.5127386	-0.22307594	0.07662623	-2.9112217	0.0036002	0.012102688	MED23
11401.46028	0.22449279	0.05259649	4.2682084	1.97E-05	0.000107521	CCN2
3.594217929	3.215156903	1.27909599	2.5136166	0.01195	0.034867612	LINC01013
1774.877639	-0.4044754	0.0549614	-7.359263	1.85E-13	2.93E-12	RPS12
164.4704097	0.346491329	0.14203348	2.4395046	0.0147074	0.041675407	EYA4
1599.970766	0.647855816	0.05512365	11.752775	6.83E-32	4.79E-30	SGK1
1510.55182	-0.16396583	0.05318627	-3.0828603	0.0020502	0.007352696	HBS1L
260.029613	-0.38167348	0.11022192	-3.4627728	0.0005346	0.002202649	AHI1
3470.637242	-0.28551432	0.04430701	-6.4439983	1.16E-10	1.37E-09	BCLAF1
695.0870695	0.59426417	0.07473347	7.9517805	1.84E-15	3.57E-14	IFNGR1
253.9540151	1.827516981	0.12112453	15.087918	1.94E-51	2.92E-49	TNFAIP3
712.2164408	0.48574176	0.07995318	6.0753273	1.24E-09	1.29E-08	PERP
331.2175609	-1.02379301	0.10600668	-9.6578159	4.55E-22	1.60E-20	ARFGEF3
254.9445571	-0.45187493	0.11252935	-4.0156183	5.93E-05	0.000295516	HEBP2
94.81983809	0.480008159	0.18598249	2.580932	0.0098534	0.029461932	NHSL1
220.9267924	0.383002346	0.14003039	2.7351373	0.0062354	0.019729633	ABRACL
3464.986731	-0.424323	0.04882397	-8.690874	3.60E-18	8.99E-17	CITED2
606.5170234	0.358860011	0.08846337	4.0565946	4.98E-05	0.000251864	ADGRG6

313.1349004	-0.56780459	0.10579704	-5.366923	8.01E-08	6.51E-07	HIVEP2
696.371719	0.360480758	0.08557201	4.2126013	2.52E-05	0.000134664	PHACTR2
1927.421589	-0.75246997	0.10726231	-7.015232	2.30E-12	3.19E-11	UTRN
384.0552288	-0.28044167	0.09757444	-2.8741306	0.0040514	0.013445436	SHPRH
421.4606021	-0.30255842	0.099396	-3.0439698	0.0023348	0.00824572	STXBP5
181.1837838	1.57992605	0.17795348	8.8783092	6.79E-19	1.83E-17	SAMD5
1858.23449	-0.28051388	0.05080432	-5.5214573	3.36E-08	2.88E-07	TAB2
712.0003621	-0.32311983	0.07104326	-4.5482122	5.41E-06	3.26E-05	LATS1
1128.06716	-0.14832728	0.06045336	-2.4535821	0.0141441	0.040348811	NUP43
13.41781584	2.093066645	0.60133558	3.4806965	0.0005001	0.002076634	RAET1G
92.17591205	0.623597623	0.19322584	3.2272994	0.0012496	0.004709872	ULBP2
23.35517694	1.240198448	0.40779041	3.0412644	0.0023559	0.008306682	ULBP1
149.9482092	0.428119275	0.14674738	2.9173896	0.0035297	0.011884274	ULBP3
12.34748684	1.590607179	0.64105019	2.4812522	0.0130922	0.037717085	PPP1R14C
695.6781675	-0.27010453	0.07379756	-3.6600742	0.0002521	0.001112882	MTHFD1L
3098.829285	0.814240261	0.04446998	18.309885	6.90E-75	2.00E-72	AKAP12
65.30882123	-0.626198	0.21767271	-2.8767868	0.0040175	0.013348067	RGS17
1045.942627	-0.3899544	0.06299963	-6.1897885	6.02E-10	6.53E-09	SCAF8
124.1220789	-0.43872513	0.15508333	-2.8289639	0.0046699	0.015253478	TFB1M
742.0794084	-0.30584125	0.07469577	-4.0944922	4.23E-05	0.000217039	ARID1B
732.1974888	0.520273135	0.07469007	6.9657609	3.27E-12	4.48E-11	SYNJ2
583.0040385	0.489022434	0.07973449	6.1331354	8.62E-10	9.12E-09	SERAC1
314.5323564	0.311649205	0.10488037	2.9714731	0.0029637	0.0101998	GTF2H5
729.0608782	0.261495131	0.06927841	3.7745545	0.0001603	0.000736657	DYNLT1
56.76613605	0.766400921	0.24636064	3.1108902	0.0018652	0.006759027	SYTL3
5298.125808	0.218278505	0.04784616	4.5620907	5.06E-06	3.07E-05	EZR
5.214818801	2.416780758	0.89326764	2.7055506	0.0068191	0.021370447	LINC02901
48.75424117	1.201703024	0.27392439	4.386988	1.15E-05	6.57E-05	FNDC1
1872.723711	0.507509827	0.0548721	9.2489589	2.27E-20	6.93E-19	SOD2
1655.553964	-0.39133852	0.05327377	-7.345801	2.05E-13	3.22E-12	ACAT2
4102.846193	-0.24378265	0.04765796	-5.1152559	3.13E-07	2.32E-06	TCP1
14.04285809	1.485257271	0.52205825	2.845003	0.0044411	0.014588353	LPAL2
26.52515395	1.362624888	0.39156617	3.4799352	0.0005015	0.002080557	MAP3K4-AS1
381.5189004	-0.27285787	0.09700386	-2.8128557	0.0049104	0.015940136	MAP3K4
450.7442893	-0.67859604	0.08649265	-7.8457074	4.31E-15	8.02E-14	RPS6KA2
1066.559484	-0.31841353	0.06021399	-5.2880321	1.24E-07	9.76E-07	AFDN
4406.747299	0.430727225	0.05843957	7.3704726	1.70E-13	2.70E-12	THBS2
220.897038	-0.29817236	0.12340773	-2.4161562	0.0156853	0.043966456	WDR27
304.7028184	-0.62237572	0.10119119	-6.150493	7.72E-10	8.24E-09	C6orf120
958.8063859	-0.60533096	0.07190537	-8.4184385	3.82E-17	8.64E-16	PHF10
135.6718585	0.408355816	0.15142835	2.6966933	0.0070032	0.021856636	PRKAR1B
255.1422489	-0.54277551	0.11720645	-4.6309355	3.64E-06	2.27E-05	DNAAF5
165.3193767	0.807410768	0.15297622	5.2780149	1.31E-07	1.03E-06	MICALL2
17.95887737	1.11963095	0.45642258	2.4530578	0.0141648	0.040381173	PSMG3-AS1
2858.07484	-0.17497358	0.04893312	-3.57577	0.0003492	0.001496442	EIF3B
236.0281367	0.331414285	0.12409923	2.6705587	0.0075725	0.023356865	CHST12
137.1237844	0.47432347	0.15825723	2.9971678	0.002725	0.009462433	LFNG
677.6643535	0.369686215	0.07982664	4.6311132	3.64E-06	2.27E-05	TTYH3

137.3195913	1.528986313	0.1727698	8.8498471	8.76E-19	2.33E-17	AMZ1
860.1667467	-0.30657824	0.07891729	-3.8848043	0.0001024	0.000488065	FOXK1
149.9710627	0.668091047	0.15080016	4.4303073	9.41E-06	5.46E-05	AP5Z1
498.7680919	-0.44371505	0.08461347	-5.2440236	1.57E-07	1.22E-06	TNRC18
401.8877142	0.671025961	0.09951416	6.74302	1.55E-11	1.97E-10	FBXL18
58393.95875	0.149697211	0.03682666	4.0649141	4.81E-05	0.000244182	ACTB
1299.225826	0.703111379	0.05635074	12.477413	9.92E-36	8.41E-34	FSCN1
2043.295441	-0.19653823	0.05413096	-3.6307916	0.0002826	0.001231479	EIF2AK1
220.0917646	-0.36987585	0.12323096	-3.0014847	0.0026867	0.009340485	USP42
296.8636487	0.45302691	0.11204126	4.0433935	5.27E-05	0.000264556	DAGLB
646.6327508	-0.23776755	0.07209162	-3.2981303	0.0009733	0.003773039	ZNF12
383.7158211	-0.42470761	0.09002546	-4.7176388	2.39E-06	1.54E-05	RPA3
125.3388611	-0.39951853	0.16146198	-2.4743815	0.0133467	0.038344506	UMAD1
305.4562796	1.259517236	0.11530207	10.923631	8.89E-28	4.84E-26	GLCCI1
489.9176338	-0.33696548	0.0835737	-4.0319561	5.53E-05	0.000276891	PHF14
671.1407278	-0.53356927	0.08470586	-6.2990836	2.99E-10	3.37E-09	TMEM106B
8.238928746	1.873108072	0.73910362	2.534297	0.0112673	0.03313133	SCIN
90.32203426	-0.84436075	0.18663782	-4.5240603	6.07E-06	3.62E-05	ETV1
303.10487	-0.34838655	0.10882438	-3.2013649	0.0013678	0.005107433	ANKMY2
535.1546922	0.896542716	0.08418009	10.650294	1.74E-26	8.66E-25	TSPAN13
682.5782401	0.591485149	0.08295529	7.1301677	1.00E-12	1.46E-11	AHR
594.0546532	-0.5204482	0.07623982	-6.8264615	8.70E-12	1.14E-10	SNX13
109.712042	1.497477855	0.178832	8.373657	5.59E-17	1.25E-15	HDAC9
1238.788482	-1.14564487	0.06474098	-17.695821	4.52E-70	1.19E-67	ITGB8
244.099035	-0.42392585	0.11697616	-3.6240364	0.00029	0.001260952	SP4
1021.079758	-0.6519419	0.06395008	-10.194544	2.10E-24	8.85E-23	CDCA7L
252.827961	2.366814837	0.1575847	15.019319	5.49E-51	8.16E-49	IL6
159.7254257	0.522994756	0.15324078	3.4128955	0.0006428	0.002595843	SNHG26
1909.659828	0.338390087	0.05067743	6.6773328	2.43E-11	3.04E-10	FAM126A
5.194677792	3.008427814	1.23910634	2.4279012	0.0151865	0.042809669	GPNMB
313.3783163	-0.46918052	0.1051167	-4.4634251	8.07E-06	4.74E-05	PALS2
16377.46947	-0.36817356	0.0322033	-11.432789	2.87E-30	1.83E-28	HNRNPA2B1
3150.153916	-0.28198891	0.04409255	-6.3953861	1.60E-10	1.87E-09	CBX3
58.95891103	-0.81309994	0.23331128	-3.4850435	0.0004921	0.002046634	SKAP2
340.0146904	-0.48411608	0.09926556	-4.8769793	1.08E-06	7.31E-06	HIBADH
2914.170093	0.194971274	0.04669274	4.175623	2.97E-05	0.000156838	TAX1BP1
3552.80793	-0.18588352	0.04711895	-3.944984	7.98E-05	0.000388526	SCRN1
697.7948253	-0.31613153	0.07214281	-4.3820242	1.18E-05	6.70E-05	FKBP14
146.6557334	0.366238676	0.15210043	2.407874	0.0160457	0.044853726	MTURN
1145.802356	-1.21934445	0.06133634	-19.879642	6.11E-88	2.95E-85	PDE1C
554.4496162	-0.26269566	0.07989044	-3.288199	0.0010083	0.003891462	AVL9
709.3307542	0.448589694	0.07469522	6.0056008	1.91E-09	1.94E-08	FKBP9
152.4968897	-1.05732038	0.14292894	-7.3975248	1.39E-13	2.22E-12	BBS9
1034.149324	-0.27644015	0.06294469	-4.3917943	1.12E-05	6.45E-05	DPY19L1
134.0238829	-0.58654987	0.15091348	-3.8866631	0.0001016	0.000484876	DPY19L2P1
20.5868831	1.12327924	0.4553726	2.4667256	0.0136355	0.039064417	EEPD1
12033.14772	-0.14856982	0.0385955	-3.8494074	0.0001184	0.000556649	ANLN
533.2093125	0.51360117	0.0835778	6.1451865	7.99E-10	8.51E-09	AMPH

906.0645555	0.233612806	0.06488092	3.6006398	0.0003174	0.001368079	RALA
22.40229379	1.145153141	0.45416303	2.5214583	0.011687	0.034197562	LINC00265
1664.257122	0.600125247	0.06006631	9.9910464	1.67E-23	6.48E-22	INHBA
15.20059881	1.444799732	0.50227868	2.8764903	0.0040212	0.013358065	LUARIS
944.119761	0.590013933	0.06707176	8.796757	1.41E-18	3.64E-17	STK17A
559.2117385	-0.23153298	0.07952483	-2.9114553	0.0035975	0.012095982	COA1
392.271928	0.403308941	0.09375645	4.301666	1.70E-05	9.36E-05	URGCP
173.5222997	0.573462349	0.15594966	3.6772272	0.0002358	0.001047322	UBE2D4
653.3599368	-0.30930286	0.07165922	-4.3163025	1.59E-05	8.80E-05	POLD2
7.514203897	3.02876133	1.02023421	2.9686922	0.0029907	0.010284412	MYL7
1266.712225	0.133370198	0.05559705	2.3988717	0.0164457	0.045831774	YKT6
827.537157	0.181157785	0.07365714	2.4594735	0.0139141	0.039783982	OGDH
392.1017104	0.24891905	0.0945734	2.6320197	0.0084879	0.025823038	ZMIZ2
2763.027252	-0.33039294	0.04399418	-7.509923	5.92E-14	9.88E-13	H2AZ2
1823.558908	0.700478799	0.06452748	10.855511	1.88E-27	9.98E-26	IGFBP3
504.1973269	-0.90745748	0.09030548	-10.048753	9.30E-24	3.72E-22	TNS3
14.74442353	1.624079002	0.53572505	3.0315532	0.002433	0.008552598	C7orf57
94.87048626	1.070432682	0.19645873	5.448639	5.08E-08	4.24E-07	UPP1
346.2612793	0.370849391	0.11520406	3.2190653	0.0012861	0.004833616	GRB10
1906.987723	-0.5825442	0.06333541	-9.1977652	3.65E-20	1.09E-18	EGFR
501.5884419	-0.5446778	0.08795472	-6.1927071	5.91E-10	6.42E-09	NIPSNAP2
4472.071802	-0.1539955	0.04318756	-3.5657377	0.0003628	0.001548389	CCT6A
228.8885115	-0.29008382	0.12109242	-2.3955573	0.0165951	0.046181655	ZNF736
467.3898999	-0.38757837	0.08963561	-4.3239329	1.53E-05	8.52E-05	ZNF680
275.8155537	-0.65506016	0.11023459	-5.9424191	2.81E-09	2.78E-08	ZNF138
2126.726495	-0.37835674	0.05313528	-7.1206315	1.07E-12	1.56E-11	VKORC1L1
795.4150507	0.19058998	0.07391258	2.5785866	0.0099205	0.029642999	TPST1
2563.209991	0.298955992	0.04769566	6.2679916	3.66E-10	4.08E-09	SBDS
895.9895835	0.275377227	0.07502363	3.6705399	0.000242	0.001072643	SBDSP1
750.0390028	-0.27698205	0.07457662	-3.7140601	0.000204	0.00092007	POM121
65.33613195	0.515905426	0.21725094	2.3746983	0.0175633	0.048403453	NSUN5P2
89.8287934	-0.59987357	0.19116121	-3.1380507	0.0017008	0.006227863	GTF2IP4
146.0261501	0.552344558	0.14794979	3.7333245	0.000189	0.00085772	NSUN5
6036.808021	-0.20351374	0.04272247	-4.7636229	1.90E-06	1.24E-05	BAZ1B
643.1399024	0.257461438	0.07614919	3.3810136	0.0007222	0.002880476	BCL7B
102.9502453	1.084584743	0.18444528	5.880252	4.10E-09	3.96E-08	STX1A
172.6232665	0.450974836	0.13966904	3.2288819	0.0012428	0.004689986	ABHD11
11.80791598	2.714345162	0.73316022	3.7022537	0.0002137	0.000960038	CLDN4
492.3658364	-0.32177043	0.08282346	-3.8850158	0.0001023	0.000487775	LIMK1
3876.962657	-0.17669613	0.04025561	-4.3893543	1.14E-05	6.51E-05	EIF4H
16.35274836	1.534570675	0.48130245	3.1883708	0.0014308	0.00531647	LAT2
353.6544957	-0.59335778	0.10076089	-5.8887706	3.89E-09	3.77E-08	CLIP2
398.6910363	-0.323947	0.09569847	-3.3850802	0.0007116	0.002840645	GTF2IRD1
1589.339093	-0.51540636	0.05279674	-9.7620877	1.64E-22	5.98E-21	GTF2I
747.0513317	-0.24552468	0.07142739	-3.4374022	0.0005873	0.002398672	POM121C
630.9046595	-0.7800985	0.08344975	-9.3481227	8.92E-21	2.81E-19	HIP1
317.8986886	0.326376038	0.1035728	3.1511752	0.0016261	0.005979855	RHBDD2
623.457067	0.236822913	0.08180921	2.8948199	0.0037938	0.012684775	POR

1983.750972	0.158529564	0.04805874	3.2986622	0.0009715	0.003767578	MDH2
10.04636948	2.024176748	0.64116596	3.1570247	0.0015939	0.005873607	ENSG00000230882
12.66452139	1.695007914	0.5724021	2.9612189	0.0030642	0.01050407	GTF2IP7
15.21852184	-1.1636763	0.44634864	-2.6071017	0.0091312	0.027503376	SRRM3
2077.4331	0.196686922	0.06002308	3.2768546	0.0010497	0.00402921	HSPB1
7.411385938	2.052390426	0.77361318	2.6529931	0.0079781	0.024426061	SSC4D
3.718034767	3.983264316	1.39392586	2.8575869	0.0042688	0.014064737	UPK3B
2573.645805	-0.2122575	0.04762962	-4.4564177	8.33E-06	4.88E-05	PTPN12
1065.311313	-0.32932793	0.060907	-5.4070625	6.41E-08	5.28E-07	RSBN1L
57.81990254	-0.60316941	0.23078458	-2.6135602	0.0089604	0.027057104	MAGI2
1468.779566	-0.22147445	0.05690262	-3.892166	9.94E-05	0.000475049	MAGI2-AS3
1132.204099	0.365861423	0.06504561	5.6246902	1.86E-08	1.65E-07	GNAI1
3101.276166	0.351182755	0.0451189	7.7834951	7.05E-15	1.28E-13	SEMA3C
895.6539486	-0.20243413	0.07028058	-2.8803709	0.0039721	0.013222511	CACNA2D1
372.0116006	1.419478738	0.11080891	12.81015	1.44E-37	1.33E-35	SEMA3D
49.68962915	1.285473862	0.29453679	4.3643914	1.27E-05	7.22E-05	ELAPOR2
17.78435778	1.45083633	0.46828062	3.0982199	0.0019469	0.007025535	ABCB4
1555.825305	1.445965975	0.06466021	22.362531	9.12E-111	7.21E-108	ABCB1
577.28628	-0.40502436	0.08475897	-4.7785426	1.77E-06	1.16E-05	SLC25A40
1271.474294	-0.25977537	0.06101335	-4.2576812	2.07E-05	0.000112216	DBF4
216.1688729	-0.32076046	0.12492985	-2.5675245	0.0102428	0.030468952	ADAM22
909.3396022	-0.37036197	0.06356423	-5.8265783	5.66E-09	5.38E-08	SRI
74.15319471	-0.87789366	0.20001728	-4.3890891	1.14E-05	6.52E-05	STEAP2
34.27247538	-0.72256854	0.28912625	-2.4991454	0.0124493	0.036118404	CFAP69
649.8290511	-0.19087999	0.07585989	-2.5162175	0.0118622	0.034652034	GTPBP10
1018.036203	-0.39636702	0.07127181	-5.5613434	2.68E-08	2.33E-07	CDK14
557.8502583	0.34921333	0.08997375	3.88128	0.0001039	0.000494516	FZD1
266.0371447	-0.30902375	0.11463566	-2.6957035	0.007024	0.021902032	KRIT1
2445.998322	-0.16871682	0.04470455	-3.7740411	0.0001606	0.00073798	ANKIB1
884.0399452	-0.18913675	0.06447162	-2.9336435	0.0033501	0.011356338	GATAD1
21.87133161	1.230427289	0.40721554	3.0215627	0.0025147	0.008813232	ENSG00000244055
458.5652647	-0.40645176	0.08454852	-4.8073194	1.53E-06	1.02E-05	PEX1
10.55100665	1.898389655	0.59651866	3.1824481	0.0014604	0.005419464	CDK6-AS1
954.20009	1.488607389	0.08761693	16.989951	9.75E-65	2.23E-62	SAMD9
230.3265258	2.476992872	0.15112179	16.390706	2.23E-60	4.67E-58	SAMD9L
637.5640469	-0.39694076	0.07255017	-5.4712592	4.47E-08	3.76E-07	VPS50
413.7477196	0.695893818	0.09374658	7.4231381	1.14E-13	1.85E-12	TFPI2
213.2957986	0.660651886	0.12454849	5.3043749	1.13E-07	8.97E-07	GNG11
632.5128362	-0.26768479	0.07127757	-3.7555265	0.000173	0.000791391	BET1
949.7257462	-0.22823717	0.06291766	-3.6275536	0.0002861	0.001245773	SGCE
14404.17074	0.250486177	0.04419598	5.6676235	1.45E-08	1.30E-07	PEG10
335.8157503	-0.5361753	0.10197101	-5.2581148	1.46E-07	1.14E-06	PPP1R9A
1214.297441	-0.30190018	0.05931532	-5.0897509	3.59E-07	2.63E-06	SEM1
1036.98479	-0.26731685	0.06186606	-4.3208968	1.55E-05	8.63E-05	LMTK2
841.7735199	0.246212757	0.07493203	3.2858146	0.0010169	0.00392009	BRI3
1235.515104	-0.14972301	0.05705903	-2.624002	0.0086903	0.026342129	BAIAP2L1
2140.004864	-0.61739512	0.06753002	-9.1425285	6.10E-20	1.79E-18	TRRAP
62.3409909	0.537478307	0.21663184	2.4810679	0.0130989	0.037719919	ARPC1B

2272.279053	-0.23539178	0.04693393	-5.0153862	5.29E-07	3.78E-06	PDAP1
539.5705454	-0.2523542	0.08263826	-3.0537211	0.0022602	0.008019841	CPSF4
298.4677485	0.818808898	0.12937714	6.328853	2.47E-10	2.81E-09	ZNF789
2141.187973	-0.17859653	0.04628331	-3.8587671	0.000114	0.000537357	ZNF655
344.6557474	0.292168395	0.09899557	2.9513278	0.0031641	0.010800772	ZSCAN25
2322.117701	-0.3941256	0.06047013	-6.5176909	7.14E-11	8.59E-10	ZKSCAN1
1793.825609	-0.22035691	0.05092079	-4.3274452	1.51E-05	8.40E-05	COPS6
4132.887418	-0.31561839	0.04717199	-6.6908005	2.22E-11	2.78E-10	MCM7
870.7103987	-0.37548469	0.0676515	-5.5502791	2.85E-08	2.47E-07	TAF6
185.5233102	0.509830831	0.13970553	3.6493247	0.0002629	0.001156974	TRAPPC14
35.97927598	1.275868355	0.3293655	3.8737158	0.0001072	0.000508319	GAL3ST4
226.5739524	0.459249594	0.13511581	3.3989332	0.0006765	0.00271879	CASTOR3
95.52923204	0.442934004	0.18526457	2.3908188	0.0168108	0.046673172	ZCWPW1
91.99414311	-0.61623661	0.19678259	-3.1315606	0.0017388	0.006351126	PPP1R35
372.2823941	0.241455797	0.09845942	2.4523382	0.0141931	0.040448764	TSC22D4
277.250918	0.430924542	0.11362349	3.7925655	0.0001491	0.00068885	AGFG2
883.6622612	-0.19447453	0.06278109	-3.097661	0.0019505	0.007035877	TRIP6
31.47086271	1.721840431	0.35858938	4.801705	1.57E-06	1.04E-05	ACHE
27764.52804	0.549598601	0.03810074	14.42488	3.61E-47	4.75E-45	SERPINE1
1066.695517	0.226335862	0.06121054	3.6976616	0.0002176	0.000976005	PLOD3
401.9734972	-0.22845005	0.09152927	-2.4959233	0.012563	0.036387433	IFT22
1511.384674	-0.31227801	0.06023994	-5.1839033	2.17E-07	1.65E-06	CUX1
19.6628723	-0.96444381	0.39641585	-2.4329093	0.0149781	0.042304421	RASA4
284.9802129	-0.54846514	0.1098205	-4.9941964	5.91E-07	4.19E-06	ARMC10
1210.774437	-0.21483042	0.06266237	-3.4283802	0.0006072	0.002472281	PMPCB
2146.263572	-0.23597291	0.05113752	-4.614477	3.94E-06	2.45E-05	PSMC2
1814.680988	-0.52018356	0.05054163	-10.29218	7.64E-25	3.35E-23	KMT2E
1876.760559	-0.21399918	0.04987984	-4.2902941	1.78E-05	9.80E-05	NAMPT
154.8605787	0.4822759	0.14434077	3.3412313	0.0008341	0.003283812	CCDC71L
493.1750931	0.258546563	0.09247085	2.7959792	0.0051743	0.016697086	PRKAR2B
1114.426821	-0.36187446	0.06873682	-5.264638	1.40E-07	1.10E-06	HBP1
1035.204811	-0.40189369	0.06301591	-6.3776549	1.80E-10	2.08E-09	COG5
62.28745151	-0.56676059	0.21443204	-2.6430779	0.0082156	0.025082425	DUS4L
1191.868982	-0.22676802	0.05714543	-3.9682618	7.24E-05	0.000355453	CBLL1
2117.954609	-0.17282291	0.05287376	-3.268595	0.0010808	0.004135449	DLD
88.04535044	1.694227386	0.22198416	7.6321995	2.31E-14	4.01E-13	NRCAM
722.9486645	-0.38334853	0.07413201	-5.1711609	2.33E-07	1.76E-06	THAP5
365.705244	0.452142139	0.10569244	4.2779041	1.89E-05	0.000103138	DNAJB9
45.42133969	-0.94293553	0.24738231	-3.811653	0.000138	0.000642023	IMMP2L
246.2095444	0.340676425	0.12091906	2.8173924	0.0048415	0.01574019	IFRD1
418.3798275	-0.23680383	0.09333303	-2.5371923	0.0111746	0.032919791	TMEM168
498.7974378	-0.28723466	0.0831919	-3.4526758	0.0005551	0.002278125	SMIM30
661.7175982	-0.51487909	0.07495399	-6.8692688	6.45E-12	8.56E-11	MDFIC
3356.918372	0.371314016	0.04606409	8.0608127	7.58E-16	1.54E-14	TES
12085.33699	-0.15188034	0.03718179	-4.0848043	4.41E-05	0.00022563	CAV1
8822.713705	-0.32198104	0.04476566	-7.1925897	6.36E-13	9.45E-12	MET
15.77161654	1.440630579	0.52669985	2.7352022	0.0062342	0.019729332	WNT2
77.73542729	1.314457953	0.21852036	6.0152654	1.80E-09	1.83E-08	TSPAN12

110.5714816	-0.57732279	0.16984333	-3.399149	0.000676	0.002717273	CPED1
518.0038015	0.218310486	0.0858233	2.5437204	0.0109679	0.032382262	FAM3C
867.6978242	-0.38255423	0.07438627	-5.1428071	2.71E-07	2.03E-06	AASS
953.1312119	-0.19686397	0.0599713	-3.2826363	0.0010284	0.003954033	NDUFA5
599.5477507	-0.4130877	0.08426423	-4.9022901	9.47E-07	6.48E-06	POT1
4629.276594	-0.13820725	0.04281512	-3.2280011	0.0012466	0.004699352	SND1
213.9068878	-0.55081227	0.11946348	-4.610717	4.01E-06	2.49E-05	HILPDA
544.2334494	-0.37893302	0.07935774	-4.7749975	1.80E-06	1.18E-05	METTL2B
7.482744223	3.490766956	1.01191517	3.4496636	0.0005613	0.002300979	FAM71F2
11.98099033	3.333104932	0.77192947	4.3178879	1.58E-05	8.74E-05	FAM71F1
271.8521422	0.411224154	0.11530536	3.5663925	0.0003619	0.001544906	CCDC136
14254.18344	-0.12460013	0.04412538	-2.8237745	0.0047462	0.015479374	FLNC
752.4146334	0.26982966	0.07118486	3.7905484	0.0001503	0.000694284	ATP6V1F
10.80272139	1.540681822	0.60373551	2.5519152	0.0107133	0.031727449	ATP6V1FNB
1964.062091	-0.39208011	0.04993697	-7.8514997	4.11E-15	7.68E-14	TNPO3
221.2523333	-0.6641505	0.12554893	-5.2899733	1.22E-07	9.67E-07	SMO
496.4388414	0.376283674	0.0903485	4.1648025	3.12E-05	0.000163967	STRIP2
310.0855813	-0.47250194	0.1111961	-4.2492671	2.14E-05	0.000115792	NRF1
3519.568449	0.195274124	0.04129908	4.7282919	2.26E-06	1.46E-05	UBE2H
1444.256596	-0.26399685	0.06078274	-4.3432863	1.40E-05	7.87E-05	KLHDC10
7818.410126	1.006614408	0.10407645	9.671875	3.97E-22	1.40E-20	CPA4
488.4552689	-0.22630806	0.08400103	-2.6941106	0.0070577	0.021983334	CEP41
4988.465508	-0.3221434	0.05231468	-6.1578015	7.38E-10	7.89E-09	MEST
620.9010864	-0.68728738	0.08243192	-8.3376365	7.58E-17	1.67E-15	COPG2
63.55388975	1.001787044	0.23326528	4.2946256	1.75E-05	9.63E-05	ENSG00000285106
65.40692416	0.972322945	0.22334725	4.3534136	1.34E-05	7.55E-05	ENSG00000226380
1939.945764	-0.59161753	0.05708204	-10.364337	3.60E-25	1.63E-23	MKLN1
1008.988369	0.794919754	0.06722179	11.825328	2.89E-32	2.08E-30	PODXL
5.020006909	2.230237162	0.93249731	2.3916821	0.0167714	0.046585707	PLXNA4
1559.062899	-0.16191936	0.05442639	-2.9750157	0.0029297	0.010094729	CHCHD3
1540.822687	-0.43649317	0.05152575	-8.4713603	2.43E-17	5.60E-16	EXOC4
807.1608498	-0.40244864	0.07006956	-5.7435589	9.27E-09	8.59E-08	SLC35B4
1630.581473	0.625563268	0.05679549	11.014313	3.26E-28	1.86E-26	AKR1B1
672.6239728	0.745376523	0.078087	9.545462	1.36E-21	4.54E-20	BPGM
35887.18973	0.554589757	0.03916712	14.159576	1.63E-45	1.95E-43	CALD1
7.888253613	2.271893756	0.74844431	3.0354881	0.0024015	0.008450327	TMEM140
3749.942171	-0.46086535	0.04408163	-10.454816	1.39E-25	6.49E-24	NUP205
8223.789794	-0.1548922	0.04565149	-3.3929274	0.0006915	0.002772294	MTPN
4.777773815	2.566216449	1.06744766	2.4040677	0.0162138	0.045258019	CHRM2
6162.393007	-0.41613974	0.04675706	-8.9000408	5.58E-19	1.51E-17	CREB3L2
55.28452159	1.48543957	0.27089458	5.48346	4.17E-08	3.52E-07	ATP6V0A4
2015.67637	0.630252261	0.05625109	11.204268	3.89E-29	2.31E-27	ZC3HAV1
450.9518611	-0.44852508	0.08616574	-5.2053758	1.94E-07	1.49E-06	TTC26
565.9287254	-0.24942095	0.09239669	-2.6994577	0.0069453	0.021707055	UBN2
1552.968454	-0.66277675	0.07508225	-8.8273425	1.07E-18	2.83E-17	HIPK2
250.3018027	1.079841386	0.12487986	8.6470418	5.29E-18	1.29E-16	PARP12
41.99374668	0.658365379	0.27018216	2.4367463	0.0148201	0.041933142	KDM7A-DT
1531.520904	0.243333013	0.05760158	4.2244158	2.40E-05	0.000128342	SLC37A3

1657.558503	-0.19048777	0.05704535	-3.339234	0.0008401	0.003303776	MKRN1
329.4702194	-0.91790896	0.09975483	-9.201649	3.52E-20	1.06E-18	DENND2A
475.1903433	0.270208768	0.08681155	3.11259	0.0018545	0.006724428	NDUFB2
924.007139	-0.29980531	0.06442041	-4.6538873	3.26E-06	2.05E-05	BRAF
602.0970965	0.41445252	0.0941178	4.403551	1.06E-05	6.13E-05	MRPS33
1872.636152	0.327025749	0.05707938	5.7293147	1.01E-08	9.28E-08	TMEM178B
882.2394224	-0.22026946	0.07039682	-3.1289687	0.0017542	0.006400695	AGK
687.2195432	-0.47200511	0.07681036	-6.1450709	7.99E-10	8.51E-09	DENND11
1779.062364	-0.30405532	0.04935594	-6.1604607	7.25E-10	7.77E-09	SSBP1
734.7299051	0.60281508	0.07641301	7.8889066	3.05E-15	5.77E-14	GSTK1
4096.324139	0.251327252	0.0426535	5.8923008	3.81E-09	3.69E-08	ZYX
992.7824677	-0.49041525	0.06975083	-7.0309592	2.05E-12	2.86E-11	TCAF1
5.832186107	2.298058855	0.88464726	2.5977121	0.0093847	0.028210924	EEF1A1P10
1828.331369	-0.17395249	0.05450733	-3.1913597	0.001416	0.005266272	CUL1
1723.548896	-0.40801402	0.05300134	-7.6981826	1.38E-14	2.46E-13	EZH2
44.14891214	1.192365064	0.31292601	3.8103738	0.0001388	0.000644837	ZNF425
494.6161763	-0.32067501	0.08294612	-3.8660639	0.0001106	0.000522957	REPIN1
723.9745334	0.345354058	0.07430511	4.6477836	3.36E-06	2.10E-05	CHPF2
1304.114091	-0.16180118	0.05427978	-2.980874	0.0028743	0.009923278	NUB1
948.8733457	-0.1951382	0.06659418	-2.9302589	0.0033868	0.011458442	RHEB
755.2440577	0.372659173	0.07590803	4.9093509	9.14E-07	6.27E-06	PRKAG2
669.1810352	-0.19516691	0.07332666	-2.6616091	0.0077768	0.023893777	GALNT11
1715.12289	-0.65973195	0.13454568	-4.9034048	9.42E-07	6.45E-06	KMT2C
1378.904771	-0.15427917	0.06043976	-2.5526105	0.0106919	0.031669585	XRCC2
553.791976	-0.43328912	0.07903639	-5.4821469	4.20E-08	3.55E-07	PAXIP1
1587.307711	-1.3785731	0.13088149	-10.532987	6.09E-26	2.89E-24	INSIG1
1224.282315	-0.25234621	0.05627904	-4.4838402	7.33E-06	4.32E-05	RBM33
1555.035943	-0.24304254	0.05465407	-4.4469247	8.71E-06	5.08E-05	LMBR1
3866.985109	-0.38537553	0.03913397	-9.8475948	7.02E-23	2.65E-21	NCAPG2
370.290948	-0.41671593	0.0962325	-4.3303035	1.49E-05	8.31E-05	FBXO25
1448.825967	-0.36366547	0.05531147	-6.5748656	4.87E-11	5.95E-10	AGPAT5
27.34450885	0.938996007	0.3519631	2.667882	0.0076331	0.023506237	FAM86B3P
314.6115656	1.2614952	0.11631392	10.845608	2.09E-27	1.11E-25	PRAG1
8.456065851	1.901239447	0.65125721	2.9193373	0.0035078	0.011824627	CLDN23
1335.358676	-0.13522236	0.0556208	-2.4311474	0.0150511	0.042476222	ERI1
509.6466902	-0.43614711	0.08358862	-5.2177811	1.81E-07	1.40E-06	PPP1R3B
1836.897107	-0.53816719	0.06167207	-8.7262712	2.63E-18	6.65E-17	TNKS
166.9063918	0.431852139	0.14068358	3.0696697	0.002143	0.007650586	PINX1.1
2624.974351	-0.32507426	0.06108826	-5.3213866	1.03E-07	8.23E-07	FDFT1
5296.713578	0.605829061	0.04468206	13.558665	7.04E-42	7.70E-40	CTSB
100.1575094	-1.1541969	0.18587049	-6.2096833	5.31E-10	5.80E-09	LONRF1
6875.659414	-0.31122532	0.04066557	-7.6532887	1.96E-14	3.43E-13	DLC1
1974.886861	-0.13969403	0.0515101	-2.7119739	0.0066884	0.021002353	TUSC3
1022.115764	-0.41957886	0.06337849	-6.6202093	3.59E-11	4.42E-10	ZDHHC2
3263.806029	-0.37543251	0.04146881	-9.0533702	1.39E-19	3.94E-18	CNOT7
4930.809231	-0.49051794	0.04437678	-11.053482	2.11E-28	1.21E-26	PCM1
931.27473	0.551210607	0.06870804	8.022505	1.04E-15	2.09E-14	ASAH1
198.5394359	0.486690237	0.1350722	3.6031858	0.0003143	0.001356086	NAT1

5.972433669	2.337085096	0.94165824	2.4818825	0.013069	0.037664989	NAT2
863.4291182	-0.62016322	0.07025395	-8.8274497	1.07E-18	2.83E-17	PSD3
14.1256218	1.391254623	0.52221879	2.6641221	0.007719	0.023745376	ENSG00000187229
2604.414771	0.263619828	0.05906792	4.462995	8.08E-06	4.74E-05	SH2D4A
935.7992942	-0.27870974	0.06418442	-4.3423273	1.41E-05	7.89E-05	INTS10
378.1864411	0.372574367	0.09906529	3.7608973	0.0001693	0.000775814	LZTS1
2971.31968	-0.53109743	0.04523107	-11.741873	7.77E-32	5.39E-30	XPO7
509.4487961	0.234749299	0.08415922	2.7893472	0.0052814	0.017005046	REEP4
771.7567264	0.743083704	0.07381613	10.066685	7.75E-24	3.14E-22	BMP1
7.923642493	2.187618698	0.73031313	2.995453	0.0027404	0.009506308	PHYHIP
2002.250372	-0.15252016	0.04721538	-3.2303069	0.0012366	0.004668698	CCAR2
3129.540823	0.466141923	0.04262891	10.934877	7.85E-28	4.31E-26	TNFRSF10B
3.432070573	3.63185076	1.40974491	2.5762468	0.0099879	0.029802706	ENSG00000246130
5.620479481	2.498909509	0.90197469	2.7704874	0.0055972	0.017915723	ENSG00000253616
3965.132764	-0.50736658	0.05680886	-8.9311175	4.22E-19	1.16E-17	TNFRSF10D
42.06288503	1.318591051	0.32403546	4.0692801	4.72E-05	0.000240073	TNFRSF10A
1513.611988	-0.30367505	0.05536535	-5.4849297	4.14E-08	3.50E-07	ENTPD4
910.1991842	0.221721785	0.06621908	3.3483067	0.0008131	0.003209826	SLC25A37
440.873362	0.920835067	0.10615152	8.6747233	4.15E-18	1.03E-16	NKX3-1
182.9862579	1.127179038	0.1520266	7.4143541	1.22E-13	1.97E-12	STC1
19.88904979	1.015995196	0.41849526	2.4277341	0.0151935	0.042822465	ENSG00000287185
1160.734408	-0.20911026	0.06377822	-3.2787094	0.0010428	0.004006807	KCTD9
125.4251538	-0.49093399	0.16221619	-3.0264179	0.0024747	0.008685174	ENSG00000289357
2645.219373	-0.1495041	0.05100943	-2.930911	0.0033797	0.011441096	PPP2R2A
9276.636393	-0.34486072	0.03949437	-8.7318964	2.50E-18	6.34E-17	BNIP3L
356.6622508	0.322489944	0.10029808	3.2153151	0.001303	0.004888762	TRIM35
71.02196996	0.514170789	0.21003232	2.4480556	0.014363	0.040832385	PTK2B
1009.901685	1.179837717	0.08151979	14.473022	1.79E-47	2.40E-45	CLU
1299.75402	-0.43833018	0.05536409	-7.9172295	2.43E-15	4.64E-14	PBK
928.1624295	-1.00699483	0.06890525	-14.614197	2.28E-48	3.15E-46	ZNF395
8.691086014	2.22697173	0.71167809	3.129184	0.0017529	0.006397589	EXTL3-AS1
337.6879135	-0.29196449	0.09724649	-3.002314	0.0026794	0.009318801	INTS9
362.8324602	-0.48078579	0.10502919	-4.5776396	4.70E-06	2.87E-05	HMBOX1
72.33601417	-0.94909258	0.22436089	-4.2302051	2.33E-05	0.000125276	ENSG00000259366
631.1004889	1.107051486	0.10069406	10.994208	4.07E-28	2.32E-26	DUSP4
11.19047001	1.526656338	0.57074918	2.6748288	0.0074767	0.023110656	RBPMS-AS1
43.48896005	0.816772223	0.27109832	3.0128265	0.0025883	0.009032708	ENSG00000279041
740.8019725	-0.17848971	0.07480187	-2.3861663	0.0170251	0.0471292	GTF2E2
1062.728825	-0.2576254	0.06395411	-4.028285	5.62E-05	0.000281007	WRN
89.04793616	0.491077319	0.2014695	2.4374773	0.0147901	0.041868892	RNF122
222.1182274	0.432583716	0.12329163	3.508622	0.0004504	0.00188612	ZNF703
1161.936879	-0.40561832	0.06548454	-6.1941079	5.86E-10	6.37E-09	ERLIN2
909.6551928	-0.21100469	0.06670375	-3.1633106	0.0015599	0.005757999	PLPBP
1059.068218	-0.16669299	0.06377614	-2.6137204	0.0089562	0.027049119	BAG4
3309.99703	-0.40149484	0.04635431	-8.6614346	4.66E-18	1.15E-16	NSD3
2160.757452	0.239873667	0.04906635	4.8887609	1.01E-06	6.91E-06	FGFR1
8714.812204	-0.15514841	0.03796871	-4.0862173	4.38E-05	0.000224393	TACC1
1525.145749	-0.36986507	0.05784768	-6.3937754	1.62E-10	1.88E-09	PLEKHA2

1405.6613	0.32822669	0.05779566	5.6790892	1.35E-08	1.23E-07	TM2D2
15965.64122	-0.20396127	0.03631918	-5.615801	1.96E-08	1.74E-07	ADAM9
4344.001737	-1.14681983	0.05798548	-19.777708	4.63E-87	2.12E-84	SFRP1
2162.865039	0.311920596	0.04819294	6.4723296	9.65E-11	1.14E-09	GPAT4
67.93024421	1.502383353	0.2425812	6.1933214	5.89E-10	6.40E-09	ANK1
2016.939214	-0.3896192	0.05960732	-6.5364323	6.30E-11	7.61E-10	KAT6A
1184.336887	0.660364437	0.06529873	10.112975	4.84E-24	1.98E-22	PLAT
2404.808268	-0.20400979	0.05315767	-3.8378238	0.0001241	0.000581544	VDAC3
973.8159474	0.430040881	0.0638073	6.7396819	1.59E-11	2.01E-10	SLC20A2
52.55079343	-0.60420726	0.24060456	-2.5112045	0.012032	0.035071475	SMIM19
9.9013094	3.390712767	0.86207421	3.9332029	8.38E-05	0.000405919	CHRNA6
2867.965437	-0.37659268	0.05620936	-6.6998215	2.09E-11	2.62E-10	HOOK3
869.1181451	0.36263612	0.07500844	4.834604	1.33E-06	8.93E-06	HGSNAT
731.5100725	-0.47741515	0.07143301	-6.683397	2.33E-11	2.92E-10	SPIDR
15241.15525	-0.72698555	0.11938877	-6.0892291	1.13E-09	1.19E-08	PRKDC
7514.312037	-0.23502361	0.03901761	-6.0235273	1.71E-09	1.74E-08	MCM4
2718.572782	-0.20572605	0.04602463	-4.469912	7.83E-06	4.61E-05	UBE2V2
445.0076005	0.680251195	0.09467278	7.1852877	6.71E-13	9.93E-12	SNAI2
3327.768513	-0.37560983	0.05055587	-7.4295987	1.09E-13	1.76E-12	RB1CC1
79.28777416	0.814282684	0.19861657	4.0997723	4.14E-05	0.000212334	ENSG00000237807
1704.972739	-0.23850094	0.0520951	-4.578184	4.69E-06	2.86E-05	TCEA1
1865.156886	-0.24595304	0.04799013	-5.1250755	2.97E-07	2.21E-06	LYPLA1
434.3844286	0.427016989	0.09358191	4.5630291	5.04E-06	3.06E-05	LYN
7254.400525	-0.21428816	0.03937528	-5.4422008	5.26E-08	4.38E-07	RPS20
280.0059306	-0.69528085	0.10677958	-6.5113653	7.45E-11	8.93E-10	PLAG1
702.2263696	0.567660492	0.07547952	7.5207219	5.45E-14	9.14E-13	CHCHD7
3.102072978	3.408597125	1.43626444	2.3732378	0.0176329	0.048556805	FAM110B
237.0337798	-0.77652292	0.11995767	-6.4733079	9.59E-11	1.14E-09	UBXN2B
3188.811421	0.329859506	0.04610704	7.1542108	8.42E-13	1.23E-11	SDCBP
712.7457056	-0.44651258	0.07121773	-6.269683	3.62E-10	4.04E-09	NSMAF
34.86708561	1.779332252	0.35440367	5.0206372	5.15E-07	3.69E-06	CA8
517.000024	-0.27845838	0.09151496	-3.0427633	0.0023442	0.008273807	CHD7
11317.43166	-0.22094979	0.03758093	-5.8793056	4.12E-09	3.98E-08	ASPH
2156.662794	0.455229551	0.05372685	8.4730365	2.39E-17	5.54E-16	GGH
1981.20221	-0.29810833	0.05231735	-5.698078	1.21E-08	1.11E-07	ARMC1
1067.779813	-0.17682352	0.06541294	-2.7031886	0.0068678	0.021495795	MTFR1
113.5043042	0.624437001	0.16492681	3.7861462	0.000153	0.000705943	TRIM55
277.753395	0.334652953	0.10979226	3.0480559	0.0023033	0.008149314	RRS1
2442.871889	0.139625497	0.0489595	2.851857	0.0043465	0.014288283	MYBL1
942.1097197	-0.17294468	0.06158512	-2.808222	0.0049816	0.016144206	COPS5
2514.449723	-0.16799631	0.04610279	-3.6439513	0.0002685	0.001178133	ARFGEF1
35.64965088	1.232862008	0.31979624	3.8551485	0.0001157	0.000544482	PREX2
89.59957759	0.599356334	0.19944572	3.00511	0.0026548	0.009237256	C8orf34
960.7794262	-0.48191466	0.06786347	-7.1012382	1.24E-12	1.78E-11	NCOA2
536.9016047	0.241679969	0.08191945	2.9502146	0.0031755	0.010830067	LACTB2
2879.19749	-0.40539856	0.04671744	-8.6776709	4.04E-18	1.01E-16	RPL7
338.6564459	0.636966038	0.10475856	6.0803243	1.20E-09	1.25E-08	RDH10
1218.628451	-0.22108005	0.06014803	-3.6755996	0.0002373	0.001053216	STAU2

466.3521505	0.265107445	0.0933835	2.8389109	0.0045268	0.014816619	TMEM70
580.570135	0.277787513	0.08102266	3.4285163	0.0006069	0.002471621	GDAP1
134.9798722	-0.53967816	0.1494505	-3.611083	0.0003049	0.001320034	PCBP2P2
206.2756018	-0.32771834	0.1248411	-2.6250838	0.0086628	0.026263161	LINC01111
134.6912628	-0.55966449	0.14421248	-3.8808325	0.0001041	0.000495292	ZFHX4-AS1
2836.272156	-0.40235199	0.06457651	-6.2306243	4.65E-10	5.10E-09	ZFHX4
466.2991713	-0.45686783	0.08687652	-5.2588181	1.45E-07	1.13E-06	PEX2
22.28717462	1.080896107	0.42039568	2.5711399	0.0101364	0.030180557	HEY1
586.9740449	1.464514363	0.10239355	14.3028	2.10E-46	2.65E-44	PAG1
42.94915885	1.000070382	0.26961745	3.7092199	0.0002079	0.000936379	FABP5
3.601864571	4.142869028	1.39374553	2.9724716	0.0029541	0.010168693	FABP4
966.9931343	0.407823755	0.06792603	6.0039393	1.93E-09	1.96E-08	IMPA1
789.0167319	-0.38126375	0.06726667	-5.6679447	1.45E-08	1.30E-07	ZFAND1
601.2974456	-0.28341778	0.07587598	-3.7352767	0.0001875	0.000852268	RBIS
3101.169499	-0.36820825	0.05374955	-6.8504437	7.36E-12	9.71E-11	CPNE3
174.0598401	0.594668694	0.1448232	4.10617	4.02E-05	0.000206846	RIPK2-DT
392.7270921	0.522263781	0.09404525	5.5533246	2.80E-08	2.44E-07	RIPK2
412.0274885	-0.3512223	0.089044	-3.9443681	8.00E-05	0.000388981	DECR1
589.7278714	0.2155475	0.08912835	2.4183945	0.0155892	0.043767449	TMEM64
228.035646	-0.40142733	0.12204297	-3.2892296	0.0010046	0.003879714	C8orf88
517.4068906	-0.70191709	0.08402873	-8.3532985	6.64E-17	1.47E-15	TRIQK
1071.864532	-0.22156332	0.05998874	-3.6934151	0.0002213	0.000990668	RBM12B
332.8296254	-0.27491422	0.09812637	-2.8016346	0.0050844	0.016446887	TMEM67
2520.003092	-0.40507543	0.04594423	-8.8166775	1.18E-18	3.09E-17	VIRMA
28.15458074	-0.98569488	0.31749756	-3.1045747	0.0019055	0.006885138	ESRP1
1276.415705	-0.4297577	0.05907926	-7.2742572	3.48E-13	5.34E-12	DPY19L4
1108.990361	-0.4005719	0.06374849	-6.2836297	3.31E-10	3.70E-09	INTS8
1188.533897	1.135852554	0.06757476	16.808827	2.10E-63	4.75E-61	TP53INP1
1003.727892	0.801672312	0.07381921	10.859942	1.79E-27	9.57E-26	GDF6
2098.5809	0.130708384	0.05451748	2.3975501	0.0165051	0.045968262	PTDSS1
2460.199586	1.065800559	0.05542995	19.227883	2.16E-82	8.55E-80	SDC2
141.5434679	0.497743023	0.15649478	3.1805727	0.0014698	0.005446529	CPQ
5745.463753	-0.36549676	0.04858056	-7.5235186	5.33E-14	8.96E-13	RPL30
69.74343381	0.710637763	0.22550381	3.1513337	0.0016253	0.005977875	ERICH5
1301.915558	-0.34466311	0.05882733	-5.8588944	4.66E-09	4.47E-08	STK3
6.769606153	1.99706868	0.82310065	2.4262752	0.0152547	0.042966522	KCNS2
221.960289	0.619188942	0.12697386	4.876507	1.08E-06	7.33E-06	OSR2
1175.454328	-0.57201459	0.07284292	-7.8527134	4.07E-15	7.62E-14	VPS13B
492.0570132	0.511500827	0.09055839	5.6482989	1.62E-08	1.45E-07	SPAG1
43022.67056	-0.14317242	0.0369018	-3.8798222	0.0001045	0.000497081	PABPC1
15799.79057	-0.20799629	0.03752016	-5.543588	2.96E-08	2.56E-07	YWHAZ
13.90414605	1.857405252	0.49993823	3.7152695	0.000203	0.000916394	NCALD
4094.471828	-0.54188563	0.05700299	-9.5062672	1.98E-21	6.56E-20	UBR5
1555.294393	0.322732426	0.05764392	5.5987245	2.16E-08	1.90E-07	KLF10
1563.141367	-0.28427988	0.05540757	-5.1307044	2.89E-07	2.15E-06	FZD6
1759.023511	0.257674934	0.05112618	5.0399803	4.66E-07	3.36E-06	LRP12
60.16224647	-0.71374037	0.23287465	-3.0649123	0.0021773	0.007758999	ZFPM2-AS1
1201.572316	-0.25182623	0.06159218	-4.0886072	4.34E-05	0.000222225	OXR1

5087.487461	-0.43958024	0.04358318	-10.086008	6.37E-24	2.60E-22	EIF3E
660.524296	-0.30743187	0.0741403	-4.146623	3.37E-05	0.000176471	EMC2
1596.278548	-0.13860971	0.05728215	-2.4197713	0.0155303	0.043609142	NUDCD1
1522.991081	0.189953376	0.05553486	3.4204347	0.0006252	0.002534957	ENY2
8.735253613	2.030695166	0.75076807	2.7048236	0.0068341	0.021412687	SYBU
4376.937619	-0.29375666	0.04549089	-6.4574837	1.06E-10	1.25E-09	EIF3H
9367.510047	-0.46514864	0.04399027	-10.573898	3.94E-26	1.90E-24	RAD21
168.7579066	-0.44044547	0.13256895	-3.3223878	0.0008925	0.00348936	MED30
4931.656193	0.151745694	0.04344857	3.4925362	0.0004785	0.001996249	EXT1
118.4839221	-0.40802899	0.15793017	-2.5836038	0.0097774	0.029269938	SAMD12
26.90527929	2.645291203	0.46925667	5.6371946	1.73E-08	1.54E-07	CCN3
80.9759119	0.898582422	0.22338097	4.0226453	5.75E-05	0.00028741	ENPP2
1926.680725	-0.17136007	0.05197556	-3.2969355	0.0009775	0.003787437	TAF2
27.72588477	2.297781524	0.54331015	4.2292262	2.34E-05	0.000125783	COL14A1
906.8889876	-0.26777207	0.06901608	-3.8798507	0.0001045	0.000497081	MRPL13
716.5085164	-0.46256795	0.07049186	-6.5620053	5.31E-11	6.46E-10	MTBP
265.2106595	-0.6430894	0.10639732	-6.0442258	1.50E-09	1.55E-08	ZHX2
623.1287288	-0.3193554	0.07483844	-4.2672645	1.98E-05	0.000107875	TBC1D31
8714.102005	-0.31400963	0.03790285	-8.2845911	1.19E-16	2.56E-15	ATAD2
285.9644641	-0.30805628	0.10473107	-2.941403	0.0032673	0.01110598	NTAQ1
114.1265979	1.413325463	0.18724159	7.5481386	4.42E-14	7.48E-13	FBXO32
375.1165073	-0.4217176	0.09665736	-4.3630161	1.28E-05	7.26E-05	TATDN1
1439.130835	-0.53204714	0.05598006	-9.5042268	2.02E-21	6.68E-20	WASHC5
353.9010512	-0.28352253	0.09588155	-2.9570084	0.0031064	0.010629721	NSMCE2
346.1982649	0.629143343	0.10699003	5.8803921	4.09E-09	3.96E-08	TRIB1
615.8238719	0.462362769	0.07901503	5.8515801	4.87E-09	4.67E-08	LRATD2
1514.674517	-0.65161176	0.05716342	-11.399104	4.22E-30	2.65E-28	MYC
344.0442478	0.414043875	0.10586349	3.911111	9.19E-05	0.000442199	PVT1
5250.175812	-0.50401946	0.04076057	-12.365367	4.02E-35	3.30E-33	ASAP1
1762.491803	0.212586367	0.06456456	3.2926168	0.0009926	0.003838393	EFR3A
355.1979392	0.630711828	0.10081809	6.2559388	3.95E-10	4.39E-09	ST3GAL1
9.379042125	1.632668463	0.60660972	2.6914644	0.0071139	0.022130748	ST3GAL1-DT
285.7856742	-0.33172912	0.11430811	-2.9020611	0.0037072	0.012426283	ZFAT
586.6292765	0.299745207	0.08519841	3.5182019	0.0004345	0.001825474	KHDRBS3
144.7602492	-0.7979963	0.14775194	-5.4009191	6.63E-08	5.45E-07	TRAPPC9
990.4683308	-0.42363668	0.06275546	-6.7505951	1.47E-11	1.88E-10	CHRAC1
1413.216641	-0.49034235	0.07132675	-6.8745925	6.22E-12	8.27E-11	AGO2
3758.555651	-0.13275531	0.04517773	-2.9385122	0.0032979	0.011203511	PTK2
915.6616332	-0.16947998	0.06464001	-2.6219052	0.008744	0.026481668	DENND3
614.3544517	-0.50772528	0.08164293	-6.218852	5.01E-10	5.48E-09	JRK
892.2488281	0.20430085	0.06530543	3.1283902	0.0017577	0.006411963	LY6K
43.50344449	1.029238021	0.28921258	3.5587595	0.0003726	0.001587383	LYNX1
1165.357618	0.486380289	0.07199746	6.7555202	1.42E-11	1.82E-10	LY6E
44.0236591	0.711701476	0.27673435	2.5717859	0.0101175	0.030140729	RHPN1
17.1995154	1.716397519	0.57402483	2.9901102	0.0027888	0.009665757	MAFA
294.3428266	0.366805825	0.11169326	3.2840462	0.0010233	0.00393866	GSDMD
62.87665974	0.588305675	0.22407985	2.6254287	0.008654	0.026245716	NAPRT
203.1451785	0.336740106	0.13277707	2.5361315	0.0112085	0.032980576	ZNF707

192.0190089	0.530906892	0.128957	4.1169297	3.84E-05	0.000198482	FAM83H
8.815427786	2.202451318	0.71506154	3.0800864	0.0020694	0.007410083	IQANK1
856.0474226	0.831994019	0.08514348	9.7716708	1.49E-22	5.45E-21	NRBP2
60.68578903	1.945762176	0.28410349	6.8487795	7.45E-12	9.80E-11	EPPK1
96.10265203	1.889403351	0.19721031	9.5806519	9.64E-22	3.26E-20	PARP10
1042.651439	0.50780652	0.06729476	7.5460041	4.49E-14	7.59E-13	GRINA
225.893215	0.342719781	0.13901528	2.465339	0.0136884	0.039177208	SHARPIN
228.5452953	0.3186592	0.1261238	2.5265588	0.0115186	0.03378456	MROH1
6.927193912	2.943407479	1.20454571	2.443583	0.0145422	0.041261094	SCRT1
325.5619311	0.342606684	0.1018427	3.364077	0.000768	0.003046428	SLC52A2
359.2322946	0.54510026	0.09870397	5.5225766	3.34E-08	2.87E-07	KIFC2
12731.656	-0.17343576	0.036052	-4.8107116	1.50E-06	1.00E-05	RPL8
723.4891225	0.169965093	0.06851188	2.4808121	0.0131083	0.037740745	C8orf33
145.1205024	0.465398571	0.15508285	3.0009674	0.0026912	0.009354498	KANK1
159.0690103	-0.89213687	0.14305267	-6.2364222	4.48E-10	4.93E-09	RFX3
1246.27839	0.153887955	0.05746385	2.6779959	0.0074064	0.02291771	AK3
379.4122844	0.643092358	0.09853588	6.5264793	6.73E-11	8.12E-10	PDCD1LG2
3199.060876	0.124786883	0.047071	2.6510351	0.0080246	0.024555153	RIC1
806.1108808	-0.23273306	0.06907724	-3.3691713	0.0007539	0.002997511	KIAA2026
523.5598623	-0.28558353	0.08301748	-3.4400411	0.0005816	0.002379316	KDM4C
35.49178262	1.225674739	0.32271939	3.7979581	0.0001459	0.000674934	LURAP1L
1793.695041	-0.26196252	0.05212319	-5.0258346	5.01E-07	3.60E-06	MPDZ
3137.253787	-0.65138529	0.05457406	-11.935805	7.70E-33	5.72E-31	NFIB
791.460655	-0.29408348	0.06827712	-4.3072038	1.65E-05	9.14E-05	ZDHHC21
1560.969272	-0.18146269	0.05522924	-3.285627	0.0010176	0.003921833	SNAPC3
7008.595528	-0.72407731	0.04219826	-17.158938	5.39E-66	1.30E-63	PSIP1
836.9697909	-0.42362923	0.06504526	-6.5128378	7.37E-11	8.84E-10	CNTLN
333.8326798	-0.63283886	0.10482428	-6.0371401	1.57E-09	1.61E-08	ADAMTSL1
1579.296318	-0.20575513	0.061496	-3.3458295	0.0008204	0.003235701	RRAGA
2356.818935	-0.19690115	0.04864701	-4.0475491	5.18E-05	0.000260714	HAUS6
1313.594871	0.582787211	0.06249629	9.3251487	1.11E-20	3.45E-19	PLIN2
1483.401616	-0.22414733	0.0546226	-4.1035643	4.07E-05	0.000209005	DENND4C
20948.12253	-0.36744975	0.03607804	-10.184859	2.32E-24	9.76E-23	RPS6
135.2480051	1.560238159	0.18154526	8.5942103	8.38E-18	2.03E-16	ACER2
102.6440758	1.34875934	0.19579834	6.8885128	5.64E-12	7.57E-11	ENSG00000260912
486.0675543	0.528882916	0.09385042	5.6353816	1.75E-08	1.56E-07	MLLT3
869.6228241	-0.41421473	0.06492002	-6.3803852	1.77E-10	2.05E-09	FOCAD
1621.441221	-0.29279602	0.05324238	-5.4993039	3.81E-08	3.24E-07	KLHL9
1638.498469	-0.25346821	0.05131582	-4.9393775	7.84E-07	5.45E-06	MTAP
1469.973325	-0.2793826	0.05266533	-5.3048675	1.13E-07	8.95E-07	CDKN2A
204.0873026	-0.56468944	0.13282317	-4.2514378	2.12E-05	0.000114817	CDKN2B-AS1
1067.805008	-0.3655243	0.08079262	-4.524229	6.06E-06	3.61E-05	CDKN2B
194.4319272	-0.56168846	0.12791437	-4.3911288	1.13E-05	6.46E-05	IFT74
3.174504505	3.727036726	1.41931665	2.6259374	0.0086411	0.026211094	ENSG00000254396
5.428154509	-2.16951382	0.80988778	-2.6787832	0.007389	0.022876103	LINGO2
3259.817036	-0.32845039	0.03926931	-8.3640479	6.06E-17	1.35E-15	ACO1
27.26082715	3.018262896	0.45411982	6.646402	3.00E-11	3.73E-10	DDX58
8631.946528	0.145984465	0.04676833	3.1214386	0.0017997	0.006547591	DNAJA1

2310.703855	-0.18615196	0.04443902	-4.1889301	2.80E-05	0.000148551	SMU1
2349.566204	0.301339364	0.04461522	6.754183	1.44E-11	1.84E-10	B4GALT1
1200.876481	-0.24313709	0.05587225	-4.3516612	1.35E-05	7.60E-05	NFX1
1051.403706	0.355605848	0.07316515	4.8603171	1.17E-06	7.91E-06	NOL6
334.6418074	-0.53473712	0.10706001	-4.994742	5.89E-07	4.18E-06	ANKRD18B
24.81078669	-1.09662029	0.37022559	-2.9620326	0.0030562	0.010482548	CYP4F26P
2175.425934	-0.44323378	0.04726671	-9.3772934	6.77E-21	2.17E-19	UBE2R2
2094.490198	0.237036581	0.0460824	5.1437548	2.69E-07	2.02E-06	UBAP2
479.0318481	-0.33014835	0.08346173	-3.9556855	7.63E-05	0.000372684	KIF24
437.8379879	0.268059043	0.09671148	2.7717396	0.0055758	0.017856815	MYORG
66.77811368	0.565936976	0.2370503	2.3874131	0.0169674	0.047022047	GALT
26.83675568	1.166522081	0.37098783	3.1443675	0.0016645	0.006106541	IL11RA
840.6490058	-0.21528435	0.072121	-2.9850438	0.0028354	0.009804577	FANCG
341.2169198	-0.30805604	0.09377722	-3.2849773	0.0010199	0.003927406	PIGO
1207.647358	-0.34755912	0.05552792	-6.259178	3.87E-10	4.31E-09	STOML2
460.7881713	0.420111728	0.08570181	4.902017	9.49E-07	6.48E-06	FAM214B
1032.547261	0.601735165	0.06587038	9.1351408	6.53E-20	1.91E-18	RUSC2
16691.89361	0.56318779	0.03867757	14.561095	4.97E-48	6.75E-46	TPM2
10274.34701	-0.18282858	0.03844145	-4.7560266	1.97E-06	1.29E-05	TLN1
818.9545544	0.31268381	0.07000028	4.4668939	7.94E-06	4.67E-05	CREB3
629.2706495	0.311532701	0.08019439	3.8847195	0.0001024	0.000488102	GBA2
1162.78531	-0.36008328	0.06666631	-5.401278	6.62E-08	5.44E-07	RGP1
463.6985517	0.47753505	0.09700004	4.9230398	8.52E-07	5.88E-06	RECK
567.5593043	0.94815417	0.09449096	10.034337	1.08E-23	4.29E-22	GLIPR2
2912.645002	0.200219124	0.04705964	4.2545829	2.09E-05	0.000113498	CLTA
1604.981269	-0.23016072	0.05518578	-4.1706529	3.04E-05	0.000160007	GNE
2948.187973	-0.15365052	0.04264924	-3.6026551	0.000315	0.001358185	MELK
1902.051596	-0.2487794	0.04927923	-5.0483618	4.46E-07	3.22E-06	EBLN3P
606.5476484	-0.39086231	0.07625094	-5.1259996	2.96E-07	2.20E-06	ZCCHC7
1009.754224	-0.30711321	0.06137447	-5.0039247	5.62E-07	4.00E-06	POLR1E
558.5548209	-0.19686888	0.07850708	-2.5076575	0.0121534	0.035366185	EXOSC3
805.1029832	-0.39776643	0.08687895	-4.5783981	4.69E-06	2.86E-05	DCAF10
295.2787484	0.637249375	0.11157631	5.711332	1.12E-08	1.03E-07	SHB
907.730376	0.284491844	0.07142749	3.9829462	6.81E-05	0.000335888	IGFBPL1
7.420072848	1.723400059	0.68446608	2.517875	0.0118065	0.034506788	TCEA1P3
62.78812789	0.843979041	0.23276304	3.6259151	0.0002879	0.001252758	CNTNAP3
250.2041295	-0.59495657	0.13674435	-4.3508676	1.36E-05	7.62E-05	PGM5P2
42.62278292	1.020867345	0.28851751	3.5383202	0.0004027	0.001705047	CNTNAP3B
31.7025337	1.183379851	0.35145186	3.3671179	0.0007596	0.003015784	LINC01410
6.0756789	-1.86972797	0.72129625	-2.5921776	0.0095371	0.028619387	ENSG00000233178
61.87024342	0.607919101	0.23906592	2.5428932	0.0109939	0.032445073	TJP2
2764.776466	-0.4340337	0.05633987	-7.7038467	1.32E-14	2.36E-13	PTAR1
6213.685446	1.148037078	0.08511859	13.487501	1.85E-41	1.98E-39	MAMDC2
76.73682691	1.412494601	0.22456915	6.2897981	3.18E-10	3.57E-09	RPL24P8
2590.390028	-0.32622882	0.04833923	-6.7487382	1.49E-11	1.90E-10	SMC5
6118.179594	0.174595477	0.04029984	4.3324108	1.47E-05	8.24E-05	CEMIP2
562.1125471	0.228084813	0.07738495	2.9474054	0.0032045	0.010918249	ABHD17B
3533.371496	0.246757075	0.04224579	5.8409858	5.19E-09	4.94E-08	ZFAND5

5546.365704	0.470139749	0.05548541	8.4732145	2.39E-17	5.53E-16	ANXA1
464.7371372	-0.53510475	0.0908599	-5.8893389	3.88E-09	3.76E-08	CARNMT1
62.26971346	0.683038458	0.2451871	2.7857846	0.0053398	0.017183515	NMRK1
942.5601016	0.505756364	0.06999307	7.2258058	4.98E-13	7.49E-12	OSTF1
22.01808174	2.185768284	0.52629738	4.153105	3.28E-05	0.000171851	PCSK5
641.4150651	0.215776012	0.07867243	2.7427145	0.0060934	0.019335873	RFK
967.8331833	0.248891828	0.0658232	3.7812172	0.0001561	0.000718545	GCNT1
1140.861548	0.473547468	0.07758173	6.1038526	1.04E-09	1.09E-08	PRUNE2
2633.280423	-0.29848356	0.1233157	-2.420483	0.0154999	0.043530903	VPS13A
4.42931892	3.723396943	1.37671939	2.7045431	0.0068398	0.021419918	ENSG00000234819
2822.467224	-0.43054412	0.04863701	-8.8521911	8.58E-19	2.29E-17	PSAT1
786.8508804	0.53324995	0.07503576	7.1066105	1.19E-12	1.72E-11	TLE1
178.6932651	0.474207437	0.14841498	3.1951453	0.0013976	0.005207701	RASEF
5779.757859	-0.2101018	0.03862903	-5.4389611	5.36E-08	4.45E-07	UBQLN1
85.84081488	-0.73315754	0.21230951	-3.4532488	0.0005539	0.002274366	GKAP1
115.3020143	-0.62774586	0.16452407	-3.8155259	0.0001359	0.000632882	KIF27
13693.56073	-0.35513141	0.03532107	-10.05438	8.79E-24	3.53E-22	HNRNPK
1012.080236	-0.28561904	0.06436351	-4.4375926	9.10E-06	5.30E-05	RMI1
841.9731551	-0.24626368	0.07250148	-3.396671	0.0006821	0.002737567	AGTPBP1
1306.992676	-0.27490121	0.0588979	-4.6674195	3.05E-06	1.93E-05	NAA35
663.8171625	0.319823354	0.08010349	3.9926268	6.53E-05	0.000323196	ISCA1
3011.627644	-0.39641858	0.04497464	-8.8142688	1.20E-18	3.15E-17	SPIN1
405.4289266	0.850620847	0.10101638	8.4206227	3.74E-17	8.50E-16	S1PR3
71.81662591	0.525657626	0.21557616	2.4383847	0.0147531	0.041777522	SHC3
14.45123816	1.529420507	0.50113519	3.051912	0.0022739	0.008060111	ENSG00000224945
1776.351483	-0.33551832	0.05599914	-5.9914906	2.08E-09	2.10E-08	CKS2
548.1018909	-0.40964913	0.08641685	-4.7403849	2.13E-06	1.38E-05	SECISBP2
174.7323725	-0.5425149	0.13189696	-4.1131721	3.90E-05	0.000201443	SEMA4D
116.0617122	-0.54548783	0.16353443	-3.3356147	0.0008511	0.003343305	AUH
6354.967939	-0.49890167	0.0392007	-12.726858	4.19E-37	3.76E-35	IARS1
337.818443	-0.39521287	0.10133815	-3.8999415	9.62E-05	0.000461317	CENPP
294.2695008	0.567849228	0.11195261	5.0722286	3.93E-07	2.86E-06	NINJ1
4856.566625	-0.52342965	0.03692926	-14.173848	1.33E-45	1.61E-43	FAM120A
816.082075	-0.58083949	0.07363508	-7.8880817	3.07E-15	5.80E-14	PHF2
103.887284	0.572521892	0.18370518	3.1165257	0.00183	0.006647788	MIRLET7A1HG
2516.364521	0.120890743	0.04759148	2.5401761	0.0110797	0.032664748	MFSD14B
568.1690758	0.328339798	0.08040267	4.0836928	4.43E-05	0.000226513	AOPEP
477.6057673	-0.29793304	0.09724549	-3.0637208	0.002186	0.007786774	FANCC
163.7018784	-0.75338253	0.14432888	-5.2199014	1.79E-07	1.38E-06	PTCH1
1209.197831	-0.28322907	0.06673754	-4.2439242	2.20E-05	0.000118401	ERCC6L2
1308.651837	-0.48447413	0.05818951	-8.3257983	8.38E-17	1.84E-15	ZNF367
664.6768216	-0.45879502	0.07197385	-6.3744684	1.84E-10	2.12E-09	CDC14B
620.20382	-0.39480115	0.0780774	-5.0565357	4.27E-07	3.10E-06	ZNF510
643.1708023	-0.91587731	0.07409954	-12.360095	4.30E-35	3.51E-33	MFSD14C
964.0719657	0.273663498	0.06914918	3.9575812	7.57E-05	0.000370155	CTSV
310.6911407	0.540207673	0.10775985	5.0130699	5.36E-07	3.83E-06	TDRD7
2850.407788	-0.34724213	0.04505141	-7.7076861	1.28E-14	2.30E-13	NCBP1
5772.874403	-0.69255653	0.03667581	-18.883194	1.57E-79	5.45E-77	ANP32B

1168.77329	0.567830869	0.07248567	7.8336985	4.74E-15	8.75E-14	TRIM14
22.73719862	0.98840407	0.37828106	2.6128828	0.0089782	0.027106086	GABBR2
26.98401682	1.254358602	0.39481019	3.1771181	0.0014875	0.005505962	COL15A1
745.6601453	-0.36055003	0.07267989	-4.9607949	7.02E-07	4.91E-06	STX17
2024.060014	0.191444886	0.04885006	3.9190309	8.89E-05	0.000428868	ERP44
6.463577317	2.245280407	0.86641791	2.591452	0.0095572	0.028674861	UPF3AP3
288.4059244	-0.47686819	0.11563057	-4.1240666	3.72E-05	0.000193002	INVS
1268.185992	-0.25757028	0.06578583	-3.9152853	9.03E-05	0.000435099	TEX10
986.5859464	-0.31169756	0.07174889	-4.344284	1.40E-05	7.84E-05	MSANTD3
489.8927533	-0.32624696	0.08128501	-4.0136176	5.98E-05	0.000297777	ZNF189
5286.918946	-0.43844311	0.03525952	-12.434744	1.69E-35	1.42E-33	SMC2
541.3221575	0.266850256	0.08961516	2.9777356	0.0029039	0.01001352	ABCA1
1863.745893	-0.53562537	0.05462663	-9.8052053	1.07E-22	3.99E-21	SLC44A1
1378.820708	-0.5495849	0.0656613	-8.3699971	5.76E-17	1.29E-15	FKTN
700.769662	-0.20261606	0.08575645	-2.3626918	0.0181428	0.049803068	TMEM38B
4.719099344	2.262317073	0.92579851	2.4436387	0.01454	0.041261094	ENSG00000226535
6710.719783	-0.12950931	0.04205517	-3.0795099	0.0020734	0.007422124	RAD23B
77.11895742	1.869132209	0.24726418	7.559252	4.05E-14	6.90E-13	KLF4
1534.844201	-0.39793665	0.05244794	-7.587269	3.27E-14	5.61E-13	ELP1
371.3519321	-0.26760748	0.09991705	-2.6782966	0.0073998	0.022905295	ABITRAM
5537.57591	-0.57911682	0.05039226	-11.492178	1.44E-30	9.30E-29	CTNNAL1
4705.171364	-0.26081937	0.04276988	-6.0982013	1.07E-09	1.12E-08	TMEM245
76.38858858	1.199928652	0.22809041	5.2607588	1.43E-07	1.12E-06	FRRS1L
542.2590394	-0.29918603	0.08167532	-3.6631143	0.0002492	0.001101148	PTPN3
162.5418801	0.385368455	0.15047237	2.5610579	0.0104354	0.030978417	SVEP1
1076.742172	-0.41010403	0.07275907	-5.6364659	1.74E-08	1.55E-07	LPAR1
2614.140336	-0.66719129	0.04686255	-14.237195	5.38E-46	6.59E-44	ECPAS
961.1560689	-0.17268271	0.06355188	-2.7171926	0.0065838	0.020707681	PTGR1
3072.264029	-0.32836088	0.04589013	-7.1553707	8.34E-13	1.23E-11	UGCG
280.2239229	0.860881002	0.12060837	7.1378211	9.48E-13	1.39E-11	SUSD1
2798.669008	-0.22373588	0.0548228	-4.0810739	4.48E-05	0.000228883	PTBP3
584.8731922	-0.72201609	0.07744698	-9.3227147	1.13E-20	3.53E-19	KIAA1958
113.4718299	-0.79109709	0.16403929	-4.8226074	1.42E-06	9.45E-06	ZFP37
1559.943475	-0.13562641	0.05471475	-2.4787905	0.0131829	0.037936474	FKBP15
468.3879265	-0.69593423	0.09092449	-7.6539799	1.95E-14	3.42E-13	ZNF618
738.291414	0.372864539	0.08036104	4.639867	3.49E-06	2.18E-05	COL27A1
54.11748766	0.660623836	0.25206253	2.6208728	0.0087705	0.026545258	WHRN
1104.840458	0.367908372	0.06120693	6.0108942	1.85E-09	1.88E-08	ATP6V1G1
11.86590389	1.634038231	0.55545364	2.9418085	0.003263	0.011093618	ENSG00000228714
142.0179821	0.434497162	0.15416705	2.818353	0.0048271	0.015701973	ASTN2
472.6464868	1.934489449	0.10720925	18.044053	8.79E-73	2.35E-70	TLR4
6.933979036	2.433742126	1.02436798	2.3758475	0.0175087	0.048279147	BRINP1
2758.629497	-0.56952789	0.04961227	-11.479578	1.67E-30	1.07E-28	CDK5RAP2
782.6894404	-0.19679195	0.07589971	-2.5927893	0.0095201	0.028573469	MEGF9
2114.173817	-0.31167098	0.04750081	-6.5613824	5.33E-11	6.48E-10	FBXW2
1836.145448	-0.19131968	0.055907	-3.4221062	0.0006214	0.002521775	PSMD5
1520.99739	-0.4186064	0.05266179	-7.948959	1.88E-15	3.65E-14	PHF19
26.11816107	2.551744591	0.44150608	5.7796364	7.49E-09	7.03E-08	TRAF1

70.16470592	0.695893774	0.2381949	2.921531	0.0034832	0.011752442	C5
661.4906306	-0.5545124	0.07316756	-7.578665	3.49E-14	5.99E-13	CNTRL
683.5174472	0.559726593	0.08540012	6.5541661	5.60E-11	6.78E-10	GSN
2254.726247	0.739270108	0.05454454	13.553512	7.55E-42	8.21E-40	STOM
57.53816155	0.943307032	0.2317174	4.0709374	4.68E-05	0.000238441	PTGS1
814.7762023	-0.32738644	0.06822689	-4.7984954	1.60E-06	1.06E-05	PDCL
330.6495489	-0.43250983	0.10037642	-4.308879	1.64E-05	9.08E-05	ZBTB26
2252.469767	-0.28402251	0.05018563	-5.6594391	1.52E-08	1.36E-07	RABGAP1
48.44538445	-1.04195352	0.26277659	-3.9651688	7.33E-05	0.000359485	MIR600HG
891.143129	-0.51688879	0.0661935	-7.8087543	5.78E-15	1.06E-13	STRBP
385.2763978	-0.26970268	0.09369861	-2.8784065	0.0039969	0.013287336	DENND1A
1235.473926	-0.63838479	0.05937519	-10.75171	5.82E-27	3.02E-25	NEK6
2180.529247	-0.12941534	0.04836373	-2.675876	0.0074534	0.023042667	PSMB7
4618.968508	-0.20840264	0.04677009	-4.4558956	8.35E-06	4.89E-05	RPL35
1043.763847	0.246157764	0.06395886	3.848689	0.0001188	0.000557982	ARPC5L
649.0266433	-0.62551181	0.07557565	-8.2766319	1.27E-16	2.73E-15	SCAI
2936.233491	-0.14964679	0.04704883	-3.1806696	0.0014694	0.005445868	PPP6C
19180.81268	-0.11508402	0.04359813	-2.6396548	0.0082991	0.025319389	HSPA5
2223.898903	-0.21395584	0.04783364	-4.4729156	7.72E-06	4.54E-05	GAPVD1
2100.420687	-0.30256757	0.05235235	-5.7794456	7.49E-09	7.03E-08	MAPKAP1
1075.41525	-0.48852816	0.06197785	-7.8823023	3.21E-15	6.05E-14	PBX3
135.7016189	0.386166297	0.16184014	2.3860971	0.0170283	0.047130564	ANGPTL2
186.7708655	0.651603896	0.13633303	4.7795012	1.76E-06	1.16E-05	ZNF79
5133.150756	-0.25035052	0.04470447	-5.6001234	2.14E-08	1.89E-07	RPL12
2655.36215	0.34456598	0.05510027	6.2534359	4.02E-10	4.46E-09	NIBAN2
1320.733352	0.324708511	0.06431713	5.0485539	4.45E-07	3.22E-06	STXBP1
681.0774868	-0.23175555	0.07334496	-3.1598019	0.0015788	0.005822845	CDK9
683.4445462	0.329428001	0.07161858	4.5997559	4.23E-06	2.61E-05	ENG
358.7974277	0.436287626	0.10003515	4.3613434	1.29E-05	7.31E-05	FAM102A
656.5531332	0.514395468	0.07542315	6.8201267	9.10E-12	1.19E-10	SLC25A25
43.9663476	0.865477535	0.27231053	3.1782742	0.0014815	0.005486388	SLC25A25-AS1
247.5293363	0.573172589	0.1285921	4.4572923	8.30E-06	4.86E-05	BBLN
1788.114823	-0.27005337	0.049664	-5.4376078	5.40E-08	4.48E-07	CIZ1
2367.305295	-0.23553896	0.05412654	-4.3516351	1.35E-05	7.60E-05	GOLGA2
1890.081246	-0.2316121	0.05280197	-4.3864293	1.15E-05	6.58E-05	ODF2
1382.421993	-0.35298217	0.05568505	-6.3389039	2.31E-10	2.65E-09	GLE1
16164.8124	0.25969219	0.03793846	6.845091	7.64E-12	1.00E-10	SPTAN1
409.9322301	-0.55231427	0.09668431	-5.7125535	1.11E-08	1.02E-07	DYNC2I2
9850.841646	-0.47921857	0.03963754	-12.090017	1.19E-33	9.22E-32	SET
266.5140075	-0.42825322	0.10529124	-4.0673207	4.76E-05	0.000241887	PKN3
899.587524	-0.26214564	0.06774535	-3.8695742	0.000109	0.000516047	ZER1
1579.267485	0.222531825	0.0536693	4.1463524	3.38E-05	0.000176626	LRRC8A
2716.438267	-0.50204717	0.04697783	-10.686896	1.17E-26	5.94E-25	NUP188
685.5393658	0.265436337	0.0789775	3.360911	0.0007769	0.003076544	SH3GLB2
362.1235697	0.341208382	0.10410854	3.2774292	0.0010476	0.004022354	MIGA2
1101.418571	-0.34641979	0.06018526	-5.7558905	8.62E-09	8.02E-08	PTPA
14.03301234	2.131739447	0.63438328	3.3603336	0.0007785	0.003080983	PTGES
1269.489038	0.185655785	0.05809288	3.1958443	0.0013942	0.00519733	TOR1B

1004.293963	0.282802331	0.06288296	4.4972807	6.88E-06	4.07E-05	TOR1A
1421.771094	-0.17767511	0.0643085	-2.7628558	0.0057298	0.018306329	NCS1
156.2305211	0.454507533	0.14787801	3.0735303	0.0021154	0.007560047	ASS1
2168.751596	-0.14141903	0.04917495	-2.8758346	0.0040296	0.013381161	FUBP3
1231.949144	-0.19919783	0.05527059	-3.6040473	0.0003133	0.001352268	EXOSC2
2667.414761	-0.17315615	0.04812676	-3.5979185	0.0003208	0.001382126	ABL1
1864.168373	-0.36722057	0.04998112	-7.3471852	2.02E-13	3.20E-12	NUP214
5081.690175	-0.17617243	0.04663447	-3.7777299	0.0001583	0.000728485	PRRC2B
1283.078945	-0.16716526	0.05785571	-2.8893478	0.0038604	0.012882878	RAPGEF1
3632.078487	-0.47817904	0.06025651	-7.9357238	2.09E-15	4.03E-14	SETX
816.2477246	-0.18272058	0.06666944	-2.7406945	0.0061309	0.019434415	TTF1
984.1430548	-0.20210094	0.06155035	-3.2835058	0.0010252	0.003944473	TSC1
15.06202837	1.695915404	0.52054634	3.2579528	0.0011222	0.004278644	CEL
73.49156729	0.66440128	0.21642123	3.069945	0.002141	0.007645109	RALGDS
668.2432058	-0.29926564	0.07249826	-4.1279012	3.66E-05	0.000190039	MED22
6916.0001	-0.45124144	0.03978659	-11.341546	8.17E-30	5.02E-28	RPL7A
223.4194075	0.800145888	0.12543056	6.3791943	1.78E-10	2.06E-09	SLC2A6
456.2823906	-0.50866728	0.08596604	-5.9170722	3.28E-09	3.21E-08	BRD3OS
1435.489162	-0.30571881	0.06259779	-4.8838592	1.04E-06	7.07E-06	BRD3
1399.768457	-0.18316206	0.05444804	-3.3639791	0.0007683	0.003046729	WDR5
6266.140018	0.75408951	0.11857349	6.3596806	2.02E-10	2.32E-09	COL5A1
456.6107646	-0.39208741	0.09084125	-4.3161823	1.59E-05	8.80E-05	PPP1R26
1463.389729	-0.31095722	0.05368487	-5.7922687	6.94E-09	6.53E-08	CAMSAP1
825.4533779	0.168870809	0.07114269	2.3736918	0.0176112	0.048510762	NACC2
323.3992554	0.354381217	0.11046801	3.2079985	0.0013366	0.00500083	SNAPC4
3097.891623	-0.16598877	0.04304249	-3.8563927	0.0001151	0.000542013	SEC16A
55.92352803	0.850043226	0.24282859	3.5005896	0.0004642	0.001940616	CCDC183-AS1
592.807977	0.26314285	0.09525604	2.7624795	0.0057364	0.018317348	PHPT1
34.7458063	0.93703189	0.32782776	2.8583055	0.0042591	0.014040906	CLIC3
97.24038488	0.431470204	0.1811744	2.3815186	0.0172414	0.047667452	NPDC1
462.7359241	0.283820252	0.08792271	3.2280655	0.0012463	0.004699314	UAP1L1
1043.885629	0.233812136	0.06781834	3.447624	0.0005655	0.00231624	MAN1B1
327.5345418	0.266172297	0.10028679	2.6541112	0.0079518	0.024349556	NDOR1
754.7311562	-0.31289102	0.06893797	-4.5387329	5.66E-06	3.40E-05	NELFB
157.8544439	0.390044923	0.14817711	2.6322886	0.0084812	0.025811634	ARRDC1
965.5261742	-0.2038179	0.0636683	-3.2012462	0.0013683	0.005107433	EHMT1
1114.455304	-0.26926171	0.05910039	-4.5560055	5.21E-06	3.15E-05	ZMYND11
1474.680351	-0.16354287	0.05358092	-3.0522593	0.0022713	0.008054073	IDI1
33.99734147	0.929614418	0.33507456	2.7743509	0.0055312	0.017733691	LINC00702
2210.026617	-0.18649337	0.05352692	-3.4841043	0.0004938	0.002052331	TASOR2
67.26398623	-1.27626829	0.20886298	-6.1105529	9.93E-10	1.05E-08	LINC00707
1420.270464	-0.15757103	0.05839642	-2.6982997	0.0069695	0.021770974	ATP5F1C
647.5956866	0.301755642	0.0756116	3.9908643	6.58E-05	0.000325515	TAF3
266.9538329	-0.26434154	0.11005753	-2.4018487	0.0163125	0.045489668	USP6NL
148.9618301	0.804149433	0.1543681	5.2092981	1.90E-07	1.46E-06	PROSER2
1031.974394	-0.38876803	0.06374225	-6.0990636	1.07E-09	1.12E-08	UPF2
737.8982935	-0.23473631	0.07320673	-3.2064855	0.0013437	0.00502504	DHTKD1
190.156128	0.392236327	0.1302398	3.0116471	0.0025983	0.009066053	SEC61A2

1236.998856	0.672754675	0.05918555	11.366873	6.11E-30	3.80E-28	OPTN
1074.931709	-0.16019434	0.06024597	-2.6590051	0.0078372	0.024057991	MCM10
856.1974062	-0.35416725	0.06547779	-5.4089675	6.34E-08	5.23E-07	SEPHS1
303.4855572	-0.65670526	0.10646136	-6.1684843	6.89E-10	7.42E-09	BEND7
379.0822598	-0.58694443	0.08964911	-6.5471303	5.87E-11	7.10E-10	FRMD4A
976.1996719	1.052729377	0.07072788	14.88422	4.17E-50	6.10E-48	FAM107B
450.8449438	-0.28596457	0.08697876	-3.2877518	0.0010099	0.003895809	SUV39H2
1111.883166	-0.20026121	0.0601555	-3.329059	0.0008714	0.003416829	NMT2
435.583749	-0.42735052	0.08912983	-4.794697	1.63E-06	1.08E-05	FAM171A1
1667.321288	0.474041398	0.05464971	8.6741803	4.17E-18	1.03E-16	RSU1
32118.09921	0.161218138	0.03484617	4.6265675	3.72E-06	2.31E-05	VIM
246.132271	0.531925681	0.11792801	4.5105965	6.46E-06	3.84E-05	HACD1
381.9004202	-0.46497802	0.09511923	-4.8883703	1.02E-06	6.92E-06	MLLT10
519.1937604	0.456771062	0.09260015	4.9327246	8.11E-07	5.61E-06	DNAJC1
423.0209764	-0.37080625	0.0895254	-4.1419111	3.44E-05	0.000179542	BMI1
547.4000207	0.326176599	0.08104757	4.0245082	5.71E-05	0.000285226	PIP4K2A
144.6240106	0.412143158	0.15392084	2.6776306	0.0074145	0.022930473	KIAA1217
1301.20356	-0.26153953	0.05727748	-4.5661846	4.97E-06	3.02E-05	ARHGAP21
99.81855117	0.716916272	0.17904702	4.004067	6.23E-05	0.000309092	APBB1IP
3748.735081	-0.13629631	0.0410196	-3.3227115	0.0008915	0.003486097	YME1L1
1549.471863	0.195567845	0.0557287	3.5092841	0.0004493	0.001881884	RAB18
335.4564739	-0.53275736	0.10045088	-5.3036607	1.14E-07	9.00E-07	MPP7
2763.034262	-0.20342484	0.04619073	-4.404019	1.06E-05	6.11E-05	WAC
227.3322	1.695488719	0.14421441	11.756722	6.52E-32	4.59E-30	BAMBI
390.1734172	0.303859395	0.0969892	3.1329198	0.0017308	0.006329768	SVIL
454.7019705	0.501465261	0.09398982	5.3353146	9.54E-08	7.68E-07	JCAD
65.95725098	0.712877106	0.23649574	3.0143338	0.0025754	0.008995169	MAP3K8
21698.77107	0.106500329	0.03949574	2.6965014	0.0070072	0.021865311	ITGB1
1355.042028	-0.43141545	0.05342281	-8.0754908	6.72E-16	1.37E-14	PARD3
1178.972283	-0.28005403	0.06175591	-4.5348541	5.76E-06	3.46E-05	CUL2
6.041294286	2.618167539	1.05523631	2.4811197	0.013097	0.037719919	FZD8
659.4913209	-0.50142042	0.07494682	-6.690349	2.23E-11	2.79E-10	ZNF248
202.1141535	-0.50851753	0.12385125	-4.1058734	4.03E-05	0.000207051	ZNF25
1208.061311	-0.26098205	0.06616507	-3.9444085	8.00E-05	0.000388981	ZNF33A
1627.388471	-0.55701868	0.06715513	-8.2945063	1.09E-16	2.37E-15	ZNF37A
14.02995744	-1.53383261	0.45822798	-3.3473133	0.000816	0.00321989	ENSG00000272983
717.3546466	-0.66869184	0.08013871	-8.3441807	7.17E-17	1.59E-15	ZNF37BP
2719.521942	-0.14671483	0.04441804	-3.3030461	0.0009564	0.003714138	BMS1
1095.76476	0.520948729	0.06277469	8.2987059	1.05E-16	2.29E-15	CSGALNACT2
5243.417786	-0.27631674	0.03881142	-7.1194692	1.08E-12	1.57E-11	HNRNPF
1036.61032	-0.51428278	0.06346853	-8.1029567	5.36E-16	1.10E-14	ZNF22
325.6001268	-0.81350084	0.10314801	-7.8867335	3.10E-15	5.86E-14	MARCHF8
615.2943987	-0.20375855	0.07426553	-2.7436492	0.006076	0.019299065	WASHC2C
5023.653034	0.151386345	0.04163933	3.6356578	0.0002773	0.001210282	NCOA4
4.768730121	2.685680132	1.08623847	2.472459	0.0134187	0.038525895	ANXA8L1
6.555346508	2.400446278	0.92977641	2.5817457	0.0098302	0.029397616	ANXA8
19.35667829	2.492392489	0.52530938	4.7446183	2.09E-06	1.35E-05	ENSG00000276850
22.37480763	1.62450417	0.40017598	4.0594744	4.92E-05	0.000249213	DRGX

50.77358432	0.706156579	0.27406368	2.576615	0.0099773	0.029776092	TIMM23B
546.9662812	0.436302496	0.08555729	5.0995364	3.40E-07	2.51E-06	SGMS1
867.1581112	-0.23539441	0.06519145	-3.6108174	0.0003052	0.001320729	CSTF2T
1660.787709	0.182170974	0.06182003	2.9467954	0.0032109	0.010935345	DKK1
2234.46662	-0.19218153	0.0453714	-4.2357418	2.28E-05	0.000122527	ZWINT
2400.982869	-0.18856241	0.04754637	-3.9658637	7.31E-05	0.00035854	TFAM
2826.870261	-0.2211676	0.05187619	-4.2633742	2.01E-05	0.000109634	BICC1
35.2067579	0.981247337	0.31858112	3.0800549	0.0020696	0.007410083	FAM13C
1967.776787	-0.33979546	0.05439533	-6.2467768	4.19E-10	4.64E-09	CCDC6
17.29808245	-1.22154537	0.45763832	-2.6692375	0.0076024	0.023424019	ANK3
3441.389728	0.230215587	0.05175108	4.4485179	8.65E-06	5.05E-05	ARID5B
45.79731161	0.852515917	0.27857247	3.0603021	0.0022111	0.007871381	ZNF365
546.4926587	0.372308399	0.08120439	4.5848309	4.54E-06	2.79E-05	NRBF2
2208.280051	-0.18771914	0.04938923	-3.8008115	0.0001442	0.000667835	JMJD1C
4620.974467	0.120642918	0.0447094	2.698379	0.0069678	0.021769698	REEP3
175.9356975	-0.6846071	0.13673591	-5.0067837	5.53E-07	3.94E-06	LINC01515
1602.933142	0.185481517	0.0521615	3.5559086	0.0003767	0.001603127	SIRT1
1864.592518	-0.37249467	0.05324873	-6.9953718	2.65E-12	3.66E-11	HERC4
233.3172117	0.61667257	0.12745213	4.8384643	1.31E-06	8.77E-06	PBLD
5162.756	-0.49570016	0.04168402	-11.89185	1.30E-32	9.57E-31	HNRNPH3
836.4256827	-0.15658023	0.06513852	-2.403804	0.0162255	0.045263996	RUFY2
702.7614569	-0.18321296	0.0694182	-2.6392642	0.0083086	0.02534307	DNA2
220.7813097	-0.75055193	0.12729796	-5.8960248	3.72E-09	3.62E-08	TET1
2437.258763	-0.19362266	0.04865418	-3.979569	6.90E-05	0.000340406	CCAR1
1750.239846	-0.38744749	0.05456012	-7.1012943	1.24E-12	1.78E-11	DDX50
6315.226353	0.237125151	0.04774017	4.966994	6.80E-07	4.77E-06	SRGN
2812.581733	-0.21489299	0.04781601	-4.4941636	6.98E-06	4.13E-05	VPS26A
62.14527395	1.354914862	0.25406512	5.3329433	9.66E-08	7.76E-07	HKDC1
195.2565865	0.42598755	0.13391599	3.1810059	0.0014676	0.005440711	TSPAN15
124.494661	-0.73359938	0.16122969	-4.5500267	5.36E-06	3.24E-05	COL13A1
218.6416905	-0.66418072	0.13576151	-4.8922608	9.97E-07	6.80E-06	MACROH2A2
50.87269972	0.803999943	0.25595299	3.1412016	0.0016826	0.006166438	AIFM2
4458.232583	0.196138496	0.0412332	4.7568099	1.97E-06	1.28E-05	SAR1A
1764.222429	-0.21623835	0.05818291	-3.7165268	0.000202	0.000912084	PPA1
308.387644	-0.32406253	0.10581578	-3.0625163	0.0021948	0.007816581	LRRC20
2589.603479	0.211309588	0.05032024	4.1992962	2.68E-05	0.000142345	EIF4EBP2
42.70114816	1.393212721	0.35138919	3.9648708	7.34E-05	0.000359833	ADAMTS14
67.80563212	0.905619321	0.24192818	3.7433396	0.0001816	0.0008284	VSIR
10065.50019	0.609827733	0.03931298	15.512123	2.87E-54	4.80E-52	PSAP
1482.169523	0.610196598	0.07190015	8.4867218	2.13E-17	4.94E-16	CHST3
4.129082645	4.340557254	1.40909359	3.0803896	0.0020673	0.007406333	SPOCK2
648.6904432	-0.78697572	0.07849406	-10.025927	1.17E-23	4.66E-22	DDIT4
1266.841897	-0.23316154	0.05808823	-4.0139203	5.97E-05	0.000297481	MICU1
1281.260326	-0.3123019	0.05779444	-5.4036673	6.53E-08	5.37E-07	MRPS16
523.8431932	-0.49399713	0.07912386	-6.2433395	4.28E-10	4.74E-09	PPP3CB
418.7174947	-0.54721276	0.09614134	-5.691753	1.26E-08	1.15E-07	USP54
9.075848248	1.776455361	0.6610214	2.68744	0.0072002	0.022361189	MYOZ1
8.795428246	4.54227275	1.13574936	3.9993619	6.35E-05	0.000314941	SYNPO2L

978.1549285	-0.37167958	0.0627624	-5.9220103	3.18E-09	3.12E-08	FUT11
906.7302661	0.469714967	0.06849942	6.8572111	7.02E-12	9.29E-11	ZSWIM8
693.326088	1.689059151	0.08195911	20.608559	2.30E-94	1.33E-91	PLAU
15073.6529	0.477350192	0.03667826	13.014526	1.01E-38	9.67E-37	VCL
2786.245453	-0.22894998	0.04823426	-4.7466256	2.07E-06	1.34E-05	AP3M1
1898.775053	-0.14206669	0.04718106	-3.0110957	0.0026031	0.009078893	ADK
1042.455659	-0.69049106	0.06363706	-10.850455	1.98E-27	1.05E-25	KAT6B
238.6380219	0.5289553	0.11665964	4.5341757	5.78E-06	3.46E-05	ZNF503
478.5762311	-0.66991812	0.08473555	-7.9059867	2.66E-15	5.06E-14	KCNMA1
11642.00158	-0.26298282	0.03947672	-6.6617184	2.71E-11	3.37E-10	RPS24
774.1785466	-0.32103291	0.07147846	-4.4913237	7.08E-06	4.18E-05	ZMIZ1
2490.304533	0.148046033	0.05172801	2.862009	0.0042096	0.013901597	PPIF
265.8526959	-0.26545658	0.1112158	-2.3868602	0.016993	0.047062819	NUTM2B-AS1
25.49776878	-1.11620333	0.33738754	-3.3083715	0.0009384	0.003649923	ENSG00000280355
144.5538418	0.447400175	0.15241798	2.9353505	0.0033317	0.01130948	LINC00857
439.4680083	0.442914723	0.09135861	4.8480896	1.25E-06	8.39E-06	PRXL2A
1632.63824	0.257953525	0.06438052	4.0067018	6.16E-05	0.000306015	TSPAN14
3280.752078	-0.16096772	0.04503579	-3.5742177	0.0003513	0.001504235	GHITM
153.416997	0.605862302	0.16114074	3.7598331	0.00017	0.000778711	CERNA2
82.31860321	0.710318007	0.2038301	3.4848533	0.0004924	0.002047574	CDHR1
3076.111416	-0.2168062	0.04292811	-5.0504488	4.41E-07	3.19E-06	WAPL
66.21267373	1.850404336	0.28149149	6.5735712	4.91E-11	5.99E-10	LDB3
2335.5062	-0.24405517	0.04712647	-5.1787286	2.23E-07	1.69E-06	GLUD1
3313.998609	0.21137655	0.04819438	4.3859172	1.15E-05	6.59E-05	PAPSS2
1603.502999	0.140931327	0.05512671	2.5564979	0.0105732	0.031344604	ATAD1
22.55190314	1.174395237	0.42102568	2.7893672	0.0052811	0.017005046	KLLN
376.6007736	2.860052137	0.15406583	18.563832	6.30E-77	2.07E-74	ACTA2
255.4670785	2.274002722	0.1420073	16.013281	1.03E-57	2.02E-55	FAS
3.827322517	3.084520025	1.26874124	2.4311656	0.0150503	0.042476222	ENSG00000286116
486.3562806	3.169726485	0.13016374	24.35184	5.54E-131	6.89E-128	IFIT2
605.7184915	2.600577377	0.10189965	25.520964	1.15E-143	2.23E-140	IFIT3
736.3815828	3.38873179	0.28930293	11.713438	1.09E-31	7.45E-30	IFIT1
657.8111761	0.483190545	0.07876127	6.1348752	8.52E-10	9.03E-09	IFIT5
4137.156443	-0.30328395	0.04557891	-6.6540414	2.85E-11	3.54E-10	KIF20B
8693.254624	1.830237476	0.0982439	18.629528	1.85E-77	6.31E-75	ANKRD1
2717.926231	-0.20391247	0.04904517	-4.1576466	3.22E-05	0.00016888	PCGF5
619.0333646	0.257412519	0.08537603	3.0150444	0.0025694	0.008979532	HECTD2
965.7624179	0.222517426	0.07527717	2.9559748	0.0031168	0.010659113	PPP1R3C
2367.214137	-0.15831252	0.05350899	-2.9586153	0.0030902	0.010584868	BTAF1
6381.258221	-0.33256981	0.04240302	-7.8430681	4.40E-15	8.16E-14	KIF11
203.90989	0.462749858	0.12951723	3.5728826	0.0003531	0.001510659	HHEX
574.1983367	-0.33800753	0.07958187	-4.2472934	2.16E-05	0.000116744	EXOC6
56.07957825	1.887412132	0.28989795	6.5106087	7.48E-11	8.96E-10	RBP4
520.7416409	-0.26008155	0.0861116	-3.0202849	0.0025254	0.008841593	PLCE1
365.6317387	0.48430132	0.10622662	4.5591332	5.14E-06	3.11E-05	PLCE1-AS1
1577.045251	-0.28601146	0.05619561	-5.0895699	3.59E-07	2.63E-06	NOC3L
720.7140061	0.214641848	0.07398989	2.9009617	0.0037202	0.012465158	TBC1D12
1619.922536	0.348280103	0.0543608	6.4068245	1.49E-10	1.73E-09	PDLIM1

76.24791988	1.22415746	0.23472876	5.2152001	1.84E-07	1.41E-06	SORBS1
2193.609958	-0.31496915	0.04431532	-7.1074546	1.18E-12	1.71E-11	ALDH18A1
158.0681318	1.229803948	0.16680429	7.3727356	1.67E-13	2.66E-12	TLL2
8064.980877	-0.13944057	0.03673044	-3.7963212	0.0001469	0.000679224	TM9SF3
73.58526213	0.624376199	0.2066956	3.0207523	0.0025215	0.008835072	PIK3AP1
1909.497605	-0.31890096	0.06485346	-4.9172543	8.78E-07	6.04E-06	LCOR
550.8211811	-0.21449891	0.07746552	-2.7689599	0.0056236	0.017996621	ARHGAP19
737.8146915	0.233466163	0.0741969	3.1465756	0.0016519	0.006065737	RRP12
1231.189586	-0.20529854	0.05827168	-3.5231271	0.0004265	0.001796658	PGAM1
896.1250914	-0.54111585	0.06223443	-8.6947991	3.47E-18	8.73E-17	MMS19
739.5178268	0.240004106	0.07214951	3.3264829	0.0008795	0.003445461	PI4K2A
180.2567838	0.498353472	0.13201424	3.7749979	0.00016	0.000735931	AVPI1
800.9462219	0.25941705	0.068901	3.7650696	0.0001665	0.000763979	MARVELD1
403.0981035	0.353015452	0.09122307	3.8698045	0.0001089	0.0005157	ZFYVE27
1142.11015	-0.44666337	0.06035925	-7.4000815	1.36E-13	2.19E-12	GOT1
325.0521329	0.386938313	0.10937738	3.5376448	0.0004037	0.001708998	SLC25A28
1499.258905	0.338593188	0.05594716	6.0520179	1.43E-09	1.48E-08	ENTPD7
642.0407278	-0.20578406	0.07645298	-2.6916421	0.0071101	0.022126877	COX15
1826.625911	-0.39539228	0.04939443	-8.0047943	1.20E-15	2.39E-14	ERLIN1
699.8558347	-0.30540715	0.06904044	-4.4235982	9.71E-06	5.62E-05	CWF19L1
716.0400688	0.429894543	0.07616564	5.6442054	1.66E-08	1.48E-07	BLOC1S2
6776.871465	-1.25739887	0.09519008	-13.209347	7.75E-40	7.74E-38	SCD
115.3185452	-0.83339646	0.18387452	-4.5324195	5.83E-06	3.49E-05	OLMALINC
2048.302605	-0.16421784	0.05017944	-3.2726118	0.0010656	0.004081537	HIF1AN
3206.820305	-0.16742193	0.04693875	-3.5668168	0.0003613	0.001542787	SLF2
597.5724786	-0.33601527	0.07846958	-4.2821085	1.85E-05	0.000101335	MRPL43
552.5650482	0.197394799	0.0814908	2.4222955	0.0154228	0.04335638	TWNK
674.914843	-0.19782065	0.07411117	-2.6692422	0.0076023	0.023424019	BTRC
240.6791884	-0.33111605	0.11743625	-2.8195386	0.0048093	0.015658729	DPCD
342.5243601	-0.49791683	0.09614546	-5.1787867	2.23E-07	1.69E-06	NPM3
3991.045874	-0.17769871	0.0469883	-3.7817646	0.0001557	0.000717156	OGA
42.90404736	0.896982195	0.29254524	3.0661316	0.0021685	0.007733766	ELOVL3
526.9447906	0.3776487	0.08347453	4.5241192	6.06E-06	3.62E-05	NFKB2
2777.899027	0.130082214	0.04481214	2.902834	0.003698	0.012400437	ACTR1A
1229.753531	0.171522122	0.05978445	2.869009	0.0041176	0.013626086	TRIM8
460.4172001	-0.56444303	0.09299376	-6.0696869	1.28E-09	1.33E-08	ARL3
921.3677969	0.210802681	0.06640328	3.1745823	0.0015005	0.005549574	WBP1L
208.1445876	0.694653904	0.13188009	5.2673143	1.38E-07	1.09E-06	BORCS7
302.0779982	-0.34610401	0.11181052	-3.0954511	0.0019651	0.007075309	CNNM2
2775.769579	0.148405821	0.05022096	2.9550575	0.0031261	0.01068392	NT5C2
3028.323536	0.939090315	0.05428495	17.299275	4.76E-67	1.22E-64	INA
415.7511655	-0.30147505	0.08661563	-3.4806078	0.0005003	0.002076826	TAF5
2419.759475	-0.29134526	0.04600133	-6.3334097	2.40E-10	2.73E-09	SLK
28.29016442	1.60257987	0.36314735	4.4130292	1.02E-05	5.88E-05	COL17A1
409.9870862	0.790173602	0.09411186	8.3961104	4.62E-17	1.03E-15	ITPRIP
2442.380287	-0.22394714	0.0572742	-3.9100873	9.23E-05	0.000443954	ADD3
444.4851228	-0.90182705	0.11704006	-7.7052854	1.31E-14	2.34E-13	MXI1
453.1367052	0.279276589	0.0907303	3.0780961	0.0020833	0.007455893	DUSP5

5201.740483	-0.37532495	0.03904616	-9.6123391	7.09E-22	2.45E-20	SMC3
1426.405208	-0.6192582	0.0580682	-10.664326	1.49E-26	7.51E-25	PDCD4
387.0300869	-0.31442693	0.09169435	-3.4290765	0.0006056	0.002467102	BBIP1
56.90541484	2.502884648	0.37660577	6.6459009	3.01E-11	3.74E-10	ADRA2A
42.99809491	1.447696059	0.30386148	4.764329	1.89E-06	1.24E-05	ACSL5
1217.447219	-0.16661885	0.05623547	-2.9628781	0.0030478	0.010455862	ZDHHC6
581.8786616	-0.20425649	0.076681	-2.6637171	0.0077283	0.023769774	VTI1A
46.29417872	-0.6991911	0.25985652	-2.6906814	0.0071306	0.022174821	ENSG00000260917
646.3641948	0.201269246	0.07360891	2.7343053	0.0062512	0.01977596	TCF7L2
792.9914314	0.292541547	0.07210327	4.0572576	4.97E-05	0.000251296	CASP7
526.0161326	-0.449705	0.08731363	-5.1504558	2.60E-07	1.95E-06	DCLRE1A
1141.108607	-0.50577383	0.05739549	-8.8120828	1.23E-18	3.20E-17	NHLRC2
124.2707976	0.417186939	0.16118562	2.5882392	0.0096468	0.028928745	ABLIM1
1511.153789	-0.1606913	0.05590098	-2.8745702	0.0040458	0.01343186	FHIP2A
595.3325544	-0.97687561	0.0950763	-10.274649	9.17E-25	3.98E-23	GFRA1
471.9553353	0.310376858	0.08758098	3.5438842	0.0003943	0.001672317	HSPA12A
669.9423301	-0.276599	0.07080396	-3.9065469	9.36E-05	0.000449506	FAM204A
2202.904637	-0.18159967	0.04998703	-3.6329354	0.0002802	0.001221901	CACUL1
10372.33052	-0.18008441	0.03250067	-5.5409448	3.01E-08	2.60E-07	EIF3A
208.4521445	-0.33146764	0.12255072	-2.7047385	0.0068358	0.021412687	SFXN4
3294.100323	-0.22858036	0.0500992	-4.5625553	5.05E-06	3.06E-05	PRDX3
168.7086638	0.429219729	0.14154397	3.0324126	0.0024261	0.008530147	GRK5
1356.14129	0.353683032	0.06325204	5.5916461	2.25E-08	1.97E-07	BAG3
877.0078399	-0.26481194	0.06690841	-3.9578275	7.56E-05	0.000369878	INPP5F
3057.109551	-0.17126197	0.04101964	-4.1751212	2.98E-05	0.000157089	MCMBP
1712.385192	-0.27310998	0.0506014	-5.3972808	6.77E-08	5.54E-07	WDR11
655.3129315	-0.48992778	0.07269089	-6.7398782	1.59E-11	2.01E-10	ATE1
269.8071932	0.495576627	0.11775113	4.2086782	2.57E-05	0.000136813	TACC2
789.6775972	0.443453745	0.07553386	5.8709263	4.33E-09	4.17E-08	PLEKHA1
225.5797891	1.894026147	0.16781963	11.286082	1.54E-29	9.38E-28	HTRA1
449.4332065	-0.37911041	0.08864849	-4.2765579	1.90E-05	0.000103731	ACADSB
2687.248983	0.226957962	0.04987543	4.5504965	5.35E-06	3.23E-05	OAT
1449.926426	-0.18760145	0.05477679	-3.4248345	0.0006152	0.002500088	CTBP2
598.5689615	0.460423452	0.078125	5.8934205	3.78E-09	3.67E-08	UROS
1984.845916	-0.23877196	0.05130026	-4.6544008	3.25E-06	2.04E-05	BCCIP
2727.962985	-0.43508129	0.04722521	-9.212903	3.17E-20	9.55E-19	DOCK1
16.04545599	1.789267655	0.52784473	3.3897613	0.0006995	0.00279975	INSYN2A
17859.87625	-0.63430694	0.05370552	-11.810834	3.43E-32	2.44E-30	MKI67
140.237592	-0.60196849	0.14713918	-4.0911503	4.29E-05	0.000219931	MGMT
7004.501292	-0.38495885	0.04250821	-9.0561052	1.35E-19	3.85E-18	BNIP3
722.7610245	0.186543554	0.07586674	2.458832	0.013939	0.039842034	DPYSL4
1003.295318	-0.170749	0.06300937	-2.7098987	0.0067304	0.02111895	TUBGCP2
37.88895948	2.528515333	0.58873717	4.2948117	1.75E-05	9.62E-05	IFITM1
1109.914653	0.983952037	0.12784416	7.6964955	1.40E-14	2.49E-13	IFITM3
1302.13096	0.24354615	0.05993088	4.0637839	4.83E-05	0.000245082	PHRF1
174.0166712	1.266744643	0.15614466	8.1126352	4.95E-16	1.02E-14	IRF7
3086.356602	0.329717322	0.04182246	7.8837384	3.18E-15	5.99E-14	EPS8L2
1905.216428	-0.22271539	0.04738229	-4.7003932	2.60E-06	1.66E-05	TALDO1

214.4969382	0.494763241	0.12714402	3.8913608	9.97E-05	0.000476265	PIDD1
3745.144877	-0.15682283	0.04623771	-3.3916649	0.0006947	0.002783575	RPLP2
1174.180936	0.16058124	0.06454948	2.4877232	0.0128564	0.037132095	PNPLA2
2397.43783	0.162197063	0.05334618	3.0404623	0.0023622	0.008325462	CD151
846.1835682	0.37503739	0.07530407	4.9803072	6.35E-07	4.48E-06	POLR2L
688.5050759	0.23884706	0.07625013	3.132415	0.0017337	0.006337567	TOLLIP
562.1945385	0.31413176	0.08665845	3.6249407	0.000289	0.001257177	CTSD
911.1200374	-0.62612809	0.07203816	-8.6916174	3.57E-18	8.94E-17	IGF2
2233.444151	0.288750302	0.05068248	5.6972405	1.22E-08	1.11E-07	CD81
42.77635638	0.848652064	0.27900236	3.0417379	0.0023522	0.008295308	SLC22A18
328.6470473	0.7156935	0.13090177	5.467409	4.57E-08	3.84E-07	PHLDA2
1571.140437	-0.24744609	0.05325942	-4.6460529	3.38E-06	2.12E-05	CARS1
659.8123782	0.346963432	0.07732303	4.4871939	7.22E-06	4.26E-05	ZNF195
4943.539774	-0.19695829	0.04182227	-4.7094114	2.48E-06	1.59E-05	NUP98
1142.808028	0.411259711	0.06627518	6.2053351	5.46E-10	5.96E-09	STIM1
7530.907439	-0.2527928	0.03572432	-7.076211	1.48E-12	2.10E-11	RRM1
297.2711773	0.567347739	0.10944892	5.183676	2.18E-07	1.65E-06	TRIM21
813.716403	0.433279966	0.0680394	6.3680746	1.91E-10	2.20E-09	TRIM5
482.6379603	2.627970417	0.10982194	23.929375	1.52E-126	1.65E-123	TRIM22
92.95611749	0.637190096	0.19349909	3.2929876	0.0009913	0.003835041	CAVIN3
631.3966423	0.266560521	0.07991852	3.3354035	0.0008518	0.00334509	SMPD1
418.1421467	0.254072165	0.09217263	2.7564816	0.0058427	0.018612269	TRIM3
925.1451835	0.277497891	0.06502634	4.2674692	1.98E-05	0.00010781	ARFIP2
86.1613646	-0.64525516	0.20706307	-3.1162252	0.0018318	0.006653177	DCHS1
55.37063463	-0.69663683	0.23609735	-2.9506339	0.0031712	0.010819623	ZNF214
31.41169315	1.045693028	0.34105954	3.0660131	0.0021693	0.007735245	PPFIBP2
183.2293253	-1.22211617	0.12630463	-9.6759412	3.82E-22	1.35E-20	NLRP10
2355.947182	-0.220438	0.04430123	-4.9758889	6.49E-07	4.57E-06	EIF3F
690.4429942	0.32195552	0.07545681	4.2667525	1.98E-05	0.000108089	TUB
108.9592114	0.466153589	0.1729204	2.6957697	0.0070226	0.021902032	STK33
6060.218045	-0.38572624	0.03714277	-10.384962	2.90E-25	1.33E-23	RPL27A
1315.324843	0.401742406	0.0618217	6.4984045	8.12E-11	9.68E-10	NRIP3
10260.95844	-0.36838914	0.03886329	-9.4791035	2.56E-21	8.45E-20	IPO7
446.6715136	-0.26262682	0.08804703	-2.9828014	0.0028562	0.009870803	ZNF143
1639.929733	-0.20990562	0.05473703	-3.8348008	0.0001257	0.000588404	WEE1
68.2925542	0.760629678	0.22308952	3.4095268	0.0006508	0.002623241	SBF2-AS1
1056.873402	-0.81814792	0.06396628	-12.7903	1.86E-37	1.71E-35	SBF2
2607.289395	-0.16182609	0.0554665	-2.9175464	0.003528	0.011880602	ADM
153.2225276	0.708462096	0.16360801	4.330241	1.49E-05	8.31E-05	MTRNR2L8
2148.006369	0.235198311	0.05228261	4.4985953	6.84E-06	4.05E-05	RNF141
4.412395233	4.465111358	1.37214066	3.2541207	0.0011374	0.004331086	IRAG1
72.22239472	0.768774822	0.22377764	3.4354407	0.0005916	0.002414402	ZBED5-AS1
3495.821743	0.205066069	0.04429158	4.6299104	3.66E-06	2.28E-05	DKK3
4659.933306	0.179723561	0.04523457	3.9731459	7.09E-05	0.000348831	MICAL2
12958.56397	-0.23582492	0.04256224	-5.5407074	3.01E-08	2.60E-07	TEAD1
2323.972882	-0.33458609	0.05319971	-6.2892464	3.19E-10	3.58E-09	RRAS2
98.8996152	0.618465876	0.18418979	3.3577642	0.0007858	0.003107642	CYP2R1
4093.124037	-0.15435297	0.04200624	-3.6745248	0.0002383	0.001057389	C11orf58

294.8796965	0.844116922	0.1164766	7.2470944	4.26E-13	6.47E-12	PLEKHA7
4571.694508	-0.25366326	0.04477226	-5.6656345	1.46E-08	1.32E-07	RPS13
14.78327804	1.148687012	0.45813748	2.5072976	0.0121658	0.035396294	SNORD14A
3399.818747	-0.30009209	0.05582251	-5.3758255	7.62E-08	6.21E-07	PIK3C2A
1290.467049	-0.40200783	0.05530315	-7.2691673	3.62E-13	5.53E-12	NUCB2
18.22651649	1.095122108	0.44577186	2.4566874	0.0140225	0.040074062	ENSG00000260196
854.9568933	-0.44087082	0.06703339	-6.5768835	4.80E-11	5.88E-10	SAAL1
1971.647922	0.2485724	0.04866758	5.1075565	3.26E-07	2.41E-06	HPS5
83672.42042	-0.1561926	0.03160351	-4.9422552	7.72E-07	5.37E-06	LDHA
45.92266747	-0.61366011	0.25164417	-2.4386025	0.0147442	0.041765961	ENSG00000256006
639.957707	-0.22304882	0.07664005	-2.9103428	0.0036103	0.012129744	ZDHHC13
291.838514	-0.66754592	0.10558119	-6.3225839	2.57E-10	2.91E-09	E2F8
1086.450188	0.383250661	0.07586591	5.0516846	4.38E-07	3.17E-06	NAV2
15.63031953	1.77138591	0.48460648	3.6553079	0.0002569	0.001131466	NAV2-AS6
434.840946	-0.65253426	0.08653577	-7.5406307	4.68E-14	7.90E-13	PRMT3
687.0331748	-0.5689168	0.07328538	-7.7630329	8.29E-15	1.51E-13	CCDC34
1907.416695	0.207450614	0.05766001	3.5978247	0.0003209	0.001382282	LGR4
2319.364111	-0.11013077	0.04608123	-2.3899266	0.0168517	0.046761089	KIF18A
353.8753115	-0.49380173	0.09656626	-5.113605	3.16E-07	2.34E-06	METTL15
581.4667981	-0.20065034	0.07886632	-2.5441828	0.0109534	0.03234491	DNAJC24
388.1942824	-0.57487964	0.09077254	-6.333189	2.40E-10	2.73E-09	ELP4
1968.912716	-0.15258006	0.05021558	-3.0385007	0.0023776	0.008373069	RCN1
3616.324402	-0.19633564	0.04657749	-4.215247	2.50E-05	0.000133177	EIF3M
2472.221934	-0.69591055	0.0539456	-12.900227	4.49E-38	4.22E-36	QSER1
290.4857459	0.578436729	0.1108762	5.2169601	1.82E-07	1.40E-06	DEPDC7
617.3080962	0.264690263	0.07968193	3.3218353	0.0008943	0.003493919	TCP11L1
6379.319121	0.308924914	0.04202586	7.3508288	1.97E-13	3.12E-12	CD59
1004.592491	-0.37969496	0.06088613	-6.2361482	4.48E-10	4.94E-09	FBXO3
11085.34768	-0.39121764	0.03983814	-9.8201789	9.22E-23	3.47E-21	CAPRIN1
1543.061455	-0.28298204	0.05120006	-5.5269868	3.26E-08	2.80E-07	NAT10
445.885612	1.327656475	0.09928826	13.371737	8.85E-41	9.10E-39	ABTB2
446.7149947	-0.30300476	0.08650203	-3.5028632	0.0004603	0.001925516	APIP
5.51442554	2.200265428	0.86660945	2.5389354	0.011119	0.032772884	ENSG00000289526
11.1111888	1.603111823	0.60580608	2.6462458	0.0081391	0.024874926	ENSG00000255521
12992.81837	0.261588274	0.04804678	5.4444491	5.20E-08	4.33E-07	CD44
5.35092194	2.692734821	1.00507032	2.6791507	0.0073809	0.022855084	ENSG00000251194
387.2570734	-0.31589268	0.09399169	-3.3608576	0.000777	0.003076544	FJX1
5131.946018	-0.30572658	0.03871869	-7.8960987	2.88E-15	5.45E-14	TRIM44
460.8760956	0.332032618	0.09060018	3.6648119	0.0002475	0.001094705	PRR5L
253.6238365	-0.39506902	0.11181617	-3.5332012	0.0004106	0.001736715	IFTAP
4537.219318	-0.18666941	0.04186722	-4.4586052	8.25E-06	4.84E-05	API5
1586.307632	-0.15393674	0.05480704	-2.8087038	0.0049741	0.016126077	TTC17
782.6520956	0.20213443	0.07410871	2.7275395	0.0063809	0.020138507	HSD17B12
5.660480409	3.022621203	1.20455956	2.5093165	0.0120965	0.03522408	C11orf96
152.8180028	0.566865424	0.15589737	3.6361449	0.0002767	0.001208907	ACCS
392.4819749	1.149436514	0.10026654	11.463809	2.00E-30	1.28E-28	CD82
646.9132105	0.83375642	0.08688712	9.5958571	8.32E-22	2.83E-20	TP53I11
258.1495625	0.573876114	0.11282828	5.0862791	3.65E-07	2.67E-06	SLC35C1

252.6228994	0.331606861	0.1201941	2.7589279	0.0057991	0.018493843	MAPK8IP1
446.8520051	-0.33049377	0.08976509	-3.6817629	0.0002316	0.001030967	PHF21A
823.3000492	-0.33111576	0.06455588	-5.1291342	2.91E-07	2.17E-06	CREB3L1
512.3127038	0.385895307	0.08594895	4.4898197	7.13E-06	4.21E-05	DGKZ
785.7684881	0.523178189	0.07138432	7.3290356	2.32E-13	3.64E-12	MDK
153.9547907	0.408382882	0.15067102	2.7104275	0.0067197	0.021089115	ZNF408
11690.32566	-0.28721755	0.03782968	-7.5923863	3.14E-14	5.40E-13	CKAP5
389.0473824	-0.6663798	0.09259776	-7.1964999	6.18E-13	9.21E-12	LRP4
219.8934789	-0.74554994	0.11838972	-6.2974215	3.03E-10	3.41E-09	C11orf49
917.3051436	-0.20665661	0.06396632	-3.2307096	0.0012348	0.004663138	ARFGAP2
816.5633558	0.901531337	0.07996833	11.273605	1.77E-29	1.08E-27	DDB2
378.8482182	0.257753291	0.09299817	2.7715952	0.0055782	0.017861445	ACP2
1341.499006	-0.15730877	0.05465584	-2.8781695	0.0039999	0.013294778	MADD
3564.939897	-0.24778521	0.04004763	-6.1872621	6.12E-10	6.62E-09	PSMC3
3559.46466	-0.17509866	0.04737876	-3.6957203	0.0002193	0.00098248	CELF1
2235.020981	-0.39791876	0.04534332	-8.7756863	1.70E-18	4.35E-17	NUP160
6708.244264	-0.57251355	0.03331996	-17.182301	3.60E-66	8.83E-64	SSRP1
9.035933051	-1.41361329	0.58960795	-2.3975479	0.0165052	0.045968262	RTN4RL2
380.6138215	0.868635926	0.10854493	8.0025469	1.22E-15	2.42E-14	UBE2L6
12.16699024	2.253519967	0.66224245	3.4028625	0.0006668	0.002683091	SERPING1
374.9771637	0.230059577	0.09305881	2.4721956	0.0134286	0.038547937	CLP1
2770.387295	-0.25356255	0.04498438	-5.6366804	1.73E-08	1.55E-07	ZFP91
2776.453732	-0.54071236	0.04820881	-11.216048	3.40E-29	2.03E-27	FAM111B
3221.741567	-0.30188446	0.04287662	-7.0407715	1.91E-12	2.68E-11	FAM111A
2551.502881	-0.3077462	0.04607914	-6.6786439	2.41E-11	3.01E-10	PATL1
1029.033282	0.389088531	0.06000516	6.4842511	8.92E-11	1.06E-09	STX3
738.9385849	-0.18348586	0.07029817	-2.6101086	0.0090513	0.027293728	MRPL16
2076.13521	-0.20937819	0.05173817	-4.0468807	5.19E-05	0.000261327	PRPF19
227.7596193	0.807508411	0.12856307	6.2810292	3.36E-10	3.76E-09	TMEM132A
6.634070171	2.806640262	0.86310506	3.2517945	0.0011468	0.004360958	SLC15A3
6516.691779	-0.15836556	0.03479743	-4.5510709	5.34E-06	3.22E-05	DDB1
563.5209559	0.34203054	0.0807488	4.2357352	2.28E-05	0.000122527	TMEM138
86.19079376	-0.45709771	0.18727248	-2.4408163	0.0146541	0.041544656	TMEM216
26.32482874	1.370390673	0.35693352	3.8393443	0.0001234	0.000578245	RPLP0P2
667.8103694	-0.20164456	0.0749812	-2.6892681	0.0071609	0.022264949	MYRF
2624.722594	-0.603676	0.05203814	-11.600645	4.09E-31	2.72E-29	FADS2
4528.131927	-0.35306759	0.04815947	-7.3312187	2.28E-13	3.58E-12	FADS1
1158.177198	0.505004213	0.06178868	8.1730864	3.01E-16	6.29E-15	FADS3
7895.285886	0.563391345	0.0371862	15.150551	7.51E-52	1.16E-49	FTH1
2124.239247	-0.18279212	0.05525243	-3.308309	0.0009386	0.003649923	INCENP
353.3319721	0.362093688	0.1014559	3.5689761	0.0003584	0.001531256	ASRGL1
47138.4921	-0.79235077	0.13174194	-6.0144157	1.81E-09	1.84E-08	AHNAK
317.8208104	-0.32958477	0.10298643	-3.2002736	0.001373	0.005122502	EML3
241.1054212	0.487744534	0.12456897	3.9154577	9.02E-05	0.000434908	B3GAT3
12147.73121	-0.12025774	0.03954441	-3.0410808	0.0023573	0.008310063	GANAB
151.4476982	0.804650441	0.15210467	5.2901101	1.22E-07	9.66E-07	LBHD1
934.6180973	-0.16429498	0.06170798	-2.6624593	0.0077572	0.023846145	UBXN1
129.6418969	-0.53832789	0.15203107	-3.5409071	0.0003988	0.001690064	TMEM223

2041.490835	0.499006276	0.05551165	8.9892165	2.49E-19	6.95E-18	NXF1
2599.667528	0.484958438	0.05378989	9.015792	1.95E-19	5.50E-18	SLC3A2
18.26172175	2.004482759	0.4956952	4.0437809	5.26E-05	0.000264272	PLAAT4
548.8660738	0.457643773	0.087712	5.2175731	1.81E-07	1.40E-06	PLAAT3
992.6696247	0.152119792	0.06102563	2.4927199	0.0126769	0.036674589	MARK2
6064.278586	-0.23106306	0.04425654	-5.220992	1.78E-07	1.37E-06	STIP1
10.03967705	1.463636706	0.61083815	2.3961122	0.01657	0.04611918	FERMT3
352.4272177	-0.6316877	0.10348997	-6.1038544	1.04E-09	1.09E-08	VEGFB
1225.997505	-0.20593932	0.05865347	-3.5111192	0.0004462	0.001870743	PLCB3
881.8259063	-0.35449577	0.06775187	-5.2322657	1.67E-07	1.30E-06	TRMT112
898.6581857	-0.19939521	0.06253013	-3.1887862	0.0014287	0.005311107	MEN1
27.29299749	1.521973953	0.38950945	3.9074121	9.33E-05	0.000448276	CDC42BPG
3147.249179	0.486688634	0.05160571	9.4309069	4.07E-21	1.32E-19	EHD1
604.9439199	0.282611005	0.07976353	3.5431107	0.0003954	0.001676821	ATG2A
7.557274233	2.89980556	0.82935355	3.4964649	0.0004715	0.001968973	BATF2
722.7097495	0.262618557	0.07279394	3.607698	0.0003089	0.001335044	SYVN1
37.61367384	1.718002466	0.331663	5.1799642	2.22E-07	1.68E-06	SLC25A45
532.9241613	0.740816985	0.08761217	8.4556398	2.78E-17	6.37E-16	FRMD8
6387.600167	0.650475518	0.19186705	3.3902408	0.0006983	0.002796144	NEAT1
444.4704158	0.3339595	0.09608696	3.4755966	0.0005097	0.002111985	LTBP3
525.8169527	0.273115958	0.08721178	3.1316407	0.0017383	0.006350729	EHBP1L1
675.3775149	0.204173614	0.07558538	2.7012316	0.0069083	0.021603254	MAP3K11
444.5088177	-0.25545141	0.09311101	-2.7435146	0.0060785	0.019300431	KAT5
13847.51873	0.149838075	0.03247195	4.6143844	3.94E-06	2.45E-05	CFL1
1214.717854	0.403769397	0.05964797	6.7692055	1.29E-11	1.66E-10	EFEMP2
1990.663514	-0.28289995	0.04660626	-6.0699994	1.28E-09	1.33E-08	SART1
1442.628607	-0.20318136	0.0539702	-3.7646951	0.0001668	0.000764924	BANF1
6255.967633	-0.21764619	0.03610864	-6.0275385	1.66E-09	1.70E-08	SF3B2
812.1094461	0.184094192	0.07160606	2.5709305	0.0101426	0.030191587	BRMS1
379.9771406	-0.4645516	0.09135301	-5.0852357	3.67E-07	2.69E-06	B4GAT1
29.77207333	1.707136161	0.38582936	4.4245885	9.66E-06	5.60E-05	ENSG00000254510
16.81795141	1.556966855	0.50134758	3.1055637	0.0018992	0.006866219	DPP3-DT
546.0767423	0.205801767	0.08306411	2.4776256	0.013226	0.038047949	DPP3
462.9499386	0.513523385	0.09149153	5.612797	1.99E-08	1.76E-07	CTSF
277.0862171	0.444657113	0.11735635	3.7889481	0.0001513	0.0006984	CCS
265.1669674	-0.31542531	0.11335039	-2.7827457	0.0053901	0.01731964	PC
2489.607252	-0.1999083	0.05692427	-3.5118293	0.000445	0.001866202	KDM2A
832.6939246	-0.17763177	0.06531771	-2.7195039	0.006538	0.020589587	GRK2
2172.488902	-0.15706719	0.05084092	-3.0893856	0.0020057	0.007204976	PPP1CA
15.01787501	1.643440903	0.55752345	2.9477521	0.0032009	0.01090815	CARNS1
523.5456245	0.268987928	0.09241518	2.9106467	0.0036068	0.012120296	CDK2AP2
2051.297275	0.199572962	0.0551538	3.6184809	0.0002963	0.001285755	GSTP1
192.050212	0.569155298	0.13374617	4.2554884	2.09E-05	0.00011318	TCIRG1
969.5533822	0.384245248	0.06922831	5.5504061	2.85E-08	2.47E-07	C11orf24
5414.409068	-0.17705223	0.03948335	-4.4842255	7.32E-06	4.32E-05	PPP6R3
489.7635735	0.420690956	0.08872936	4.7412824	2.12E-06	1.37E-05	CPT1A
7618.346236	0.307688911	0.10309739	2.9844492	0.0028409	0.009819745	CCND1
356.1597663	0.338605941	0.10467827	3.23473	0.0012176	0.004601974	LTO1

3113.743677	0.123946822	0.04362965	2.8408853	0.0044988	0.014747407	PPFIA1
6099.640816	0.287346179	0.03791748	7.5781983	3.50E-14	6.00E-13	CTTN
144.1204362	-0.79711531	0.15528141	-5.1333597	2.85E-07	2.13E-06	SHANK2
1041.165704	-0.30962212	0.05873541	-5.2714734	1.35E-07	1.06E-06	DHCR7
740.3673444	0.383020872	0.0711941	5.3799521	7.45E-08	6.08E-07	NADSYN1
648.1594775	0.257263611	0.08338579	3.0852212	0.002034	0.007299092	RNF121
4170.56467	-0.27894071	0.04222231	-6.606477	3.94E-11	4.84E-10	NUMA1
10.38040157	2.004839985	0.65741025	3.0496026	0.0022914	0.00811242	FOLR3
2660.123746	0.145191137	0.04821679	3.0112151	0.002602	0.009077141	INPPL1
661.1089953	-0.18738664	0.0769182	-2.4361809	0.0148433	0.041978251	CLPB
728.1255105	-0.24375525	0.07054079	-3.4555221	0.0005492	0.002256337	FCHSD2
549.7770206	0.669204125	0.08419335	7.9484202	1.89E-15	3.66E-14	RELT
1806.119673	-0.25286869	0.05337757	-4.7373582	2.17E-06	1.40E-05	FAM168A
31.07423393	1.287923959	0.35737502	3.6038444	0.0003135	0.001352989	PLEKHB1
310.2283725	-0.42303269	0.10088176	-4.1933515	2.75E-05	0.00014595	MRPL48
405.3981395	-0.2794431	0.09506912	-2.9393675	0.0032888	0.011174819	PAAF1
1828.223545	0.347970756	0.04901499	7.0992721	1.25E-12	1.80E-11	UCP2
7622.86965	0.226269208	0.03832707	5.9036402	3.56E-09	3.46E-08	PPME1
704.8072676	0.662251619	0.07618093	8.6931415	3.53E-18	8.85E-17	PGM2L1
1031.179397	-0.417925	0.06604995	-6.3274083	2.49E-10	2.83E-09	POLD3
867.0768001	-0.49837508	0.06644309	-7.5007816	6.34E-14	1.06E-12	RNF169
728.673995	-0.18080983	0.07605829	-2.3772535	0.0174421	0.048130448	NEU3
191.3460541	0.562673454	0.12970921	4.3379608	1.44E-05	8.05E-05	ARRB1
12842.18069	-0.41113942	0.03981483	-10.326288	5.36E-25	2.38E-23	RPS3
183.7651343	0.77985066	0.13843664	5.6332676	1.77E-08	1.58E-07	GDPD5
3591.702689	0.288003682	0.04613883	6.2421111	4.32E-10	4.77E-09	SERPINH1
32.93200478	1.237815035	0.36819038	3.3618886	0.0007741	0.003067176	MAP6
464.5214319	-0.25384329	0.08503657	-2.9851072	0.0028348	0.009804492	UVRAG
8.151098425	2.794710574	0.99172852	2.8180198	0.0048321	0.015713791	WNT11
1309.130262	-0.24616505	0.05813952	-4.2340402	2.30E-05	0.000123347	THAP12
43.47821342	-0.86871379	0.26330931	-3.2992141	0.0009696	0.003761017	EMSY-DT
1380.20255	-0.41489183	0.05569676	-7.4491193	9.40E-14	1.53E-12	EMSY
10.44948289	3.200710043	0.86694796	3.6919287	0.0002226	0.00099494	LINC02757
20.8969564	1.701955146	0.46496419	3.6604005	0.0002518	0.001111747	LRRC32
239.1679313	0.308781429	0.11965656	2.5805641	0.0098639	0.029488277	TSKU
900.2114018	0.483514379	0.06605385	7.3200033	2.48E-13	3.87E-12	CAPN5
970.3975776	-0.25694005	0.062959	-4.0810695	4.48E-05	0.000228883	PAK1
2109.93315	-0.37206088	0.04866052	-7.6460516	2.07E-14	3.62E-13	CLNS1A
3240.08085	-0.16889759	0.04652672	-3.63012	0.0002833	0.001234069	RSF1
11.65779846	1.43334486	0.54838413	2.6137607	0.0089552	0.027049119	FTH1P16
232.2220552	0.318683511	0.12028406	2.6494243	0.0080629	0.024663836	AAMDC
609.2254238	-0.35247442	0.07512697	-4.6917161	2.71E-06	1.73E-05	INTS4
254.6423784	0.559921342	0.11881101	4.7127058	2.44E-06	1.57E-05	NDUFC2
473.9153962	-0.38122648	0.08330789	-4.5761149	4.74E-06	2.89E-05	NARS2
1261.896623	0.158844798	0.06118209	2.5962631	0.0094244	0.0283155	DDIAS
1397.814632	0.345076394	0.0608082	5.6748332	1.39E-08	1.26E-07	RAB30
542.6992547	-0.23017081	0.08282514	-2.7789969	0.0054527	0.017501393	TMEM126B
348.0494622	-0.43411927	0.10525078	-4.124618	3.71E-05	0.000192597	TMEM126A

464.7340112	1.183927335	0.09347522	12.665681	9.16E-37	8.09E-35	SYTL2
10630.30002	0.223210455	0.04196761	5.3186359	1.05E-07	8.35E-07	PICALM
73.61710096	1.778348717	0.24234243	7.3381648	2.17E-13	3.41E-12	CCDC81
2395.532626	0.881595682	0.0516441	17.0706	2.46E-65	5.85E-63	PRSS23
345.0139801	-0.47421349	0.09867176	-4.8059695	1.54E-06	1.02E-05	TMEM135
1407.699344	-0.19985415	0.05953773	-3.3567648	0.0007886	0.003118186	CTSC
76.9735968	2.426238848	0.28046105	8.6508941	5.11E-18	1.25E-16	NAALAD2
754.738982	0.74269245	0.0926914	8.0125278	1.12E-15	2.25E-14	FAT3
8.69132606	2.204127016	0.65887233	3.3453021	0.0008219	0.003241124	HEPHL1
1805.128873	-0.34396159	0.05106542	-6.7357053	1.63E-11	2.07E-10	MRE11
191.5392578	1.065523756	0.14646744	7.2748163	3.47E-13	5.32E-12	ENDOD1
77.55371739	1.645677293	0.27515842	5.9808357	2.22E-09	2.23E-08	SESN3
1106.132506	-0.47080172	0.06533923	-7.2054986	5.78E-13	8.63E-12	CEP57
1346.764962	-0.36143371	0.05421875	-6.6662126	2.62E-11	3.27E-10	MTMR2
564.959161	-0.4776903	0.08010133	-5.963575	2.47E-09	2.47E-08	MAML2
2074.487523	0.229042374	0.04799469	4.7722442	1.82E-06	1.19E-05	CCDC82
224.9173052	0.688477684	0.13080143	5.2635333	1.41E-07	1.11E-06	ARHGAP42
81.71367428	-0.86512007	0.19038735	-4.5439997	5.52E-06	3.32E-05	CEP126
5078.170417	-0.4044041	0.0467331	-8.6534837	5.00E-18	1.23E-16	YAP1
323.5995444	1.897162839	0.11669462	16.257501	1.98E-59	4.04E-57	BIRC3
3010.540136	0.780223907	0.04786764	16.299612	9.93E-60	2.06E-57	BIRC2
162.1913622	1.39277294	0.1525026	9.1327814	6.68E-20	1.95E-18	MMP1
5.628054015	2.084207192	0.84863705	2.4559465	0.0140514	0.040128327	MMP3
4.51532607	2.44894214	0.97385083	2.5146994	0.0119134	0.034784057	MMP13
813.2302478	-1.07167431	0.13324454	-8.0429138	8.77E-16	1.77E-14	DYNC2H1
24.62225732	1.276118407	0.42000859	3.038315	0.0023791	0.008376534	PDGFD
51.27585693	1.230356596	0.26398835	4.6606473	3.15E-06	1.99E-05	CASP4
20.84636843	1.861654337	0.43945923	4.236239	2.27E-05	0.000122335	CASP1
664.7982822	-0.32126822	0.07220473	-4.4494066	8.61E-06	5.03E-05	CWF19L2
2512.450805	-0.2368546	0.0463296	-5.1123819	3.18E-07	2.35E-06	CUL5
1236.939081	-0.33214188	0.05789373	-5.7370958	9.63E-09	8.89E-08	ACAT1
2033.311964	-0.14803087	0.04875948	-3.0359401	0.0023979	0.008439377	NPAT
2495.425578	-0.51606635	0.06383111	-8.0848719	6.22E-16	1.27E-14	ATM
2477.450011	-0.57159234	0.04945692	-11.557379	6.77E-31	4.48E-29	POGLUT3
6886.123782	-0.30885035	0.04085047	-7.5605088	4.01E-14	6.84E-13	RDX
1981.791011	0.156402316	0.0497689	3.142571	0.0016747	0.00613896	LAYN
1407.404027	-0.21576198	0.06004967	-3.5930589	0.0003268	0.001406428	PPP2R1B
29.38850909	-1.11619429	0.31532538	-3.5398175	0.0004004	0.00169623	FDXACB1
410.837019	2.35290148	0.12129274	19.398536	7.94E-84	3.29E-81	CRYAB
228.5985106	0.50531565	0.12287069	4.1125809	3.91E-05	0.0002019	DIXDC1
1156.493262	-0.22891202	0.06178784	-3.7048072	0.0002116	0.000951107	NKAPD1
385.7748445	0.255021071	0.10619343	2.4014769	0.016329	0.04552862	TIMM8B
985.5702559	-0.28168342	0.06530652	-4.3132513	1.61E-05	8.92E-05	SDHD
131.7778052	2.291783259	0.19326171	11.858445	1.95E-32	1.42E-30	IL18
761.1543075	0.222639553	0.06912264	3.2209353	0.0012777	0.004804264	PTS
1123.276897	-0.19410756	0.05892482	-3.2941562	0.0009872	0.003819981	ZW10
883.7080743	-0.55584425	0.06489424	-8.5653864	1.08E-17	2.57E-16	USP28
610.9257923	-0.36412347	0.07577795	-4.8051376	1.55E-06	1.03E-05	RBM7

1405.044396	1.367974972	0.0654096	20.913978	3.99E-97	2.40E-94	CADM1
456.530879	-0.2181296	0.0892421	-2.4442456	0.0145155	0.04120556	BUD13
15512.01112	1.090043968	0.03822763	28.51456	7.73E-179	4.48E-175	TAGLN
645.6781881	0.304657502	0.08370496	3.6396588	0.000273	0.001194631	PCSK7
597.0617031	-0.35661271	0.07884508	-4.5229546	6.10E-06	3.63E-05	RNF214
402.4037786	0.494477602	0.10170784	4.861745	1.16E-06	7.86E-06	BACE1
107.0769084	-0.96759116	0.1699239	-5.6942617	1.24E-08	1.13E-07	BACE1-AS
876.5444613	0.401965193	0.06737218	5.9663384	2.43E-09	2.43E-08	CEP164
5.907655823	2.458574733	0.88307925	2.784093	0.0053678	0.017257428	SCN4B
439.3492423	0.234235931	0.08726365	2.6842326	0.0072697	0.022550701	ATP5MG
3154.021674	-0.55325686	0.12884974	-4.2938144	1.76E-05	9.66E-05	KMT2A
377.1196629	-0.27073893	0.09621864	-2.8137887	0.0048961	0.015905824	IFT46
5074.170771	-0.33562433	0.04714451	-7.1190541	1.09E-12	1.57E-11	DDX6
1589.073296	0.286093392	0.0544769	5.2516459	1.51E-07	1.17E-06	BCL9L
402.9601917	0.300964082	0.12253533	2.4561413	0.0140438	0.040121827	CENATAC
5029.943176	-0.29357771	0.04421282	-6.6401044	3.13E-11	3.88E-10	RPS25
369.261127	-0.4624442	0.10028857	-4.6111354	4.00E-06	2.48E-05	SLC37A4
5788.626986	0.17863432	0.03965107	4.5051574	6.63E-06	3.93E-05	HYOU1
344.0280587	0.552627263	0.10035787	5.5065661	3.66E-08	3.12E-07	C2CD2L
11.79301139	1.583006664	0.53549452	2.9561585	0.003115	0.010654863	ABCG4
149.6954943	0.478845154	0.16686351	2.8696816	0.0041089	0.013604905	NLRX1
3169.84045	-0.47255096	0.05504184	-8.5853042	9.06E-18	2.19E-16	CBL
6707.290622	1.035407633	0.04229328	24.481613	2.32E-132	3.10E-129	MCAM
1213.912551	-0.15070797	0.05635744	-2.6741451	0.007492	0.023145487	RNF26
5637.200951	0.624822667	0.03983935	15.683554	1.96E-55	3.44E-53	THY1
102.45379	1.033169338	0.20060243	5.1503331	2.60E-07	1.95E-06	NECTIN1
7.248253382	4.28279067	1.30589693	3.2795779	0.0010396	0.003995377	TRIM29
6281.178877	-0.29199408	0.04682515	-6.2358381	4.49E-10	4.95E-09	ARHGEF12
3180.082944	-0.34139588	0.04814234	-7.0913855	1.33E-12	1.90E-11	UBASH3B
39920.77055	-0.16194934	0.04724396	-3.4279371	0.0006082	0.002474581	HSPA8
814.0658917	-0.18989575	0.06966403	-2.7258796	0.006413	0.020232716	TBRG1
101.341422	1.136287638	0.20480388	5.5481743	2.89E-08	2.50E-07	SIAE
154.5652072	-0.65443976	0.14856493	-4.4050758	1.06E-05	6.09E-05	SPA17
385.1980232	-0.3135065	0.09200358	-3.4075469	0.0006555	0.002641122	CCDC15
113.2694836	0.780459581	0.16896583	4.6190379	3.86E-06	2.40E-05	SLC37A2
231.1154878	-0.40322965	0.12953841	-3.1128192	0.0018531	0.00672061	TMEM218
9.49706121	2.143267052	0.78243019	2.7392438	0.0061581	0.019513268	FEZ1
5096.749346	-0.16868979	0.03812936	-4.4241445	9.68E-06	5.61E-05	STT3A
180.273273	0.578593858	0.13697541	4.2240711	2.40E-05	0.000128499	HYLS1
567.8386311	-0.19209889	0.07960783	-2.4130653	0.015819	0.044298249	RPUSD4
570.2811893	0.2868182	0.0840711	3.4116149	0.0006458	0.002606857	FAM118B
375.4362044	-0.76310821	0.09640747	-7.9154467	2.46E-15	4.70E-14	KIRREL3
5747.905266	0.274849711	0.04253798	6.4612782	1.04E-10	1.23E-09	ETS1
250.2694574	0.332714048	0.11549005	2.8808894	0.0039655	0.013203308	FLI1
271.318049	-0.46632029	0.11143654	-4.1846265	2.86E-05	0.00015107	ARHGAP32
363.9953499	-0.46077045	0.09320324	-4.9437172	7.66E-07	5.34E-06	PRDM10
15526.52971	0.627390676	0.03969439	15.805526	2.85E-56	5.16E-54	APLP2
1968.997018	-0.47029147	0.05358759	-8.7761267	1.69E-18	4.34E-17	ZBTB44

259.7671135	1.634496257	0.1403539	11.645535	2.42E-31	1.63E-29	ADAMTS15
16.88714023	3.079672802	0.61855425	4.9788241	6.40E-07	4.51E-06	OPCML
4756.894443	-0.33033999	0.03786968	-8.7230733	2.71E-18	6.83E-17	NCAPD3
537.0929237	-0.20420636	0.07890725	-2.5879288	0.0096555	0.028944839	THYN1
581.8566642	0.466064892	0.07888307	5.9083004	3.46E-09	3.37E-08	ACAD8
1992.079049	-0.23166274	0.05878945	-3.9405497	8.13E-05	0.000394564	KDM5A
7426.577449	-0.17637913	0.05055862	-3.4886065	0.0004855	0.002022434	WNK1
2155.660773	-0.15998064	0.05352669	-2.9888014	0.0028007	0.009700238	ERC1
1084.853868	-0.19681034	0.06186795	-3.1811355	0.001467	0.005439436	WNT5B
3103.059416	-0.24790607	0.05948192	-4.1677552	3.08E-05	0.000161907	FKBP4
1041.886532	-0.20502241	0.05899333	-3.475349	0.0005102	0.002113432	RHNO1
1474.315659	-0.29537607	0.0539671	-5.4732619	4.42E-08	3.72E-07	TULP3
496.7529404	0.273132732	0.08364999	3.2651854	0.0010939	0.004182794	TSPAN9
5.923244142	2.999894272	1.02433808	2.9286173	0.0034047	0.011512406	ENSG00000250770
338.8723865	-0.70613202	0.09773132	-7.225238	5.00E-13	7.52E-12	PARP11
174.7818999	0.634423269	0.13874866	4.572464	4.82E-06	2.94E-05	TIGAR
1743.17367	-0.27203288	0.05448367	-4.9929247	5.95E-07	4.21E-06	RAD51AP1
25.6027125	2.592086424	0.464228	5.5836495	2.36E-08	2.06E-07	VWF
1266.179696	0.960089837	0.06289966	15.263832	1.33E-52	2.14E-50	CD9
1140.436898	-0.17088822	0.0621228	-2.750813	0.0059448	0.018913157	TNFRSF1A
544.8217589	1.14377863	0.09492903	12.048776	1.97E-33	1.50E-31	VAMP1
1899.711901	-0.13560192	0.05080125	-2.6692638	0.0076018	0.023424019	MRPL51
8995.043577	-0.45428525	0.03996547	-11.366945	6.11E-30	3.80E-28	NCAPD2
83304.80295	-0.29144538	0.03098947	-9.4046597	5.22E-21	1.68E-19	GAPDH
399.4035477	0.8103659	0.1016406	7.9728561	1.55E-15	3.03E-14	IFFO1
9.040287226	1.576428062	0.63700099	2.4747655	0.0133324	0.038322298	LAG3
3.286166613	3.526494441	1.43266218	2.4614975	0.0138358	0.039579727	CD4
1832.947756	-0.27877611	0.05002876	-5.572317	2.51E-08	2.20E-07	USP5
12661.2678	-0.26069515	0.03322672	-7.8459489	4.30E-15	8.01E-14	TPI1
2051.397315	-0.19399136	0.0479643	-4.0444948	5.24E-05	0.000263544	ATN1
359.9891299	0.413870201	0.10964135	3.7747637	0.0001602	0.000736428	C12orf57
6.151292408	2.530740275	1.03947036	2.434644	0.0149064	0.04212955	PTPN6
1899.058843	-0.34303749	0.04868687	-7.045791	1.84E-12	2.59E-11	PHB2
42.45635361	1.041185535	0.30267612	3.4399329	0.0005819	0.002379708	C1S
1199.807025	-0.3770764	0.08078676	-4.6675518	3.05E-06	1.93E-05	SLC2A3
972.5877071	-0.44698336	0.06329691	-7.0616935	1.64E-12	2.32E-11	FOXJ2
31.77797833	4.272110659	0.70385375	6.0695999	1.28E-09	1.33E-08	MFAP5
2511.133755	0.169265862	0.04653879	3.6370924	0.0002757	0.001205985	M6PR
89.04874379	1.831982141	0.22910704	7.9961844	1.28E-15	2.54E-14	OLR1
922.666934	0.631640313	0.07681632	8.2227357	1.99E-16	4.21E-15	GABARAPL1
570.1360238	-0.26134605	0.08028531	-3.2552165	0.0011331	0.004317245	MAGOHB
617.7592378	0.657120738	0.08089861	8.1227687	4.56E-16	9.44E-15	ETV6
102.1039958	-0.61621847	0.18561041	-3.3199563	0.0009003	0.003514362	BORCS5
1054.152953	-0.38985727	0.06328597	-6.1602476	7.26E-10	7.78E-09	CREBL2
2322.74583	-0.21016247	0.04891172	-4.2967708	1.73E-05	9.54E-05	CDKN1B
4771.905358	0.379161862	0.05447082	6.9608259	3.38E-12	4.64E-11	GPRC5A
634.353397	0.268292194	0.08034474	3.3392627	0.00084	0.003303776	HEBP1
460.8674821	-0.34804281	0.08576629	-4.0580372	4.95E-05	0.000250532	FAM234B

2622.535765	-0.22256417	0.04284978	-5.1940565	2.06E-07	1.57E-06	ATF7IP
68.92363877	1.101578785	0.2370873	4.6463003	3.38E-06	2.12E-05	ENSG00000261324
31.56222249	1.094533051	0.35013001	3.1260761	0.0017716	0.006461278	H4-16
170.0046285	0.527850265	0.14091271	3.7459379	0.0001797	0.000820302	H2AJ
3337.970072	-0.1827275	0.04396227	-4.1564616	3.23E-05	0.000169627	WBP11
189.9983801	1.415651682	0.14516176	9.7522353	1.80E-22	6.58E-21	ARHGDIB
17.95681997	1.65984007	0.5398805	3.0744583	0.0021089	0.007541217	PTPRO
1996.91709	0.438030211	0.05125336	8.546371	1.27E-17	3.01E-16	EPS8
4540.712109	-0.17010083	0.04581862	-3.7124824	0.0002052	0.000925106	STRAP
914.4796405	-0.16901244	0.06592349	-2.5637666	0.0103543	0.03075872	DERA
263.0524491	0.359185977	0.12307254	2.9184899	0.0035173	0.011853888	MGST1
588.3905958	-0.19107699	0.07847267	-2.4349497	0.0148939	0.042100813	PLEKHA5
1560.785061	-0.40747116	0.05128857	-7.9446774	1.95E-15	3.75E-14	AEBP2
39.34341873	1.649551507	0.30562484	5.3973084	6.76E-08	5.54E-07	PDE3A
4094.473425	0.107398588	0.04385243	2.4490909	0.0143217	0.040755175	RECQL
181.1067637	0.918848934	0.14425264	6.3697202	1.89E-10	2.18E-09	SPX
91.56992709	0.465053306	0.18642918	2.4945307	0.0126124	0.036524452	KCNJ8
1154.926234	-0.60931031	0.05951899	-10.237243	1.35E-24	5.77E-23	C2CD5
1126.729	-0.19404743	0.06650794	-2.9176582	0.0035267	0.011880602	ETNK1
3285.178219	-0.82593154	0.0566455	-14.580708	3.73E-48	5.10E-46	BCAT1
126.9582134	0.406562196	0.16792534	2.4210889	0.0154741	0.043486495	BHLHE41
55.73437678	0.733916371	0.26860083	2.7323682	0.0062881	0.019885386	SSPN
767.3974548	0.511266452	0.08440044	6.057628	1.38E-09	1.43E-08	ITPR2
1517.164999	-0.30987733	0.05963274	-5.1964295	2.03E-07	1.55E-06	INTS13
1487.68047	0.147965143	0.05585564	2.6490634	0.0080715	0.024681503	FGFR1OP2
987.0491358	-0.20821942	0.06387137	-3.2599807	0.0011142	0.004250966	MED21
849.273072	0.478407339	0.07212232	6.6332771	3.28E-11	4.06E-10	STK38L
1517.014749	-0.14261671	0.05293543	-2.6941638	0.0070565	0.021983334	ARNTL2
610.6297737	-0.338993	0.07759527	-4.3687329	1.25E-05	7.10E-05	CCDC91
2258.719382	-0.30442131	0.04413135	-6.8980741	5.27E-12	7.11E-11	ERGIC2
1996.833088	-0.62424547	0.04936649	-12.645125	1.19E-36	1.04E-34	IPO8
780.1654427	0.342255828	0.08649162	3.9570983	7.59E-05	0.0003708	CAPRIN2
12.37859752	3.439980719	0.94303669	3.6477698	0.0002645	0.00116282	TSPAN11
1726.765297	0.32898215	0.0521892	6.3036444	2.91E-10	3.28E-09	DENND5B
79.0447071	-0.56930007	0.20862966	-2.728759	0.0063573	0.020067834	AMN1
547.9254491	-0.2576893	0.08193467	-3.1450583	0.0016605	0.006093426	RESF1
548.2066707	-0.42615245	0.08521994	-5.0006189	5.71E-07	4.05E-06	BICD1
6.972532321	-2.51988636	0.79490293	-3.1700554	0.0015241	0.005630778	ENSG00000276115
984.0935134	1.117849409	0.07148616	15.637285	4.06E-55	6.92E-53	FGD4
253.223906	-0.45271223	0.11279601	-4.0135483	5.98E-05	0.00029778	ALG10
424.2344996	-0.58596993	0.0946138	-6.1932819	5.89E-10	6.40E-09	ALG10B
1059.636503	0.492958205	0.06824704	7.2231441	5.08E-13	7.63E-12	KIF21A
1198.181787	-0.42393977	0.06110921	-6.9374125	3.99E-12	5.45E-11	ZCRB1
1263.483306	-0.55628676	0.07004437	-7.9419191	1.99E-15	3.83E-14	PUS7L
2641.253567	-0.20620963	0.04988791	-4.1334587	3.57E-05	0.000185665	TWF1
104.2836885	0.420959789	0.17317529	2.4308305	0.0150643	0.042506486	PLEKHA8P1
3266.534754	-0.34456397	0.04788206	-7.1960973	6.20E-13	9.23E-12	ANO6
162.0134496	-0.519618	0.13827198	-3.7579414	0.0001713	0.000784207	ENSG00000273015

1036.302376	-0.72415863	0.06272385	-11.545188	7.81E-31	5.12E-29	ARID2
6614.816966	-0.24898732	0.04257005	-5.8488853	4.95E-09	4.73E-08	SCAF11
13033.83797	-0.17618844	0.04441216	-3.9671213	7.27E-05	0.000356956	SLC38A2
44.13670532	0.744382325	0.27684597	2.6887959	0.007171	0.022288213	ENSG00000257261
27.37167817	1.144859426	0.37165988	3.0803955	0.0020673	0.007406333	ENSG00000275481
74.24837726	1.659881304	0.25518455	6.5046309	7.79E-11	9.32E-10	SLC38A4
2842.253831	1.30059343	0.05370986	24.215172	1.54E-129	1.79E-126	AMIGO2
30.05696782	0.859861177	0.33729725	2.5492682	0.0107949	0.0319421	RPAP3-DT
40.92677447	0.996284725	0.28367525	3.5120608	0.0004446	0.001865026	RAPGEF3
231.5941378	0.531297584	0.12582074	4.2226551	2.41E-05	0.000129229	SLC48A1
441.4523603	1.053308168	0.0992484	10.612848	2.60E-26	1.28E-24	VDR
2340.290018	-0.2804345	0.04771394	-5.8774124	4.17E-09	4.02E-08	TMEM106C
19.46091693	1.93038582	0.58081506	3.3235808	0.0008887	0.003476813	COL2A1
890.4983313	-0.41442097	0.06567905	-6.309789	2.79E-10	3.16E-09	SENP1
1322.707958	-0.38731539	0.06192088	-6.2550046	3.98E-10	4.42E-09	PFKM
589.2413463	-0.32308324	0.07592686	-4.2551904	2.09E-05	0.000113261	ASB8
893.7666287	0.235930264	0.06451568	3.6569444	0.0002552	0.001125125	KANSL2
1842.44445	-0.19364768	0.0504578	-3.8378149	0.0001241	0.000581544	CCNT1
1046.233216	0.1878407	0.06224825	3.0176061	0.0025478	0.008912937	ADCY6
2606.999532	-0.16676957	0.04355346	-3.8290771	0.0001286	0.000600963	DDX23
33.34357851	1.461953053	0.35366618	4.1337089	3.57E-05	0.000185519	RND1
1315.352525	-0.38640375	0.11667567	-3.3117766	0.0009271	0.003610636	KMT2D
86.08559517	0.593852073	0.19596027	3.0304719	0.0024417	0.008578081	RHEBL1
18677.78855	-0.23543847	0.03131887	-7.5174634	5.58E-14	9.35E-13	TUBA1B
2671.420532	0.878942622	0.05419521	16.218087	3.76E-59	7.51E-57	TUBA1A
8684.937998	0.13112613	0.0444547	2.9496572	0.0031813	0.010843244	TUBA1C
119.5687128	-0.52886661	0.1627179	-3.2502054	0.0011532	0.004384442	C1QL4
316.7594212	-0.44520893	0.10021817	-4.4423972	8.90E-06	5.19E-05	DNAJC22
1425.790604	-0.3185192	0.06193541	-5.1427637	2.71E-07	2.03E-06	SPATS2
602.7825463	-0.26584273	0.07528305	-3.531243	0.0004136	0.001748349	MCRS1
64.3249171	0.688560875	0.23819708	2.8907192	0.0038436	0.012834188	BCDIN3D
4866.834825	-0.21729952	0.03993728	-5.441019	5.30E-08	4.41E-07	RACGAP1
1724.446519	-0.16796596	0.04965828	-3.382436	0.0007185	0.002866259	SMARCD1
8565.424644	0.556438973	0.04120596	13.503847	1.48E-41	1.59E-39	LIMA1
1482.65267	-0.26335694	0.05667059	-4.6471538	3.37E-06	2.11E-05	DIP2B
724.6483187	-0.37706907	0.07068972	-5.3341427	9.60E-08	7.72E-07	ATF1
704.5741981	-0.24001401	0.07186407	-3.3398331	0.0008383	0.00329815	SLC11A2
1180.756117	0.441030543	0.06328519	6.9689377	3.19E-12	4.39E-11	CSRNP2
438.9889396	-0.29197293	0.09404992	-3.1044465	0.0019064	0.006886475	SLC4A8
778.8028515	-0.42483966	0.07907876	-5.3723614	7.77E-08	6.32E-07	SCN8A
679.0199551	0.18987684	0.07428876	2.5559297	0.0105905	0.031385133	ACVR1B
753.2449172	0.435431664	0.07773918	5.6011869	2.13E-08	1.88E-07	KRT80
4747.081912	1.110510855	0.09322026	11.912763	1.02E-32	7.48E-31	KRT7
30.1617065	1.586497223	0.34442312	4.6062449	4.10E-06	2.54E-05	KRT86
214.6170945	1.762211391	0.14352766	12.277852	1.19E-34	9.50E-33	KRT81
3.740907057	3.511891948	1.42608674	2.4626075	0.0137931	0.039463914	KRT83
453.5959852	0.779988007	0.08756284	8.9077514	5.21E-19	1.42E-17	KRT8
7693.001007	-0.7087405	0.04630871	-15.304692	7.11E-53	1.16E-50	EIF4B

298.0425076	-0.81769726	0.10493151	-7.7926758	6.56E-15	1.20E-13	TNS2
158.8118076	0.663401068	0.13751929	4.8240582	1.41E-06	9.39E-06	IGFBP6
1006.367253	-0.26172669	0.06305697	-4.1506386	3.32E-05	0.000173611	ZNF740
406.4706693	-0.22684381	0.08986333	-2.5243201	0.0115922	0.033966122	RARG
848.4486338	-0.42531511	0.07284943	-5.8382765	5.27E-09	5.02E-08	ESPL1
157.8823299	0.668112424	0.15430027	4.3299497	1.49E-05	8.32E-05	PFDN5
630.0182204	-0.18937502	0.07609535	-2.4886542	0.0128228	0.037065797	AAAS
2272.199426	-0.47073015	0.04625423	-10.177018	2.51E-24	1.05E-22	SP1
558.572917	0.323610973	0.08186183	3.9531364	7.71E-05	0.000376467	PRR13
3459.435639	-0.24561631	0.04282674	-5.735115	9.74E-09	8.99E-08	PCBP2
847.4867116	-0.20456375	0.07090605	-2.8849972	0.0039142	0.013047239	ATF7
946.9148584	-0.26336138	0.06543003	-4.0250844	5.70E-05	0.000284693	ATP5MC2
6755.914621	-0.47471508	0.04368115	-10.867733	1.64E-27	8.81E-26	HNRNPA1
2555.08419	-0.25394716	0.04596626	-5.524643	3.30E-08	2.84E-07	COPZ1
357.654819	0.517868363	0.09854011	5.2554065	1.48E-07	1.15E-06	ZNF385A
1906.691843	0.494090137	0.04753511	10.394215	2.63E-25	1.21E-23	ITGA5
1213.00075	0.286629841	0.06111304	4.6901584	2.73E-06	1.74E-05	GDF11
619.245467	0.265979483	0.08044211	3.3064708	0.0009448	0.003673135	ORMDL2
27.70492124	0.841658222	0.33056156	2.5461467	0.0108919	0.032190832	MMP19
196.8933361	0.404854933	0.12658046	3.1983999	0.0013819	0.005153694	PYM1
1171.657621	0.585232493	0.0604733	9.6775351	3.76E-22	1.33E-20	DGKA
36.86851767	1.556899882	0.33191543	4.6906523	2.72E-06	1.74E-05	PMEL
162.7329458	-0.6834819	0.14186984	-4.8176687	1.45E-06	9.68E-06	IKZF4
499.7933855	-0.63450966	0.08867772	-7.1552317	8.35E-13	1.23E-11	ERBB3
4004.087655	-0.2133357	0.03858303	-5.5292625	3.22E-08	2.77E-07	PA2G4
2111.609255	-0.18729104	0.05666103	-3.305465	0.0009482	0.00368552	RPL41
4600.692365	-0.20134104	0.04117888	-4.8894253	1.01E-06	6.89E-06	ESYT1
139.1683956	0.399497578	0.15959996	2.5031184	0.0123104	0.03577515	ZC3H10
10549.98655	0.329635791	0.04492116	7.3380968	2.17E-13	3.41E-12	MYL6
3985.995042	-0.54077444	0.04016959	-13.462283	2.61E-41	2.76E-39	SMARCC2
244.858986	0.677604919	0.1295941	5.2286711	1.71E-07	1.32E-06	COQ10A
3857.241453	-0.26313963	0.03800641	-6.9235588	4.40E-12	6.00E-11	CS
1717.528042	0.42028105	0.06168355	6.8135034	9.53E-12	1.24E-10	STAT2
2443.068574	-0.20453767	0.04395651	-4.6531822	3.27E-06	2.05E-05	TIMELESS
26.55391914	1.312338733	0.36769174	3.5691276	0.0003582	0.001530747	GLS2
2673.352418	-0.32572896	0.04873526	-6.6836401	2.33E-11	2.92E-10	BAZ2A
9402.507264	-0.39955178	0.04012756	-9.9570424	2.35E-23	9.06E-22	PTGES3
5648.508073	-0.3474538	0.03945254	-8.8068812	1.29E-18	3.34E-17	NACA
583.4344635	-0.44407527	0.07503002	-5.9186342	3.25E-09	3.18E-08	PRIM1
7.393945921	2.489968157	0.9228794	2.6980428	0.0069748	0.021783868	TAC3
483.1460766	-0.66769593	0.09477972	-7.0447132	1.86E-12	2.61E-11	NAB2
626.5744634	0.297645046	0.08033704	3.704954	0.0002114	0.000950802	STAT6
1988.244054	-0.3969148	0.0467022	-8.498846	1.91E-17	4.47E-16	SHMT2
258.7416758	-0.50008969	0.11401756	-4.386076	1.15E-05	6.59E-05	R3HDM2
12.13362464	-1.89290436	0.55859646	-3.3886794	0.0007023	0.002810172	GLI1
2655.867879	-0.24111037	0.04424611	-5.4493016	5.06E-08	4.23E-07	MARS1
525.4724516	-0.33464288	0.08112368	-4.1250947	3.71E-05	0.000192313	MBD6
2285.979297	-0.24113142	0.04418876	-5.4568499	4.85E-08	4.06E-07	DCTN2

982.8360557	0.236652669	0.06909559	3.4250039	0.0006148	0.002499114	PIP4K2C
255.6330281	0.276054301	0.1143694	2.4137077	0.0157911	0.044234485	DTX3
149.3224785	-0.4078427	0.13989285	-2.9153935	0.0035524	0.011955931	ARHGEF25
5500.345448	0.231413888	0.04280097	5.4067446	6.42E-08	5.28E-07	OS9
1933.459869	-0.31275169	0.04667789	-6.7002098	2.08E-11	2.62E-10	CDK4
121.6489451	-0.57419471	0.15589652	-3.6831786	0.0002303	0.001025781	MARCHF9
193.6119725	-0.33133398	0.12561406	-2.6377142	0.0083467	0.025446867	EEF1AKMT3
19.55529131	1.858019579	0.48708705	3.8145535	0.0001364	0.000635209	AVIL
2651.251191	-0.62692339	0.0437167	-14.340592	1.22E-46	1.56E-44	CTDSP2
6.293499468	2.382336744	0.94188204	2.5293366	0.0114278	0.033568412	ENSG00000257953
85.48686842	-0.46057462	0.1941164	-2.3726724	0.0176599	0.04862349	ATP23
128.6887145	-0.54488386	0.15912645	-3.4242193	0.0006166	0.002505169	GIHCG
1990.234966	-0.24525342	0.05419574	-4.5253263	6.03E-06	3.60E-05	MON2
653.7754014	-0.4678298	0.07970247	-5.8697026	4.37E-09	4.20E-08	PPM1H
5012.184689	-0.68655631	0.03777348	-18.175618	8.05E-74	2.22E-71	XPOT
1071.516661	0.15877644	0.06003806	2.6445963	0.0081788	0.024987718	TBK1
889.9216016	0.7595221	0.07461455	10.179276	2.45E-24	1.03E-22	RASSF3
978.3572652	-0.16104261	0.06348272	-2.5367945	0.0111873	0.032942727	LEMD3
1058.532476	-0.45833958	0.07999536	-5.7295768	1.01E-08	9.27E-08	HMGA2
87.28968862	-0.5761764	0.18829741	-3.0599274	0.0022139	0.007878009	HELB
6503.148165	-0.32037535	0.04545062	-7.0488663	1.80E-12	2.53E-11	CAND1
1714.405271	0.165734065	0.05511975	3.0068001	0.0026401	0.009192312	DYRK2
8.837103141	3.242744385	0.97666811	3.3202112	0.0008995	0.003511943	LINC02421
550.2701753	-0.30830244	0.08317548	-3.7066506	0.00021	0.000944702	MDM1
3001.566277	-0.36356232	0.04126766	-8.809861	1.25E-18	3.26E-17	NUP107
734.037569	0.496411294	0.07406194	6.7026507	2.05E-11	2.58E-10	SLC35E3
5114.318237	1.011428741	0.04655905	21.723569	1.23E-104	8.90E-102	MDM2
2104.248792	-0.19686685	0.0520121	-3.78502	0.0001537	0.000708584	CPSF6
6906.393683	-0.18406811	0.04237352	-4.3439417	1.40E-05	7.85E-05	CCT2
464.964229	-0.53337743	0.08801866	-6.0598225	1.36E-09	1.41E-08	RAB3IP
1541.175489	-0.19142786	0.05265085	-3.6357982	0.0002771	0.001209927	CNOT2
53.16079718	-0.800233	0.2381995	-3.3595074	0.0007808	0.003089507	PTPRB
2005.659431	-0.37730379	0.05149755	-7.3266359	2.36E-13	3.70E-12	ZFC3H1
949.5855422	-0.18782007	0.07274685	-2.5818309	0.0098278	0.029395423	TMEM19
1248.55947	-0.22638813	0.06056566	-3.7378957	0.0001856	0.000844104	TBC1D15
1758.021986	-0.16350768	0.05243371	-3.1183695	0.0018185	0.006610479	ATXN7L3B
7244.435973	0.530261928	0.0467148	11.351048	7.33E-30	4.52E-28	GLIPR1
1766.394822	-0.4512188	0.04937097	-9.1393551	6.28E-20	1.84E-18	KRR1
17620.13329	-0.40811944	0.03853881	-10.58983	3.32E-26	1.61E-24	NAP1L1
18.72396553	2.252519236	0.51001334	4.4165888	1.00E-05	5.79E-05	LNCOG
560.7327297	-0.3637258	0.0823571	-4.4164473	1.00E-05	5.80E-05	BBS10
5070.33046	-0.10600732	0.04462029	-2.3757647	0.0175126	0.048279147	OSBPL8
1229.526747	-0.1893754	0.06248906	-3.0305368	0.0024412	0.008578081	ZDHHC17
1113.655842	0.860542788	0.06878469	12.510673	6.53E-36	5.62E-34	NAV3
5115.302263	-0.30895879	0.04020325	-7.68492	1.53E-14	2.72E-13	PAWR
3970.780689	-0.10153097	0.04216505	-2.4079412	0.0160428	0.044852682	PPP1R12A
179.5455067	-1.06606058	0.14005398	-7.6117833	2.70E-14	4.67E-13	PTPRQ
1185.237595	-0.29997849	0.06281673	-4.7754553	1.79E-06	1.18E-05	CEP290

1803.567923	-0.21205224	0.05449329	-3.891346	9.97E-05	0.000476265	TMTC3
602.2962176	1.706761731	0.09175664	18.600962	3.16E-77	1.06E-74	KITLG
471.6152447	-0.5427815	0.08168904	-6.6444835	3.04E-11	3.77E-10	POC1B
3760.331809	0.225337874	0.04434745	5.0811912	3.75E-07	2.74E-06	ATP2B1
5.553344595	2.878212073	0.93349793	3.0832549	0.0020475	0.007345981	DCN
1005.634818	0.26863088	0.06524302	4.1173886	3.83E-05	0.000198147	BTG1
11.53378784	1.708114952	0.58923252	2.898881	0.003745	0.012533685	PLEKHG7
1675.459105	-0.47095674	0.05603739	-8.4043304	4.30E-17	9.69E-16	EEA1
440.1125292	0.282963827	0.08797165	3.2165342	0.0012975	0.00486909	NUDT4
1737.233011	-0.31566142	0.05455542	-5.7860688	7.21E-09	6.78E-08	MRPL42
56.91474159	-0.58427492	0.24300501	-2.404374	0.0162002	0.045227371	CRADD
341.412216	2.471723882	0.13035432	18.961581	3.54E-80	1.31E-77	PLXNC1
464.9011692	-0.23598457	0.0844804	-2.7933648	0.0052163	0.01681705	CEP83
962.22375	-0.20704488	0.06170921	-3.3551701	0.0007932	0.003134793	NDUFA12
682.1559964	0.301437492	0.07387988	4.0801026	4.50E-05	0.00022977	FGD6
4102.975036	-0.13861004	0.04609923	-3.0067757	0.0026403	0.009192312	METAP2
1301.464528	0.93759499	0.06204867	15.110637	1.38E-51	2.10E-49	NTN4
1027.198528	-0.29438096	0.0617293	-4.7689017	1.85E-06	1.21E-05	SNRPF
15.69024711	1.614788595	0.54196923	2.9794839	0.0028873	0.00996446	AMDHD1
1917.638432	-0.33520342	0.05253755	-6.3802638	1.77E-10	2.05E-09	LTA4H
1992.955726	-0.21021183	0.05487651	-3.8306341	0.0001278	0.000597493	ELK3
2021.737612	-0.2919972	0.04973036	-5.8716087	4.32E-09	4.16E-08	NEDD1
276.5684036	-0.64535805	0.10796851	-5.9772802	2.27E-09	2.28E-08	TMPO-AS1
9696.34439	-0.58953647	0.03950308	-14.923811	2.31E-50	3.40E-48	TMPO
7432.899874	-0.15939659	0.03660279	-4.3547668	1.33E-05	7.51E-05	SLC25A3
1546.356458	-0.20100132	0.05251423	-3.827559	0.0001294	0.000604356	APAF1
754.7309343	-0.20389477	0.07174905	-2.8417767	0.0044863	0.014720111	UHRF1BP1L
663.1013747	-0.37772837	0.07084892	-5.3314624	9.74E-08	7.82E-07	ACTR6
1814.631291	-0.589104	0.0604382	-9.7472124	1.90E-22	6.88E-21	GAS2L3
1858.790448	-0.2930022	0.06879566	-4.2590216	2.05E-05	0.000111615	UTP20
1786.851486	-0.14623637	0.05557227	-2.6314632	0.0085018	0.025842757	ARL1
2188.606559	0.383955911	0.05443207	7.0538541	1.74E-12	2.45E-11	GNPTAB
1809.844915	1.463920851	0.0538855	27.167251	1.58E-162	4.59E-159	DRAM1
412.9979639	-0.35039508	0.0899509	-3.8954038	9.80E-05	0.000469005	WASHC3
927.1410898	-0.36249356	0.06561654	-5.5244236	3.31E-08	2.84E-07	NUP37
1107.037375	-0.35552533	0.06132079	-5.7977944	6.72E-09	6.33E-08	PARPBP
29441.22089	-0.12333038	0.0409308	-3.013144	0.0025856	0.009025074	HSP90B1
800.22	0.412809497	0.0720304	5.7310457	9.98E-09	9.19E-08	TDG
43.12503596	1.029108292	0.27871257	3.6923642	0.0002222	0.000993748	GLT8D2
728.9499706	-0.24732878	0.07232269	-3.4197951	0.0006267	0.002540331	HCFC2
11946.8317	-0.26816037	0.04814446	-5.569911	2.55E-08	2.22E-07	TXNRD1
159.5925167	1.240844183	0.15325269	8.0967204	5.65E-16	1.16E-14	CHST11
246.0565708	0.301195772	0.11998211	2.5103391	0.0120615	0.035143245	SLC41A2
929.5164052	-0.45852566	0.06789678	-6.7532757	1.45E-11	1.85E-10	APPL2
2506.223801	0.255023514	0.05714382	4.462836	8.09E-06	4.75E-05	C12orf75
957.8875377	0.610402951	0.06933347	8.8038713	1.32E-18	3.43E-17	NUAK1
3052.971904	0.164876097	0.04770062	3.4564772	0.0005473	0.002249951	CKAP4
598.3572775	-0.35092353	0.0829093	-4.2326198	2.31E-05	0.000124091	POLR3B

4573.094026	-0.21771631	0.0424505	-5.1287105	2.92E-07	2.17E-06	TMEM263
1189.322383	-0.19696433	0.05981599	-3.2928373	0.0009918	0.003836237	CRY1
1860.387802	-0.12154293	0.05007838	-2.4270541	0.015222	0.04288875	PWP1
369.6543631	0.447165542	0.09688827	4.6152702	3.93E-06	2.44E-05	FICD
1593.374773	-0.26576291	0.0518986	-5.1208108	3.04E-07	2.26E-06	SART3
23.41481681	1.291307969	0.38642574	3.3416717	0.0008328	0.003279442	SELPLG
15661.75619	-0.11531944	0.03580628	-3.2206487	0.001279	0.004808031	CORO1C
417.50622	-0.49894358	0.08845602	-5.6405833	1.69E-08	1.51E-07	USP30
1616.730076	-0.48068938	0.0511819	-9.3917841	5.90E-21	1.89E-19	UNG
279.48374	-0.5356587	0.11102193	-4.8248009	1.40E-06	9.36E-06	MMAB
191.2595937	-0.46225332	0.12712231	-3.6362879	0.0002766	0.00120854	MVK
78.97282934	1.565005123	0.2387617	6.554674	5.58E-11	6.77E-10	FAM222A
1154.758045	-0.21369344	0.06496037	-3.289597	0.0010033	0.003875995	GLTP
3444.453388	0.267738476	0.04352239	6.1517412	7.66E-10	8.18E-09	ANKRD13A
432.8616325	-0.47611299	0.08942136	-5.3243765	1.01E-07	8.11E-07	IFT81
2163.074018	-0.12461229	0.05220713	-2.3868826	0.0169919	0.047062819	ARPC3
781.2222	-0.35509809	0.06850641	-5.1834285	2.18E-07	1.66E-06	GPN3
548.6028714	-0.29474388	0.08211596	-3.5893614	0.0003315	0.001424412	FAM216A
18.72653081	1.334801925	0.44258427	3.0159271	0.0025619	0.008955232	ENSG00000278993
26.14761434	1.105189773	0.36853078	2.9989076	0.0027095	0.009414207	RAD9B
204.3105144	-0.36585241	0.12476184	-2.9324063	0.0033635	0.011397239	TCTN1
6546.800811	-0.3093832	0.04082818	-7.5776868	3.52E-14	6.02E-13	PPP1CC
117.6790756	0.433860693	0.17209453	2.5210604	0.0117002	0.034230254	PHETA1
1851.280329	-0.26248887	0.04857362	-5.4039384	6.52E-08	5.37E-07	ATXN2
64.54128709	0.696272944	0.23051923	3.0204549	0.002524	0.00884036	ALDH2
1634.525103	-0.14000802	0.05663995	-2.4718952	0.0134399	0.038573978	ERP29
1195.362052	-0.25754558	0.05984584	-4.3034835	1.68E-05	9.28E-05	NAA25
777.9302977	0.282515979	0.0705322	4.0054894	6.19E-05	0.000307501	TRAFD1
1086.912606	-0.32867208	0.07441347	-4.416836	1.00E-05	5.79E-05	HECTD4
6790.338775	-0.38697206	0.04156096	-9.3109519	1.27E-20	3.93E-19	RPL6
8006.680309	-0.35174306	0.04583993	-7.6732896	1.68E-14	2.95E-13	PTPN11
170.264574	4.201002246	0.45362463	9.2609659	2.03E-20	6.20E-19	OAS1
576.8469124	2.552808279	0.25141745	10.153664	3.19E-24	1.32E-22	OAS3
153.4959526	4.621770391	0.59280944	7.7963846	6.37E-15	1.17E-13	OAS2
917.9999428	-0.16819086	0.06421997	-2.6189807	0.0088193	0.026667974	DDX54
214.3505538	0.330182674	0.12605948	2.619261	0.0088121	0.026650705	TPCN1
237.2073566	0.601038351	0.11782198	5.1012413	3.37E-07	2.49E-06	SLC8B1
29.85433207	1.551275833	0.38096153	4.0720013	4.66E-05	0.000237424	SDSL
810.3223757	0.829099111	0.07474607	11.09221	1.37E-28	7.99E-27	TBX3
3254.111263	-0.46556898	0.04847574	-9.6041647	7.68E-22	2.62E-20	MED13L
112.2268571	-0.58015835	0.16504142	-3.5152288	0.0004394	0.001843807	RNFT2
7.059800052	3.332840431	1.18136746	2.8211717	0.0047849	0.01558799	HRK
1309.403192	-0.3210086	0.05720951	-5.6111055	2.01E-08	1.78E-07	FBXO21
1321.086325	-0.31809391	0.0581628	-5.4690263	4.53E-08	3.81E-07	RFC5
682.8354728	0.202243263	0.07920552	2.5533987	0.0106677	0.031603382	TAOK3
2610.920872	-0.29185327	0.04540554	-6.4277017	1.30E-10	1.52E-09	RPS2P5
1190.495963	-0.28469865	0.05718304	-4.9787252	6.40E-07	4.51E-06	SUDS3
10.45174179	2.268888777	0.73402266	3.0910337	0.0019946	0.007172511	HSPB8

539.7274599	0.459087973	0.08288687	5.5387298	3.05E-08	2.63E-07	PRKAB1
3130.017742	-0.38497246	0.0462007	-8.3326107	7.91E-17	1.74E-15	CIT
1036.594265	0.212244434	0.059588	3.5618655	0.0003682	0.001570507	RAB35
2781.132719	-0.24341152	0.04422924	-5.5034076	3.73E-08	3.17E-07	GCN1
8681.588187	-0.37632466	0.03633137	-10.35812	3.84E-25	1.73E-23	RPLP0
21.08026752	-0.96084209	0.36014461	-2.6679341	0.0076319	0.023506237	MSI1
708.0652765	0.221918625	0.07689568	2.8859699	0.0039021	0.013016962	TRIAP1
858.4962165	-0.25600219	0.06613801	-3.870727	0.0001085	0.000514172	GATC
3023.298768	-0.21873187	0.0493392	-4.433227	9.28E-06	5.39E-05	SRSF9
2409.487411	-0.17276859	0.04616443	-3.7424616	0.0001822	0.000831082	RNF10
1061.265124	-0.44751002	0.06033039	-7.4176549	1.19E-13	1.92E-12	UNC119B
899.3990827	-0.21865607	0.06404619	-3.4140371	0.0006401	0.002586191	SPPL3
13.85907443	2.962497683	0.60247372	4.9172231	8.78E-07	6.04E-06	OASL
219.2097048	0.333384265	0.1297466	2.5695029	0.0101845	0.030305894	P2RX4
674.4521672	-0.23074738	0.07388748	-3.1229564	0.0017904	0.006521951	CAMKK2
1070.780657	0.187353214	0.06059102	3.0920952	0.0019875	0.00715135	RNF34
631.409625	-0.38709998	0.07295896	-5.3057227	1.12E-07	8.91E-07	KDM2B
594.125101	0.209707131	0.07739455	2.7095853	0.0067367	0.0211351	TMEM120B
640.1010391	0.56155663	0.08193838	6.8534013	7.21E-12	9.51E-11	CFAP251
3311.870131	-0.21456038	0.04012326	-5.3475314	8.92E-08	7.20E-07	CLIP1
2035.980061	-0.76810041	0.05417179	-14.178973	1.24E-45	1.50E-43	KNTC1
3392.870022	-0.20488709	0.04629312	-4.425865	9.61E-06	5.57E-05	DENR
451.4751644	0.302681728	0.08906819	3.3983145	0.000678	0.002724316	HIP1R
425.9618524	0.284017528	0.09041913	3.1411221	0.001683	0.006166812	VPS37B
1859.172407	-0.34288483	0.05060755	-6.7753685	1.24E-11	1.60E-10	MPHOSPH9
531.6591756	-0.34387364	0.08373523	-4.1066782	4.01E-05	0.000206453	MTRFR
3030.91017	-0.20526299	0.05203477	-3.9447273	7.99E-05	0.000388725	SBNO1
1005.676483	-0.2398811	0.06424263	-3.7339866	0.0001885	0.000856201	GTF2H3
247.2795237	-0.61910939	0.11869943	-5.2157741	1.83E-07	1.41E-06	TCTN2
399.8113842	0.292536192	0.09081296	3.2213045	0.0012761	0.004801194	ATP6V0A2
159.66783	0.518208536	0.14866236	3.4858086	0.0004907	0.00204223	CCDC92
4125.680443	-0.34693987	0.04159593	-8.3407163	7.38E-17	1.63E-15	ZNF664
869.8688555	0.50206476	0.07277285	6.899067	5.23E-12	7.07E-11	SCARB1
530.3043905	0.257674866	0.08479249	3.0388879	0.0023745	0.008364009	DHX37
862.3730167	0.288516335	0.07349931	3.925429	8.66E-05	0.000418672	AACS
55.60847579	0.719726788	0.24275937	2.9647744	0.003029	0.010399836	ENSG00000279233
1492.561868	-0.23298367	0.05598448	-4.1615758	3.16E-05	0.000166201	STX2
8568.865066	-0.30043137	0.04116602	-7.2980434	2.92E-13	4.51E-12	RAN
69.28097547	0.737507935	0.2238043	3.2953251	0.0009831	0.00380582	MMP17
589.2437037	0.228735195	0.08153414	2.8053918	0.0050255	0.016274531	ULK1
1838.162561	-0.51033607	0.06338858	-8.0509148	8.22E-16	1.67E-14	EP400
344.2428913	0.303982741	0.10204726	2.9788429	0.0028934	0.009981368	DDX51
239.644333	0.297380352	0.11980926	2.4821149	0.0130605	0.037646674	CHFR
185.5398338	-0.5766708	0.13273632	-4.3444837	1.40E-05	7.83E-05	ZNF891
193.76136	0.463029552	0.13928312	3.3243766	0.0008862	0.003468467	ZNF10
1072.040277	0.180641638	0.06408649	2.8187163	0.0048216	0.015687217	PSPC1
1271.389202	-0.17935013	0.06215942	-2.8853253	0.0039101	0.013036147	ZMYM2
93.78232898	-0.97403014	0.18240242	-5.3400067	9.29E-08	7.49E-07	IFT88

842.9043703	-0.2271461	0.06674585	-3.4031492	0.0006661	0.002682138	XPO4
1407.294046	0.238308528	0.06045513	3.9419075	8.08E-05	0.000392665	SAP18
20.26921401	1.053597456	0.41477753	2.5401508	0.0110805	0.032664748	MIPEPP3
1562.756857	-0.22762021	0.05853655	-3.8885145	0.0001009	0.000481457	ZDHHC20
18.93717406	2.467286183	0.57539598	4.2879795	1.80E-05	9.90E-05	FGF9
3485.438871	-0.46658875	0.15800493	-2.9530013	0.003147	0.010747535	SACS
43.8301456	3.389537764	0.46369796	7.3097965	2.68E-13	4.15E-12	ENSG00000289688
55.23063331	0.754549621	0.28647637	2.6338983	0.0084411	0.025703084	TNFRSF19
122.5530986	-0.52568141	0.15269987	-3.4425792	0.0005762	0.002358209	MIPEP
5.217775053	2.816971363	1.0541707	2.6722156	0.0075352	0.023270737	ANKRD20A19P
1110.775768	-0.31754247	0.06005137	-5.2878477	1.24E-07	9.77E-07	PARP4
664.599343	-0.44846849	0.07103044	-6.3137509	2.72E-10	3.08E-09	CENPJ
2208.205119	-0.22151206	0.04908641	-4.5126966	6.40E-06	3.80E-05	NUP58
297.042179	0.831947909	0.11796359	7.052582	1.76E-12	2.47E-11	SHISA2
12.98153362	2.131930603	0.63815208	3.3407877	0.0008354	0.003288319	RASL11A
201.7531166	-0.3483541	0.1217332	-2.8616194	0.0042148	0.013916051	MTIF3
544.5696451	-0.2875229	0.07766703	-3.7019943	0.0002139	0.000960475	POLR1D
629.2122653	-0.3604708	0.07586387	-4.7515477	2.02E-06	1.31E-05	PAN3
22.00096346	1.385660519	0.4232892	3.2735551	0.001062	0.004070713	FLT1
2561.347458	-0.18883171	0.04440232	-4.2527439	2.11E-05	0.000114256	SLC7A1
2140.181981	-0.40541324	0.05404503	-7.5013978	6.31E-14	1.05E-12	HMGB1
1067.565143	-0.288876	0.07764614	-3.7204167	0.0001989	0.000899317	BRCA2
1068.603487	-0.2794466	0.06398389	-4.3674528	1.26E-05	7.13E-05	N4BP2L2
1193.334573	-0.48314662	0.05658809	-8.5379565	1.37E-17	3.23E-16	PDS5B
67.38018372	0.94926849	0.23166592	4.097575	4.18E-05	0.000214296	ENSG00000276672
156.0806088	-1.0234195	0.15063191	-6.7941747	1.09E-11	1.41E-10	NBEA
12.30315874	1.574219638	0.54257428	2.9013901	0.0037151	0.012450525	MAB21L1
875.2259191	-0.26608402	0.06484479	-4.1033986	4.07E-05	0.000209093	SPART
6.950641993	3.615701191	1.17879225	3.067293	0.0021601	0.007705358	CCNA1
652.5077514	-0.40377488	0.07241017	-5.5762182	2.46E-08	2.15E-07	EXOSC8
620.4692976	-0.18020095	0.07568588	-2.3809057	0.0172701	0.047739247	PROSER1
604.5354395	-0.38262317	0.07473526	-5.1197141	3.06E-07	2.27E-06	COG6
175.0657864	0.513070141	0.15040244	3.4113152	0.0006465	0.002609118	FOXO1
265.0955958	-0.39291277	0.11692456	-3.3603956	0.0007783	0.003080983	MRPS31
297.8311889	-0.8077516	0.1114554	-7.2473082	4.25E-13	6.46E-12	VWA8
682.7812028	-0.63354533	0.07762268	-8.1618587	3.30E-16	6.87E-15	AKAP11
97.87047846	2.187181386	0.22183316	9.8595783	6.23E-23	2.37E-21	EPSTI1
42.71408769	0.846770828	0.28884293	2.9315962	0.0033722	0.011424782	CCDC122
97.62715441	0.487569962	0.1942693	2.5097634	0.0120812	0.035185435	LACC1
1311.576514	0.297299172	0.0577404	5.1488939	2.62E-07	1.97E-06	TSC22D1
15594.54414	-0.18387576	0.03350975	-5.4872307	4.08E-08	3.46E-07	TPT1
424.4715672	0.258195534	0.09211695	2.8029102	0.0050644	0.016388075	SLC25A30
2107.000226	-0.286033	0.04910515	-5.824908	5.71E-09	5.42E-08	ZC3H13
50.39772046	3.061329951	0.37412324	8.1826778	2.78E-16	5.83E-15	LCP1
1006.093311	-0.33938945	0.06579602	-5.158206	2.49E-07	1.88E-06	ESD
765.2238585	-0.16611991	0.06708059	-2.476423	0.0132706	0.038157435	SUCLA2
3013.535699	0.59882362	0.04663683	12.840144	9.77E-38	9.08E-36	ITM2B
2936.685499	-0.1726834	0.04357292	-3.9630898	7.40E-05	0.000362325	RB1

153.2561097	0.394576268	0.15180084	2.5993023	0.0093413	0.028095126	RCBTB2
91.42240658	-0.55095398	0.17924752	-3.0737049	0.0021142	0.007557179	CAB39L
18.93793546	1.39040006	0.4326962	3.2133401	0.001312	0.004919739	PHF11
496.3855109	-0.40292971	0.08601262	-4.6845419	2.81E-06	1.79E-05	RCBTB1
1547.473122	-0.19601967	0.06278924	-3.1218671	0.0017971	0.00654202	KPNA3
239.2085959	0.560148365	0.1312497	4.2678069	1.97E-05	0.000107681	THSD1
432.8286768	-0.25451105	0.09245342	-2.7528572	0.0059078	0.018802352	VPS36
4149.433676	-0.28203674	0.04617818	-6.1075756	1.01E-09	1.06E-08	CKAP2
2780.2035	-0.30350728	0.04482838	-6.7704272	1.28E-11	1.65E-10	DIAPH3
124.8060805	-0.70311028	0.15613219	-4.5033012	6.69E-06	3.96E-05	TDRD3
126.1927551	1.021198236	0.17042649	5.9920159	2.07E-09	2.09E-08	PCDH9
1168.618651	-0.16258359	0.06164297	-2.6375042	0.0083519	0.025458165	MZT1
384.7619754	-0.24130914	0.0943747	-2.5569261	0.0105602	0.031316728	BORA
236.908377	-0.3709854	0.12134558	-3.0572634	0.0022337	0.007938622	PIBF1
567.8778007	0.829959831	0.08780343	9.4524758	3.31E-21	1.08E-19	KLF5
930.6482682	-1.09172264	0.06830978	-15.981937	1.71E-57	3.30E-55	KLF12
16.18474542	1.183581707	0.47250698	2.5048978	0.0122487	0.03561948	ENSG00000261553
133.436368	0.608022467	0.17545088	3.4654854	0.0005293	0.002183135	KCTD12
315.0475112	0.338061964	0.10742353	3.1470011	0.0016495	0.006058196	CLN5
969.4387953	-0.32933509	0.08262979	-3.98567	6.73E-05	0.000332152	MYCBP2
10.65295871	1.48979983	0.58981725	2.5258668	0.0115413	0.033839756	POU4F1
1282.166577	-0.14909416	0.05667113	-2.6308664	0.0085168	0.025883662	RBM26
2022.626992	0.423486524	0.05341454	7.9282998	2.22E-15	4.26E-14	NDFIP2
150.852775	-0.73247756	0.14754652	-4.9643838	6.89E-07	4.82E-06	SLITRK5
27.90252559	2.041277216	0.42434994	4.8103629	1.51E-06	1.00E-05	GPC5
512.288267	-0.72648869	0.09051138	-8.0264897	1.00E-15	2.02E-14	ABCC4
1809.492779	0.219113148	0.06372532	3.4384	0.0005852	0.002391535	DNAJC3
5778.517681	-0.32896149	0.03962082	-8.3027437	1.02E-16	2.22E-15	IPO5
241.7086979	0.266476065	0.11007703	2.4208144	0.0154858	0.043512315	ZIC2
65.03187923	0.534541873	0.2246195	2.3797661	0.0173236	0.047856699	PCCA-DT
241.5782139	-0.42358945	0.11625878	-3.6435049	0.000269	0.001179881	PCCA
76.2566906	-0.68053656	0.19824535	-3.4327997	0.0005974	0.002436408	TMTC4
130.9770823	-0.43855045	0.15527378	-2.8243689	0.0047374	0.015459387	NALCN
1167.183195	-0.1459796	0.05869888	-2.486923	0.0128853	0.037197192	TPP2
1561.427776	0.205809897	0.05376459	3.8279821	0.0001292	0.00060348	EFNB2
116.1963856	1.444337426	0.17392687	8.30428	1.00E-16	2.19E-15	ENSG00000275216
889.6447575	0.184794453	0.06735704	2.7435061	0.0060787	0.019300431	IRS2
10652.43205	0.935174086	0.09132036	10.240586	1.30E-24	5.60E-23	COL4A1
9664.707198	0.632511104	0.05388494	11.738179	8.12E-32	5.60E-30	COL4A2
588.0417959	0.354324314	0.09341191	3.7931385	0.0001488	0.000687627	ANKRD10
625.4924459	-0.44959933	0.07570971	-5.938463	2.88E-09	2.84E-08	TUBGCP3
804.6969397	-0.1881453	0.07047852	-2.669541	0.0075955	0.023415303	ATP11A
644.661426	-0.2332109	0.07868536	-2.9638411	0.0030383	0.010429369	PCID2
1855.188201	-0.24295632	0.05226739	-4.6483347	3.35E-06	2.10E-05	CUL4A
3902.984923	0.389198807	0.04320202	9.0088106	2.08E-19	5.85E-18	LAMP1
27.24249206	1.349142422	0.38068226	3.5440118	0.0003941	0.001671916	ADPRHL1
805.055362	-0.17639759	0.06927104	-2.5464839	0.0108814	0.032165216	TMCO3
11.16714842	1.760371341	0.58353168	3.0167537	0.002555	0.008934444	TMEM255B

24.59311906	1.365395017	0.40423936	3.3776895	0.000731	0.002910181	GAS6-AS1
567.8008436	0.375169638	0.08061099	4.6540757	3.25E-06	2.05E-05	GAS6
134.5387304	0.541474317	0.1590712	3.4039746	0.0006641	0.002674666	GAS6-DT
415.5976864	-0.31026	0.09157948	-3.3878768	0.0007044	0.002817112	TTC5
14.43047535	-1.37665987	0.45790158	-3.0064536	0.0026431	0.009200214	ENSG00000258768
1411.899002	-0.23658722	0.05296731	-4.4666651	7.94E-06	4.67E-05	PARP2
2782.248907	-0.14833576	0.04412741	-3.3615333	0.0007751	0.003070425	APEX1
1005.915226	0.568716955	0.06253877	9.0938303	9.56E-20	2.76E-18	PNP
17.22684992	-1.20072281	0.45702325	-2.6272686	0.0086073	0.026117883	ANG
81.45850892	-0.54565782	0.18763474	-2.9080853	0.0036365	0.012210577	NDRG2
4.215258553	2.743310948	1.09814093	2.4981411	0.0124847	0.03620278	RNASE7
395.1516506	-0.35157628	0.09229785	-3.809149	0.0001394	0.000647694	ARHGEF40
213.5662287	-0.36971374	0.15618874	-2.3670961	0.0179283	0.049253202	LINC00641
6759.97575	-0.38955768	0.04053766	-9.6097725	7.27E-22	2.50E-20	HNRNPC
8755.430665	-0.26945955	0.03611371	-7.4614204	8.56E-14	1.40E-12	SUPT16H
4046.876765	-0.16119221	0.04651725	-3.4652137	0.0005298	0.002183788	CHD8
2127.518588	-0.12638578	0.04521675	-2.7951094	0.0051882	0.016735869	TOX4
428.4675235	-0.5417563	0.08924986	-6.0701084	1.28E-09	1.33E-08	SALL2
1249.159523	-0.16486557	0.05830733	-2.8275272	0.0046909	0.015319213	OXA1L
2276.7443	0.353716283	0.04626543	7.6453691	2.08E-14	3.64E-13	MMP14
1935.230632	0.27405481	0.04743002	5.7780877	7.56E-09	7.09E-08	LRP10
2064.098788	-0.2335918	0.04748451	-4.9193267	8.68E-07	5.98E-06	PRMT5
3445.060266	-0.26822118	0.04743442	-5.6545688	1.56E-08	1.40E-07	AJUBA
1305.473226	-0.15608247	0.0564308	-2.765909	0.0056764	0.018152491	PSMB5
532.780055	0.319072173	0.09825582	3.2473617	0.0011648	0.004423655	CDH24
164.2058094	0.40466558	0.14937171	2.709118	0.0067462	0.021161076	SLC22A17
93.40898426	0.567774434	0.1924384	2.9504218	0.0031734	0.01082493	ZFHX2
778.862297	0.490156537	0.07966916	6.1523997	7.63E-10	8.15E-09	AP1G2
543.2111741	2.269854435	0.10093937	22.487304	5.53E-112	4.58E-109	DHRS2
140.1925773	-0.48789142	0.15918476	-3.0649379	0.0021772	0.007758999	DHRS4-AS1
737.1526843	-0.46101187	0.07118752	-6.4760206	9.42E-11	1.12E-09	DCAF11
1362.698514	0.517639511	0.06133556	8.439468	3.19E-17	7.27E-16	PSME2
46.7310228	1.264538834	0.27281288	4.6351875	3.57E-06	2.23E-05	IRF9
66.70898142	1.437944895	0.22220871	6.4711454	9.73E-11	1.15E-09	REC8
698.6786312	-0.26299361	0.07222256	-3.6414333	0.0002711	0.001187023	GMPR2
32.54572798	1.127146978	0.32707778	3.4461129	0.0005687	0.002328682	TGM1
221.4759827	0.344010686	0.12803515	2.6868457	0.007213	0.022395021	DHRS1
1241.520518	0.150769545	0.05574323	2.7047151	0.0068363	0.021412687	NOP9
108.7592885	0.677367354	0.18364427	3.6884752	0.0002256	0.00100647	LTB4R2
480.6800674	0.60362304	0.10636414	5.6750617	1.39E-08	1.25E-07	LTB4R
143.1915656	0.419204983	0.15623559	2.6831593	0.007293	0.022611103	NYNRIN
358.8287119	0.336490672	0.10268806	3.2768237	0.0010498	0.00402921	SDR39U1
120.0314745	-0.57922602	0.16554843	-3.4988312	0.0004673	0.00195252	NOVA1
344.3638275	0.328112641	0.10208887	3.2139903	0.001309	0.004910315	PRKD1
1914.552046	-0.16965429	0.06077039	-2.791726	0.0052428	0.016893055	G2E3
1698.681571	-0.21001913	0.05293712	-3.9673318	7.27E-05	0.000356742	SCFD1
446.4525859	0.398337311	0.096699	4.1193528	3.80E-05	0.000196582	COCH
5.73777419	2.228912784	0.83846038	2.65834	0.0078527	0.024092765	ENSG00000258525

170.1795943	-0.44247	0.13753435	-3.2171599	0.0012947	0.004861259	AP4S1
4927.532672	-0.27266586	0.04509017	-6.0471246	1.47E-09	1.52E-08	HECTD1
558.1222519	0.252043452	0.0862082	2.9236598	0.0034594	0.011683708	HEATR5A
286.7332374	-0.48851481	0.10647804	-4.5879394	4.48E-06	2.75E-05	NUBPL
3137.058991	-0.20551219	0.04713449	-4.3601234	1.30E-05	7.34E-05	ARHGAP5
112.8080058	0.566960567	0.17960521	3.1567044	0.0015956	0.005878814	EGLN3
1109.526495	0.280914844	0.06513535	4.3127863	1.61E-05	8.93E-05	SPTSSA
909.7132537	-0.24871868	0.06310715	-3.9412121	8.11E-05	0.000393695	EAPP
3891.807011	-0.25576629	0.04318988	-5.9219032	3.18E-09	3.12E-08	SNX6
4908.262478	-0.27544952	0.05074173	-5.4284616	5.68E-08	4.71E-07	CFL2
3211.424132	0.201026373	0.05172478	3.8864615	0.0001017	0.000485146	BAZ1A
2394.429967	-0.12309138	0.04533138	-2.7153678	0.0066202	0.020799573	SRP54
1352.35181	0.264999163	0.05619217	4.7159443	2.41E-06	1.55E-05	FAM177A1
496.3945794	0.723091998	0.08840062	8.1797165	2.85E-16	5.97E-15	NFKBIA
358.162007	-0.46606613	0.1143282	-4.0765631	4.57E-05	0.00023302	RALGAPA1
311.8534239	-0.68809207	0.10237946	-6.7209976	1.80E-11	2.28E-10	PTCSC3
1060.772773	-0.41648934	0.06149271	-6.7729867	1.26E-11	1.63E-10	MBIP
78.34790781	-0.60196677	0.20553632	-2.928761	0.0034032	0.011509324	NKX2-1
261.1489243	-0.50313725	0.11925218	-4.2191032	2.45E-05	0.00013108	MIPOL1
6.057953411	2.416583892	0.88046688	2.7446619	0.0060573	0.019243135	ENSG00000289687
363.5598058	0.565323163	0.10276726	5.5010046	3.78E-08	3.21E-07	FOXA1
27.85569794	1.218780892	0.35657442	3.4180267	0.0006308	0.002553918	SSTR1
7317.951356	-0.12250719	0.04072363	-3.008258	0.0026275	0.00915309	SEC23A
1076.762629	0.202725944	0.06066617	3.3416639	0.0008328	0.003279442	TRAPPC6B
2781.73408	-0.11562651	0.04702034	-2.4590744	0.0139296	0.039821679	FKBP3
942.1845742	-0.17230637	0.06456326	-2.6687989	0.0076123	0.023450475	FANCM
1507.358083	-0.47701632	0.05215199	-9.1466556	5.87E-20	1.73E-18	MIS18BP1
48.0275062	-0.80551572	0.25220961	-3.1938344	0.001404	0.005230286	MDGA2
1316.732582	-0.25130985	0.06520412	-3.8542017	0.0001161	0.000546298	RPS29
408.537427	-0.72832134	0.09085482	-8.0163206	1.09E-15	2.18E-14	POLE2
646.9608651	-0.24226862	0.07321886	-3.308828	0.0009369	0.003645611	KLHDC2
4439.417574	-0.12441756	0.04578836	-2.7172309	0.0065831	0.020707681	ARF6
48.72046602	0.917555968	0.26461703	3.4674865	0.0005254	0.002169003	LINC01588
2950.448364	0.166177004	0.04473361	3.7148131	0.0002034	0.000917811	MAP4K5
129.3931107	1.049955032	0.1737497	6.0429172	1.51E-09	1.56E-08	ATL1
6162.756624	-0.43696382	0.05021204	-8.7023708	3.25E-18	8.18E-17	NIN
142.8596026	0.593715901	0.15775599	3.7635079	0.0001675	0.00076816	TRIM9
4727.116209	-0.14147659	0.03815258	-3.7081791	0.0002088	0.000939992	FRMD6
39.84744605	1.418231231	0.34935762	4.05954	4.92E-05	0.000249213	GNG2
2373.71596	-0.13091167	0.05403636	-2.4226588	0.0154074	0.043320047	RTRAF
563.3312214	1.605515305	0.08786599	18.272319	1.37E-74	3.92E-72	NID2
88.09091631	-0.6066339	0.18776088	-3.2308855	0.0012341	0.004661282	PTGER2
240.6855585	-0.28777698	0.11979959	-2.4021532	0.0162989	0.045459107	GPR137C
3428.358963	-0.37716654	0.04178301	-9.0267917	1.77E-19	4.99E-18	ERO1A
1826.607605	-0.26815945	0.04793341	-5.5944162	2.21E-08	1.95E-07	PSMC6
8253.880532	0.335404119	0.0413297	8.1153283	4.84E-16	1.00E-14	FERMT2
440.4739807	1.188811735	0.10427636	11.400587	4.15E-30	2.62E-28	BMP4
1244.414415	-0.27203756	0.06242675	-4.3577079	1.31E-05	7.42E-05	CDKN3

2925.9699	-0.19865093	0.05213167	-3.8105617	0.0001387	0.00064452	GMFB
2245.743064	0.23722047	0.04894531	4.8466438	1.26E-06	8.44E-06	SAMD4A
365.0511387	0.901912617	0.1097126	8.2206837	2.02E-16	4.28E-15	GCH1
2753.511166	-0.41987925	0.04467784	-9.3979312	5.56E-21	1.79E-19	WDHD1
4164.03912	-0.14571048	0.03942075	-3.6962885	0.0002188	0.00098079	MAPK1IP1L
676.702476	0.694114053	0.08199801	8.4650109	2.56E-17	5.91E-16	LGALS3
4706.083425	-0.34044121	0.04126453	-8.2502142	1.58E-16	3.39E-15	DLGAP5
15311.85306	-0.12035829	0.03711693	-3.2426792	0.0011841	0.004487198	KTN1
256.835775	0.278085582	0.1152848	2.4121618	0.0158582	0.044379605	PELI2
3484.346923	-0.14928256	0.04534455	-3.2921827	0.0009941	0.003843469	EXOC5
1033.44762	0.17692274	0.06332661	2.7938135	0.0052091	0.016796847	AP5M1
1785.709398	-0.1804366	0.05230983	-3.4493823	0.0005619	0.002302292	PSMA3
920.9825085	-0.37599085	0.06322833	-5.9465572	2.74E-09	2.71E-08	KIAA0586
190.0246526	2.306265005	0.18294765	12.606147	1.95E-36	1.69E-34	DACT1
906.2543035	0.752743939	0.06943969	10.840255	2.22E-27	1.17E-25	DAAM1
204.7412317	0.66108043	0.14399704	4.5909306	4.41E-06	2.72E-05	L3HYPDH
2256.628524	-0.15842777	0.0523724	-3.025024	0.0024861	0.008721781	PCNX4
605.8551072	0.472041232	0.07687989	6.1399832	8.25E-10	8.77E-09	DHRS7
1781.511539	-0.3469983	0.04956484	-7.0008968	2.54E-12	3.52E-11	PPM1A
225.625117	0.35519815	0.1340705	2.6493385	0.0080649	0.024665754	SIX1
581.8786244	-0.28912491	0.07955917	-3.6340865	0.000279	0.00121707	TRMT5
75.55781414	0.84144478	0.20781543	4.0490004	5.14E-05	0.000259272	PRKCH
4.852414605	2.372817356	0.93021439	2.5508285	0.0107467	0.03181029	LINC01303
9394.658969	0.575108964	0.0397989	14.450375	2.49E-47	3.31E-45	HIF1A
234.4725679	-1.22852452	0.12371327	-9.9304184	3.07E-23	1.18E-21	HIF1A-AS3
794.1815034	-0.16455671	0.06967244	-2.3618621	0.0181834	0.049906786	SNAPC1
2704.564729	-0.32917336	0.04877428	-6.7489125	1.49E-11	1.90E-10	PPP2R5E
1015.086251	0.196708766	0.06710571	2.9313266	0.0033752	0.011432475	SGPP1
3303.51758	-0.8483881	0.21756299	-3.8995056	9.64E-05	0.000461893	SYNE2
3662.574889	-0.29904647	0.0398174	-7.5104463	5.89E-14	9.85E-13	MTHFD1
1339.123123	0.375145496	0.05693889	6.5885638	4.44E-11	5.44E-10	ZBTB1
584.0044633	0.570790727	0.07940556	7.1882969	6.56E-13	9.73E-12	HSPA2
593.0057684	0.394441394	0.07864923	5.015197	5.30E-07	3.79E-06	PLEKHG3
18.68668349	1.506261435	0.43543527	3.4592086	0.0005418	0.002229892	SPTB
418.3988063	-0.54775644	0.09043728	-6.0567551	1.39E-09	1.44E-08	GPHN
2921.305181	-0.13459642	0.04583552	-2.9365091	0.0033193	0.011273919	PALS1
2602.373441	0.279138216	0.04894745	5.7028143	1.18E-08	1.08E-07	ATP6V1D
33.68073151	1.221196214	0.31691276	3.8534144	0.0001165	0.00054791	PLEK2
226.8507811	0.491825175	0.12914342	3.8083642	0.0001399	0.000649405	PIGH
92.85556069	-0.44198957	0.18609117	-2.3751238	0.0175431	0.048355347	ARG2
1332.430958	-0.22222308	0.0539858	-4.1163246	3.85E-05	0.000198945	VTI1B
159.7853637	-0.45696291	0.13693117	-3.3371725	0.0008464	0.003326124	RAD51B
23600.3192	0.241737197	0.03395033	7.1203196	1.08E-12	1.56E-11	ACTN1
377.7099798	-0.42405012	0.09197284	-4.6106016	4.02E-06	2.49E-05	EXD2
2735.696677	-0.18775665	0.05836952	-3.2166899	0.0012968	0.004867499	ERH
596.4873069	0.825589851	0.08490478	9.7237146	2.39E-22	8.60E-21	SUSD6
120.1798923	1.543298645	0.20258866	7.6178927	2.58E-14	4.47E-13	SMOC1
1458.879442	-0.31092143	0.05537444	-5.6148911	1.97E-08	1.74E-07	SYNJ2BP

83.02825076	1.177317087	0.22673514	5.1924774	2.08E-07	1.58E-06	TTC9
14.94248563	1.954294837	0.51299518	3.8095774	0.0001392	0.000646745	ENSG00000269927
1101.798446	0.202978579	0.06348457	3.1972902	0.0013873	0.005172455	SIPA1L1
837.729191	0.276118197	0.06939948	3.9786785	6.93E-05	0.000341489	ZFYVE1
321.2394943	0.345700346	0.10488185	3.2960931	0.0009804	0.003797121	RIOX1
463.4751497	-0.6387542	0.09298146	-6.8696941	6.43E-12	8.54E-11	ALDH6A1
466.7287892	-0.33014241	0.08552816	-3.8600434	0.0001134	0.000534703	LIN52
461.920827	0.276591382	0.08665398	3.1919063	0.0014134	0.005258373	ABCD4
981.941873	0.61608855	0.06420888	9.595068	8.39E-22	2.85E-20	NPC2
345.815805	-0.34993547	0.10676886	-3.2775051	0.0010473	0.004022354	ISCA2
324.9865562	0.512412944	0.10344935	4.9532737	7.30E-07	5.09E-06	LTBP2
1638.46709	-0.14409993	0.05705298	-2.5257212	0.0115461	0.033842371	FCF1
2852.464679	-0.29956988	0.04996134	-5.9960336	2.02E-09	2.05E-08	YLPM1
165.5262592	0.444195075	0.14066023	3.1579295	0.0015889	0.005857889	RPS6KL1
118.9604853	1.893526265	0.20115705	9.4131736	4.81E-21	1.55E-19	PGF
341.5824744	0.302561879	0.11328352	2.6708376	0.0075662	0.023341606	MLH3
1825.629865	0.177434401	0.05306211	3.3439	0.0008261	0.003256073	NEK9
185.4694166	-2.45652326	0.55851578	-4.3983059	1.09E-05	6.27E-05	FOS
1304.377401	-0.42559074	0.05582629	-7.6234829	2.47E-14	4.29E-13	TTLL5
1652.432828	-0.49713823	0.05509078	-9.023983	1.81E-19	5.11E-18	GPATCH2L
349.1935927	-0.37893828	0.09606913	-3.9444333	8.00E-05	0.000388981	CIPC
235.7469523	0.330433944	0.11625135	2.8424095	0.0044774	0.014696466	GSTZ1
1449.484566	-0.52698781	0.0597897	-8.8140226	1.21E-18	3.15E-17	TMED8
5000.514094	0.136952863	0.0454261	3.0148498	0.0025711	0.008983491	SPTLC2
2675.454998	-0.2082821	0.04625129	-4.5032711	6.69E-06	3.96E-05	SNW1
237.1332779	0.380601492	0.13296805	2.8623529	0.0042051	0.013889156	NRXN3
2713.845971	-0.42108298	0.04775896	-8.8168365	1.18E-18	3.09E-17	GTF2A1
55.71058587	1.186223691	0.28630506	4.143216	3.42E-05	0.00017863	STON2
4523.865678	-0.27290129	0.04240445	-6.4356751	1.23E-10	1.44E-09	SEL1L
858.9154208	-0.40356056	0.06657468	-6.0617728	1.35E-09	1.40E-08	PTPN21
3155.986866	-0.2712404	0.04307483	-6.2969584	3.04E-10	3.41E-09	ZC3H14
782.4493086	-0.37502932	0.07520773	-4.9865794	6.15E-07	4.34E-06	TDP1
503.5528073	-0.29986554	0.08032205	-3.7332905	0.000189	0.00085772	NRDE2
8044.077481	0.188701619	0.04404264	4.284521	1.83E-05	0.0001004	CALM1
433.9643584	-0.60811633	0.08793502	-6.9155192	4.66E-12	6.33E-11	TTC7B
380.1606622	-0.28956856	0.09340624	-3.1000989	0.0019346	0.006982567	RPS6KA5
76.98809228	0.905960984	0.22559546	4.0158654	5.92E-05	0.000295376	FBLN5
1548.894151	-0.3953846	0.05458589	-7.2433487	4.38E-13	6.63E-12	TRIP11
565.1671391	-0.36202246	0.07767356	-4.6608198	3.15E-06	1.99E-05	ATXN3
395.5457602	0.258997927	0.09095199	2.8476334	0.0044046	0.014473802	NDUFB1
3051.346937	-0.40376918	0.04860332	-8.3074401	9.78E-17	2.14E-15	CPSF2
1964.026334	0.775852932	0.05607218	13.836683	1.53E-43	1.75E-41	LGMN
575.1664053	0.23746071	0.08000375	2.9681196	0.0029963	0.010299519	MOAP1
1498.541356	-0.21131825	0.05312307	-3.9779	6.95E-05	0.000342512	UBR7
1388.881606	-0.26196041	0.05558788	-4.7125451	2.45E-06	1.57E-05	BTBD7
5228.858902	0.173609037	0.03566286	4.8680632	1.13E-06	7.63E-06	DDX24
207.6346734	0.330187727	0.13021156	2.5357788	0.0112198	0.033002634	IFI27L1
97.90324571	4.255704029	0.53889325	7.8971188	2.85E-15	5.41E-14	IFI27

92.17591606	0.44921141	0.18451901	2.4344993	0.0149124	0.042139548	IFI27L2
191.0116238	0.766271472	0.14123317	5.4255772	5.78E-08	4.78E-07	PPP4R4
67.27031105	0.89906037	0.22803059	3.9427183	8.06E-05	0.000391449	SERPINA1
1681.07193	-0.23247903	0.06357307	-3.6568789	0.0002553	0.001125127	DICER1
577.150186	-0.38804289	0.07929452	-4.8936914	9.90E-07	6.75E-06	CLMN
228.9008221	-0.43396385	0.12682175	-3.421841	0.000622	0.002523647	SYNE3
476.2324837	-0.4026965	0.09096518	-4.4269302	9.56E-06	5.54E-05	C14orf132
1405.784228	-0.32165623	0.06790839	-4.7366201	2.17E-06	1.40E-05	ATG2B
684.312281	0.269887467	0.07258148	3.7184069	0.0002005	0.000906029	GSKIP
9961.230542	-0.2429647	0.03762172	-6.4580962	1.06E-10	1.25E-09	PAPOLA
477.6670088	-0.21046533	0.08438625	-2.4940713	0.0126287	0.03655958	EVL
3854.327581	-0.23213857	0.04108231	-5.650573	1.60E-08	1.43E-07	YY1
583.811932	0.554152285	0.09395243	5.8982219	3.67E-09	3.57E-08	SLC25A29
2198.640302	-0.2595488	0.04756616	-5.4565853	4.85E-08	4.07E-07	WARS1
10586.4901	0.903902646	0.18227753	4.9589362	7.09E-07	4.95E-06	MEG3
403.3184564	0.501619835	0.17997368	2.7871844	0.0053168	0.017112629	MEG8
134.499564	1.23852691	0.16970646	7.2980541	2.92E-13	4.51E-12	MEG9
3038.062118	-0.23358729	0.04633119	-5.0416858	4.61E-07	3.33E-06	PPP2R5C
12965.78262	-0.37963793	0.10157001	-3.7376971	0.0001857	0.00084455	DYNC1H1
69506.86777	-0.3913505	0.07821884	-5.0032768	5.64E-07	4.01E-06	HSP90AA1
558.9694512	0.22019734	0.08734511	2.5210037	0.0117021	0.034230254	TECPR2
1017.514956	0.240886114	0.06478045	3.7185002	0.0002004	0.000905931	TRAF3
3888.220427	-0.09474407	0.03964885	-2.3895795	0.0168677	0.046790369	CDC42BPB
381.1121905	0.951265317	0.09934081	9.5757753	1.01E-21	3.41E-20	TNFAIP2
6929.183387	-0.18722087	0.04373402	-4.2808974	1.86E-05	0.000101824	EIF5
568.2175966	0.994134568	0.08847033	11.236927	2.69E-29	1.60E-27	CKB
238.9188261	0.565537287	0.12116494	4.6674995	3.05E-06	1.93E-05	TRMT61A
638.2315625	0.414962309	0.07611124	5.4520503	4.98E-08	4.17E-07	KLC1
202.4279261	0.599266497	0.13023916	4.6012774	4.20E-06	2.59E-05	ENSG00000269940
195.2593191	0.606703778	0.13885188	4.3694314	1.25E-05	7.08E-05	ENSG00000269958
646.0686122	0.439614251	0.07661034	5.7383149	9.56E-09	8.84E-08	PPP1R13B
54.56375624	0.998926243	0.27843404	3.5876585	0.0003337	0.001433036	ADSS1
522.0411883	0.212458943	0.08114864	2.6181455	0.0088409	0.026724043	SIVA1
3238.132173	0.138783245	0.04361296	3.182156	0.0014618	0.005423774	AKT1
853.7456365	0.455022466	0.06942225	6.5544185	5.59E-11	6.77E-10	CEP170B
763.6197052	1.638245841	0.08555475	19.148508	9.96E-82	3.77E-79	AHNAK2
12.35457697	1.514262275	0.56878479	2.6622763	0.0077614	0.023854891	LINC02298
179.7747862	0.858401432	0.14433583	5.9472511	2.73E-09	2.71E-08	JAG2
549.3745577	0.382045587	0.08423578	4.5354315	5.75E-06	3.45E-05	BRF1
168.0326135	-0.50311887	0.13129133	-3.83208	0.0001271	0.000594152	ENSG00000251602
992.2430451	-0.34116216	0.06591327	-5.175925	2.27E-07	1.72E-06	MTA1
374.8950745	0.579794485	0.0972864	5.9596665	2.53E-09	2.53E-08	CRIP2
18.48263167	2.001635709	0.48910852	4.0924164	4.27E-05	0.000218862	NBEAP1
2114.374507	-0.27021721	0.05027394	-5.3748963	7.66E-08	6.24E-07	CYFIP1
1810.14591	-0.43063647	0.0537605	-8.010277	1.14E-15	2.29E-14	SNHG14
4242.95369	-0.38771115	0.04212745	-9.2032907	3.47E-20	1.04E-18	UBE3A
1924.625146	-0.47067206	0.10266457	-4.5845616	4.55E-06	2.79E-05	HERC2
6165.316817	-0.22277457	0.04000524	-5.5686352	2.57E-08	2.24E-07	TJP1

290.9842109	-0.45737635	0.10312222	-4.4352842	9.20E-06	5.35E-05	HMGN2P5
844.4676405	-0.1781452	0.06830264	-2.6081743	0.0091027	0.027429427	FAN1
1184.609328	0.491963	0.06646177	7.402195	1.34E-13	2.15E-12	MTMR10
2904.638517	-0.40550549	0.04188146	-9.6822202	3.59E-22	1.27E-20	ARHGAP11A
8139.68177	0.642471142	0.04213208	15.248978	1.67E-52	2.67E-50	GREM1
16.48179726	1.990856229	0.55175693	3.6082125	0.0003083	0.001332732	ENSG00000259408
1274.589027	0.194474986	0.05803232	3.3511497	0.0008048	0.003178495	EMC7
908.564739	-0.32893864	0.07026422	-4.681453	2.85E-06	1.81E-05	SLC12A6
53.67871967	0.746334467	0.25077068	2.9761632	0.0029188	0.010061018	ENSG00000279092
577.0542205	0.418707066	0.10682938	3.9194001	8.88E-05	0.00042842	GOLGA8B
223.1547044	1.944799874	0.15264245	12.740885	3.50E-37	3.16E-35	ACTC1
2249.403838	-0.3201514	0.05120479	-6.2523719	4.04E-10	4.48E-09	AQR
2392.033045	-0.41584024	0.04747048	-8.759976	1.95E-18	4.97E-17	ZNF770
98.62928172	-0.49644684	0.17560963	-2.826991	0.0046988	0.015342005	DPH6
471.3859106	-0.5244568	0.08856785	-5.9215256	3.19E-09	3.13E-08	MEIS2
1619.09956	-0.15981098	0.05564881	-2.8717774	0.0040817	0.01352531	SPRED1
1901.774788	-0.28402779	0.05197064	-5.4651582	4.62E-08	3.89E-07	FAM98B
163.756038	0.45246991	0.15497711	2.9195919	0.0035049	0.011818928	RASGRP1
32863.84508	0.278164996	0.09950553	2.7954726	0.0051824	0.016720176	THBS1
27.48584561	0.827637346	0.33413641	2.4769445	0.0132512	0.038114327	ENSG00000261136
2763.585245	-0.28198595	0.05099145	-5.5300638	3.20E-08	2.76E-07	GPR176
41.58568996	1.221489637	0.32755171	3.7291505	0.0001921	0.000870072	SRP14-DT
134.690035	0.880618095	0.17294419	5.0919208	3.54E-07	2.61E-06	BMF
2959.521245	-0.31746433	0.05137716	-6.1790951	6.45E-10	6.95E-09	BUB1B
6.692912388	2.611038688	0.86062088	3.0339012	0.0024141	0.008491466	PAK6
1324.979925	0.274692733	0.06194715	4.4343079	9.24E-06	5.37E-05	INAFM2
1329.662087	0.493325929	0.0718948	6.8617746	6.80E-12	9.00E-11	CCDC9B
111.1534732	0.423861434	0.17570592	2.4123343	0.0158507	0.04436574	DISP2
1948.802766	-0.14408405	0.05097462	-2.8265842	0.0047047	0.015358623	KNSTRN
657.4821969	-0.23869903	0.0767947	-3.1082749	0.0018818	0.006816291	BAHD1
315.3320197	0.266965655	0.10917569	2.4452847	0.0144738	0.041100439	CCDC32
3990.855126	-0.32785167	0.0532667	-6.1549084	7.51E-10	8.03E-09	KNL1
8.353286349	1.769611617	0.73232674	2.4164236	0.0156738	0.043948345	GCHFR
118.6960908	0.515126066	0.15947651	3.2301062	0.0012374	0.004670963	SPINT1
25.48544994	1.309502661	0.37455189	3.4961849	0.000472	0.001970567	RHOV
543.5617334	0.408711622	0.08538971	4.7864269	1.70E-06	1.12E-05	VPS18
22.9295104	1.574411915	0.46342716	3.3973234	0.0006805	0.002733572	DLL4
820.8842967	-0.18880871	0.06658492	-2.8356078	0.0045739	0.014956637	INO80
2430.972068	-0.28773528	0.05133122	-5.6054639	2.08E-08	1.84E-07	CHP1
2366.276621	-0.3276812	0.05276577	-6.2101096	5.29E-10	5.79E-09	OIP5-AS1
321.9223738	-0.2715862	0.10124349	-2.6825053	0.0073073	0.022651327	OIP5
506.7052369	-0.36348003	0.08363987	-4.3457746	1.39E-05	7.79E-05	RPAP1
259.9655073	-0.33510221	0.11284596	-2.9695543	0.0029823	0.010257628	TYRO3
1054.412918	-0.37776427	0.07652104	-4.9367372	7.94E-07	5.51E-06	MGA
901.462679	0.674292502	0.06925331	9.7366108	2.10E-22	7.62E-21	MAPKBP1
1629.153479	0.180157392	0.0603078	2.9872982	0.0028146	0.009744184	EHD4
1303.096597	-0.15339695	0.05906951	-2.5968887	0.0094072	0.028268861	VPS39
1177.484943	0.247909968	0.05877606	4.2178734	2.47E-05	0.000131716	TMEM87A

172.9155707	-0.42898599	0.13225954	-3.2435165	0.0011806	0.004475981	GANC
4324.12931	-0.5986383	0.04392374	-13.629038	2.69E-42	2.96E-40	ZNF106
1286.352912	-0.22734445	0.06051806	-3.756638	0.0001722	0.000788093	HAUS2
480.2976795	-0.40236268	0.09536264	-4.2192905	2.45E-05	0.000131012	STARD9
1244.711645	-0.52558813	0.07072727	-7.4311953	1.08E-13	1.74E-12	TTBK2
135.7992696	0.473366079	0.1562573	3.0294013	0.0024504	0.008605064	TMEM62
4.283267938	3.02631834	1.2384427	2.4436482	0.0145396	0.041261094	TGM5
1145.964844	-0.5484105	0.05853137	-9.3695145	7.29E-21	2.33E-19	TUBGCP4
2583.535369	-0.434211	0.04798749	-9.0484201	1.45E-19	4.11E-18	TP53BP1
824.8399987	0.328542681	0.07345454	4.472735	7.72E-06	4.55E-05	MAP1A
12358.50397	0.155544247	0.03845349	4.0449969	5.23E-05	0.000263132	PDIA3
1653.253602	-0.59587287	0.06124346	-9.7295763	2.26E-22	8.14E-21	WDR76
2450.0597	-0.28254212	0.05447567	-5.1865746	2.14E-07	1.63E-06	GOLM2
15.70143595	1.4421321	0.48274432	2.9873621	0.002814	0.009744086	GAPDHP43
2121.644232	-0.26641621	0.05462244	-4.8774128	1.07E-06	7.30E-06	CTDSPL2
219.0828749	-0.33572232	0.11908793	-2.8191129	0.0048157	0.015673645	EIF3J-DT
880.5613096	-0.61499654	0.07111038	-8.6484775	5.22E-18	1.28E-16	SPG11
9605.847603	0.944152022	0.04495443	21.002427	6.23E-98	3.87E-95	B2M
336.2984309	-0.33230272	0.10002718	-3.3221243	0.0008933	0.003491087	SORD
13.51747526	2.386012423	0.58700182	4.0647445	4.81E-05	0.000244289	C15orf48
2054.609986	-0.29278407	0.05130723	-5.7064876	1.15E-08	1.06E-07	BLOC1S6
132.5042715	0.562158956	0.15422099	3.6451521	0.0002672	0.001174127	SQOR
6.450206491	-1.93772424	0.78544894	-2.4670276	0.013624	0.039037912	SEMA6D
1389.282459	-0.48513256	0.05952917	-8.1494929	3.65E-16	7.60E-15	DUT
8381.395588	1.644298908	0.09815848	16.751471	5.52E-63	1.23E-60	FBN1
3.857965332	3.033553532	1.27006061	2.3885108	0.0169168	0.046904226	ENSG00000274654
11.03807156	1.414224727	0.58323569	2.4247911	0.0153172	0.043115258	FBN1-DT
1537.733263	-0.54679339	0.05860677	-9.3298681	1.06E-20	3.32E-19	CEP152
39.00158314	1.897185918	0.35627818	5.325013	1.01E-07	8.09E-07	SHC4
3160.901876	-0.19735265	0.04424853	-4.4600946	8.19E-06	4.80E-05	COPS2
249.5036564	-0.50966993	0.10952028	-4.6536582	3.26E-06	2.05E-05	GALK2
251.8815036	-0.44028182	0.11566161	-3.8066374	0.0001409	0.000653431	DTWD1
2357.486611	-0.22897026	0.04610694	-4.9660692	6.83E-07	4.79E-06	USP8
23.58424007	1.763237417	0.44936755	3.9238201	8.72E-05	0.000421128	TNFAIP8L3
9.328423076	4.571418595	1.27881889	3.5747193	0.0003506	0.001501724	SCG3
501.0126422	0.324614508	0.09063828	3.5814282	0.0003417	0.001465843	TMOD2
1736.5789	-0.22099653	0.0508874	-4.3428539	1.41E-05	7.88E-05	MAPK6
2335.274817	-0.16518013	0.05849516	-2.8238253	0.0047454	0.015479374	MYO5A
597.3181852	0.220187843	0.07532599	2.9231325	0.0034653	0.011696698	FAM214A
1939.19771	-0.39477541	0.05009685	-7.8802448	3.27E-15	6.14E-14	RSL24D1
582.4006712	-0.38296552	0.0843993	-4.5375435	5.69E-06	3.42E-05	RAB27A
113.2904396	-0.39593413	0.16589618	-2.3866381	0.0170032	0.047081414	PIGB
387.8109271	0.256293032	0.09545253	2.6850314	0.0072523	0.022504902	CCPG1
429.4053366	-0.53643068	0.08646791	-6.2038119	5.51E-10	6.01E-09	PYGO1
2222.279704	-0.23966576	0.05043328	-4.7521353	2.01E-06	1.31E-05	NEDD4
126.8687275	-0.73153576	0.15541978	-4.7068381	2.52E-06	1.61E-05	TEX9
201.4256286	-0.58285512	0.13355076	-4.3642965	1.28E-05	7.22E-05	MNS1
1851.105933	-0.61316395	0.04842134	-12.663093	9.47E-37	8.32E-35	TCF12

271.4412331	-0.57767536	0.11413672	-5.0612577	4.16E-07	3.03E-06	CGNL1
665.4447055	-0.42181916	0.076418	-5.5198925	3.39E-08	2.90E-07	POLR2M
945.7876984	-0.21108774	0.07289725	-2.8956885	0.0037833	0.012654587	MINDY2
2979.406742	-0.25694964	0.05235512	-4.9078224	9.21E-07	6.32E-06	CCNB2
1220.818522	0.153526045	0.06137211	2.5015603	0.0123647	0.035914948	GTF2A2
2969.866515	-0.11837342	0.04974035	-2.379827	0.0173208	0.047856392	BNIP2
1170.614804	-0.56905259	0.05761063	-9.8775628	5.21E-23	1.99E-21	ICE2
633.0089275	0.524010916	0.08277221	6.3307591	2.44E-10	2.77E-09	TLN2
20899.07284	0.698555577	0.04177753	16.720845	9.24E-63	2.03E-60	TPM1
1685.326166	0.562025866	0.0520557	10.796624	3.57E-27	1.88E-25	RPS27L
1530.807883	-0.3755613	0.05960742	-6.3005795	2.97E-10	3.34E-09	RAB8B
84.43284854	0.63032927	0.21366055	2.9501434	0.0031763	0.01083044	CA12
496.1493473	-0.5294728	0.08552771	-6.1906578	5.99E-10	6.50E-09	USP3
1273.670731	-0.4824799	0.1289405	-3.7418801	0.0001826	0.00083279	HERC1
1956.277306	-0.14435769	0.04886125	-2.9544411	0.0031324	0.010701714	SNX1
5762.758291	0.169665022	0.03832788	4.4266733	9.57E-06	5.55E-05	PPIB
454.4044461	-0.26952898	0.09432572	-2.8574284	0.0042709	0.014066434	PCLAF
1800.08224	-0.42048799	0.05425496	-7.7502225	9.17E-15	1.66E-13	ZNF609
1494.616088	-0.19330864	0.0553926	-3.4897915	0.0004834	0.002015412	OAZ2
324.197523	0.722100998	0.10905921	6.6211829	3.56E-11	4.40E-10	PLEKHO2
19.38694974	1.177802739	0.43345229	2.7172604	0.0065825	0.020707681	ENSG00000274383
919.383045	-0.33753121	0.06738848	-5.0087377	5.48E-07	3.91E-06	SPG21
624.6447019	-0.26602417	0.07260949	-3.6637657	0.0002485	0.001098909	PDCD7
1012.036081	-0.19755908	0.06361319	-3.1056306	0.0018987	0.006866094	CLPX
32.96586508	1.41811951	0.34945374	4.0581037	4.95E-05	0.000250532	IGDCC4
1695.237634	-0.27647158	0.05523963	-5.0049503	5.59E-07	3.98E-06	DPP8
850.6387086	-0.26996246	0.06396888	-4.2202157	2.44E-05	0.000130515	INTS14
826.5531017	-0.42045054	0.07084072	-5.9351536	2.94E-09	2.90E-08	DENND4A
535.1190062	-0.36263322	0.08114944	-4.4687088	7.87E-06	4.63E-05	DIS3L
675.9726318	-0.20307691	0.06993658	-2.9037296	0.0036875	0.012369793	TIPIN
1994.974256	-0.19353307	0.04998108	-3.8721269	0.0001079	0.000511366	MAP2K1
19631.12832	-0.39124179	0.03711713	-10.540734	5.61E-26	2.67E-24	RPL4
196.8100359	0.595532289	0.14395925	4.1368114	3.52E-05	0.000183303	SMAD6
1948.166378	0.131572778	0.04830344	2.7238798	0.006452	0.02034087	SMAD3
282.9088199	-0.77761665	0.10997443	-7.0708859	1.54E-12	2.18E-11	MAP2K5
1765.529527	-0.36760782	0.04971956	-7.3936253	1.43E-13	2.29E-12	PIAS1
1026.323198	1.170733411	0.13618906	8.5963839	8.23E-18	2.00E-16	ITGA11
2756.212564	-0.46980972	0.04530952	-10.368897	3.43E-25	1.56E-23	ANP32A
91.72061333	-0.61329164	0.1903996	-3.2210764	0.0012771	0.004802939	PAQR5
199.7460808	1.233033843	0.14492129	8.5083002	1.77E-17	4.14E-16	KIF23-AS1
9.045710086	2.735856511	0.88366119	3.0960469	0.0019612	0.00706257	LINC02204
1851.478758	-0.28459114	0.05332065	-5.3373529	9.43E-08	7.60E-07	UACA
135.2574851	-0.87297574	0.15169506	-5.7548068	8.67E-09	8.07E-08	LRRC49
224.2748807	-0.34481325	0.13124383	-2.6272721	0.0086072	0.026117883	THSD4
1384.642857	-0.6021769	0.07348625	-8.1944156	2.52E-16	5.30E-15	MYO9A
26.64572019	1.188729975	0.41849329	2.8404995	0.0045043	0.01475969	GRAMD2A
37982.11431	-0.21194735	0.03013585	-7.0330644	2.02E-12	2.82E-11	PKM
893.4837275	0.256613188	0.07368935	3.4823645	0.000497	0.002064727	HEXA

3.300230722	3.624712927	1.43102098	2.5329558	0.0113105	0.033252739	HEXA-AS1
805.5770092	0.380440186	0.07098499	5.3594457	8.35E-08	6.76E-07	CD276
388.4297609	-0.53856034	0.09116344	-5.9076349	3.47E-09	3.38E-08	LOXL1-AS1
391.0145417	-0.28181603	0.09608542	-2.9329738	0.0033573	0.011378645	LOXL1
66.19369655	0.578164439	0.22925936	2.5218793	0.011673	0.034162409	STOML1
701.9033982	0.495697868	0.07904518	6.27107	3.59E-10	4.00E-09	PML
4.153382023	2.592889141	1.08302635	2.3941145	0.0166605	0.046319245	STRA6
420.6778114	0.34619025	0.09428589	3.6717079	0.0002409	0.001068842	SEMA7A
23.34755922	2.927739653	0.53453677	5.4771529	4.32E-08	3.65E-07	ENSG00000260103
47.35805812	2.328205534	0.31227845	7.4555433	8.95E-14	1.46E-12	CYP1A1
777.7258088	-0.17560531	0.07171096	-2.4487931	0.0143336	0.040782217	CSK
319.0858946	0.257212024	0.10352397	2.4845649	0.012971	0.037411491	ULK3
918.7299866	0.269109369	0.06702464	4.015081	5.94E-05	0.000296105	SCAMP2
687.3219263	-0.38201443	0.07661303	-4.9862856	6.16E-07	4.35E-06	MPI
165.5254446	0.44837222	0.14077461	3.185036	0.0014474	0.005374672	SCAMP5
223.9803325	0.501610654	0.13559198	3.6994124	0.0002161	0.000969547	C15orf39
2413.082182	-0.527591	0.04923003	-10.716853	8.48E-27	4.35E-25	SIN3A
892.9617838	-0.20944157	0.06502037	-3.2211684	0.0012767	0.004802435	PTPN9
253.5599483	-0.33911136	0.11147746	-3.0419725	0.0023503	0.008290527	SNUPN
946.3736178	-0.24231732	0.07490392	-3.235042	0.0012162	0.004597947	SNX33
984.8392964	0.794253535	0.07192782	11.042368	2.39E-28	1.37E-26	CSPG4
515.3784458	-0.29585893	0.0923314	-3.2043155	0.0013538	0.005059808	SCAPER
2611.323512	-0.31730468	0.04598551	-6.9001018	5.20E-12	7.03E-11	RCN2
3186.423158	0.527069183	0.04887801	10.78336	4.13E-27	2.16E-25	TSPAN3
33.4363598	1.143751547	0.32347135	3.5358666	0.0004064	0.001720124	ENSG00000269951
1176.449241	0.212645119	0.05984405	3.5533211	0.0003804	0.001616607	TBC1D2B
1661.181164	0.137996917	0.05599047	2.4646502	0.0137147	0.039246136	IDH3A
25.36097171	1.418445287	0.41593779	3.4102342	0.0006491	0.00261766	ACSBG1
2547.412894	-0.33794354	0.04773981	-7.0788619	1.45E-12	2.06E-11	IREB2
3776.17073	-0.16549953	0.04187153	-3.9525548	7.73E-05	0.000377277	PSMA4
342.5133719	0.62263413	0.10143625	6.1381816	8.35E-10	8.86E-09	CHRNA5
159.3458684	1.113264461	0.17235888	6.4589911	1.05E-10	1.24E-09	ADAMTS7
4652.444331	-0.25819315	0.04269283	-6.0476936	1.47E-09	1.52E-08	MORF4L1
275.2306084	-0.33910387	0.10485696	-3.2339662	0.0012208	0.004613296	FAH
33.52148895	1.146726535	0.33944324	3.3782571	0.0007295	0.002905509	CEMIP
454.4075151	0.58519551	0.09574358	6.112112	9.83E-10	1.04E-08	MEX3B
699.3378475	-0.41342094	0.07599448	-5.4401445	5.32E-08	4.43E-07	EFL1
1401.578638	-0.34470435	0.06267041	-5.5002724	3.79E-08	3.22E-07	ENSG00000237550
201.2012562	0.370301506	0.12284523	3.0143744	0.0025751	0.008995169	RPS17
90.01980684	2.262041652	0.26645134	8.4895112	2.08E-17	4.84E-16	AP3B2
43.63806266	0.925401558	0.27319532	3.3873259	0.0007058	0.002821478	ENSG00000252690
1570.256027	-0.36896497	0.05219934	-7.0683836	1.57E-12	2.21E-11	HDGFL3
2017.919495	-0.3563939	0.04762385	-7.4835179	7.24E-14	1.19E-12	BNC1
243.659785	0.731205477	0.13271234	5.5097022	3.59E-08	3.06E-07	ADAMTSL3
440.8608433	0.232027066	0.09382558	2.4729618	0.0133998	0.038478098	WDR73
104.0744326	0.450972784	0.18175498	2.4812128	0.0130936	0.037717085	KLHL25
4.037039497	3.186017713	1.24452027	2.5600368	0.0104661	0.031058978	LINC00052
828.7809071	0.612659263	0.06904445	8.8734038	7.09E-19	1.91E-17	AEN

31.85846142	1.80305304	0.33906284	5.3177547	1.05E-07	8.38E-07	ISG20
6.411600361	2.007586322	0.78066782	2.5716269	0.0101222	0.030146417	ACAN
324.2198099	1.1600846	0.11544034	10.049214	9.26E-24	3.71E-22	HAPLN3
5462.496285	1.237040213	0.03833045	32.273041	1.67E-228	1.45E-224	MFGE8
2493.494276	-0.12861281	0.04732113	-2.717873	0.0065703	0.020680112	ABHD2
3543.966631	-0.31507353	0.04629912	-6.805174	1.01E-11	1.31E-10	FANCI
46.12927027	-0.60042627	0.24917748	-2.409633	0.0159686	0.044659621	PEX11A
17281.27383	-0.20345767	0.03438494	-5.9170582	3.28E-09	3.21E-08	IQGAP1
1214.281641	-0.24150279	0.05857716	-4.122815	3.74E-05	0.000193823	BLM
445.2640079	-0.42338346	0.08459108	-5.0050606	5.58E-07	3.98E-06	FURIN
23.79249849	0.845769977	0.34774366	2.4321651	0.0150089	0.042384568	FES
1439.931621	-0.15389527	0.05450618	-2.8234464	0.004751	0.015489413	UNC45A
32.72609869	0.809366372	0.33902692	2.3873219	0.0169716	0.047026222	SLCO3A1
65.99019802	0.656338753	0.22465194	2.9215807	0.0034826	0.011752442	FAM174B
439.5291654	-0.40208303	0.08805892	-4.566068	4.97E-06	3.02E-05	CHASERR
2408.303676	-0.35495965	0.05723791	-6.2014782	5.59E-10	6.10E-09	CHD2
5153.717017	-0.22818531	0.04098033	-5.5681667	2.57E-08	2.25E-07	NR2F2
478.2922549	0.538709537	0.10737133	5.0172567	5.24E-07	3.75E-06	ARRDC4
1298.344971	-0.22702422	0.06565816	-3.4576697	0.0005449	0.002241607	IGF1R
1039.826896	0.225752369	0.07132987	3.1649064	0.0015513	0.005728945	SYNM
391.0007454	-0.44685563	0.09156873	-4.8800024	1.06E-06	7.21E-06	TTC23
11.61841016	1.723941878	0.55478135	3.1074258	0.0018872	0.006830211	HSP90B2P
565.2883166	-0.24315536	0.0791448	-3.0722849	0.0021243	0.007588544	ASB7
39.91192738	0.9455892	0.28859342	3.2765446	0.0010509	0.004032305	ALDH1A3
98.17385272	-0.62171495	0.17315098	-3.5905944	0.0003299	0.001418741	LRRK1
735.0071815	0.327031501	0.07799469	4.1929967	2.75E-05	0.000146134	SELENOS
240.4331809	0.479969375	0.11580793	4.1445296	3.41E-05	0.000177823	TARS3
898.1067564	0.338254414	0.0686893	4.9244118	8.46E-07	5.84E-06	SNRNP25
187.280141	0.348190813	0.13858031	2.5125561	0.011986	0.034954992	MPG
784.5444351	0.177360385	0.06975743	2.5425302	0.0110053	0.032470701	LUC7L
972.4423937	0.390716795	0.06858286	5.6970038	1.22E-08	1.11E-07	FAM234A
356.247416	0.299444014	0.09812687	3.0516007	0.0022762	0.008066829	PGAP6
64.6245902	0.541229632	0.21946378	2.4661455	0.0136576	0.039120164	DECR2
575.5102601	0.223665873	0.07976206	2.8041637	0.0050447	0.01632753	RAB11FIP3
219.4278117	-0.30914984	0.12011916	-2.573693	0.010062	0.029997829	RAB40C
609.3368253	0.249942535	0.08242379	3.0324077	0.0024261	0.008530147	RHOT2
24.95780927	2.097958614	0.43321466	4.8427692	1.28E-06	8.59E-06	RHBDL1
802.0244127	0.236124276	0.07284873	3.2412959	0.0011899	0.004508049	STUB1
131.1907119	1.139599994	0.16794687	6.7854794	1.16E-11	1.50E-10	MSLN
200.7301563	0.512010612	0.131221	3.9018953	9.54E-05	0.000457734	RPUSD1
227.7442801	-0.47985357	0.12057323	-3.9797686	6.90E-05	0.000340217	CACNA1H
61.9536882	1.272666433	0.27978589	4.5487155	5.40E-06	3.25E-05	BAIAP3
405.3904933	0.382428579	0.09427209	4.0566468	4.98E-05	0.000251864	GNPTG
935.0215811	0.426134284	0.06472315	6.5839547	4.58E-11	5.61E-10	CLCN7
373.989164	0.247440396	0.09213264	2.6856976	0.0072379	0.022464097	TELO2
207.8741943	-0.62596567	0.14974716	-4.1801505	2.91E-05	0.000153935	IFT140
531.1602555	0.385985978	0.09773268	3.9494055	7.83E-05	0.000381847	MAPK8IP3
61.55539775	0.659255219	0.23432442	2.8134294	0.0049016	0.015917661	NME3

144.1623251	0.914889984	0.17049176	5.366183	8.04E-08	6.53E-07	EME2
207.6949061	0.347852301	0.12655941	2.7485297	0.0059863	0.019031469	SPSB3
9350.892726	-0.30015709	0.03472166	-8.6446652	5.40E-18	1.32E-16	RPS2
629.9837142	0.176616574	0.07427036	2.3780224	0.0174058	0.048053069	ZNF598
694.8487252	0.249908399	0.07228405	3.4573103	0.0005456	0.002243537	SLC9A3R2
766.9678977	0.268376463	0.0765731	3.5048402	0.0004569	0.001911735	PKD1
1078.733142	-0.21790286	0.06365431	-3.4232223	0.0006188	0.002512615	TRAF7
443.4663607	0.516727584	0.0896467	5.7640447	8.21E-09	7.67E-08	PGP
264.024579	0.279857262	0.11242292	2.4893257	0.0127986	0.037002009	E4F1
185.8777029	0.789787507	0.16903078	4.672448	2.98E-06	1.89E-05	ABCA3
217.4308872	0.374735211	0.13059833	2.8693721	0.0041129	0.013613041	AMDHD2
1256.646536	0.159646299	0.05738309	2.782114	0.0054006	0.017350186	PDPK1
583.3128977	0.698177318	0.16058248	4.34778	1.38E-05	7.72E-05	ERVK13-1
1023.598211	0.518417365	0.06924007	7.487245	7.03E-14	1.16E-12	KCTD5
298.1259886	-0.36957289	0.11109572	-3.3266167	0.0008791	0.003444583	PAQR4
1369.456498	0.148254714	0.05322493	2.7854375	0.0053456	0.017195561	TNFRSF12A
91.46937525	0.558814855	0.20212229	2.7647364	0.0056969	0.018214519	MMP25-AS1
78.17648129	2.324073675	0.24499664	9.486145	2.40E-21	7.91E-20	IL32
292.4285213	-0.34951365	0.10503285	-3.3276604	0.0008758	0.003432479	TIGD7
454.361157	0.280352908	0.09020832	3.1078385	0.0018846	0.00682352	NAA60
1416.020208	-0.34088952	0.05548194	-6.1441531	8.04E-10	8.56E-09	TRAP1
2200.419162	-0.3542223	0.06035868	-5.8686224	4.39E-09	4.22E-08	CREBBP
66.34362358	-0.82928661	0.22453658	-3.6933252	0.0002213	0.000990763	LINC02861
857.3499786	-0.39102827	0.06796307	-5.7535402	8.74E-09	8.12E-08	ADCY9
73.27037248	-0.9735837	0.20009053	-4.8657161	1.14E-06	7.71E-06	TFAP4
34.6848079	0.788416775	0.32314273	2.4398406	0.0146937	0.041643445	ENSG00000280063
688.5442023	0.457088362	0.08392785	5.4462058	5.15E-08	4.30E-07	VASN
997.6236136	0.499154957	0.07048024	7.0821967	1.42E-12	2.02E-11	HMOX2
379.4219923	0.354741724	0.09523253	3.7250056	0.0001953	0.000884264	CDIP1
180.0550797	0.432171462	0.13276644	3.2551257	0.0011334	0.00431768	UBALD1
138.9503904	0.391528428	0.15876441	2.4660969	0.0136594	0.039120164	NUDT16L1
190.4161113	0.317577092	0.12965742	2.4493554	0.0143112	0.04073192	ZNF500
397.2226539	0.280129725	0.10098162	2.7740665	0.005536	0.017745928	ALG1
298.4533024	0.430487193	0.10522091	4.0912705	4.29E-05	0.000219882	METTL22
788.9357657	0.38254986	0.07146943	5.3526363	8.67E-08	7.00E-07	PMM2
3723.851636	-0.26996714	0.04688386	-5.7582099	8.50E-09	7.92E-08	USP7
214.4454086	0.547943353	0.12638507	4.3355071	1.45E-05	8.13E-05	ATF7IP2
324.824791	-0.33681803	0.11347083	-2.9683226	0.0029943	0.010294752	RMI2
632.6997122	0.272618673	0.08048629	3.3871443	0.0007062	0.002822697	SNN
5830.557565	-0.10387581	0.03823839	-2.7165318	0.006597	0.020737814	GSPT1
519.5028329	0.213759034	0.08114752	2.634203	0.0084335	0.025684528	CPPED1
874.3790624	-0.4877185	0.08089632	-6.0289328	1.65E-09	1.69E-08	MRTFB
749.7533825	0.388538312	0.07250517	5.3587669	8.38E-08	6.78E-07	NOMO1
1921.305408	0.276299737	0.05234904	5.2780291	1.31E-07	1.03E-06	RRN3
40.75885191	0.874303842	0.31691268	2.7588162	0.0058011	0.018496771	BMERB1
1159.555074	-0.30979983	0.06165663	-5.0245985	5.04E-07	3.63E-06	MARF1
1007.05554	-0.29835583	0.0655144	-4.5540495	5.26E-06	3.18E-05	NDE1
1600.990472	-0.38251973	0.05231146	-7.3123505	2.63E-13	4.08E-12	CEP20

127.0859053	0.404712126	0.16751196	2.4160192	0.0156912	0.043975914	NOMO3
9932.520918	0.322543367	0.04306023	7.4905168	6.86E-14	1.13E-12	ARL6IP1
4599.937623	-0.3621856	0.14923201	-2.4269966	0.0152244	0.04288875	SMG1
411.202329	-0.31019886	0.08651753	-3.5853873	0.0003366	0.001444496	COQ7
774.2457427	0.172728205	0.06701917	2.5772953	0.0099577	0.029732849	ITPRIPL2
1036.483353	-0.35432627	0.06185528	-5.7283112	1.01E-08	9.33E-08	CCP110
752.8377464	-0.18216525	0.07336072	-2.4831442	0.0130228	0.037544284	VPS35L
42.5379583	-0.83122248	0.27228399	-3.0527777	0.0022673	0.008041815	IQCK
386.8987857	0.394372655	0.10286747	3.8337938	0.0001262	0.000590343	DCUN1D3
38.82124758	2.941625871	0.46352834	6.3461619	2.21E-10	2.53E-09	DNAH3
1514.989464	-0.29603666	0.05946199	-4.9785865	6.41E-07	4.51E-06	METTL9
84.94987139	0.563630576	0.2047534	2.7527287	0.0059101	0.01880629	RRN3P1
533.2528095	-0.28011575	0.08016489	-3.4942446	0.0004754	0.001983993	EEF2K
1228.65084	0.221507863	0.0554003	3.9983152	6.38E-05	0.000315977	CDR2
83.9528452	0.754514957	0.19632001	3.8432912	0.0001214	0.000569481	RRN3P3
597.1629584	-0.28772949	0.07959157	-3.6150748	0.0003003	0.001301678	USP31
182.0095754	-0.43302162	0.13188302	-3.2833766	0.0010257	0.003945408	COG7
680.2189458	-0.23610512	0.07011495	-3.3674003	0.0007588	0.003013463	EARS2
1942.36034	-0.14092446	0.04998397	-2.8193933	0.0048115	0.01566289	UBFD1
2479.764028	-0.13935072	0.05068068	-2.7495827	0.0059671	0.018980836	TNRC6A
2071.693167	0.200663248	0.04633268	4.3309228	1.48E-05	8.29E-05	ARHGAP17
543.6623034	-0.23225496	0.07944889	-2.9233252	0.0034631	0.01169173	LCMT1
464.4981666	0.371139613	0.08622098	4.3045161	1.67E-05	9.25E-05	ZKSCAN2
344.7512459	-0.39143628	0.10338517	-3.7861939	0.000153	0.000705943	NSMCE1
358.2336061	0.820844213	0.10173981	8.0680732	7.14E-16	1.46E-14	IL4R
323.2741213	-0.23768605	0.09852172	-2.4125242	0.0158425	0.044349773	KATNIP
160.2280195	0.357157561	0.14544494	2.4556204	0.0140642	0.04015364	SGF29
3009.043736	-0.18370814	0.05115615	-3.591125	0.0003293	0.001416206	ATXN2L
2484.138785	-0.11814136	0.04384474	-2.6945389	0.0070486	0.02196297	TUFM
39.95785656	0.781151508	0.30784279	2.5375014	0.0111647	0.032901882	ATP2A1
89.16694992	0.622473026	0.19282372	3.2281973	0.0012457	0.004698166	QPRT
1403.352677	-0.26461246	0.05512594	-4.800144	1.59E-06	1.05E-05	KIF22
1550.717855	-0.29019477	0.05353755	-5.4203967	5.95E-08	4.91E-07	MAZ
41.58443487	-0.7031625	0.27091805	-2.5954804	0.0094459	0.028375163	PRRT2
762.6361616	0.857104803	0.07451449	11.502525	1.28E-30	8.28E-29	MVP
810.2345828	0.31673259	0.07561444	4.1887845	2.80E-05	0.000148561	SEZ6L2
1632.34949	0.30806369	0.05105704	6.033716	1.60E-09	1.64E-08	TAOK2
590.4112377	-0.49180702	0.0868074	-5.6654966	1.47E-08	1.32E-07	HIRIP3
912.3530535	-0.15743029	0.06615706	-2.3796444	0.0173294	0.0478649	ALDOA
20.7407972	-1.06542987	0.40077664	-2.6584131	0.007851	0.024091792	GDPD3
432.7898678	-0.47934434	0.0945181	-5.0714553	3.95E-07	2.87E-06	MAPK3
1557.479394	0.209671312	0.05363215	3.9094336	9.25E-05	0.000445034	CD2BP2
1180.481109	0.268903062	0.06058296	4.4385925	9.05E-06	5.28E-05	SEPHS2
311.7181444	0.426387399	0.10839507	3.933642	8.37E-05	0.000405291	PHKG2
2209.121743	0.112786049	0.04606192	2.4485746	0.0143423	0.04080028	RNF40
521.4491133	-0.28638231	0.08045652	-3.5594666	0.0003716	0.001583503	BCL7C
98.68160225	-0.57838392	0.17113372	-3.3797192	0.0007256	0.002891423	FBXL19-AS1
788.8300209	-0.18901479	0.07104955	-2.6603235	0.0078066	0.023976708	BCKDK

1045.450336	0.258172354	0.06791917	3.8011707	0.000144	0.000667485	TGFB1I1
4175.479451	-0.17551228	0.04338111	-4.0458229	5.21E-05	0.000262282	VPS35
1180.55321	0.27622925	0.06008956	4.5969588	4.29E-06	2.64E-05	ORC6
359.1282335	0.425420201	0.09854411	4.3170536	1.58E-05	8.77E-05	C16orf87
866.7406521	-0.18702481	0.07052953	-2.6517236	0.0080082	0.024509457	GPT2
2929.463083	0.155343849	0.05511159	2.8187149	0.0048216	0.015687217	NETO2
856.2578338	-0.24740677	0.06520288	-3.794415	0.000148	0.000684281	ITFG1
1141.803445	-0.4017948	0.05934341	-6.7706722	1.28E-11	1.65E-10	PHKB
1274.891761	-0.38161044	0.06190763	-6.1641902	7.08E-10	7.60E-09	LONP2
874.678122	0.245545316	0.06576382	3.7337446	0.0001887	0.000856801	TENT4B
1377.550863	-0.19064634	0.05631354	-3.3854443	0.0007106	0.00283763	BRD7
4.169662345	2.668833978	1.08666016	2.4559969	0.0140494	0.040128327	CYLD-AS1
1455.640855	-0.13440549	0.05515696	-2.436782	0.0148186	0.041933142	CYLD
91.14774988	-0.66897625	0.17818434	-3.7544054	0.0001738	0.000794523	HNRNPA1P48
729.9272565	-0.73155259	0.07766132	-9.4197802	4.52E-21	1.46E-19	CHD9
1699.28515	-0.54125465	0.06354596	-8.5175307	1.63E-17	3.83E-16	RBL2
211.8870388	-0.34562415	0.12338546	-2.801174	0.0050917	0.016467162	AKTIP
426.9686324	-0.37234146	0.09516995	-3.9123846	9.14E-05	0.000439994	RPGRIP1L
833.0263981	-0.47305463	0.07169688	-6.597981	4.17E-11	5.11E-10	FTO
326.2469376	-0.45730023	0.11959103	-3.8238673	0.0001314	0.000612823	CRNDE
190.6914872	1.762885767	0.17010637	10.363432	3.64E-25	1.64E-23	MMP2
1137.124596	-0.39924351	0.06776354	-5.8917153	3.82E-09	3.70E-08	NUDT21
2602.781938	-0.13743555	0.04852675	-2.8321606	0.0046235	0.015107477	MT2A
591.501738	-0.27308405	0.08500793	-3.212454	0.0013161	0.004932398	MT1E
1461.358138	-0.17476682	0.05400709	-3.2359976	0.0012122	0.004583579	NUP93
961.5084954	0.248702002	0.06407596	3.881362	0.0001039	0.000494485	HERPUD1
78.30939791	1.313546675	0.21319015	6.1613853	7.21E-10	7.73E-09	NLRC5
1145.948697	-0.21031923	0.0567739	-3.7045053	0.0002118	0.000951994	PSME3IP1
830.4412512	-0.302646	0.07115075	-4.2535884	2.10E-05	0.000113897	RSPRY1
617.0711624	0.199170188	0.07995063	2.4911648	0.0127325	0.036823255	CIAPIN1
426.8216756	-0.21537407	0.08885731	-2.4238195	0.0153582	0.043195818	COQ9
270.2073333	0.290950517	0.11518294	2.5259861	0.0115374	0.033833971	DOK4
202.4917214	1.974561504	0.16853495	11.716036	1.05E-31	7.25E-30	ADGRG1
661.9727245	-0.24194108	0.07342378	-3.2951323	0.0009838	0.003807584	KIFC3
5.336734489	3.366041429	1.0333401	3.2574381	0.0011242	0.004285471	CNGB1
1124.425151	0.426870777	0.06802181	6.2754988	3.49E-10	3.90E-09	USB1
379.0745769	0.388255942	0.0985694	3.9389095	8.19E-05	0.00039716	MMP15
2432.807164	-0.38232862	0.04863739	-7.8607964	3.82E-15	7.16E-14	CSNK2A2
6271.711504	-0.41734566	0.03843422	-10.858699	1.81E-27	9.67E-26	CNOT1
2796.374393	-0.2278808	0.04641808	-4.9093111	9.14E-07	6.27E-06	GOT2
90.4316839	2.813897978	0.28719793	9.7977654	1.15E-22	4.26E-21	CDH8
448.2269052	0.24326297	0.09104886	2.6717849	0.0075449	0.023288212	CMTM3
826.1516462	-0.46366697	0.07218712	-6.4231261	1.34E-10	1.56E-09	CMTM4
4506.354332	0.173001308	0.04478708	3.8627502	0.0001121	0.000529385	DYNC1LI2
1191.80281	-0.24050264	0.0576007	-4.1753425	2.98E-05	0.000156984	NAE1
464.5253276	-0.46718872	0.0846155	-5.5213136	3.36E-08	2.88E-07	PDP2
751.4579885	0.379652354	0.07316227	5.1891823	2.11E-07	1.61E-06	CES2
580.1657559	-0.28995185	0.08003685	-3.6227294	0.0002915	0.00126696	FHOD1

859.7370191	0.429960215	0.06953879	6.1830266	6.29E-10	6.80E-09	ATP6V0D1
1511.66306	-0.47929479	0.05768824	-8.3083628	9.70E-17	2.13E-15	CTCF
594.6692716	0.4000631	0.0872318	4.5862069	4.51E-06	2.77E-05	CENPT
33.06427896	1.438790358	0.3227595	4.4577785	8.28E-06	4.85E-05	PSMB10
115.5079056	0.520560304	0.18232522	2.8551195	0.0043021	0.014158381	LCAT
1003.688772	0.379210042	0.06375171	5.9482334	2.71E-09	2.69E-08	SLC12A4
29.02127728	1.316625211	0.41397856	3.1804188	0.0014706	0.005448261	SMPD3
324.846207	-0.52303686	0.10189604	-5.1330441	2.85E-07	2.13E-06	TANGO6
469.1203907	1.415523974	0.10211561	13.861975	1.08E-43	1.24E-41	HAS3
2152.538893	-0.17136917	0.04691218	-3.6529783	0.0002592	0.001141207	CYB5B
1546.685225	-0.40707616	0.11679244	-3.4854666	0.0004913	0.002044353	NFAT5
1210.244523	-0.33028975	0.07229281	-4.5687771	4.91E-06	2.99E-05	NQO1
578.5528404	-0.19790342	0.07639353	-2.5905785	0.0095815	0.028742779	WWP2
3532.057514	-0.28359347	0.04764041	-5.9527929	2.64E-09	2.63E-08	AARS1
701.5691029	-0.27834699	0.07693099	-3.6181386	0.0002967	0.001287135	COG4
4388.053026	-0.4348705	0.0396642	-10.963805	5.70E-28	3.19E-26	SF3B3
494.3226049	-0.4382171	0.08505713	-5.1520324	2.58E-07	1.94E-06	VAC14
3.865439148	2.646359333	1.09631937	2.4138581	0.0157846	0.044223357	VAC14-AS1
3289.239559	-0.18896067	0.04259059	-4.4366761	9.14E-06	5.32E-05	AP1G1
1192.584629	0.177674014	0.06447418	2.7557388	0.005856	0.018651168	ATXN1L
250.8068216	-0.51978834	0.11478847	-4.5282276	5.95E-06	3.55E-05	ENSG00000259768
2491.745469	-0.22540913	0.04846112	-4.6513397	3.30E-06	2.07E-05	RFWD3
62.10545823	0.57146274	0.2217426	2.5771446	0.009962	0.029740711	MLKL
437.9188725	-0.27698426	0.08706353	-3.1814039	0.0014656	0.005435558	WDR59
395.8585185	-0.22670305	0.09546987	-2.3746031	0.0175678	0.048408269	ZFP1
1053.363252	0.259301288	0.06799565	3.8134983	0.000137	0.000637758	BCAR1
594.6865856	-0.57417345	0.07663287	-7.4925217	6.76E-14	1.12E-12	CFDP1
449.3429074	-0.25191312	0.08584475	-2.9345198	0.0033406	0.011330947	TMEM170A
3155.094494	-0.22983436	0.0435102	-5.28231	1.28E-07	1.01E-06	KARS1
1105.247768	0.257826317	0.06468227	3.9860429	6.72E-05	0.000331819	TERF2IP
687.4101301	-0.29798304	0.0712232	-4.1837915	2.87E-05	0.000151581	MON1B
155.7679454	0.484158393	0.1490766	3.2477156	0.0011634	0.004420088	CDYL2
1211.146896	0.188846514	0.05853014	3.2264833	0.0012532	0.004721278	CMIP
115.7631105	0.725623507	0.17896537	4.0545469	5.02E-05	0.000253811	PLCG2
639.4996504	0.443279884	0.07571514	5.8545737	4.78E-09	4.59E-08	CDH13
1309.632067	0.243882413	0.06085034	4.0079053	6.13E-05	0.000304547	HSBP1
43.16564938	1.211185469	0.28842304	4.1993367	2.68E-05	0.000142345	OSGIN1
2874.293242	-0.14168688	0.04419645	-3.2058429	0.0013467	0.005035191	MBTPS1
892.4456787	-0.19911804	0.0679361	-2.9309608	0.0033792	0.011441096	HSDL1
7868.986743	0.36346883	0.04448706	8.1702145	3.08E-16	6.43E-15	COTL1
831.3412248	-0.42306267	0.0703981	-6.009575	1.86E-09	1.89E-08	KLHL36
2847.03718	-0.21794814	0.04588619	-4.7497548	2.04E-06	1.32E-05	USP10
821.7940068	1.565330134	0.15656848	9.9977345	1.56E-23	6.07E-22	CRISPLD2
403.6831686	0.364499535	0.09908373	3.6787022	0.0002344	0.001042133	KIAA0513
4.536853088	4.069264554	1.35952135	2.9931597	0.0027611	0.009574217	CIBAR2
760.8097074	-0.43079535	0.06949762	-6.1987066	5.69E-10	6.20E-09	GINS2
1247.756433	0.243629889	0.06012168	4.0522802	5.07E-05	0.000255959	FOXC2
1414.245876	0.764592558	0.06237106	12.258771	1.51E-34	1.20E-32	FOXL1

2039.410218	0.597836099	0.05050786	11.836496	2.53E-32	1.82E-30	MAP1LC3B
1026.883969	-0.22801195	0.06632409	-3.4378449	0.0005864	0.0023954	ZCCHC14
6020.916474	0.297690857	0.05605622	5.3105766	1.09E-07	8.70E-07	SLC7A5
42.13288647	1.379466281	0.36179044	3.8128876	0.0001374	0.000639166	ZNF469
914.8251696	0.509564202	0.07182797	7.0942313	1.30E-12	1.86E-11	CYBA
292.7089564	0.422726103	0.10820613	3.9066744	9.36E-05	0.000449398	GALNS
3264.671014	-0.13748578	0.04587089	-2.9972338	0.0027244	0.009462275	ANKRD11
1229.612507	0.256051874	0.06965699	3.6758961	0.000237	0.001052261	SPG7
5482.808028	-0.25483211	0.03911866	-6.5143357	7.30E-11	8.76E-10	RPL13
246.3688289	0.473570785	0.12216264	3.87656	0.0001059	0.000502965	SPATA33
43.36014152	1.5089546	0.31510022	4.7888085	1.68E-06	1.11E-05	ENSG00000275734
1134.624485	-0.24231322	0.06050277	-4.0049937	6.20E-05	0.00030797	FANCA
121.8320419	0.448313002	0.16939561	2.6465444	0.0081319	0.024857347	SPIRE2
41.02476016	1.270955734	0.30356504	4.1867658	2.83E-05	0.000149836	TUBB3
1448.985545	-0.2739473	0.05904966	-4.6392695	3.50E-06	2.19E-05	RFLNB
361.3891792	-0.23224423	0.09531358	-2.436633	0.0148247	0.041939449	TLCD3A
936.9588104	-0.16725316	0.06352812	-2.6327421	0.0084699	0.025786229	GEMIN4
2116.782844	0.187806299	0.04868094	3.8579021	0.0001144	0.000538969	NXN
1580.246912	0.238521853	0.05462401	4.3666119	1.26E-05	7.15E-05	ABR
18792.52493	-0.25043519	0.04111858	-6.0905599	1.13E-09	1.18E-08	YWHAE
4875.707207	-0.29570049	0.0425085	-6.9562667	3.49E-12	4.78E-11	MYO1C
96.96129923	-0.64797083	0.18029265	-3.5939947	0.0003256	0.001401731	PITPNA-AS1
1802.801069	0.320831787	0.05063287	6.3364325	2.35E-10	2.68E-09	PITPNA
303.7043751	0.605522463	0.11881538	5.0963305	3.46E-07	2.55E-06	SLC43A2
23.4383401	1.175059759	0.39955975	2.9408862	0.0032727	0.011122353	SCARF1
12898.6154	-0.42582	0.03851402	-11.056234	2.05E-28	1.19E-26	PRPF8
700.0702746	0.628742855	0.08831539	7.1192898	1.08E-12	1.57E-11	MIR22HG
6454.778163	-0.17888489	0.03690352	-4.8473663	1.25E-06	8.41E-06	RPA1
77.13953953	0.705128614	0.20347124	3.4654952	0.0005293	0.002183135	HIC1
201.1134748	0.513101955	0.14379211	3.5683595	0.0003592	0.001534487	SRR
3296.118949	-0.27018419	0.04270209	-6.3271884	2.50E-10	2.83E-09	TSR1
329.3227166	-0.31515843	0.09811473	-3.2121419	0.0013175	0.004936694	MNT
1030.738314	-0.28842722	0.06017809	-4.7928942	1.64E-06	1.09E-05	METTL16
6441.301043	-0.21467152	0.0362493	-5.9220879	3.18E-09	3.12E-08	PAFAH1B1
1043.787274	0.181971273	0.06029847	3.0178425	0.0025458	0.008907782	CLUH
560.7709838	-0.7727252	0.07746081	-9.9756917	1.95E-23	7.54E-22	RAP1GAP2
11.61837615	1.598814121	0.60170573	2.6571363	0.0078808	0.024170437	ASPA
95.18482348	0.609298262	0.20068207	3.036137	0.0023963	0.008435573	TRPV1
2381.032141	-0.24871858	0.04785625	-5.1972022	2.02E-07	1.55E-06	NCBP3
105.6740073	-0.90815377	0.18371445	-4.9432897	7.68E-07	5.34E-06	CAMKK1
158.6164846	0.386219577	0.14326992	2.6957478	0.0070231	0.021902032	ATP2A3
1246.148575	-0.1684231	0.06317616	-2.6659282	0.0076776	0.023630732	ZZEF1
190.8036385	0.850506093	0.15218617	5.5885898	2.29E-08	2.01E-07	CYB5D2
1120.957734	0.351665999	0.06808823	5.1648571	2.41E-07	1.82E-06	MYBBP1A
8.079146346	3.28257873	1.33906786	2.4513909	0.0142305	0.040535458	ALOX15
76.50835222	-0.62345771	0.20853879	-2.9896486	0.002793	0.009675303	CXCL16
1132.27889	-0.26177722	0.06090599	-4.2980535	1.72E-05	9.49E-05	PSMB6
1909.618613	0.186443658	0.04935484	3.7776164	0.0001583	0.000728625	MINK1

38.3185297	0.818359092	0.29243794	2.7984026	0.0051356	0.016590783	ENO3
2441.628041	-0.22667965	0.04835557	-4.6877674	2.76E-06	1.76E-05	RABEP1
1439.608734	-0.28552062	0.05271128	-5.416689	6.07E-08	5.01E-07	NUP88
849.4431792	0.277071402	0.06505322	4.2591494	2.05E-05	0.000111587	RPAIN
2190.256885	-0.23850853	0.05481151	-4.3514318	1.35E-05	7.61E-05	C1QBP
619.723939	0.806221924	0.08959931	8.998082	2.30E-19	6.42E-18	ENSG00000286190
425.3520374	0.392884948	0.09269988	4.2382464	2.25E-05	0.000121284	NLRP1
788.1789713	0.16695224	0.07021755	2.3776426	0.0174237	0.048094956	PIMREG
728.8271352	-0.3057949	0.07195422	-4.2498538	2.14E-05	0.000115561	KIAA0753
86.7323251	-0.52920961	0.19062448	-2.7761892	0.0055	0.01763698	ENSG00000282936
10.80448464	3.5931807	0.74049269	4.8524189	1.22E-06	8.22E-06	XAF1
3517.334737	0.472476785	0.04320858	10.934791	7.86E-28	4.31E-26	ACADVL
827.4690323	-0.27800284	0.06906894	-4.0250053	5.70E-05	0.000284706	DVL2
691.1860478	-0.24578408	0.07344757	-3.346388	0.0008187	0.003229922	CTDNEP1
6174.775326	-0.27029858	0.04445982	-6.079615	1.20E-09	1.26E-08	EIF5A
497.2783486	0.198791191	0.08137458	2.442915	0.0145692	0.04131732	NEURL4
407.0007464	0.225818571	0.09117564	2.4767424	0.0132588	0.038129612	KCTD11
14.74345085	1.56220926	0.53973867	2.8943809	0.0037991	0.012700084	TNK1
1314.969695	-0.18395598	0.0555831	-3.3095671	0.0009344	0.003636812	NLGN2
103.8330894	1.047289279	0.21314362	4.913538	8.94E-07	6.15E-06	ATP1B2
199.1015816	-0.39816952	0.12376583	-3.2171199	0.0012948	0.004861259	EFNB3
283.4976836	-0.37957566	0.10580973	-3.5873418	0.0003341	0.001434423	KDM6B
2968.562544	-0.34541956	0.04495425	-7.6838019	1.54E-14	2.74E-13	CHD3
69.64366626	-0.64221817	0.20734765	-3.0973014	0.0019529	0.007040037	KCNAB3
1021.173869	-0.26671401	0.06132982	-4.3488475	1.37E-05	7.69E-05	CNTROB
497.4106133	0.563624535	0.09468268	5.9527731	2.64E-09	2.63E-08	VAMP2
284.0127504	-0.52396492	0.10764943	-4.8673266	1.13E-06	7.65E-06	CTC1
1236.184805	-0.35179265	0.0563646	-6.2413762	4.34E-10	4.79E-09	PFAS
1504.320432	0.194457636	0.0561601	3.4625587	0.0005351	0.00220388	NDEL1
5465.200953	-0.57968023	0.04212409	-13.761253	4.36E-43	4.95E-41	MYH10
87.46366584	1.700747249	0.23598409	7.2070421	5.72E-13	8.55E-12	NTN1
288.8292619	-0.27399054	0.1101828	-2.4866905	0.0128938	0.037215324	STX8
77.84176757	-0.71376351	0.19063717	-3.7440942	0.000181	0.000826131	USP43
19.45050087	2.012850098	0.47564053	4.2318725	2.32E-05	0.000124466	MYH2
983.87896	-0.2224561	0.06142627	-3.6215143	0.0002929	0.001272357	MAP2K4
44.78370202	1.364896896	0.26898882	5.074177	3.89E-07	2.84E-06	LINC00670
602.8408356	1.774209103	0.09231823	19.218405	2.60E-82	1.00E-79	MYOCD
77.47440809	-0.71711619	0.2030627	-3.5315013	0.0004132	0.001747067	HS3ST3A1
353.9512555	-0.23124167	0.09666994	-2.3920742	0.0167535	0.046555219	COX10
1028.906383	1.029204556	0.07192872	14.308674	1.93E-46	2.45E-44	PMP22
909.2393437	0.541203464	0.06467623	8.3678884	5.87E-17	1.31E-15	TTC19
3427.14643	-0.37208445	0.04495009	-8.2777234	1.26E-16	2.71E-15	NCOR1
167.2081761	0.470907813	0.1517307	3.1035763	0.001912	0.006905325	PIGL
3675.668365	-0.3544281	0.04743105	-7.4724919	7.87E-14	1.29E-12	SNHG29
127.8368375	-0.70600732	0.16844959	-4.1912083	2.77E-05	0.000147201	ZNF624
73.88032928	0.504108004	0.20552347	2.4528002	0.0141749	0.040403476	PLD6
524.4565323	0.228957396	0.08369032	2.7357692	0.0062235	0.019698956	FLCN
3685.128162	-0.19962104	0.04805436	-4.1540675	3.27E-05	0.000171183	COPS3

25.42745917	1.03730328	0.39537006	2.6236263	0.0086999	0.02636201	RASD1
94.65202737	-0.58075167	0.1742034	-3.3337563	0.0008568	0.003363442	PEMT
1074.09892	-0.35892052	0.06252832	-5.7401271	9.46E-09	8.76E-08	RAI1
427.6599341	-0.85151559	0.08765622	-9.714263	2.62E-22	9.40E-21	SREBF1
1895.660253	0.624155748	0.0507703	12.293718	9.79E-35	7.85E-33	TOM1L2
573.4517683	-0.24565239	0.08027956	-3.0599618	0.0022137	0.007878009	GID4
2461.697416	-0.16871116	0.05039042	-3.3480801	0.0008137	0.003211722	ALKBH5
1025.095012	-0.29100366	0.06395218	-4.550332	5.36E-06	3.23E-05	LLGL1
1150.327972	-0.30752749	0.05923478	-5.1916712	2.08E-07	1.59E-06	SHMT1
8.664750483	1.790410802	0.64851062	2.7608041	0.0057659	0.018401436	LGALS9C
1130.397034	-0.21958242	0.06006059	-3.6560149	0.0002562	0.001128638	EPN2
178.9630078	-0.33331922	0.13116959	-2.5411318	0.0110494	0.032584287	B9D1
7.927725968	2.047238892	0.79599793	2.5719148	0.0101138	0.030136836	RNF112
2136.25564	0.591756686	0.05408235	10.941772	7.28E-28	4.02E-26	SPECC1
18.60462217	1.596384785	0.48708034	3.2774568	0.0010475	0.004022354	CCDC144NL-AS1
9729.90488	-0.20273281	0.04074019	-4.9762361	6.48E-07	4.56E-06	USP22
209.6185367	0.321743904	0.13113038	2.4536183	0.0141427	0.040348811	DHRS7B
551.190248	0.645014164	0.09206529	7.0060515	2.45E-12	3.40E-11	NATD1
1243.591042	0.344102219	0.06953481	4.9486323	7.47E-07	5.21E-06	MAP2K3
744.0583781	0.314585472	0.0881033	3.5706434	0.0003561	0.001522664	LINC02693
4108.871026	0.163969234	0.05123886	3.2000955	0.0013738	0.005124569	WSB1
263.9323816	0.913784899	0.11919694	7.6661773	1.77E-14	3.12E-13	KSR1
16.21925256	2.837045739	0.51137302	5.5478988	2.89E-08	2.50E-07	LGALS9
746.6813044	0.206656435	0.072372	2.855475	0.0042973	0.014145218	NLK
7.578376973	2.108421075	0.71550974	2.9467399	0.0032114	0.010935345	ENSG00000260777
2182.797243	0.124112964	0.05242959	2.3672314	0.0179217	0.049243404	TMEM97
2954.295922	0.320265576	0.04578807	6.9945194	2.66E-12	3.68E-11	TNFAIP1
487.6605024	0.877856808	0.09152411	9.5915359	8.68E-22	2.94E-20	SARM1
44.45357856	1.954010202	0.3628457	5.3852374	7.23E-08	5.91E-07	VTN
267.2134185	0.429085062	0.11049585	3.8832685	0.0001031	0.000490891	SLC46A1
524.5271596	0.474471366	0.08164071	5.8117009	6.18E-09	5.85E-08	UNC119
3309.588488	-0.34199092	0.04061936	-8.4194077	3.78E-17	8.58E-16	SPAG5
5392.264646	-0.27438873	0.04063688	-6.7522091	1.46E-11	1.86E-10	KIAA0100
756.1491487	0.21895731	0.07412312	2.9539679	0.0031372	0.010716033	SDF2
1193.080118	-0.17987536	0.05955282	-3.0204339	0.0025241	0.00884036	RAB34
1054.870657	-0.26342818	0.06135652	-4.2934017	1.76E-05	9.67E-05	RPL23A
769.323801	0.503348419	0.07343558	6.8542855	7.17E-12	9.46E-11	TRAF4
1078.231914	0.277976755	0.06653088	4.1781614	2.94E-05	0.000155192	FLOT2
134.2014478	-0.37233412	0.14818173	-2.5126857	0.0119816	0.034950338	DHRS13
1616.124391	-0.44830975	0.05338878	-8.3970778	4.58E-17	1.03E-15	MYO18A
5243.311989	-0.3974868	0.09561645	-4.1570966	3.22E-05	0.000169236	TAOK1
295.8170364	0.259654227	0.10446036	2.4856724	0.0129307	0.037315758	ABHD15
802.9638331	0.224313654	0.07453943	3.0093288	0.0026183	0.009124543	GIT1
73.06604041	0.687879798	0.21039399	3.2694841	0.0010774	0.004123381	CORO6
20.45282114	2.335991533	0.51514381	4.5346396	5.77E-06	3.46E-05	EFCAB5
595.4223161	-0.26458236	0.08023248	-3.2976965	0.0009748	0.003778031	CRLF3
1045.656629	-0.39833473	0.06137411	-6.4902726	8.57E-11	1.02E-09	ATAD5
3577.37383	-0.58123277	0.06229051	-9.3309997	1.05E-20	3.29E-19	NF1

26.47170741	-1.38705914	0.3542907	-3.9150312	9.04E-05	0.000435436	RAB11FIP4
15.20618618	2.799638726	0.61124677	4.5802102	4.65E-06	2.84E-05	ENSG00000263567
4486.573676	-0.21350595	0.04159997	-5.1323585	2.86E-07	2.13E-06	SUZ12
11.1778055	1.981333902	0.58691317	3.3758553	0.0007359	0.00292831	ENSG00000277511
1073.329901	-0.68830636	0.06166443	-11.162129	6.25E-29	3.70E-27	RHOT1
445.9020637	-0.45020278	0.08840106	-5.0927307	3.53E-07	2.60E-06	C17orf75
2729.68412	-0.24537298	0.04794648	-5.1176434	3.09E-07	2.29E-06	PSMD11
282.486377	0.342310121	0.11596827	2.9517566	0.0031597	0.010788829	MYO1D
8.549598973	1.961949173	0.66642606	2.9439863	0.0032401	0.011025606	CCL11
557.330506	-0.52716211	0.08286076	-6.3620235	1.99E-10	2.29E-09	LIG3
186.3162097	-0.35497591	0.13590053	-2.6120274	0.0090007	0.027164571	RFFL
786.3653905	1.558460394	0.08495932	18.343607	3.71E-75	1.13E-72	SLFN5
8317.349521	-0.08744319	0.03610341	-2.4220199	0.0154345	0.043382257	AP2B1
19.30414962	4.439609655	0.71983128	6.1675698	6.93E-10	7.45E-09	CCL5
814.5341661	0.189927835	0.07430393	2.5560941	0.0105854	0.031375655	PIGW
1436.125026	-0.18959963	0.05736519	-3.3051337	0.0009493	0.003689055	AATF
1789.326273	-0.52424123	0.05764612	-9.0941293	9.53E-20	2.75E-18	ACACA
1685.24556	0.314146603	0.05750203	5.4632262	4.68E-08	3.93E-07	DUSP14
1730.67431	-0.36124405	0.05655696	-6.3872611	1.69E-10	1.96E-09	SYNRG
885.393978	-0.17801544	0.06548099	-2.7185818	0.0065562	0.020643327	MRPL45
635.6772115	-0.20269468	0.0760993	-2.6635553	0.007732	0.023777008	SOCS7
890.0435575	-0.4954338	0.07198247	-6.8827008	5.87E-12	7.86E-11	MLLT6
645.7260832	-0.33093531	0.0759988	-4.3544806	1.33E-05	7.52E-05	PCGF2
12209.37421	-0.33609812	0.03564373	-9.4293769	4.13E-21	1.34E-19	RPL23
16.13958868	1.380084462	0.52902503	2.6087319	0.0090878	0.027389524	LINC00672
13031.42027	-0.17921508	0.03441721	-5.207135	1.92E-07	1.47E-06	RPL19
66.14758768	-1.16979954	0.21877626	-5.3470132	8.94E-08	7.22E-07	STAC2
798.8906868	-0.4134102	0.06737494	-6.135964	8.46E-10	8.98E-09	FBXL20
3555.635341	-0.33468601	0.04583758	-7.3015639	2.84E-13	4.40E-12	MED1
3636.867205	-0.21704666	0.04880175	-4.4475177	8.69E-06	5.07E-05	CDK12
611.6637254	0.230848261	0.07795327	2.9613674	0.0030628	0.010501076	STARD3
1076.333316	0.234119892	0.06644825	3.5233418	0.0004261	0.001796073	ORMDL3
397.8936574	-0.40988755	0.09352435	-4.3826829	1.17E-05	6.69E-05	THRA
2564.295967	-0.23816686	0.04403659	-5.4083852	6.36E-08	5.24E-07	MSL1
2583.342232	-0.18856209	0.04709963	-4.0034729	6.24E-05	0.000309781	CASC3
1491.957391	-0.23425886	0.05791468	-4.0448958	5.23E-05	0.00026317	WIPF2
60.61180047	0.660705206	0.23111684	2.8587498	0.0042531	0.014023916	ENSG00000266208
29008.9499	-0.23153806	0.03529718	-6.5596766	5.39E-11	6.55E-10	TOP2A
1389.537267	1.411449072	0.06367062	22.167982	7.00E-109	5.29E-106	IGFBP4
4.004856635	3.482129203	1.25375575	2.7773585	0.0054803	0.017583374	KRTAP1-5
378.0887686	-0.27749745	0.10593243	-2.6195704	0.0088041	0.026631169	KRTAP2-3
136.7571996	1.563155647	0.16962593	9.2153105	3.10E-20	9.35E-19	KRT17
7292.784326	-0.23505303	0.03961273	-5.9337754	2.96E-09	2.92E-08	EIF1
84.75853769	2.833942509	0.3304742	8.575382	9.88E-18	2.37E-16	HAP1
266.8338085	1.125869481	0.13117922	8.5826819	9.27E-18	2.23E-16	JUP
2099.055368	0.302217156	0.04936477	6.1221226	9.23E-10	9.74E-09	CNP
2417.349585	-0.15618894	0.04748017	-3.2895619	0.0010034	0.003875995	DNAJC7
1314.933269	0.222548753	0.06033763	3.6883907	0.0002257	0.001006546	NKIRAS2

9.026378392	2.733447807	0.7693974	3.5527126	0.0003813	0.001619163	DHX58
708.6426492	0.300783582	0.07029584	4.2788245	1.88E-05	0.000102745	KAT2A
700.3781371	0.190886986	0.07190841	2.6545852	0.0079406	0.024319658	RAB5C
3796.757358	0.35475365	0.04404209	8.0548771	7.96E-16	1.62E-14	STAT3
1064.065987	0.349522621	0.06407006	5.4553194	4.89E-08	4.09E-07	ATP6V0A1
99.53463527	0.739041482	0.18704127	3.9512214	7.78E-05	0.000379279	NAGLU
918.3352632	-0.38839114	0.06694972	-5.8012357	6.58E-09	6.21E-08	COASY
385.1768602	0.270407283	0.10701753	2.5267569	0.0115121	0.033776903	TUBG2
916.6786792	0.272017824	0.06887173	3.9496298	7.83E-05	0.000381596	CNTNAP1
485.3882673	-0.31441818	0.09162773	-3.4314739	0.0006003	0.002447692	EZH1
285.1730406	-0.37624581	0.11002431	-3.4196608	0.000627	0.002540994	COA3
1581.580657	0.225709148	0.05493305	4.1088045	3.98E-05	0.000204925	BECN1
79.05710708	0.898907195	0.21669109	4.1483348	3.35E-05	0.000175315	AOC2
29.33365621	1.660039552	0.36114339	4.5966217	4.29E-06	2.65E-05	AOC3
7136.240651	-0.20771784	0.0451363	-4.6020128	4.18E-06	2.58E-05	RPL27
232.1724944	1.221488019	0.12435301	9.822746	8.99E-23	3.39E-21	IFI35
2074.04395	0.299334339	0.0502215	5.9602824	2.52E-09	2.52E-08	VAT1
2494.533485	-0.42699369	0.04417397	-9.6661842	4.20E-22	1.48E-20	BRCA1
4039.820518	-0.29910764	0.04693631	-6.3726277	1.86E-10	2.14E-09	NBR1
119.8948406	0.386968538	0.16258677	2.380074	0.0173092	0.047831933	TMEM106A
256.1131858	0.596193337	0.12299138	4.8474399	1.25E-06	8.41E-06	ARL4D
145.6416858	-0.38614045	0.14014249	-2.7553417	0.0058631	0.018670406	ETV4
2270.819057	-0.18441521	0.05007062	-3.683102	0.0002304	0.001025827	DUSP3
340.256939	-0.34881631	0.10285466	-3.3913517	0.0006955	0.002785476	TMEM101
392.6333492	0.281017339	0.09286685	3.0260242	0.0024779	0.008694737	G6PC3
224.7412555	0.344516912	0.12796817	2.6922078	0.0070981	0.022097289	ASB16-AS1
28.74707271	0.838815581	0.34299328	2.4455744	0.0144622	0.041074147	RUNDC3A-AS1
3427.083305	0.533280431	0.04818367	11.067659	1.80E-28	1.05E-26	GRN
1663.109822	-0.1925969	0.05545798	-3.4728436	0.000515	0.002129708	GPATCH8
1217.029715	-0.18577905	0.06230385	-2.9818231	0.0028654	0.009898466	FZD2
3238.951572	-0.27565885	0.05659266	-4.8709291	1.11E-06	7.53E-06	GJC1
3727.727154	-0.21085192	0.04036878	-5.2231433	1.76E-07	1.36E-06	EFTUD2
3143.792665	-0.15458237	0.04134428	-3.7389056	0.0001848	0.000841148	NMT1
129.526123	-0.45860913	0.17716536	-2.5885937	0.0096369	0.028903965	C1QL1
757.1417842	0.420948295	0.07082208	5.9437435	2.79E-09	2.76E-08	PLCD3
63.04798665	0.70405483	0.24530619	2.8701063	0.0041033	0.013589232	ACBD4
1912.618553	0.420562407	0.05366198	7.837251	4.61E-15	8.53E-14	HEXIM1
24.6564368	0.897389095	0.37850529	2.3708759	0.017746	0.048814095	MAP3K14-AS1
522.6264455	0.318633216	0.08762076	3.6365036	0.0002764	0.001208136	MAP3K14
183.7647353	0.679308214	0.13679459	4.9658995	6.84E-07	4.79E-06	ARHGAP27
423.1919079	0.250256822	0.09124438	2.7427094	0.0060935	0.019335873	PLEKHM1
260.8505034	-0.57279406	0.11505099	-4.9786105	6.40E-07	4.51E-06	LINC02210
5.422223303	3.501633846	1.19468554	2.9310088	0.0033786	0.011441096	WNT9B
1093.255751	0.257734619	0.05934943	4.3426635	1.41E-05	7.88E-05	GOSR2
548.0154738	-0.27683072	0.08075918	-3.4278546	0.0006084	0.002474754	ENSG00000262879
4122.214536	-0.29691621	0.04782644	-6.2082022	5.36E-10	5.85E-09	CDC27
1148.320722	2.230514455	0.08599828	25.936734	2.57E-148	5.58E-145	ITGB3
3547.795834	-0.39289488	0.04249668	-9.2453078	2.35E-20	7.16E-19	NPEPPS

19755.45422	-0.34990708	0.03109433	-11.253084	2.24E-29	1.34E-27	KPNB1
202.4636169	0.550199112	0.13772852	3.994809	6.47E-05	0.000320416	OSBPL7
391.9015429	-0.23873532	0.09180577	-2.6004392	0.0093105	0.028011891	SP2
243.6851554	0.311972226	0.1158647	2.6925562	0.0070907	0.022078165	COPZ2
5121.171785	0.19644331	0.03751065	5.2370009	1.63E-07	1.27E-06	NFE2L1
5054.177142	-0.1680539	0.04285862	-3.9211226	8.81E-05	0.000425516	CBX1
413.0553982	0.323029151	0.08985866	3.5948584	0.0003246	0.001397434	SNX11
134.179465	-0.42651255	0.15060642	-2.831968	0.0046262	0.015113742	HOXB2
157.7654332	-0.41525082	0.13938884	-2.9790823	0.0028911	0.009975551	HOXB3
35.98971766	-0.98673232	0.28928981	-3.4108782	0.0006475	0.002612697	HOXB9
9.661165191	1.586736027	0.59667806	2.6592833	0.0078307	0.024042373	LINC02086
1849.237208	-0.18851566	0.05201233	-3.6244415	0.0002896	0.001259292	CALCOCO2
1848.04342	-0.38647755	0.05682115	-6.8016497	1.03E-11	1.35E-10	IGF2BP1
41.57803106	1.759554234	0.343593	5.1210422	3.04E-07	2.26E-06	B4GALNT2
32.15657376	0.950126134	0.31427027	3.0232772	0.0025005	0.008766983	GNGT2
17.38083756	1.299737182	0.46081463	2.8205206	0.0047946	0.015616731	PHOSPHO1
80.5122836	2.041738862	0.26348709	7.7489143	9.27E-15	1.68E-13	NGFR
31.9146414	0.957266059	0.30969825	3.0909637	0.0019951	0.00717272	NXPH3
1457.958972	-0.30895836	0.05399932	-5.7215228	1.06E-08	9.69E-08	KAT7
13.36137343	2.004614031	0.635766	3.1530689	0.0016156	0.005947485	DLX3
5421.78387	0.262537291	0.03720061	7.0573377	1.70E-12	2.39E-11	ITGA3
46071.67356	0.146647195	0.04800766	3.0546622	0.0022531	0.007999618	COL1A1
15.90134746	1.513229549	0.47230496	3.2039248	0.0013557	0.005064502	TMEM92
550.0851019	0.617262977	0.08475825	7.28263	3.27E-13	5.04E-12	ACSF2
340.6523046	-0.29571021	0.10390506	-2.8459655	0.0044277	0.01454708	RSAD1
586.9320527	0.274382692	0.07785344	3.5243488	0.0004245	0.001790128	SPATA20
69.49342947	1.220108207	0.25039799	4.8726758	1.10E-06	7.47E-06	ABCC3
4847.982539	0.241104099	0.0487378	4.9469634	7.54E-07	5.26E-06	LUC7L3
871.5282367	0.377623603	0.0668586	5.6480934	1.62E-08	1.45E-07	TOB1
5892.16692	0.18481289	0.03827649	4.8283658	1.38E-06	9.21E-06	SPAG9
504.5520614	-0.36285774	0.08756607	-4.1438166	3.42E-05	0.000178323	MBTD1
1135.939895	-0.243584	0.06064552	-4.0165208	5.91E-05	0.00029464	UTP18
676.1779116	-0.39493479	0.07489875	-5.2729153	1.34E-07	1.05E-06	TOM1L1
77.58020503	0.494511289	0.20056217	2.4656259	0.0136774	0.039158736	ENSG00000279059
373.2700123	-0.5340674	0.10245717	-5.2125917	1.86E-07	1.43E-06	STXBP4
28.17607331	-1.26360116	0.32705552	-3.8635677	0.0001117	0.000527818	HLF
263.9858601	-0.78434628	0.11532701	-6.8010633	1.04E-11	1.35E-10	NOG
2115.151333	0.506720024	0.05829544	8.6922748	3.55E-18	8.90E-17	TRIM25
743.8411382	-0.37732704	0.06835897	-5.5197884	3.39E-08	2.90E-07	COIL
11.67388064	1.468287621	0.5571466	2.6353703	0.0084046	0.025605323	AKAP1-DT
1154.588584	-0.32189139	0.05770715	-5.5780158	2.43E-08	2.13E-07	MSI2
546.2802722	0.25376014	0.08031588	3.1595264	0.0015803	0.005827116	CUEDC1
1714.769426	-0.44370354	0.05060431	-8.7680979	1.82E-18	4.65E-17	VEZF1
594.9307211	0.94599722	0.27087019	3.4924375	0.0004786	0.001996507	ENSG00000264112
72.95993654	0.574935734	0.21259477	2.7043738	0.0068433	0.021426975	ENSG00000279207
1201.848652	-0.77598012	0.05867421	-13.225234	6.27E-40	6.31E-38	MTMR4
1962.773979	-0.24822877	0.05788237	-4.2885039	1.80E-05	9.88E-05	SKA2
5818.459497	-0.30928767	0.044309	-6.9802444	2.95E-12	4.05E-11	PRR11

30.58066515	-0.80896719	0.33454804	-2.4180898	0.0156022	0.04378998	GDPD1
1699.276893	-0.29591964	0.05087566	-5.8165264	6.01E-09	5.69E-08	DHX40
17250.73597	-0.29422216	0.03699007	-7.954085	1.80E-15	3.51E-14	CLTC
606.2009996	0.311247065	0.07861893	3.9589327	7.53E-05	0.000368274	PTRH2
3767.935711	0.241755777	0.0416188	5.8088124	6.29E-09	5.95E-08	VMP1
1376.968767	-0.18362604	0.05401123	-3.3997754	0.0006744	0.002711683	RPS6KB1
703.4045517	-0.33084649	0.07408626	-4.4656927	7.98E-06	4.69E-05	HEATR6
85.66293161	0.868960525	0.20377193	4.2643781	2.00E-05	0.00010921	WFDC21P
35.92138646	0.964814105	0.31148875	3.0974284	0.0019521	0.007038481	ENSG00000267248
1402.907628	-0.3275846	0.05662522	-5.7851363	7.25E-09	6.81E-08	USP32
1711.700408	-0.25268601	0.05692091	-4.4392478	9.03E-06	5.26E-05	APPBP2
810.0093783	0.397031839	0.07685149	5.1662218	2.39E-07	1.81E-06	PPM1D
303.2627853	-0.35143468	0.11138236	-3.1552096	0.0016038	0.005906518	BCAS3
72.80221468	0.553340589	0.20663052	2.6779228	0.007408	0.022918053	TBX2
928.5159276	-0.22882273	0.06250877	-3.6606502	0.0002516	0.001111228	INTS2
2999.49356	-0.33448703	0.05741003	-5.8262823	5.67E-09	5.38E-08	MED13
2322.812024	-0.48247755	0.05847053	-8.251637	1.56E-16	3.35E-15	TANC2
2122.859281	-0.27744796	0.05703219	-4.8647608	1.15E-06	7.75E-06	DCAF7
4752.47725	0.105630205	0.03997922	2.6421277	0.0082387	0.025144083	DDX42
2930.703412	-0.14653095	0.04151368	-3.5297027	0.000416	0.001757703	PSMC5
244.7955551	0.748425015	0.11908928	6.2845709	3.29E-10	3.68E-09	ERN1
2219.120557	0.188395862	0.05560696	3.3879909	0.0007041	0.002816588	TEX2
16962.45198	0.096537799	0.03054233	3.1607872	0.0015734	0.005804416	DDX5
5914.161042	-0.12500652	0.05058586	-2.4711754	0.013467	0.038638956	SMURF2
2947.757221	-0.21554512	0.0406151	-5.3070193	1.11E-07	8.86E-07	GNA13
192.3852555	-0.33433447	0.13135076	-2.5453562	0.0109166	0.03224247	CEP112
4188.862307	-0.88935559	0.04564722	-19.483236	1.52E-84	6.62E-82	PRKCA
1909.046542	-0.24764908	0.05982255	-4.1397278	3.48E-05	0.000181042	HELZ
14.23196497	1.334847042	0.55038325	2.4253046	0.0152955	0.043061284	ENSG00000285877
3264.963556	-0.20338184	0.04673078	-4.3522032	1.35E-05	7.59E-05	NOL11
5852.406241	-0.47281308	0.04102716	-11.524391	9.94E-31	6.47E-29	BPTF
344.0840217	-0.48297594	0.09590813	-5.0358185	4.76E-07	3.43E-06	C17orf58
18201.514	0.088256645	0.03574765	2.46888	0.0135537	0.038862042	KPNA2
452.1171006	-0.59355561	0.08826315	-6.7248407	1.76E-11	2.22E-10	LINC00674
883.2633521	-0.49363862	0.06885071	-7.1696953	7.52E-13	1.11E-11	ENSG00000278730
43.76194105	0.662171747	0.27939479	2.3700218	0.017787	0.048903808	SLC16A6
6957.611365	-0.24508234	0.04606983	-5.3198011	1.04E-07	8.30E-07	PRKAR1A
26.64522038	1.133775154	0.36327623	3.1209726	0.0018025	0.006556433	ABCA10
1443.540599	0.409269724	0.05647656	7.2467183	4.27E-13	6.48E-12	ABCA5
57.4987781	-0.88679611	0.24860233	-3.5671272	0.0003609	0.00154134	MAP2K6
87.16726584	2.022920812	0.23702683	8.5345648	1.41E-17	3.32E-16	KCNJ2
259.0899831	1.105302181	0.13408916	8.2430393	1.68E-16	3.58E-15	SOX9
75.11754804	1.400664811	0.23337038	6.0018963	1.95E-09	1.98E-08	LINC00511
207.3661499	-0.34775079	0.12234809	-2.8423068	0.0044788	0.01469843	SLC39A11
29.95078958	2.579008248	0.48547536	5.3123361	1.08E-07	8.63E-07	SSTR2
6717.772552	-0.11479369	0.04703965	-2.4403602	0.0146726	0.041590371	RPL38
135.685747	0.535649261	0.16239394	3.2984561	0.0009722	0.003769504	GPRC5C
1957.257416	0.450617892	0.05630949	8.0025207	1.22E-15	2.42E-14	SLC9A3R1

573.5791011	0.226196416	0.07601724	2.9755936	0.0029242	0.010077727	NAT9
566.4804711	0.328951474	0.07791912	4.2217041	2.42E-05	0.000129736	TMEM104
11.83611338	1.654739853	0.64936444	2.5482453	0.0108266	0.032025028	GRIN2C
587.5416669	1.057894895	0.1027395	10.296866	7.28E-25	3.20E-23	FDXR
533.5964068	0.624033507	0.08471056	7.3666556	1.75E-13	2.78E-12	CDR2L
363.8428974	0.274754136	0.09632354	2.8524089	0.0043389	0.014268892	ARMC7
198.0731449	-0.37726844	0.1238233	-3.0468291	0.0023127	0.008179318	NT5C
5629.618989	0.423382002	0.04556414	9.2920008	1.51E-20	4.69E-19	JPT1
3165.771093	-0.23161344	0.04935052	-4.6932317	2.69E-06	1.72E-05	SUMO2
1743.34888	-0.12841151	0.05069936	-2.5328032	0.0113154	0.033261602	NUP85
154.4995283	-0.51738796	0.14662043	-3.5287576	0.0004175	0.001762283	MIF4GD
665.2266903	-0.23780929	0.07565212	-3.1434582	0.0016696	0.006124253	TMEM94
632.5472046	-0.27347385	0.0733237	-3.7296787	0.0001917	0.000868477	CASKIN2
10193.09952	-0.39581776	0.03242729	-12.206317	2.88E-34	2.27E-32	H3-3B
732.412399	-0.3426297	0.07214429	-4.7492282	2.04E-06	1.32E-05	UNK
20.39520009	1.290933631	0.41870108	3.0831867	0.002048	0.007346148	UNC13D
57.04034135	0.809669903	0.23168889	3.4946427	0.0004747	0.001981511	TRIM47
4021.768419	-0.13595891	0.03979979	-3.4160708	0.0006353	0.002569342	SRP68
60.91043518	1.039948062	0.24413912	4.2596536	2.05E-05	0.00011137	QRICH2
744.7786781	0.693774958	0.0714612	9.708415	2.78E-22	9.89E-21	SPHK1
343.561149	0.472298616	0.10212246	4.6248259	3.75E-06	2.33E-05	RHBDF2
2291.996219	-0.25199851	0.06255448	-4.0284647	5.61E-05	0.000280873	SNHG16
5096.373263	0.1994215	0.04312322	4.6244576	3.76E-06	2.34E-05	MXRA7
467.0713703	0.225063667	0.08514134	2.6434125	0.0082075	0.025062054	MFSD11
6399.088646	-0.24839918	0.03845836	-6.4589131	1.05E-10	1.24E-09	SEC14L1
4511.878996	-0.373493	0.04062022	-9.1947553	3.76E-20	1.12E-18	SEPTIN9
536.3419506	0.227551101	0.07922658	2.8721559	0.0040768	0.013514269	AFMID
5111.782075	-0.1441751	0.04959374	-2.9071231	0.0036477	0.012243476	BIRC5
492.7731307	-0.3646698	0.08661615	-4.2101825	2.55E-05	0.00013603	SOCS3
5.736325916	3.045023415	1.01986151	2.9857225	0.0028291	0.009788042	DNAH17
751.8831052	0.53126843	0.07049375	7.5363908	4.83E-14	8.16E-13	CYTH1
1310.231946	0.144292491	0.05400904	2.6716359	0.0075482	0.023294421	USP36
15860.27321	0.476034362	0.03537799	13.455663	2.85E-41	3.01E-39	TIMP2
56.54763072	1.441397427	0.25853954	5.5751528	2.47E-08	2.16E-07	CEP295NL
4537.604738	1.110135576	0.05425786	20.460362	4.86E-93	2.72E-90	LGALS3BP
17.47436893	2.849885688	0.58105959	4.9046359	9.36E-07	6.41E-06	C1QTNF1
514.849642	0.387579843	0.08834082	4.3873245	1.15E-05	6.56E-05	CBX4
901.5465057	0.263686286	0.08049758	3.2757044	0.001054	0.004043426	TBC1D16
471.1211981	0.3883463	0.10027091	3.8729705	0.0001075	0.000509738	GAA
96.80307435	0.466902012	0.18815981	2.481412	0.0130863	0.037708495	ENSG00000262580
601.7060215	0.388429928	0.07873182	4.9335825	8.07E-07	5.59E-06	SGSH
228.9960136	0.526983061	0.12076749	4.3636169	1.28E-05	7.24E-05	SLC26A11
22.34035593	-0.88060734	0.35393078	-2.4880779	0.0128436	0.037107412	RNF213-AS1
216.4011495	0.313895792	0.12850165	2.4427375	0.0145763	0.041330906	ENDOV
770.6469289	1.064379223	0.07397298	14.388757	6.09E-47	7.90E-45	NPTX1
386.6758797	-0.6803793	0.10053755	-6.7674146	1.31E-11	1.68E-10	BAIAP2-DT
759.6066628	0.328857841	0.07063464	4.6557588	3.23E-06	2.03E-05	BAIAP2
337.236238	0.414914232	0.09824812	4.2231264	2.41E-05	0.000128999	TEPSIN

1615.60493	0.440624207	0.05221424	8.4387745	3.21E-17	7.30E-16	SLC38A10
108885.0499	-0.11213809	0.03513257	-3.1918551	0.0014136	0.005258373	ACTG1
25.17438625	1.098608498	0.41382867	2.6547423	0.0079369	0.024312614	ENSG00000289182
8.720872781	2.651622473	0.82289827	3.2222968	0.0012717	0.004787703	FSCN2
1522.932496	0.349509727	0.05651835	6.1840041	6.25E-10	6.76E-09	CCDC137
6090.166485	-0.15902056	0.03525502	-4.5105797	6.47E-06	3.84E-05	ARHGDIA
2207.54967	-0.34238755	0.05067868	-6.7560476	1.42E-11	1.82E-10	ALYREF
634.8372316	0.200173727	0.07575468	2.6423942	0.0082322	0.02512871	PCYT2
589.7169422	0.478855916	0.07906515	6.0564729	1.39E-09	1.44E-08	SIRT7
1088.468027	0.211879126	0.06343828	3.3399254	0.000838	0.003297799	MAFG
953.4968949	-0.28066148	0.06389128	-4.392798	1.12E-05	6.42E-05	PYCR1
264.7463003	-0.31590463	0.12262583	-2.5761671	0.0099902	0.02980446	RAC3
910.4170685	0.308783681	0.06266255	4.9277226	8.32E-07	5.75E-06	DUS1L
3402.425363	-0.47950362	0.04509921	-10.632197	2.11E-26	1.05E-24	FASN
258.7363657	-0.38201288	0.12021568	-3.1777293	0.0014843	0.005495538	CCDC57
30.60694895	1.002422335	0.38987825	2.5711163	0.0101371	0.030180557	ENSG00000275888
77.84146923	1.173697699	0.2558042	4.588266	4.47E-06	2.75E-05	ENSG00000280407
69.47219793	1.087041891	0.21796393	4.9872558	6.12E-07	4.33E-06	SECTM1
4018.484134	-0.11808983	0.0456902	-2.5845767	0.0097499	0.029207592	WDR45B
657.1700425	0.202624594	0.07988965	2.536306	0.0112029	0.032980576	METRNL
514.1155157	-0.36933316	0.08594228	-4.2974562	1.73E-05	9.52E-05	THOC1
258.301402	-0.57811949	0.1150997	-5.0227717	5.09E-07	3.66E-06	METTL4
2397.299147	-0.29376519	0.05862359	-5.0110409	5.41E-07	3.86E-06	SMCHD1
53.81589721	1.074513398	0.3090487	3.4768416	0.0005074	0.002102704	EMILIN2
3536.54821	0.704954982	0.05136586	13.724194	7.27E-43	8.21E-41	MYL12A
4232.660935	0.405460227	0.04614889	8.7859151	1.55E-18	3.99E-17	MYL12B
453.3356704	0.419530489	0.09703539	4.3234794	1.54E-05	8.54E-05	TGIF1
237.2656418	-0.33714559	0.11735759	-2.8728061	0.0040684	0.013494205	ZBTB14
887.7151767	-0.19995255	0.06673593	-2.9961755	0.0027339	0.009485706	MTCL1
1416.029783	-0.45738877	0.05831135	-7.8439062	4.37E-15	8.12E-14	ANKRD12
21.89253344	-1.17359345	0.38158157	-3.075603	0.0021008	0.007513872	ENSG00000273284
1554.700507	-0.26572135	0.05458476	-4.8680499	1.13E-06	7.63E-06	PPP4R1
537.780879	0.297555294	0.08743882	3.4030114	0.0006665	0.00268225	RAB31
1854.369412	-0.25645859	0.04899564	-5.2343144	1.66E-07	1.28E-06	VAPA
3663.796497	0.17905532	0.04135804	4.3293957	1.50E-05	8.34E-05	TUBB6
11.32650241	1.510115505	0.61489141	2.455906	0.014053	0.040128327	ENSG00000267199
1153.781465	0.326427238	0.06501725	5.0206251	5.15E-07	3.69E-06	SPIRE1
467.9267954	-0.22714017	0.08662743	-2.6220353	0.0087406	0.026476171	PSMG2
946.4322998	-0.36160607	0.06451945	-5.604606	2.09E-08	1.84E-07	SEH1L
856.1985546	-0.34560867	0.06742935	-5.1254932	2.97E-07	2.21E-06	CEP192
485.2408619	-0.3983607	0.09374713	-4.2493108	2.14E-05	0.000115792	FAM210A
1132.952028	-0.37941481	0.05715413	-6.6384494	3.17E-11	3.92E-10	RNMT
165.618076	0.393045357	0.15717122	2.5007463	0.0123932	0.035980432	ANKRD20A5P
1232.120416	-0.31448203	0.05722892	-5.4951593	3.90E-08	3.31E-07	SNRPD1
223.5730882	0.330783119	0.12422226	2.6628328	0.0077486	0.023823901	ABHD3
1586.98948	-0.41354863	0.06445318	-6.4162643	1.40E-10	1.63E-09	MIB1
117.2733055	1.41585242	0.18711797	7.5666299	3.83E-14	6.54E-13	GATA6
156.3191488	1.013287504	0.15993055	6.3357969	2.36E-10	2.69E-09	CABLES1

1325.908785	0.273557985	0.05717482	4.7845884	1.71E-06	1.13E-05	RIOK3
458.0719111	0.263748918	0.08843923	2.9822616	0.0028613	0.009886261	RMC1
809.8861365	0.208340543	0.06822658	3.0536564	0.0022607	0.008019936	NPC1
14.37398064	2.209938432	0.60684224	3.6417017	0.0002708	0.001186084	ANKRD29
47.07155074	0.720651296	0.26463541	2.7231855	0.0064656	0.020379978	LAMA3
4215.32203	0.492977596	0.04792132	10.287228	8.05E-25	3.52E-23	CDH2
3527.290829	0.31327727	0.05068548	6.1808092	6.38E-10	6.88E-09	DSC3
3210.084645	0.326218122	0.04968993	6.5650747	5.20E-11	6.34E-10	DSG2
214.3389949	-0.39147828	0.12863589	-3.0433053	0.0023399	0.008260595	B4GALT6
928.2286767	-0.17588129	0.06514919	-2.6996697	0.0069408	0.021701028	TRAPPC8
618.7704346	-0.18865682	0.07680991	-2.456152	0.0140434	0.040121827	RNF138
85.35839338	0.898119043	0.22618921	3.9706538	7.17E-05	0.000352201	GAREM1
476.6125266	0.861705507	0.09944849	8.6648421	4.52E-18	1.12E-16	MAPRE2
1812.577357	-0.23077537	0.04905361	-4.7045538	2.54E-06	1.63E-05	ZNF24
2978.417222	0.37435047	0.05060114	7.398064	1.38E-13	2.22E-12	GALNT1
916.8490811	-0.15791422	0.06333985	-2.4931258	0.0126624	0.036638779	ELP2
204.0762724	-0.40410804	0.12378489	-3.264599	0.0010962	0.00419054	MOCOS
147.6430734	0.473296905	0.14784769	3.2012464	0.0013683	0.005107433	FHOD3
55.83051917	-0.88952691	0.23267607	-3.8230271	0.0001318	0.000614586	KIAA1328
846.2246198	-0.23218573	0.06767112	-3.4310902	0.0006012	0.002450582	PIK3C3
1063.945246	-0.35912074	0.07855454	-4.5716104	4.84E-06	2.95E-05	EPG5
124.0001672	0.727356494	0.1599264	4.5480702	5.41E-06	3.26E-05	PSTPIP2
3951.483184	-0.21152609	0.04502954	-4.6974966	2.63E-06	1.68E-05	ATP5F1A
14.10441385	1.648020789	0.53216446	3.0968261	0.001956	0.007049873	KATNAL2
1399.832294	-0.28719308	0.05631898	-5.0994013	3.41E-07	2.51E-06	SMAD2
507.405877	-0.43207483	0.08751551	-4.937123	7.93E-07	5.50E-06	CTIF
252.7062967	0.526573001	0.12906092	4.0800345	4.50E-05	0.00022977	SMAD7
494.1011513	-0.40900252	0.08194542	-4.9911581	6.00E-07	4.25E-06	DYM
4.405849525	3.726959544	1.38613586	2.6887404	0.0071722	0.022288213	LIPG
331.8667221	0.436275492	0.09932812	4.3922655	1.12E-05	6.43E-05	ACAA2
783.7297815	-0.38045441	0.06783002	-5.6089382	2.04E-08	1.80E-07	SMAD4
467.776916	-0.42387046	0.08326992	-5.090319	3.57E-07	2.62E-06	MEX3C
1010.044423	-0.16348432	0.06064139	-2.6959198	0.0070195	0.021899598	MBD2
745.2936703	-0.23245591	0.06867181	-3.3850268	0.0007117	0.002840645	C18orf54
40.16175269	1.317888529	0.31392495	4.1981007	2.69E-05	0.000143011	RAB27B
788.257038	-0.27103704	0.06901495	-3.927222	8.59E-05	0.000415796	TXNL1
380.1585289	-0.44364366	0.09701803	-4.5727961	4.81E-06	2.93E-05	WDR7
3536.242432	-0.3960677	0.05421323	-7.3057392	2.76E-13	4.28E-12	ATP8B1
2987.94721	0.446522448	0.05010131	8.9123915	4.99E-19	1.36E-17	NEDD4L
1422.417661	0.287184184	0.0584047	4.9171414	8.78E-07	6.04E-06	ALPK2
343.7678412	-0.37621319	0.0962772	-3.907604	9.32E-05	0.000448107	ZNF532
247.5422131	0.302581593	0.11314467	2.6742895	0.0074888	0.023139631	SEC11C
4784.609654	-0.43250412	0.04239873	-10.200875	1.96E-24	8.33E-23	LMAN1
2876.845575	-0.80788249	0.04374147	-18.469487	3.64E-76	1.15E-73	CCBE1
569.0201976	1.313307196	0.08265115	15.889763	7.46E-57	1.40E-54	PMAIP1
106.5190298	0.805723019	0.18974528	4.2463403	2.17E-05	0.000117205	RNF152
474.3065956	-0.57277701	0.08362061	-6.8497108	7.40E-12	9.74E-11	PIGN
622.624594	0.200050397	0.07708684	2.5951302	0.0094555	0.028394284	RELCH

835.0218884	0.320682203	0.06846838	4.6836539	2.82E-06	1.79E-05	KDSR
8.289860494	1.51479615	0.63363326	2.3906513	0.0168185	0.046683807	SERPINB2
281.1320468	0.974080952	0.11749396	8.2904769	1.13E-16	2.44E-15	SERPINB8
446.3301729	-0.53121554	0.09093728	-5.8415597	5.17E-09	4.93E-08	RTTN
420.1843079	-0.22654918	0.08797289	-2.5752159	0.0100178	0.029876305	TIMM21
21.55002635	-0.98910066	0.38299247	-2.582559	0.0098071	0.029343556	TSHZ1
224.9362261	-0.54636812	0.11498513	-4.7516417	2.02E-06	1.31E-05	ZNF516
206.7696568	-0.32019359	0.12569175	-2.547451	0.0108513	0.032081647	ZNF236
533.3155137	0.244167765	0.08115857	3.0085273	0.0026252	0.009146814	MIER2
860.8619665	0.219523511	0.0663495	3.3085932	0.0009377	0.003647853	CDC34
9602.302485	0.297198449	0.03724135	7.9803342	1.46E-15	2.88E-14	BSG
531.246345	0.220169106	0.08141839	2.7041693	0.0068475	0.021436314	HCN2
562.3979026	0.199773004	0.07899778	2.5288433	0.0114439	0.033601475	RNF126
1126.476897	0.668759159	0.06682891	10.007035	1.42E-23	5.53E-22	FSTL3
60.39323144	1.445214595	0.25328014	5.7059925	1.16E-08	1.06E-07	MISP
6505.393745	-0.27433067	0.03795376	-7.2280242	4.90E-13	7.38E-12	PTBP1
569.5969206	-0.33718492	0.07738719	-4.3571156	1.32E-05	7.44E-05	MED16
1654.22838	0.290639189	0.05629499	5.1627893	2.43E-07	1.84E-06	TMEM259
9744.233221	0.350894392	0.03750742	9.355333	8.33E-21	2.63E-19	CNN2
401.901092	1.089423739	0.10680976	10.199665	1.99E-24	8.42E-23	ABCA7
34.19622321	1.154459814	0.34633394	3.3333719	0.000858	0.003367333	ARHGAP45
3084.036656	0.207293175	0.04780891	4.3358691	1.45E-05	8.12E-05	GPX4
2695.175921	0.275715407	0.05358253	5.1456214	2.67E-07	2.00E-06	CIRBP
1243.889396	-0.21786378	0.05942899	-3.6659512	0.0002464	0.001090119	PWWP3A
488.4362667	0.377892227	0.08576565	4.4061023	1.05E-05	6.06E-05	GAMT
2721.566219	-0.25662823	0.04346084	-5.9048156	3.53E-09	3.44E-08	DAZAP1
4672.957003	-0.17125439	0.04288628	-3.9932208	6.52E-05	0.000322479	RPS15
243.493137	0.318148207	0.12238632	2.5995405	0.0093349	0.028080491	ENSG00000267317
172.3142246	0.706779432	0.1472152	4.8009948	1.58E-06	1.05E-05	REEP6
538.3033291	-0.37249584	0.07974612	-4.6710216	3.00E-06	1.90E-05	MEX3D
1478.349102	-0.47486589	0.05250129	-9.0448418	1.50E-19	4.25E-18	TCF3
148.1662342	-0.83233818	0.13961919	-5.9614886	2.50E-09	2.51E-08	ATP8B3
415.2858557	0.579156527	0.09366997	6.1829477	6.29E-10	6.80E-09	ABHD17A
1041.250434	0.239975956	0.06272077	3.8261005	0.0001302	0.000607782	SCAMP4
32.63141412	0.947754839	0.33363879	2.8406614	0.004502	0.014754979	IZUMO4
957.19426	-0.37597685	0.06708673	-5.6043404	2.09E-08	1.85E-07	DOT1L
261.0235046	0.512697571	0.12924288	3.966931	7.28E-05	0.000357086	SPPL2B
4077.621204	-0.47196761	0.04064099	-11.613095	3.54E-31	2.36E-29	LMNB2
978.021439	-0.21335926	0.07746806	-2.7541577	0.0058843	0.018734653	GADD45B
1196.549356	0.157242496	0.0581053	2.7061645	0.0068065	0.021334821	SGTA
136.6051051	-0.35420189	0.14724024	-2.4056052	0.0161457	0.045095655	ZNF555
8.71065795	1.990477025	0.76255054	2.6102886	0.0090466	0.0272841	TLE6
35.39095221	-0.89026822	0.29095437	-3.0598208	0.0022147	0.007878678	TLE2
1968.91525	0.174026779	0.05299925	3.2835707	0.001025	0.003944438	GNA11
943.8371631	0.337192155	0.07286299	4.6277561	3.70E-06	2.30E-05	NCLN
1622.621395	-0.44413596	0.05634647	-7.8822322	3.22E-15	6.05E-14	NFIC
1133.564036	0.365775893	0.066783	5.4770811	4.32E-08	3.65E-07	MFSD12
1149.100257	-0.20209002	0.06639394	-3.0438021	0.0023361	0.008248644	HMG20B

785.982127	0.243461563	0.06951227	3.5024258	0.000461	0.001928217	PIP5K1C
40.07748215	1.117549037	0.3578817	3.1226772	0.0017921	0.006526774	ATCAY
858.2085122	0.520835727	0.07438463	7.0019265	2.52E-12	3.50E-11	DAPK3
30324.66397	-0.30035246	0.03238996	-9.2730112	1.81E-20	5.56E-19	EEF2
42.51382203	1.190249279	0.29631705	4.01681	5.90E-05	0.000294364	EBI3
1728.79244	-0.21062782	0.05045821	-4.1743025	2.99E-05	0.000157607	CHAF1A
510.3127141	-0.30247476	0.08013241	-3.7746867	0.0001602	0.000736461	UBXN6
1402.138171	-0.20672331	0.05940558	-3.4798634	0.0005017	0.002080618	HDGFL2
13.88343621	2.152445786	0.6669831	3.2271369	0.0012504	0.004711526	PLIN4
55.95798663	-1.05437886	0.22945234	-4.5951977	4.32E-06	2.66E-05	SEMA6B
256.5408579	-0.61604804	0.11744623	-5.2453625	1.56E-07	1.21E-06	TNFAIP8L1
265.922717	0.309078128	0.11206892	2.7579291	0.0058169	0.018536848	TICAM1
1738.675154	-0.14574108	0.05085014	-2.8660898	0.0041558	0.013741913	PLIN3
1301.448434	-0.49378888	0.05972428	-8.2678077	1.36E-16	2.93E-15	UHRF1
858.4218779	-0.18573914	0.06615747	-2.8075308	0.0049923	0.016175878	KDM4B
1711.028898	-0.19706338	0.04911141	-4.0125785	6.01E-05	0.00029892	SAFB
2451.903293	-0.1599603	0.04962506	-3.2233777	0.0012669	0.004770703	RPL36
1263.578618	-0.49015653	0.05774826	-8.4878141	2.11E-17	4.90E-16	LONP1
910.501397	-0.22987695	0.06366125	-3.61094	0.0003051	0.001320433	RANBP3
1132.404964	-0.24078716	0.05949037	-4.0474981	5.18E-05	0.000260714	MLLT1
5194.548909	-0.29580243	0.03843236	-7.6967019	1.40E-14	2.49E-13	KHSRP
773.0723098	-0.39620461	0.06663387	-5.9459946	2.75E-09	2.72E-08	SLC25A23
239.8780786	0.544989646	0.11698187	4.6587532	3.18E-06	2.01E-05	TUBB4A
91.80714706	0.625633092	0.18521132	3.377942	0.0007303	0.002908176	CD70
118.9346175	2.866270694	0.21397654	13.395257	6.45E-41	6.67E-39	C3
1287.188799	-0.28607189	0.05807384	-4.9260023	8.39E-07	5.80E-06	TRIP10
313.3417187	-0.76225224	0.10877268	-7.0077546	2.42E-12	3.37E-11	INSR
210.1355997	0.536146737	0.13029067	4.1150049	3.87E-05	0.000199968	MCOLN1
630.2139794	-0.21446514	0.07431514	-2.8858874	0.0039031	0.013017877	XAB2
28.44084937	-1.18025634	0.35149598	-3.357809	0.0007856	0.003107642	PRR36
556.8433163	-0.18679654	0.07903519	-2.3634604	0.0181052	0.049723433	MAP2K7
2386.451151	-0.18207529	0.05124721	-3.5528818	0.000381	0.001618912	ELAVL1
91.10361745	3.613766549	0.34839187	10.372706	3.30E-25	1.51E-23	ANGPTL4
683.4509831	0.234433439	0.07796439	3.0069298	0.002639	0.009191333	RAB11B
151.7075685	0.627652848	0.15463453	4.0589436	4.93E-05	0.000249707	MARCHF2
5387.929661	-0.32426283	0.0387972	-8.357893	6.38E-17	1.42E-15	HNRNPM
144.4732843	0.771203384	0.15294494	5.0423597	4.60E-07	3.32E-06	ADAMTS10
342.6095723	-0.33493181	0.10142871	-3.30214	0.0009595	0.003725325	ZNF558
553.6481262	-0.42453824	0.0764676	-5.551871	2.83E-08	2.46E-07	ZNF699
240.588666	-0.29176262	0.11680722	-2.4978133	0.0124962	0.03623023	ZNF559
6.710411376	2.185083887	0.80966679	2.6987446	0.0069602	0.021749707	COL5A3
248.8481988	0.881420756	0.12084958	7.2935361	3.02E-13	4.65E-12	SHFL
4845.625678	-0.16296408	0.03730575	-4.3683361	1.25E-05	7.11E-05	DNMT1
366.9721473	-0.27367758	0.09325141	-2.9348357	0.0033372	0.011321626	RAVER1
2857.525246	0.189327686	0.04658039	4.0645362	4.81E-05	0.000244436	CDC37
217.5970595	-0.33552018	0.1221412	-2.746986	0.0060146	0.01911778	PDE4A
748.7545639	-0.27381345	0.07281097	-3.7606068	0.0001695	0.000776511	KRI1
211.1309663	-0.43186812	0.12131937	-3.5597624	0.0003712	0.001582109	ILF3-DT

7427.119297	-0.37365004	0.03524728	-10.60082	2.95E-26	1.44E-24	ILF3
164.7505164	-0.3387166	0.13817918	-2.4512854	0.0142347	0.040540693	QTRT1
6.807570144	2.258018294	0.87589077	2.5779679	0.0099383	0.029685235	C19orf38
737.1240054	-0.58570755	0.06762612	-8.6609666	4.68E-18	1.15E-16	CARM1
2662.300023	-0.26496152	0.0446718	-5.9312924	3.01E-09	2.96E-08	SMARCA4
1646.596793	-0.72737322	0.07227185	-10.064405	7.94E-24	3.21E-22	LDLR
539.9114777	-0.5449249	0.0866737	-6.2870848	3.23E-10	3.62E-09	SPC24
2091.880685	-0.20163171	0.05431088	-3.7125474	0.0002052	0.000925106	KANK2
92.22347064	1.081126245	0.19562015	5.5266608	3.26E-08	2.81E-07	RAB3D
567.0589907	0.326020167	0.07959739	4.095865	4.21E-05	0.00021582	PLPPR2
240.6762254	-0.4780132	0.1170158	-4.0850313	4.41E-05	0.000225476	ECSIT
883.9306869	0.25901536	0.07158051	3.6185181	0.0002963	0.001285755	CNN1
99.74656195	-0.51145995	0.17105273	-2.9900719	0.0027891	0.009665757	ZNF69
67.86413065	0.515546165	0.2155501	2.3917695	0.0167674	0.046585707	ZNF442
722.7102593	-0.26836881	0.07016862	-3.8246271	0.000131	0.0006111	ZNF791
666.0794454	0.287796093	0.07794743	3.6921817	0.0002223	0.000994206	MAN2B1
22.54373516	-1.19964933	0.37571864	-3.192946	0.0014083	0.005244156	RNASEH2A
21961.06453	0.165073795	0.0385232	4.2850486	1.83E-05	0.000100195	CALR
1119.477558	-0.42300903	0.06789801	-6.2300655	4.66E-10	5.12E-09	RAD23A
1136.203507	-0.37314324	0.0629313	-5.9293743	3.04E-09	2.99E-08	NFIX
1395.952118	-0.26409215	0.05754192	-4.589561	4.44E-06	2.73E-05	NACC1
493.9077837	-0.57050129	0.10982655	-5.1945664	2.05E-07	1.57E-06	IER2
151.2554444	0.672395417	0.16505376	4.0737964	4.63E-05	0.000235669	MIR23AHG
533.7291937	-0.21036084	0.07902343	-2.6620061	0.0077676	0.023869835	CC2D1A
176.116487	-0.67086402	0.14570131	-4.6043788	4.14E-06	2.56E-05	RFX1
107.3100417	-0.41161652	0.16548885	-2.4872764	0.0128725	0.037172592	IL27RA
599.5351937	-0.45404305	0.07644785	-5.9392519	2.86E-09	2.83E-08	SAMD1
179.7624382	-0.50462851	0.13334487	-3.7843863	0.0001541	0.000710015	ADGRL1
926.0038607	0.185193228	0.06690847	2.7678593	0.0056426	0.018050861	AKAP8L
15268.76891	0.122751335	0.03522689	3.4845919	0.0004929	0.002049085	TPM4
1769.704529	-0.18953784	0.05093922	-3.7208628	0.0001985	0.000897963	RAB8A
711.8863815	-0.19866231	0.07608915	-2.6109151	0.00903	0.027243622	EPS15L1
980.8550818	-0.17333269	0.06266313	-2.7661033	0.0056731	0.018145013	CHERP
138.5524959	0.431505804	0.15211865	2.8366397	0.0045591	0.014916803	MED26
63.98899735	0.743599928	0.2297307	3.236833	0.0012086	0.004571176	TMEM38A
706.2027867	0.212445643	0.07665525	2.7714428	0.0055808	0.01786652	DDA1
159.1631128	3.714702734	0.46562197	7.977937	1.49E-15	2.93E-14	BST2
27.03596472	1.970779543	0.41272139	4.7750846	1.80E-06	1.18E-05	BISPR
2462.688838	0.243209706	0.05126905	4.7437918	2.10E-06	1.36E-05	COLGALT1
16.53671582	1.873483648	0.48684504	3.8482135	0.000119	0.000558915	UNC13A
953.969752	-0.33953776	0.06446991	-5.2666079	1.39E-07	1.09E-06	RPL18A
173.3433314	0.332026673	0.13646928	2.4329774	0.0149752	0.042303336	ARRDC2
193.0036832	0.431805483	0.13097588	3.296832	0.0009778	0.003787989	MPV17L2
16.80281743	2.017536748	0.63036314	3.2005944	0.0013714	0.005117901	IQCN
85.71064595	1.867466349	0.21924193	8.517834	1.63E-17	3.83E-16	GDF15
188.1571873	-0.33967626	0.13126537	-2.5877065	0.0096617	0.028953545	SSBP4
194.248185	0.381912111	0.13848162	2.7578542	0.0058182	0.018537693	ISYNA1
9.697271964	4.138256232	1.28896237	3.210533	0.0013249	0.004962276	COMP

1961.133107	0.160372643	0.048338	3.3177347	0.0009075	0.003539255	UPF1
121.2470753	0.456903091	0.17185242	2.6586945	0.0078444	0.024075929	BORCS8
217.3191248	0.304659753	0.12543453	2.4288349	0.0151474	0.04270652	RFXANK
433.2229767	0.227814474	0.08590283	2.6520019	0.0080016	0.024493572	SUGP1
104.7532734	0.671157643	0.19855285	3.3802468	0.0007242	0.002887863	YJEFN3
448.1488275	0.253962791	0.08589277	2.9567424	0.0031091	0.010636802	ATP13A1
308.5482316	-0.32681949	0.1030037	-3.1728908	0.0015093	0.005579635	ZNF85
638.5186342	-0.25986935	0.07489318	-3.4698665	0.0005207	0.00215243	ZNF430
2090.679298	-0.73214079	0.05058251	-14.474187	1.76E-47	2.38E-45	ZNF714
969.3802126	-0.47585551	0.06694718	-7.1079243	1.18E-12	1.70E-11	ZNF431
253.8552453	-0.38857996	0.11340271	-3.4265492	0.0006113	0.002485518	ZNF708
127.9189847	-0.75691286	0.16065815	-4.7113258	2.46E-06	1.58E-05	ZNF429
337.088514	-0.39122995	0.09695074	-4.0353478	5.45E-05	0.000273157	ZNF100
102.524826	-0.55372129	0.17993645	-3.0773158	0.0020887	0.007473904	ZNF43
29.05899825	-0.88101348	0.32428797	-2.7167627	0.0065924	0.020727097	ENSG00000267886
76.2061929	-0.76866829	0.20639228	-3.7243073	0.0001959	0.000886484	ZNF730
171.8955917	0.750861209	0.14016743	5.3568877	8.47E-08	6.85E-07	LINC01224
116.7256809	0.423893081	0.16800089	2.5231597	0.0116306	0.034066645	ENSG00000268362
264.7367886	-0.62270496	0.1097604	-5.6733115	1.40E-08	1.27E-07	ZNF254
221.835636	-0.30047257	0.12172464	-2.4684613	0.0135695	0.038901111	LINC00662
295.8598695	0.761751508	0.11000623	6.9246217	4.37E-12	5.96E-11	CCNE1
1277.471118	-0.23303462	0.05996382	-3.8862535	0.0001018	0.000485428	URI1
236.7766401	-0.58100603	0.11280365	-5.1505958	2.60E-07	1.95E-06	TSHZ3
971.8541772	-0.45747188	0.06440974	-7.1025262	1.22E-12	1.77E-11	ZNF507
734.5946246	-0.27468533	0.06979626	-3.935531	8.30E-05	0.00040234	DPY19L3
1026.286724	-0.38753226	0.0602842	-6.4284222	1.29E-10	1.51E-09	ANKRD27
27.42136046	1.751058903	0.41986523	4.1705261	3.04E-05	0.000160048	NUDT19-DT
609.155251	-0.19948672	0.07562314	-2.6379058	0.008342	0.025436955	CEP89
747.9072567	0.321732433	0.07089074	4.5384267	5.67E-06	3.40E-05	RHPN2
7.201671037	2.13201686	0.77208171	2.7613876	0.0057556	0.018375334	ENSG00000289378
1186.15004	-0.27029832	0.0614911	-4.3957307	1.10E-05	6.34E-05	CEBPG
329.2183457	-0.25420874	0.09913609	-2.5642402	0.0103402	0.030722022	KCTD15
3068.849137	-0.26076414	0.04343184	-6.0039863	1.93E-09	1.96E-08	LSM14A
4201.497993	-0.48567977	0.04274686	-11.361766	6.48E-30	4.01E-28	GPI
3782.496124	-0.28923632	0.04081457	-7.0865945	1.37E-12	1.96E-11	UBA2
90.61458944	0.493525765	0.19857719	2.4853094	0.0129439	0.037341442	ZNF30
531.0707967	-0.30945017	0.07699419	-4.0191365	5.84E-05	0.000291556	USF2
85.78189266	0.827640546	0.20356714	4.0656883	4.79E-05	0.000243444	DMKN
670.1641341	-0.22452892	0.07359421	-3.0509047	0.0022815	0.00808061	KMT2B
628.4724125	0.950235491	0.0854233	11.123844	9.60E-29	5.63E-27	APLP1
2564.44056	-0.31578731	0.04524537	-6.979439	2.96E-12	4.07E-11	ZNF146
218.1490533	-0.33775658	0.11830318	-2.8550084	0.0043036	0.014160658	LINC00665
95.37012412	-0.7810974	0.17747851	-4.4010815	1.08E-05	6.19E-05	ZFP14
242.669611	-0.46101825	0.11344222	-4.0639036	4.83E-05	0.000245028	ZFP82
273.2163739	-0.29634227	0.10812613	-2.740709	0.0061307	0.019434415	ZNF566
878.0616396	-0.25126575	0.06432377	-3.906266	9.37E-05	0.000449785	ZNF260
200.6811408	-0.33942047	0.13006593	-2.6096032	0.0090647	0.027329348	ZNF382
303.2826754	-0.27281677	0.10462703	-2.6075171	0.0091202	0.027477377	ZNF829

177.9930899	-0.3938575	0.12910978	-3.0505629	0.0022841	0.008088167	ZNF568
221.2868068	-0.38015359	0.12161791	-3.1258026	0.0017732	0.006464987	ZNF420
151.0497546	-0.37674264	0.14315622	-2.631689	0.0084962	0.025839145	ZNF383
301.3929483	-0.2673516	0.1017684	-2.627059	0.0086126	0.026129413	ZNF569
108.2460892	-0.51024294	0.16478158	-3.0964804	0.0019583	0.007054516	ZNF607
19.58130008	-1.03477802	0.38658006	-2.6767496	0.007434	0.022986762	ENSG00000267152
60.90123151	-0.87905798	0.22648111	-3.8813744	0.0001039	0.000494485	ZNF573
252.1739758	0.293478865	0.11418719	2.5701557	0.0101653	0.030254022	YIF1B
288.9644553	1.110657712	0.1142711	9.7194977	2.49E-22	8.95E-21	KCNK6
18.5526153	1.441006247	0.4660526	3.0919391	0.0019885	0.007153631	CATSPERG
65.47135475	-0.50631234	0.21373899	-2.3688347	0.0178442	0.049045537	FAM98C
9951.338971	0.210348755	0.03510662	5.9917115	2.08E-09	2.10E-08	ACTN4
190.8450701	0.37535149	0.13212203	2.8409455	0.004498	0.014747399	ECH1
2916.519796	-0.26423525	0.04698453	-5.6238775	1.87E-08	1.66E-07	HNRNPL
22.07025283	1.493822815	0.39468841	3.7848155	0.0001538	0.000708979	RINL
413.5581579	0.441697734	0.09280804	4.7592613	1.94E-06	1.27E-05	SIRT2
538.7316447	-0.22542591	0.08345064	-2.7013083	0.0069067	0.021602157	PAK4
1127.32594	-0.15502459	0.05801262	-2.6722562	0.0075343	0.023270737	SAMD4B
1332.905978	-0.18254639	0.06347619	-2.8758244	0.0040297	0.013381161	PAF1
955.3743373	0.212630777	0.06476188	3.2832708	0.0010261	0.003946017	MED29
3959.432583	-0.3417103	0.0457885	-7.4627976	8.47E-14	1.39E-12	RPS16
2252.963895	0.178251278	0.0457129	3.8993649	9.64E-05	0.000462034	SUPT5H
649.8685319	-0.1904745	0.08031797	-2.3715054	0.0177158	0.048754159	TIMM50
1543.27762	-0.57596782	0.05295044	-10.877489	1.48E-27	7.95E-26	FBL
1276.364763	-0.17019921	0.05959806	-2.8557845	0.0042931	0.014134113	PSMC4
1057.973297	-0.20330303	0.05976602	-3.4016491	0.0006698	0.002694408	AKT2
1218.286427	0.402209181	0.06591782	6.1016757	1.05E-09	1.10E-08	PLD3
68.32838858	-0.7600332	0.20863199	-3.6429369	0.0002695	0.001182191	PRX
206.1996509	0.383413957	0.13313933	2.8797948	0.0039793	0.01323656	SERTAD1
475.5957648	-0.2921689	0.09377994	-3.1154734	0.0018365	0.006668766	LTBP4
989.3030999	-0.44502005	0.06353089	-7.0047828	2.47E-12	3.43E-11	SNRPA
13160.17577	-0.54934331	0.03608596	-15.223187	2.48E-52	3.92E-50	AXL
5353.45557	-0.39335035	0.03492664	-11.262187	2.02E-29	1.22E-27	HNRNPUL1
33.76127366	-0.94224054	0.29337199	-3.2117604	0.0013192	0.004942189	ENSG00000286177
3775.025495	-0.15169537	0.04325346	-3.5071271	0.000453	0.001896292	RPS19
63.4899724	0.853335239	0.23016844	3.7074381	0.0002094	0.000942258	POU2F2
881.9335803	-0.19697013	0.07031842	-2.801117	0.0050926	0.016467162	CIC
350.4779773	0.370032953	0.1040269	3.5570892	0.000375	0.001596333	MEGF8
6.711189973	2.939226512	1.03484851	2.8402481	0.0045078	0.014768546	CNFN
64.11527824	3.12190072	0.30578166	10.209575	1.80E-24	7.64E-23	CEACAM1
23.96814393	1.515896795	0.3809621	3.9791276	6.92E-05	0.000340942	PSG3
202.7218762	-0.9173506	0.1495864	-6.1325803	8.65E-10	9.15E-09	PSG1
227.1145979	1.015970984	0.12316566	8.2488171	1.60E-16	3.42E-15	PSG2
486.3058163	0.234068523	0.09100586	2.5720158	0.0101108	0.03013321	PSG5
131.3627798	0.489067803	0.15785854	3.0981397	0.0019474	0.007025978	ETHE1
326.3693343	-0.5269742	0.09984278	-5.2780403	1.31E-07	1.03E-06	XRCC1
480.5814318	0.462697534	0.09045111	5.1154437	3.13E-07	2.32E-06	IRGQ
248.4455216	0.315698443	0.12609435	2.5036684	0.0122913	0.035731532	ZNF428

271.6064214	-0.32175696	0.11503096	-2.7971336	0.0051558	0.016649901	ZNF404
72.03970125	-0.60790591	0.20572006	-2.9550152	0.0031265	0.01068392	ZNF221
248.5431698	-0.40488442	0.1121348	-3.6106937	0.0003054	0.00132103	ZNF227
132.0202213	-0.36216194	0.15160894	-2.3887902	0.016904	0.046876051	ZNF229
2858.720114	0.55054712	0.0518919	10.6095	2.69E-26	1.32E-24	PVR
37.42694511	0.850054783	0.32561862	2.6105841	0.0090388	0.027265274	CEACAM19
48.62567385	2.337818673	0.34815228	6.7149313	1.88E-11	2.37E-10	APOE
1765.030088	0.153947814	0.04910275	3.1352179	0.0017173	0.006285675	CLPTM1
81.37364439	1.104003567	0.20966794	5.2654858	1.40E-07	1.09E-06	RELB
25.9083476	1.218701455	0.35068636	3.4751892	0.0005105	0.002113685	ZNF296
36.85245397	1.323996537	0.32923067	4.0214861	5.78E-05	0.000288746	TRAPPC6A
543.8366332	-0.47223596	0.08558775	-5.5175651	3.44E-08	2.94E-07	PPP1R13L
451.4719822	0.304075463	0.09522196	3.1933333	0.0014064	0.005238249	POLR1G
64.90652706	-1.64522162	0.23865657	-6.8936784	5.44E-12	7.31E-11	FOSB
24.83856565	1.01949993	0.35562946	2.8667477	0.0041471	0.01371599	PPM1N
446.864184	-0.24791399	0.08522928	-2.9087891	0.0036283	0.012187831	OPA3
145.5563331	-0.36678664	0.15173299	-2.4173164	0.0156354	0.043868985	EML2
2009.12463	-0.18083254	0.0549949	-3.288169	0.0010084	0.003891462	SNRPD2
286.8601605	0.47194197	0.12168384	3.8784278	0.0001051	0.000499529	DMPK
5424.321392	-0.39016354	0.04168665	-9.3594359	8.02E-21	2.55E-19	CALM3
79.59611264	0.738960258	0.21834332	3.384396	0.0007134	0.002846526	DACT3
303.5898713	0.27471454	0.10470211	2.6237726	0.0086962	0.02635528	FKRP
1623.948478	0.144177663	0.05749265	2.5077584	0.01215	0.035362009	AP2S1
2756.944018	-0.48815447	0.0461242	-10.583479	3.56E-26	1.72E-24	ARHGAP35
183.4902699	1.019114055	0.14389653	7.0822697	1.42E-12	2.02E-11	NPAS1
4194.122974	-0.1913029	0.04155144	-4.6040023	4.14E-06	2.56E-05	SAE1
77.49214547	1.234806733	0.22531967	5.4802438	4.25E-08	3.59E-07	BBC3
252.3354494	0.397733597	0.11509849	3.4555936	0.0005491	0.002256273	MEIS3
527.6791157	0.233146874	0.08273513	2.8179914	0.0048325	0.015713791	NAPA
1108.741746	-0.27116064	0.06137268	-4.4182629	9.95E-06	5.76E-05	NOP53
478.4055335	0.336743672	0.08759579	3.8442904	0.0001209	0.000567471	SELENOW
42.93626343	1.980216696	0.34503202	5.7392258	9.51E-09	8.80E-08	PLA2G4C
339.3511682	-0.27379624	0.09709156	-2.8199797	0.0048027	0.015640154	CARD8
41.40695276	1.145499402	0.28647411	3.998614	6.37E-05	0.000315738	CARD8-AS1
2097.724696	-0.21684662	0.05197436	-4.1721841	3.02E-05	0.000158983	KDELR1
5015.690414	-0.33726667	0.04498707	-7.4969698	6.53E-14	1.08E-12	RPL18
22.85062329	-0.9551953	0.38993311	-2.4496389	0.0143	0.040706537	DBP
2154.837935	0.389764309	0.05067179	7.6919393	1.45E-14	2.57E-13	PPP1R15A
1531.283152	0.32404875	0.05624401	5.7614803	8.34E-09	7.78E-08	NUCB1
1566.046042	-0.25372118	0.05999429	-4.2290885	2.35E-05	0.000125821	RUVBL2
130.6566415	0.447351506	0.16962105	2.6373585	0.0083554	0.025464643	TRPM4
47.58344454	1.20357338	0.30882393	3.8972802	9.73E-05	0.0004659	SLC17A7
4696.406558	-0.40396662	0.04600665	-8.7806132	1.63E-18	4.18E-17	RPL13A
3446.150947	-0.25995398	0.04468531	-5.8174375	5.98E-09	5.66E-08	RPS11
1839.816834	-0.35361541	0.04686098	-7.5460531	4.49E-14	7.59E-13	PRMT1
486.9678185	0.363471833	0.08811866	4.1247996	3.71E-05	0.000192503	CPT1C
63.33705184	0.792429899	0.242782	3.2639566	0.0010987	0.00419683	PTOV1-AS2
408.7999227	0.402745627	0.09988446	4.0321149	5.53E-05	0.000276784	PNKP

212.7980001	-0.31047802	0.12197852	-2.5453499	0.0109168	0.03224247	TBC1D17
313.8014061	0.619339065	0.10263978	6.0341039	1.60E-09	1.64E-08	ATF5
456.9659492	-0.22690012	0.09025357	-2.5140294	0.011936	0.034838514	VRK3
13.06027443	1.880590167	0.53653238	3.5050823	0.0004565	0.001910456	ENSG00000287001
272.1857259	0.616772466	0.11028173	5.5926985	2.24E-08	1.96E-07	EMC10
302.248	-0.26711986	0.10131949	-2.6364115	0.0083788	0.02553135	ZNF175
3.871923394	4.075913319	1.4024459	2.9062892	0.0036574	0.012273786	FPR1
107.8194295	-0.51560462	0.17780943	-2.8997597	0.0037345	0.012505826	ZNF613
195.0312338	-0.55838596	0.12446903	-4.4861436	7.25E-06	4.28E-05	ZNF615
211.8678278	-0.32675476	0.12217119	-2.6745646	0.0074826	0.023124759	ZNF432
295.0834263	-0.51547515	0.11254346	-4.5802319	4.64E-06	2.84E-05	ZNF616
431.5344586	-0.36982473	0.09770186	-3.7852375	0.0001536	0.000708152	ZNF766
499.2622208	-0.19429679	0.08190056	-2.37235	0.0176753	0.048658243	ZNF480
187.7876278	-0.51342666	0.13287147	-3.864085	0.0001115	0.000526928	ZNF880
317.0378271	-0.45651133	0.11495703	-3.9711476	7.15E-05	0.000351571	ZNF528
711.2233011	-0.22072788	0.08087575	-2.7292223	0.0063484	0.020054093	ZNF83
55.68372598	0.845587558	0.2565673	3.295773	0.0009815	0.003800604	ZNF600
410.8567491	-0.34883625	0.09219711	-3.7835919	0.0001546	0.000712097	ZNF702P
1135.011794	-0.19808061	0.0607143	-3.2625032	0.0011043	0.004214239	ZNF160
746.9138738	-0.31788966	0.07414928	-4.287158	1.81E-05	9.93E-05	ZNF347
455.6046378	-0.22325938	0.08779392	-2.5429937	0.0109907	0.03244418	ZNF845
280.093777	-0.42363134	0.1042861	-4.0622035	4.86E-05	0.000246532	ZNF813
628.0648895	0.243713189	0.08533637	2.8559125	0.0042913	0.014131093	ZNF331
840.1470402	-0.25203416	0.06773193	-3.7210541	0.0001984	0.000897516	CNOT3
3121.419138	-0.26857831	0.04297268	-6.2499787	4.11E-10	4.55E-09	RPS9
1394.137353	0.453656426	0.12616228	3.5958168	0.0003234	0.001392644	LENG8
696.4720643	0.429024952	0.07176616	5.9780953	2.26E-09	2.27E-08	PPP1R12C
750.4563546	-0.29697885	0.07087978	-4.1898949	2.79E-05	0.000147965	TNNT1
701.4743329	0.198274711	0.07177104	2.7626007	0.0057343	0.018317277	IL11
4707.698849	-0.13494392	0.04698577	-2.8720169	0.0040786	0.013517638	RPL28
429.8603377	-0.34116622	0.09227481	-3.6972845	0.0002179	0.000977203	ZNF667
240.9168434	-0.39736758	0.12558681	-3.164087	0.0015557	0.00574388	ZNF471
279.5896783	-0.49209823	0.10745835	-4.5794323	4.66E-06	2.85E-05	ZFP28
328.7654974	-0.4055087	0.10185739	-3.9811416	6.86E-05	0.000338353	ZNF470
300.4650656	-0.4412392	0.1029719	-4.2850449	1.83E-05	0.000100195	ZNF548
257.555586	-0.40451944	0.11847568	-3.4143668	0.0006393	0.002584264	ZNF772
236.4321986	-0.38220625	0.11838292	-3.2285591	0.0012442	0.004694265	ZNF586
246.8093075	-0.34707219	0.11321539	-3.0655919	0.0021724	0.00774456	ZNF329
136.0706736	-0.49947461	0.14990865	-3.3318598	0.0008627	0.003384917	ZNF8
130.4933145	0.535199833	0.16013672	3.3421431	0.0008313	0.00327564	A1BG-AS1
3871.814968	-0.15000004	0.04252047	-3.5277138	0.0004192	0.001768384	RPS5
37.28824657	0.835567583	0.30267648	2.7605963	0.0057696	0.01840977	MZF1-AS1
803.7271863	-0.34442089	0.06530326	-5.2741761	1.33E-07	1.05E-06	SOX12
290.7120071	-0.51214457	0.10905324	-4.69628	2.65E-06	1.69E-05	TRIB3
1612.03873	0.195761498	0.05064121	3.8656559	0.0001108	0.00052369	TBC1D20
3126.651172	-0.28219265	0.04396901	-6.4179894	1.38E-10	1.61E-09	CSNK2A1
109.8484024	0.680935555	0.17915428	3.8008332	0.0001442	0.000667835	FAM110A
4196.010339	0.206821574	0.0481902	4.2917769	1.77E-05	9.74E-05	FKBP1A

1551.341193	-0.19198213	0.05514896	-3.4811558	0.0004993	0.002073572	NSFL1C
865.0073456	-0.23679009	0.06416499	-3.6903315	0.000224	0.000999923	STK35
4768.615208	0.200934946	0.04229177	4.7511593	2.02E-06	1.31E-05	NOP56
294.3313176	0.348313289	0.11207328	3.1079066	0.0018842	0.006823371	PCED1A
695.4261076	0.385304033	0.0717532	5.3698515	7.88E-08	6.40E-07	DDRGK1
66.11930358	0.845580509	0.23364573	3.6190711	0.0002957	0.001283788	SLC4A11
5.415994147	2.349823164	0.96920175	2.4244933	0.0153298	0.043129692	ENSG00000277287
3.769716536	3.399971268	1.42010053	2.3941765	0.0166577	0.04631883	SIGLEC1
3006.814662	-0.18654968	0.04692132	-3.9757981	7.01E-05	0.000345454	MAVS
1059.269782	0.180729896	0.05903026	3.0616482	0.0022012	0.007837676	PANK2
236.4929617	0.876542149	0.13061183	6.7110472	1.93E-11	2.44E-10	SMOX
4357.842693	0.211552883	0.04814139	4.3944078	1.11E-05	6.37E-05	PRNP
497.4057358	-0.34872972	0.08195016	-4.2553881	2.09E-05	0.000113196	RASSF2
1206.995971	-0.23023749	0.0604074	-3.8114122	0.0001382	0.000642477	CDS2
359.4751983	0.51291007	0.10707045	4.7903978	1.66E-06	1.10E-05	GPCPD1
867.1056334	-0.21463349	0.06599911	-3.2520662	0.0011457	0.004357745	MCM8
23.66535922	1.874119649	0.49446679	3.790183	0.0001505	0.000695121	BMP2
4439.88318	-0.83064633	0.05005969	-16.593119	7.82E-62	1.70E-59	PLCB4
144.229531	0.738183861	0.16025727	4.6062426	4.10E-06	2.54E-05	SNAP25
2115.293156	2.632764116	0.06770969	38.883122	0	0	JAG1
1046.721878	-0.51594823	0.0596308	-8.6523781	5.04E-18	1.24E-16	BTBD3
121.3265507	-0.7736143	0.15945764	-4.851535	1.23E-06	8.25E-06	SPTLC3
261.8201003	-0.47218163	0.10974104	-4.3026894	1.69E-05	9.32E-05	KIF16B
30.29732727	1.407869633	0.38207674	3.6847824	0.0002289	0.001019866	BFSP1
9865.494167	0.432931272	0.04193261	10.324454	5.46E-25	2.42E-23	DSTN
8367.565374	0.281944474	0.03811402	7.3973946	1.39E-13	2.22E-12	RRBP1
2873.533872	-0.15901844	0.04609976	-3.4494418	0.0005617	0.002302292	SNX5
226.7877974	-0.35653415	0.11667814	-3.0557064	0.0022453	0.007976977	DTD1
791.8858832	0.356102382	0.07633172	4.6651952	3.08E-06	1.95E-05	RIN2
1354.62733	-0.21469952	0.05909416	-3.6331769	0.00028	0.001221064	CRNKL1
448.0061124	-0.69449131	0.09151292	-7.5889974	3.22E-14	5.54E-13	RALGAPA2
222.3428046	-0.89088404	0.12050586	-7.3928689	1.44E-13	2.30E-12	KIZ
3428.759086	-0.20870511	0.04619938	-4.5174871	6.26E-06	3.72E-05	XRN2
28.44640924	1.249487418	0.38281742	3.2639251	0.0010988	0.00419683	THBD
349.4097528	0.367265462	0.10013643	3.6676508	0.0002448	0.001083728	NAPB
668.0190545	0.839460541	0.07662592	10.955308	6.27E-28	3.48E-26	CST3
2710.678031	0.350018554	0.04848607	7.2189502	5.24E-13	7.85E-12	APMAP
10.61539263	1.558508368	0.59735409	2.6090193	0.0090802	0.027371272	ENSG00000274414
3548.726066	0.637278597	0.04778627	13.336017	1.43E-40	1.46E-38	PYGB
1575.960884	0.506411034	0.05266873	9.6150234	6.91E-22	2.39E-20	ABHD12
1114.986488	-0.32566728	0.05979988	-5.4459524	5.15E-08	4.30E-07	GINS1
471.7155648	-0.36337149	0.08452072	-4.2992001	1.71E-05	9.45E-05	NANP
3278.133483	0.432722953	0.04951273	8.7396297	2.34E-18	5.93E-17	HM13
860.2221022	0.647951304	0.08251488	7.8525392	4.08E-15	7.62E-14	ID1
3139.553886	0.395219539	0.04866401	8.1213929	4.61E-16	9.53E-15	BCL2L1
19091.16794	-0.21080469	0.0338143	-6.2341871	4.54E-10	5.00E-09	TPX2
11.63436445	1.258019248	0.52640979	2.3898098	0.0168571	0.046768508	MYLK2
996.1716341	0.275264716	0.06400102	4.3009425	1.70E-05	9.38E-05	PDRG1

5.269057996	2.643959315	0.94662125	2.7930488	0.0052214	0.016830367	HCK
2983.505811	-0.14640365	0.04702441	-3.1133545	0.0018497	0.006712433	TM9SF4
233.1161773	0.43964983	0.11975814	3.6711478	0.0002415	0.001070641	TSPY26P
3449.785477	-0.17430867	0.04412761	-3.9501048	7.81E-05	0.000380946	KIF3B
6911.10053	-0.27131445	0.03800645	-7.1386419	9.43E-13	1.38E-11	ASXL1
639.9982858	-0.32347256	0.08175161	-3.9567729	7.60E-05	0.000371096	NOL4L
414.8535738	0.563172591	0.10008908	5.6267136	1.84E-08	1.64E-07	DNMT3B
11970.1821	0.355387362	0.03967438	8.9576045	3.32E-19	9.19E-18	MAPRE1
412.3336845	0.339275906	0.09034483	3.755344	0.0001731	0.00079176	NECAB3
3600.777373	0.182374853	0.04519335	4.0354358	5.45E-05	0.000273133	CHMP4B
100.5488008	0.695441868	0.19176196	3.6265892	0.0002872	0.001249807	RALY-AS1
4249.037642	0.143819677	0.04139042	3.4747097	0.0005114	0.002116459	RALY
5867.613199	-0.10872704	0.04440796	-2.4483681	0.0143505	0.040814455	EIF2S2
16.66159123	1.62808704	0.49744026	3.2729298	0.0010644	0.004077931	MAP1LC3A
2776.692408	-0.36615877	0.04780382	-7.6596139	1.86E-14	3.27E-13	NCOA6
3307.611598	0.793638027	0.04820718	16.463066	6.76E-61	1.43E-58	TP53INP2
570.1177541	0.367533016	0.08988656	4.088854	4.34E-05	0.000222054	GGT7
2487.985312	0.147343172	0.04438469	3.3196846	0.0009012	0.003516995	GSS
1171.728498	0.471070365	0.06293321	7.4852433	7.14E-14	1.18E-12	MMP24OS
749.1970338	0.273193721	0.08064249	3.3877144	0.0007048	0.002818132	MMP24
1128.766471	0.287580263	0.06046328	4.7562795	1.97E-06	1.29E-05	UQCC1
2634.139997	-0.41452457	0.06127396	-6.7651019	1.33E-11	1.71E-10	CEP250
538.1383477	-0.38795997	0.10570822	-3.6701022	0.0002425	0.001074208	FER1L4
3090.109968	-0.27090113	0.04489905	-6.0335598	1.60E-09	1.64E-08	RBM12
716.6674661	-0.2661663	0.07319617	-3.6363418	0.0002765	0.00120854	NFS1
669.077959	0.31480173	0.07621742	4.1303121	3.62E-05	0.000188113	ROMO1
3171.577487	-0.22173823	0.05630147	-3.9384089	8.20E-05	0.000397878	PHF20
1953.689585	0.230743806	0.05030559	4.5868419	4.50E-06	2.76E-05	EPB41L1
1137.666381	0.187300343	0.06346381	2.9512937	0.0031645	0.010800772	AAR2
19869.95569	1.006329471	0.03598881	27.962284	4.67E-172	1.63E-168	MYL9
587.5060366	-0.4031358	0.08005382	-5.0358097	4.76E-07	3.43E-06	TGIF2
1594.408937	-0.25182942	0.06378099	-3.9483463	7.87E-05	0.000383325	NDRG3
2543.791836	-0.21079282	0.05068355	-4.1589988	3.20E-05	0.000167985	DSN1
3312.514315	0.902434384	0.06095147	14.805784	1.34E-49	1.92E-47	SAMHD1
12290.75227	0.250314096	0.03860423	6.4841107	8.93E-11	1.06E-09	RPN2
910.0716155	0.184269481	0.07380119	2.4968362	0.0125307	0.036312041	SRC
1986.67556	0.467594044	0.05195925	8.9992456	2.27E-19	6.37E-18	BLCAP
4.535613676	3.00338558	1.23788585	2.4262217	0.0152569	0.042966522	VSTM2L
83.96835222	1.493811543	0.20645958	7.2353703	4.64E-13	7.02E-12	TGM2
11.22304845	1.667301279	0.57887448	2.8802467	0.0039736	0.013225192	ENSG00000224635
930.9572978	0.354289717	0.06642839	5.3334083	9.64E-08	7.75E-07	SNHG17
140.1541174	0.722306441	0.15986374	4.5182631	6.23E-06	3.71E-05	SNHG11
5349.523437	-0.22481977	0.04601208	-4.8861028	1.03E-06	7.00E-06	RALGAPB
15.93103378	2.027426406	0.57620128	3.518608	0.0004338	0.001823123	MAFB
8239.188491	0.161887227	0.04449086	3.6386622	0.0002741	0.001198961	TOP1
36.30021582	0.877262025	0.3040365	2.8853839	0.0039094	0.013036147	PLCG1-AS1
4858.12692	0.147393658	0.03789886	3.8891319	0.0001006	0.000480366	PLCG1
2919.539867	-0.24757145	0.04314657	-5.7379176	9.58E-09	8.86E-08	ZHX3

538.7378657	0.835230111	0.09124925	9.1532822	5.52E-20	1.63E-18	LPIN3
3775.121088	-0.19393817	0.04691295	-4.1340012	3.57E-05	0.000185394	CHD6
5859.868286	0.382149491	0.04154322	9.1988405	3.62E-20	1.08E-18	SRSF6
7651.331089	0.401741444	0.03990457	10.067555	7.69E-24	3.12E-22	MYBL2
709.2507142	0.501510251	0.07791287	6.4368092	1.22E-10	1.43E-09	JPH2
538.4834609	-0.24750505	0.08287025	-2.9866576	0.0028205	0.009760736	FITM2
3538.61704	0.119036493	0.04552187	2.6149298	0.0089246	0.026967586	TTPAL
7895.807978	0.13885024	0.03993076	3.4772756	0.0005065	0.002099804	SERINC3
1073.316691	0.383123691	0.14825731	2.5841807	0.0097611	0.02922605	PABPC1L
292.3872046	-0.62223353	0.10494096	-5.9293676	3.04E-09	2.99E-08	KCNS1
9450.739369	0.223829716	0.04214017	5.3115527	1.09E-07	8.66E-07	SDC4
2105.412635	0.149574191	0.04881856	3.0638794	0.0021849	0.007784242	PIGT
344.0438798	0.445915329	0.10413128	4.2822419	1.85E-05	0.000101306	SNX21
143.9118827	0.729814507	0.1488046	4.9045158	9.37E-07	6.41E-06	ZSWIM3
12.04021344	1.342697041	0.54900961	2.4456713	0.0144583	0.041071425	SPATA25
36.66734272	0.923944307	0.32735383	2.8224637	0.0047656	0.01553114	NEURL2
3539.908257	0.184871927	0.04622329	3.9995406	6.35E-05	0.000314794	CTSA
39.2686664	1.805103677	0.36353725	4.9653884	6.86E-07	4.80E-06	ENSG00000271984
1417.584719	1.143519976	0.06713753	17.0325	4.71E-65	1.11E-62	PLTP
531.7134692	0.212751562	0.08472317	2.511138	0.0120343	0.035072211	ZNF335
1001.637569	0.320159449	0.06979862	4.5869022	4.50E-06	2.76E-05	NCOA5
742.4613928	0.264503046	0.07743756	3.4156943	0.0006362	0.002572298	CD40
45.06297554	1.089498767	0.29441284	3.7005817	0.0002151	0.000965339	SLC13A3
630.2488068	0.485965695	0.08032953	6.0496516	1.45E-09	1.50E-08	TP53RK
3835.545093	0.484475887	0.04767191	10.162712	2.91E-24	1.21E-22	NCOA3
104.6167758	1.809006158	0.2221284	8.1439661	3.83E-16	7.95E-15	SULF2
1026.050234	0.296079089	0.06515077	4.5445216	5.51E-06	3.31E-05	PREX1
3884.00696	-0.13581187	0.04653957	-2.9182018	0.0035206	0.01186255	ARFGEF2
18363.75965	-0.22978915	0.04328168	-5.3091546	1.10E-07	8.76E-07	CSE1L
4928.24919	0.86531672	0.04793896	18.050387	7.83E-73	2.13E-70	ZNFX1
19.00625932	-1.19835894	0.43886823	-2.7305666	0.0063226	0.019979874	PTGIS
3506.925951	0.431987235	0.05076131	8.5101668	1.74E-17	4.08E-16	B4GALT5
2897.9814	0.180905736	0.05262199	3.4378354	0.0005864	0.0023954	RNF114
100.921488	0.502677236	0.18365239	2.7371124	0.0061981	0.019622275	SNAI1
829.9772775	0.30530003	0.06737402	4.5314206	5.86E-06	3.50E-05	PEDS1
45.34868024	0.8703811	0.29899296	2.9110422	0.0036023	0.012107305	LINC01270
10.46618304	1.812400451	0.61067494	2.9678645	0.0029988	0.010306033	LINC01271
5124.789277	0.224038254	0.04539388	4.9354281	8.00E-07	5.54E-06	PTPN1
197.0627107	-0.33533321	0.13194232	-2.5415137	0.0110374	0.032554223	PARD6B
279.1175281	0.386554935	0.11167871	3.4613126	0.0005375	0.002213583	BCAS4
4187.083674	-0.17890284	0.03760928	-4.7568803	1.97E-06	1.28E-05	ADNP
1152.713243	-0.48974678	0.06710448	-7.2982727	2.91E-13	4.51E-12	ATP9A
374.2147075	-0.469485	0.09382794	-5.0036801	5.62E-07	4.00E-06	ZFP64
1930.506816	-0.14208822	0.05250274	-2.7063007	0.0068037	0.021329916	ZNF217
81.55823252	1.396308632	0.22682506	6.1558834	7.47E-10	7.98E-09	CYP24A1
1411.606753	0.174063581	0.06046082	2.8789482	0.00399	0.013267069	FAM210B
7174.833065	-0.15218923	0.04524243	-3.3638609	0.0007686	0.003046729	AURKA
3590.449155	-0.13913675	0.04262213	-3.2644246	0.0010969	0.004191279	RTF2

2333.657026	0.473385993	0.05050822	9.3724538	7.09E-21	2.27E-19	TFAP2C
675.3833945	0.531248417	0.07271992	7.3054044	2.76E-13	4.28E-12	RBM38
209.9475414	0.670167461	0.14919957	4.4917521	7.06E-06	4.17E-05	PMEPA1
3136.584818	-0.31356999	0.04577557	-6.8501608	7.38E-12	9.72E-11	RAB22A
4816.255302	-0.12586811	0.0479444	-2.6252932	0.0086574	0.026251587	VAPB
2379.864289	-0.15651665	0.04547132	-3.4420963	0.0005772	0.002361868	NELFCD
1190.128164	0.665183116	0.06234521	10.669354	1.42E-26	7.14E-25	CTSZ
2761.679234	0.236689395	0.04599559	5.1459146	2.66E-07	2.00E-06	ATP5F1E
3285.113301	-0.15012224	0.04772157	-3.1457941	0.0016564	0.006080683	PRELID3B
45.2375346	0.821872962	0.27411282	2.9983018	0.0027149	0.00943106	SYCP2
361.0902192	-0.34132365	0.09364643	-3.6448121	0.0002676	0.001174493	CDH4
2619.29957	-0.14241839	0.04327456	-3.2910414	0.0009982	0.00385738	LSM14B
1150.809842	0.218905506	0.05982815	3.658905	0.0002533	0.001117687	SS18L1
885.7681585	0.164253908	0.06504737	2.525143	0.0115651	0.033892395	MTG2
1954.251159	0.214383587	0.04985172	4.3004251	1.70E-05	9.40E-05	ADRM1
303.8170272	0.926154545	0.12290879	7.5352994	4.87E-14	8.22E-13	LAMA5
3294.879613	-0.17700887	0.05726015	-3.0913101	0.0019928	0.007167321	RPS21
14.03575757	1.504968371	0.54895794	2.7415003	0.0061159	0.019400954	ENSG00000277496
1280.511735	0.37475511	0.06617082	5.6634498	1.48E-08	1.33E-07	OGFR
36.93996303	1.284878917	0.30701066	4.185128	2.85E-05	0.000150783	COL9A3
621.4686508	-0.29112029	0.07979174	-3.6485016	0.0002638	0.001160393	TCFL5
3276.909954	0.151872541	0.04622599	3.2854359	0.0010182	0.003923078	YTHDF1
2526.167422	0.350202869	0.04670656	7.497937	6.48E-14	1.08E-12	ARFGAP1
14.1329825	-1.77403811	0.50808885	-3.4915903	0.0004802	0.002002369	FNDC11
1298.995613	1.684459963	0.1566575	10.752501	5.77E-27	3.00E-25	HELZ2
95.17680676	0.817631263	0.19264299	4.2442826	2.19E-05	0.000118249	MHENCR
336.760319	0.797377633	0.09973189	7.9952126	1.29E-15	2.56E-14	STMN3
694.0014512	0.382920676	0.07485864	5.1152503	3.13E-07	2.32E-06	ARFRP1
147.7576378	-0.44148326	0.15137617	-2.9164646	0.0035402	0.011917261	ZBTB46
5865.617713	0.226340182	0.03775447	5.9950561	2.03E-09	2.06E-08	TPD52L2
13.60034098	1.223120006	0.51307049	2.383922	0.0171292	0.047372356	UCKL1-AS1
2062.330697	0.302961679	0.05676815	5.3368247	9.46E-08	7.62E-07	ZNF512B
363.4710683	0.519004954	0.10360649	5.0093863	5.46E-07	3.89E-06	TCEA2
1408.056614	-0.20443137	0.0542278	-3.7698632	0.0001633	0.000749652	PCMTD2
3822.250969	-0.17505146	0.04786088	-3.6575063	0.0002547	0.001122947	HSPA13
101.2806428	-0.60672487	0.17573154	-3.4525667	0.0005553	0.002278509	ENSG00000235609
2674.932517	-0.21095468	0.05221073	-4.0404469	5.33E-05	0.000267671	NRIP1
1464.370112	-0.28079245	0.06188876	-4.5370511	5.70E-06	3.42E-05	USP25
538.7346474	0.21567366	0.08728953	2.4707851	0.0134817	0.03866839	CXADR
99.17670047	0.493083112	0.17708762	2.7844019	0.0053627	0.017244185	MIR155HG
796.8990125	-0.3352632	0.07132099	-4.7007649	2.59E-06	1.66E-05	MRPL39
374.2702416	0.941919183	0.1009701	9.3286943	1.07E-20	3.35E-19	JAM2
1108.57858	-0.3602019	0.05920222	-6.0842637	1.17E-09	1.22E-08	GABPA
16742.20977	0.292413234	0.04027409	7.2605787	3.85E-13	5.88E-12	APP
6841.041275	-0.52409687	0.05160462	-10.156007	3.12E-24	1.29E-22	ADAMTS1
1079.913436	1.242338766	0.08043083	15.446052	8.02E-54	1.33E-51	ADAMTS5
1852.553621	-0.41275196	0.05642438	-7.3151352	2.57E-13	4.01E-12	LTN1
605.7397913	0.322030216	0.07519965	4.2823365	1.85E-05	0.000101295	RWDD2B

8997.553116	-0.24399667	0.03841337	-6.3518692	2.13E-10	2.44E-09	CCT8
2081.664433	-0.40249217	0.05108055	-7.8795578	3.29E-15	6.17E-14	MAP3K7CL
2383.75179	-0.47322664	0.04853603	-9.7500078	1.84E-22	6.71E-21	BACH1
665.0673917	-0.2813982	0.07900397	-3.5618235	0.0003683	0.001570507	MIS18A
214.282069	0.423941363	0.12792828	3.3138988	0.00092	0.003584951	IFNAR2
929.0739466	0.337505665	0.06369409	5.2988539	1.17E-07	9.23E-07	TMEM50B
3053.456874	-0.3258956	0.04651794	-7.0058045	2.46E-12	3.41E-11	GART
10695.56796	-0.27295657	0.04374911	-6.2391341	4.40E-10	4.86E-09	SON
493.4307455	-0.31334695	0.08482195	-3.6941729	0.0002206	0.000988227	CRYZL1
2019.073549	-0.18704376	0.04767555	-3.923264	8.74E-05	0.000421984	ITSN1
3832.942149	-0.29670619	0.0601449	-4.9331896	8.09E-07	5.60E-06	SLC5A3
900.5063872	0.388524535	0.06766744	5.7416766	9.37E-09	8.69E-08	RCAN1
1429.935768	-0.5069837	0.05418537	-9.3564685	8.24E-21	2.62E-19	DOP1B
1691.231558	-0.15241291	0.05172961	-2.9463379	0.0032156	0.010947425	MORC3
1326.899626	-0.22774317	0.0610464	-3.7306568	0.000191	0.000866014	CHAF1B
1572.736423	-0.22436779	0.05176572	-4.3342929	1.46E-05	8.17E-05	SIM2
446.5155389	-0.4157447	0.09105579	-4.5658241	4.98E-06	3.02E-05	HLCS
8454.364488	-0.24835038	0.03836432	-6.4734728	9.58E-11	1.14E-09	TTC3
958.7326121	-0.21746174	0.06457642	-3.36751	0.0007585	0.003013463	VPS26C
1430.719981	-0.29666559	0.06690164	-4.4343544	9.23E-06	5.37E-05	DYRK1A
43.61317553	-0.7684007	0.28016582	-2.7426639	0.0060943	0.019335873	KCNJ6
957.6011672	0.322638947	0.06435998	5.013037	5.36E-07	3.83E-06	ETS2
2099.481628	-0.25917905	0.06330842	-4.0939111	4.24E-05	0.00021752	BRWD1
3948.333537	-0.11058302	0.0389497	-2.8391236	0.0045238	0.014812322	HMGN1
472.0226578	-0.46893962	0.08648729	-5.4220641	5.89E-08	4.87E-07	GET1
551.4605631	-0.74008745	0.0892482	-8.2924637	1.11E-16	2.41E-15	BACE2
77.19741202	4.507336898	0.54977938	8.1984467	2.44E-16	5.14E-15	MX2
186.6421291	3.654985652	0.39138237	9.3386568	9.76E-21	3.06E-19	MX1
256.2871786	-0.28030444	0.11580117	-2.4205665	0.0154963	0.043527928	PRDM15
190.9107839	-0.32380128	0.12576423	-2.5746691	0.0100336	0.029918445	C2CD2
661.3912553	-0.27264312	0.07863203	-3.4673292	0.0005257	0.002169757	PKNOX1
2763.221769	0.419503606	0.0527032	7.959737	1.72E-15	3.36E-14	PDXK
805.8525749	0.219726622	0.07426873	2.9585348	0.0030911	0.010585547	CSTB
1321.233476	-0.22420142	0.05452869	-4.1116228	3.93E-05	0.00020266	AGPAT3
303.7841255	-0.30410559	0.10359832	-2.9354297	0.0033309	0.01130948	LRRC3
4.625151847	2.939270773	1.23316939	2.3835094	0.0171484	0.047417956	ENSG00000274225
2470.536513	0.249477793	0.05183982	4.8124738	1.49E-06	9.93E-06	UBE2G2
5520.400689	0.144171131	0.04100524	3.5159199	0.0004382	0.001839902	PTTG1IP
1246.249287	0.26751258	0.05605057	4.7727007	1.82E-06	1.19E-05	ADARB1
836.8770817	0.239315752	0.06807955	3.5152369	0.0004394	0.001843807	POFUT2
2464.902054	0.901543604	0.04866759	18.524516	1.31E-76	4.22E-74	COL18A1
2289.844978	0.976110755	0.05328615	18.318282	5.92E-75	1.74E-72	COL6A1
1971.25281	0.56332599	0.07002589	8.0445389	8.66E-16	1.75E-14	COL6A2
119.736587	-0.84539973	0.16164738	-5.2299005	1.70E-07	1.31E-06	MCM3AP-AS1
1218.053011	-0.20155824	0.05708666	-3.5307412	0.0004144	0.001751243	DIP2A
2148.730048	0.328828118	0.05440465	6.0441177	1.50E-09	1.55E-08	ATP6V1E1
696.8136818	0.264323328	0.08398718	3.1471867	0.0016485	0.006055631	BID
17.71320319	1.052780629	0.44104512	2.3870134	0.0169859	0.047058194	ENSG00000225335

66.88244901	2.929045636	0.27292427	10.732082	7.20E-27	3.71E-25	USP18
845.4811053	0.208744809	0.07001311	2.9815105	0.0028683	0.009906608	DGCR2
415.2905558	0.249913096	0.08895203	2.8095267	0.0049614	0.016093911	ESS2
1438.174869	0.349994	0.05649466	6.19517	5.82E-10	6.34E-09	UFD1
924.7703001	-0.22548751	0.06521735	-3.4574772	0.0005453	0.002242678	CDC45
566.1512914	0.207620315	0.08155407	2.5457996	0.0109028	0.032217383	RTL10
549.3095906	0.242019248	0.0803915	3.0105081	0.0026081	0.009094652	COMT
139.1319427	0.537530201	0.16205163	3.3170305	0.0009098	0.003547392	ARVCF
256.6305937	0.289244657	0.11336348	2.5514801	0.0107266	0.031760886	TANGO2
897.5418668	0.293904861	0.06966823	4.2186355	2.46E-05	0.000131312	DGCR8
188.5093559	0.504849866	0.12978733	3.8898239	0.0001003	0.00047913	DGCR6L
335.5512634	0.613979176	0.10043233	6.1133617	9.76E-10	1.03E-08	SCARF2
1564.424015	-0.17163026	0.05460172	-3.1433123	0.0016705	0.006126014	PI4KA
1334.145588	0.161341333	0.05653366	2.8538988	0.0043186	0.014204832	SNAP29
2692.422066	-0.19751443	0.04879515	-4.047829	5.17E-05	0.000260497	CRKL
14.89981431	1.296971868	0.52570962	2.4670879	0.0136217	0.039037771	P2RX6
166.8490533	0.391774764	0.13608121	2.8789776	0.0039897	0.013267069	YDJC
839.0422776	0.246439859	0.0677623	3.6368283	0.000276	0.001206919	BCR
1255.974302	-0.29483668	0.05537378	-5.3244817	1.01E-07	8.11E-07	SMARCB1
174.0856232	0.740848948	0.15109897	4.9030708	9.43E-07	6.45E-06	SLC2A11
9.380896723	1.511646816	0.61577795	2.454857	0.0140941	0.040225794	ENSG00000272787
43.44587675	3.338166009	0.43570712	7.6614906	1.84E-14	3.23E-13	SUSD2
775.0092729	0.168987481	0.06759796	2.4998901	0.0124232	0.036060621	SPECC1L
823.8997848	-0.19589451	0.06801881	-2.8800049	0.0039767	0.01323253	SNRPD3
30.42807949	1.180620067	0.34492873	3.4227942	0.0006198	0.002515988	LRP5L
3.368035356	4.146187442	1.40837345	2.9439546	0.0032405	0.011025606	MYO18B
662.0431694	-0.18824403	0.07533671	-2.4987026	0.0124649	0.036157533	HPS4
75.30352388	-0.51211578	0.20101053	-2.5477063	0.0108434	0.032068668	TFIP11-DT
503.6142289	-0.47798286	0.08857296	-5.3964874	6.80E-08	5.56E-07	TTC28
1373.792126	-0.24462076	0.05360524	-4.5633738	5.03E-06	3.05E-05	CCDC117
1724.337985	0.129896899	0.05139567	2.52739	0.0114914	0.033727452	XBP1
475.3231623	-0.3792792	0.09671863	-3.9214701	8.80E-05	0.000425021	KREMEN1
241.9670155	0.566440658	0.1150435	4.9237086	8.49E-07	5.86E-06	GAS2L1
1630.927102	0.19432216	0.05193778	3.7414413	0.000183	0.000833808	AP1B1
44.21608775	3.118121828	0.50074764	6.2269327	4.76E-10	5.21E-09	NEFH
1048.230503	-0.16106595	0.06605014	-2.4385405	0.0147467	0.041766319	NIPSNAP1
460.8320744	0.278473431	0.0921979	3.0203881	0.0025245	0.00884036	LIF
2973.254652	-0.10350071	0.04350608	-2.3789943	0.0173599	0.047934178	SF3A1
166.0772222	0.360279225	0.14583945	2.4703825	0.0134969	0.038705549	RNF215
33.59599962	1.473304801	0.36757849	4.0081366	6.12E-05	0.000304336	TCN2
117.2632925	0.406369784	0.1619558	2.5091401	0.0121025	0.035231122	SLC35E4
110.8313786	-0.46942546	0.16629676	-2.8228178	0.0047604	0.015516906	DUSP18
273.7804349	0.429099254	0.10906571	3.9343187	8.34E-05	0.000404263	OSBP2
1370.121867	-0.22382799	0.05343518	-4.1887756	2.80E-05	0.000148561	MORC2
392.7926626	0.88497361	0.09944519	8.8991095	5.63E-19	1.52E-17	SELENOM
67.99153147	0.515861929	0.21426122	2.4076309	0.0160564	0.044876386	INPP5J
814.1959567	0.31613176	0.06980458	4.5288109	5.93E-06	3.54E-05	LIMK2
47.73086445	0.706203472	0.26266663	2.6885923	0.0071754	0.022294122	LINC01521

605.1350225	0.241910304	0.07770734	3.1130948	0.0018514	0.006715737	PISD
1197.898983	-0.42612644	0.07574368	-5.6259007	1.85E-08	1.64E-07	PRR14L
3639.569733	0.189147242	0.04300764	4.397992	1.09E-05	6.28E-05	YWHAH
1126.518346	1.596190673	0.07398956	21.573189	3.21E-103	2.15E-100	TIMP3
1404.906032	-0.24536634	0.05361768	-4.5762208	4.73E-06	2.89E-05	HMGXB4
336.0118095	0.392546491	0.1004859	3.9064832	9.36E-05	0.000449506	TOM1
206.4419163	0.731342256	0.13905853	5.2592405	1.45E-07	1.13E-06	HMOX1
1824.518217	-0.45584206	0.04760824	-9.5748565	1.02E-21	3.43E-20	MCM5
87.30225864	1.030531836	0.22008957	4.6823292	2.84E-06	1.80E-05	RASD2
163.9506711	1.522942015	0.1495911	10.1807	2.42E-24	1.01E-22	APOL6
17.96141995	2.850071576	0.52509353	5.4277408	5.71E-08	4.73E-07	APOL3
1785.79088	0.233945779	0.05503003	4.2512386	2.13E-05	0.000114884	APOL2
167.9662478	1.832719674	0.15604838	11.74456	7.53E-32	5.24E-30	APOL1
66230.94433	0.172805385	0.04153631	4.1603453	3.18E-05	0.000167049	MYH9
1252.551412	0.421326816	0.05741656	7.3380709	2.17E-13	3.41E-12	FOXRED2
3512.206257	-0.15938393	0.04453852	-3.5785639	0.0003455	0.001481634	EIF3D
191.6745136	1.554134692	0.1415879	10.976465	4.96E-28	2.81E-26	CSF2RB
124.383079	0.724139626	0.17925887	4.0396306	5.35E-05	0.000268527	TST
9.334804081	1.720543728	0.68674637	2.5053554	0.0122328	0.035579376	IL2RB
133.8947555	0.538397987	0.15758367	3.416585	0.0006341	0.002566284	C1QTNF6
273.2192927	0.305265951	0.11027426	2.7682431	0.0056359	0.018032932	RAC2
10.81430917	1.902793096	0.57414051	3.3141593	0.0009192	0.003582416	ENSG00000237862
559.1375078	0.314686832	0.07892477	3.9871747	6.69E-05	0.000330429	CDC42EP1
6362.632194	0.221622696	0.03924578	5.6470458	1.63E-08	1.46E-07	LGALS1
4449.320292	-0.26000112	0.04569502	-5.6899218	1.27E-08	1.16E-07	H1-0
3538.798229	-0.41534056	0.04033004	-10.298541	7.15E-25	3.15E-23	EIF3L
1300.587216	0.450186299	0.0577983	7.7889192	6.76E-15	1.23E-13	MICALL1
311.3190931	0.488850239	0.11198606	4.3652777	1.27E-05	7.19E-05	ENSG00000278948
244.8193579	0.32003242	0.11753716	2.7228192	0.0064727	0.020395196	PICK1
8.970661023	1.976215474	0.77893237	2.5370822	0.0111781	0.032924564	ENSG00000272720
1625.578845	-0.16675118	0.05614463	-2.9700287	0.0029777	0.010243825	TOMM22
1590.294812	0.237643246	0.05561904	4.2726958	1.93E-05	0.000105478	JOSD1
1437.552216	0.163460316	0.05498643	2.9727392	0.0029516	0.010161846	SUN2
553.859954	1.495366693	0.10461718	14.2937	2.40E-46	3.00E-44	NPTXR
1066.660426	0.544689552	0.07127255	7.6423467	2.13E-14	3.72E-13	APOBEC3C
112.5096775	0.594473357	0.16873804	3.5230548	0.0004266	0.001796713	APOBEC3F
5.3290263	3.634299617	1.36336725	2.6656791	0.0076833	0.023644058	APOBEC3H
10557.787	-0.43995161	0.03544071	-12.413736	2.20E-35	1.82E-33	RPL3
322.7286943	-0.30674871	0.10651178	-2.8799511	0.0039774	0.01323253	TAB1
3659.461624	-0.14774639	0.04349284	-3.397028	0.0006812	0.002734629	ATF4
964.9906077	-0.30561484	0.07249602	-4.2156083	2.49E-05	0.000133004	TNRC6B
274.55031	0.423496281	0.11520788	3.6759316	0.000237	0.001052261	ADSL
846.2786301	0.19158916	0.0678833	2.8223312	0.0047676	0.015534649	MRTFA
2356.626816	-0.30235763	0.06423978	-4.7067036	2.52E-06	1.61E-05	EP300
156.3853864	-0.69776519	0.16924385	-4.1228392	3.74E-05	0.000193823	TEF
803.5221009	0.195191401	0.07407854	2.6349249	0.0084156	0.025634453	POLR3H
10044.31489	-0.25627898	0.03353064	-7.6431283	2.12E-14	3.70E-13	XRCC6
1829.369464	0.133020027	0.05389943	2.46793	0.0135897	0.038952478	SNU13

8.794341572	2.144594012	0.75523044	2.8396552	0.0045162	0.014790449	MEI1
1432.490315	-0.46935875	0.06209686	-7.5584937	4.08E-14	6.93E-13	SREBF2
23.16870812	1.016667255	0.42209147	2.408642	0.016012	0.044773855	SEPTIN3
1567.783171	-0.26059068	0.05490882	-4.7458799	2.08E-06	1.35E-05	TCF20
1249.821024	0.254656936	0.05584457	4.5601024	5.11E-06	3.10E-05	RRP7A
1639.184526	0.248381649	0.05201159	4.7755061	1.79E-06	1.18E-05	CYB5R3
79.71923999	0.694738509	0.21796978	3.1873157	0.001436	0.005334764	A4GALT
2742.440952	0.378374095	0.04549135	8.3174956	8.98E-17	1.97E-15	PACSIN2
90.44879787	0.683834171	0.21831185	3.1323731	0.001734	0.006337567	TTLL1
459.4531629	-0.25348995	0.08868757	-2.8582354	0.00426	0.014041346	TTLL12
231.1933838	0.330238703	0.12564164	2.6284177	0.0085783	0.026052534	PARVB
16.80698819	1.455390931	0.47216765	3.0823605	0.0020537	0.007363534	NUP50-DT
2481.234267	-0.18306297	0.04507375	-4.0614101	4.88E-05	0.000247299	NUP50
900.1920214	0.338571309	0.07358324	4.6012012	4.20E-06	2.59E-05	KIAA0930
390.3634732	0.281493283	0.09493529	2.965107	0.0030258	0.010392704	FAM118A
873.6630829	0.612227276	0.07564418	8.0935141	5.80E-16	1.19E-14	FBLN1
118.2895395	0.499010548	0.1682648	2.9656264	0.0030207	0.010377218	MIRLET7BHG
430.8331567	-0.69405929	0.0883176	-7.8586751	3.88E-15	7.27E-14	PPARA
1649.536396	-0.18135901	0.05151465	-3.5205326	0.0004307	0.001810818	CERK
390.4642792	-0.30262612	0.08892718	-3.403078	0.0006663	0.002682216	TBC1D22A
29.41607265	0.899769243	0.33404646	2.6935452	0.0070697	0.022016709	ENSG00000285722
811.5427624	-0.16632287	0.06919208	-2.4037848	0.0162263	0.045263996	ZBED4
530.378727	0.318614827	0.08753486	3.6398621	0.0002728	0.001193988	CRELD2
20.58862897	-1.2474119	0.37589334	-3.3185262	0.0009049	0.003530031	PANX2
651.6048813	0.290642478	0.0806709	3.6028167	0.0003148	0.001357678	TUBGCP6
38.38548254	0.760646806	0.28899367	2.6320535	0.008487	0.025823038	HDAC10
306.400874	0.320301516	0.11062004	2.8955107	0.0037854	0.012659324	MAPK12
114.3765894	0.462768379	0.17260799	2.6810368	0.0073394	0.022734761	MAPK11
741.738199	0.38963465	0.07120824	5.4717638	4.46E-08	3.75E-07	LMF2
58.0304213	0.745375774	0.23950631	3.1121342	0.0018574	0.006732011	CHKB-DT
82.25095304	1.472557817	0.21193668	6.9481026	3.70E-12	5.06E-11	MAPK8IP2
175.0338272	0.493924309	0.15414695	3.2042431	0.0013542	0.005059992	ARSA
78.9718945	0.517891434	0.19070219	2.7157079	0.0066134	0.020781973	SHANK3
226.7125748	-0.75509697	0.12773446	-5.9114584	3.39E-09	3.31E-08	GYG2
277.0898843	0.787394279	0.11994389	6.5646885	5.21E-11	6.35E-10	ARSD
595.80423	-0.35783314	0.08267579	-4.3281491	1.50E-05	8.38E-05	PRKX
216.4513798	0.582821672	0.1287231	4.5277161	5.96E-06	3.56E-05	STS
204.3799723	1.207767499	0.14590025	8.2780359	1.25E-16	2.71E-15	ANOS1
1292.781893	-0.78923552	0.05590546	-14.117324	2.97E-45	3.54E-43	TBL1X
2687.978539	0.149878992	0.04929937	3.0401806	0.0023644	0.008331392	MID1
72.98536853	0.627969378	0.23502634	2.6719107	0.0075421	0.023287755	ARHGAP6
1780.206307	-0.31883913	0.05695614	-5.5979761	2.17E-08	1.91E-07	PRPS2
2621.733084	0.262256036	0.05327229	4.9229352	8.53E-07	5.88E-06	TMSB4X
96.47752211	-0.47330871	0.17983662	-2.6318818	0.0084913	0.025829004	GEMIN8
27.68536249	1.008151636	0.35829928	2.8137138	0.0048973	0.015906559	ASB9
562.3839938	-0.25039103	0.08317905	-3.0102655	0.0026102	0.009100097	PIGA
17.75085164	-1.48384687	0.43254814	-3.4304779	0.0006025	0.002455544	PIR
206.8362655	-0.46354815	0.1338431	-3.4633698	0.0005335	0.002198288	CA5B

458.0899968	-0.31301493	0.08473458	-3.6940636	0.0002207	0.000988398	CTPS2
5280.432746	-0.27871647	0.03788373	-7.3571542	1.88E-13	2.98E-12	RBBP7
132.6368439	0.457837771	0.16761404	2.7315002	0.0063047	0.019930599	NHS
1199.446871	0.307814581	0.0627895	4.9023255	9.47E-07	6.48E-06	SCML1
487.5063174	-0.32150751	0.08458837	-3.8008476	0.0001442	0.000667835	PHKA2
1884.050191	-0.18337038	0.04843503	-3.7859037	0.0001532	0.000706444	PDHA1
1392.068428	0.171937424	0.06004136	2.8636495	0.0041879	0.013840321	SH3KBP1
1822.152195	-0.49373611	0.05357677	-9.2154879	3.10E-20	9.35E-19	RPS6KA3
208.9712551	1.06795359	0.14249874	7.4944776	6.66E-14	1.10E-12	CNKSR2
5471.374859	-0.23352759	0.04325707	-5.398599	6.72E-08	5.51E-07	SMS
1244.388572	0.139654412	0.05578455	2.5034603	0.0122985	0.035746575	PRDX4
1783.023166	0.135998143	0.04977943	2.7320147	0.0062948	0.019903117	ACOT9
1317.605179	0.836507873	0.06407165	13.055819	5.89E-39	5.66E-37	SAT1
5465.260072	-0.38504102	0.04288042	-8.979414	2.72E-19	7.56E-18	EIF2S3
460.8140597	-0.71034908	0.0955072	-7.4376497	1.02E-13	1.66E-12	PDK3
1817.779935	-0.81355115	0.05199766	-15.645918	3.54E-55	6.10E-53	POLA1
96.54571649	-0.65765967	0.1749487	-3.7591572	0.0001705	0.000780612	GK
715.9811496	0.495994273	0.07277268	6.8156659	9.38E-12	1.22E-10	DMD
2513.019946	0.467274799	0.04993873	9.3569623	8.21E-21	2.61E-19	TMEM47
906.4640374	0.227094958	0.06770029	3.3544161	0.0007953	0.003142633	DYNLT3
155.5529412	0.360335663	0.14905672	2.4174399	0.0156301	0.04386118	SRPX
96.21439665	-0.41950928	0.17254074	-2.4313636	0.0150421	0.042471555	RPGR
324.5741249	-0.50457321	0.0997812	-5.0567965	4.26E-07	3.10E-06	MID1IP1
929.5456014	-0.24795035	0.06513564	-3.8066768	0.0001408	0.000653431	BCOR
3879.1713	0.282835559	0.04427344	6.3883805	1.68E-10	1.95E-09	ATP6AP2
2705.707092	-0.44811471	0.04547655	-9.8537539	6.60E-23	2.50E-21	MED14
6652.784921	-0.3379709	0.05578144	-6.0588419	1.37E-09	1.42E-08	USP9X
10873.07253	-0.11374345	0.0387539	-2.9350194	0.0033353	0.011317133	DDX3X
13.38458833	1.889459539	0.5799474	3.2579843	0.0011221	0.004278644	PINCR
259.0803522	0.366459698	0.14808656	2.4746317	0.0133374	0.03832398	MIR222HG
16.46002086	1.088893391	0.45028271	2.4182438	0.0155956	0.043778511	LINC02595
1229.761336	0.211556607	0.06605786	3.2025954	0.001362	0.005086843	RP2
1098.101054	-0.35190183	0.05937686	-5.9265818	3.09E-09	3.04E-08	RBM10
1959.977314	-0.35045088	0.05232216	-6.6979436	2.11E-11	2.65E-10	CDK16
380.7499242	-0.22484024	0.09382012	-2.3965034	0.0165523	0.046092101	ZNF41
762.4079878	-0.34655018	0.06753078	-5.1317363	2.87E-07	2.14E-06	ELK1
3348.635227	0.246280352	0.05798057	4.2476359	2.16E-05	0.000116602	RBM3
363.2198658	-0.42699275	0.09703978	-4.4001827	1.08E-05	6.22E-05	SUV39H1
637.9743498	-0.24174661	0.07903968	-3.0585474	0.0022241	0.007907913	HDAC6
89.42110093	0.599750628	0.19511951	3.0737605	0.0021138	0.007557179	KCND1
110.3790812	0.560903622	0.17072547	3.2854127	0.0010183	0.003923078	PRAF2
139.2471952	0.374702506	0.15619644	2.3989183	0.0164436	0.045831774	WDR45
457.9843848	-0.25416526	0.08971723	-2.8329591	0.0046119	0.015075457	GPKOW
18.86438442	1.195270156	0.44132278	2.7083809	0.0067612	0.021204317	SYP
108.8088858	0.635212882	0.18251602	3.4803131	0.0005008	0.00207812	NUDT11
1126.616739	0.688311689	0.06553281	10.503314	8.34E-26	3.94E-24	TSPYL2
179.7089192	-0.74691647	0.14141574	-5.2817067	1.28E-07	1.01E-06	KANTR
1699.561155	-0.22026689	0.0498414	-4.4193558	9.90E-06	5.73E-05	KDM5C

96.84760622	0.904093354	0.19430818	4.6528838	3.27E-06	2.05E-05	IQSEC2
5993.511032	-0.35541992	0.04097644	-8.6737632	4.18E-18	1.04E-16	SMC1A
729.0802843	0.176248411	0.07051813	2.4993349	0.0124427	0.036111142	HSD17B10
8275.367402	-0.6512457	0.09619286	-6.7702081	1.29E-11	1.65E-10	HUWE1
212.0494261	-0.3849131	0.12190285	-3.1575399	0.0015911	0.00586448	FGD1
685.495448	0.196680006	0.07770654	2.5310611	0.0113718	0.033415967	APEX2
160.0111692	-0.57116427	0.13723511	-4.1619398	3.16E-05	0.000165986	FAM104B
1145.087481	-0.18573314	0.06086739	-3.0514395	0.0022775	0.008069518	UBQLN2
370.5268544	0.251135347	0.09554229	2.6285254	0.0085756	0.026048836	SPIN3
814.633213	-0.30654321	0.07208387	-4.2525912	2.11E-05	0.000114299	SPIN4
1322.343772	-0.19871175	0.05360348	-3.7070683	0.0002097	0.00094339	LAS1L
11336.4085	0.187336043	0.03803084	4.9258976	8.40E-07	5.80E-06	MSN
45.19640418	1.897222262	0.33098996	5.7319632	9.93E-09	9.15E-08	EDA2R
23.7471261	-1.0302101	0.3862793	-2.6670083	0.007653	0.023563259	AR
273.1932161	1.044486001	0.11707259	8.9216957	4.59E-19	1.26E-17	EFNB1
931.4708222	-0.2340304	0.06707331	-3.4891733	0.0004845	0.002019111	IGBP1
271.6742234	-0.5533496	0.11223236	-4.9303925	8.21E-07	5.68E-06	DLG3
93.62556161	-0.67956875	0.18855664	-3.6040563	0.0003133	0.001352268	FOXO4
657.5949609	-0.57540397	0.0795785	-7.2306462	4.81E-13	7.25E-12	MED12
1296.983437	-0.61233581	0.06100514	-10.037445	1.04E-23	4.16E-22	ZMYM3
10744.56397	-0.28216312	0.03415483	-8.2612947	1.44E-16	3.09E-15	NONO
1561.810269	-0.49836998	0.06426402	-7.7550385	8.83E-15	1.60E-13	TAF1
495.855682	0.800388669	0.08798408	9.0969714	9.29E-20	2.69E-18	RTL5
498.0265923	-0.1951445	0.08009942	-2.4362785	0.0148393	0.041973751	PIN4
1596.111049	-0.28135739	0.05138478	-5.475501	4.36E-08	3.68E-07	ERCC6L
11954.6757	-0.36871035	0.03474662	-10.611401	2.64E-26	1.30E-24	RPS4X
343.0589613	-0.24598085	0.10057863	-2.4456572	0.0144588	0.041071425	CHIC1
187.4445553	0.347589852	0.14445005	2.4062979	0.0161151	0.045018774	FTX
540.306288	-0.35766077	0.07681094	-4.6563779	3.22E-06	2.03E-05	JPX
27.85526332	0.939994745	0.38895039	2.4167471	0.0156599	0.043923471	ENSG00000271533
1141.327235	0.393733954	0.0596132	6.6048112	3.98E-11	4.90E-10	SLC16A2
2365.214747	-0.53551544	0.05160143	-10.377918	3.13E-25	1.43E-23	RLIM
589.8966451	-0.25761573	0.08025104	-3.2101231	0.0013268	0.004968288	ABCB7
561.6438831	0.394188991	0.08383502	4.7019612	2.58E-06	1.65E-05	PBDC1
4247.11219	-0.29359674	0.04150102	-7.074446	1.50E-12	2.13E-11	ATRX
424.6917494	-0.48367601	0.0998999	-4.8416065	1.29E-06	8.64E-06	ATP7A
22041.2384	-0.34541585	0.0381107	-9.0634864	1.26E-19	3.61E-18	PGK1
1068.932593	-0.25044124	0.06531006	-3.8346505	0.0001257	0.000588606	TAF9B
644.0305942	-0.76494897	0.07394937	-10.344226	4.44E-25	1.99E-23	BRWD3
592.6874016	-0.45005905	0.07447232	-6.0433061	1.51E-09	1.55E-08	APOOL
1238.991734	-0.26041405	0.06215029	-4.1900697	2.79E-05	0.000147897	CHM
389.000058	-0.58019292	0.09097421	-6.3775539	1.80E-10	2.08E-09	DIAPH2
217.1283214	0.462565556	0.12249697	3.7761387	0.0001593	0.000732765	SRPX2
308.3603911	-0.27822498	0.1031697	-2.6967704	0.0070016	0.021855504	SYTL4
2221.830237	-0.24156629	0.05232629	-4.6165381	3.90E-06	2.42E-05	CENPI
543.937833	0.400035248	0.08181823	4.889317	1.01E-06	6.90E-06	GLA
1883.805461	-0.23453813	0.04881153	-4.804974	1.55E-06	1.03E-05	HNRNPH2
2048.833338	0.220434011	0.05045847	4.3686228	1.25E-05	7.10E-05	ARMCX3

77.66986402	0.715025136	0.22399399	3.1921621	0.0014121	0.005255544	ZMAT1
17.61612983	1.215221814	0.42469921	2.8613706	0.0042181	0.013921694	TCEAL2
470.1229941	-0.26351138	0.08743979	-3.0136325	0.0025814	0.009014177	GPRASP2
153.0004528	-0.45102348	0.1489211	-3.0286069	0.0024568	0.008625979	LINC00630
49.35016932	-1.06870466	0.24547675	-4.3535881	1.34E-05	7.55E-05	ENSG00000239407
2046.755599	0.287318682	0.0567842	5.0598352	4.20E-07	3.05E-06	BEX1
62.01113107	2.294609766	0.2610199	8.7909379	1.48E-18	3.82E-17	NXF3
6.252674401	3.169079453	1.18841306	2.6666481	0.0076612	0.02358435	TCEAL5
330.763863	0.330928227	0.10575202	3.1292852	0.0017523	0.006397589	TCEAL1
7624.958823	-0.27591057	0.03843917	-7.1778502	7.08E-13	1.05E-11	MORF4L2
3457.288972	-0.26743889	0.04502772	-5.939428	2.86E-09	2.83E-08	FAM199X
1105.299311	0.325852527	0.06058601	5.3783463	7.52E-08	6.13E-07	MORC4
1019.310532	-0.35109391	0.06743504	-5.2064018	1.93E-07	1.48E-06	RBM41
148.9441328	0.407177577	0.14940325	2.7253595	0.0064232	0.020257269	FRMPD3
6776.565303	0.14443271	0.0407959	3.5403733	0.0003996	0.001693073	PRPS1
246.3341518	0.937170436	0.13026847	7.1941463	6.29E-13	9.35E-12	TSC22D3
284.3711734	0.360570969	0.1124969	3.2051635	0.0013499	0.005046008	ATG4A
358.5794868	-0.32727587	0.09953257	-3.2881285	0.0010086	0.003891462	NXT2
6695.337997	-0.26126214	0.03721558	-7.0202349	2.21E-12	3.09E-11	ACSL4
45.80091436	1.098252954	0.2818549	3.8965189	9.76E-05	0.000467238	ENSG00000289365
2503.43071	-0.45897289	0.05206179	-8.8159253	1.19E-18	3.10E-17	CHRDL1
156.4187564	-0.41487685	0.1403837	-2.9553064	0.0031236	0.010678038	PAK3
425.5557601	-0.3217284	0.092387	-3.4823991	0.0004969	0.002064727	AMOT
10399.99172	0.49347849	0.04005604	12.319704	7.10E-35	5.74E-33	PLS3
1019.76393	0.264173325	0.06205939	4.2567826	2.07E-05	0.000112598	WDR44
2189.555253	0.35065904	0.05628749	6.229786	4.67E-10	5.12E-09	IL13RA1
8.284515948	2.942809638	0.99470833	2.9584649	0.0030918	0.010585867	ZCCHC12
5129.019386	-0.21869737	0.0421374	-5.1901015	2.10E-07	1.60E-06	SLC25A5
2848.367761	0.12687326	0.04311721	2.94252	0.0032555	0.011074148	UBE2A
669.6562007	-0.25495217	0.07174042	-3.5538149	0.0003797	0.001614515	NKRF
715.3363757	0.239635656	0.07400554	3.2380773	0.0012034	0.004553266	SEPTIN6
520.6197082	0.195432636	0.08050907	2.4274612	0.0152049	0.042847749	NDUFA1
541.7292418	-0.37641004	0.08184052	-4.5993116	4.24E-06	2.61E-05	NKAP
975.1176556	-0.2894961	0.06871949	-4.2127217	2.52E-05	0.000134633	ZBTB33
4469.12314	0.372542999	0.04803736	7.7552768	8.82E-15	1.60E-13	LAMP2
5073.570494	-0.38950478	0.04342958	-8.9686517	3.00E-19	8.33E-18	CUL4B
849.8517944	-0.29810416	0.06991848	-4.2635963	2.01E-05	0.000109559	MCTS1
4794.70659	-0.45482008	0.04268899	-10.654272	1.67E-26	8.32E-25	STAG2
3.457568276	4.3417716	1.39095521	3.1214316	0.0017997	0.006547591	TENM1
848.6643877	-0.23477843	0.06902806	-3.4012027	0.0006709	0.002698187	DCAF12L1
1872.495411	-0.36546729	0.05442469	-6.7151008	1.88E-11	2.37E-10	OCRL
835.307255	0.226089018	0.06689519	3.37975	0.0007255	0.002891423	ZDHHC9
759.4455376	-0.20752641	0.06947531	-2.9870525	0.0028168	0.009750075	BCORL1
326.2591581	0.738944326	0.11052665	6.6856662	2.30E-11	2.88E-10	GPC4
2131.740009	-0.52572431	0.04977096	-10.562873	4.43E-26	2.13E-24	PHF6
1857.692517	-0.24100888	0.05925399	-4.0673868	4.75E-05	0.000241887	HPRT1
1974.849481	-0.25832996	0.05075189	-5.0900557	3.58E-07	2.63E-06	PABIR2
707.0727365	0.315443964	0.07608744	4.1458085	3.39E-05	0.000176939	MOSPD1

1005.24729	0.270292797	0.06131883	4.4079901	1.04E-05	6.01E-05	RTL8C
104.1141249	0.475605214	0.18095463	2.6283119	0.008581	0.026056089	ENSG00000286964
211.1846803	-0.46878537	0.12152404	-3.8575527	0.0001145	0.000539594	ZNF75D
241.4378399	-0.29681468	0.11889064	-2.4965354	0.0125413	0.036336802	ZNF449
1782.736863	-0.23268786	0.05432719	-4.2830832	1.84E-05	0.000101019	MMGT1
2812.817809	0.502304228	0.04760283	10.551983	4.97E-26	2.38E-24	FHL1
16.08875655	1.223175047	0.49880247	2.4522233	0.0141977	0.040455048	ARHGEF6
4439.403204	-0.4785678	0.03827088	-12.504751	7.03E-36	6.02E-34	RBMX
1231.682146	-0.1510308	0.0599688	-2.5184895	0.0117859	0.034452431	ATP11C
98.4887444	0.64522922	0.1771396	3.6424899	0.00027	0.001183351	SOX3
981.6485434	-0.40264605	0.06308707	-6.3823859	1.74E-10	2.02E-09	MTMR1
1975.090477	-0.11859107	0.04809323	-2.4658578	0.0136686	0.039139843	HMGB3
1666.618023	-0.28115991	0.0670318	-4.194426	2.74E-05	0.000145304	VMA21
364.5350606	1.154675565	0.11370447	10.155059	3.15E-24	1.30E-22	GABRE
784.1226145	0.329270737	0.0794457	4.144601	3.40E-05	0.000177821	BGN
19.21903907	-1.37865927	0.39610698	-3.4805226	0.0005004	0.002076991	DUSP9
685.2998842	0.334382591	0.07562842	4.4213881	9.81E-06	5.68E-05	SLC6A8
1660.061967	0.190543115	0.05588276	3.4096938	0.0006504	0.002622243	BCAP31
106.1362673	0.936822937	0.18178449	5.1534813	2.56E-07	1.93E-06	ABCD1
128.4369588	0.552217515	0.1596185	3.4596084	0.000541	0.002227112	PLXNB3
601.1754626	0.311648256	0.07575625	4.1138292	3.89E-05	0.00020093	SSR4
4236.007418	0.43983501	0.04086222	10.763855	5.10E-27	2.66E-25	L1CAM
1982.772416	-0.46490285	0.05229841	-8.889426	6.14E-19	1.66E-17	HCFC1
1392.413346	-0.29075958	0.05754334	-5.0528801	4.35E-07	3.16E-06	MECP2
47625.89984	0.089249984	0.03701232	2.4113587	0.0158932	0.044470304	FLNA
5101.574973	-0.45002039	0.04127474	-10.903046	1.11E-27	6.04E-26	RPL10
489.5491529	0.415865354	0.08822367	4.7137619	2.43E-06	1.56E-05	DNASE1L1
1431.517779	0.387607429	0.0570671	6.7921344	1.10E-11	1.43E-10	ATP6AP1
2881.171507	0.208832131	0.0492278	4.2421587	2.21E-05	0.0001193	GDI1
1881.480128	0.212006744	0.05296569	4.0027188	6.26E-05	0.000310682	FAM50A
1266.498372	0.425077373	0.06175134	6.8836946	5.83E-12	7.81E-11	PLXNA3
492.4076842	-0.24903842	0.08586329	-2.9004061	0.0037268	0.012482464	UBL4A
339.1780355	0.313281907	0.09862203	3.1765916	0.0014902	0.00551362	SLC10A3
10.45426844	2.121562517	0.71739501	2.9573143	0.0031033	0.010621269	GAB3
85.4598186	0.52049284	0.20314583	2.5621636	0.0104022	0.030885232	F8A1
1696.648158	-0.24536643	0.05595631	-4.3849642	1.16E-05	6.62E-05	VBP1
93309.67741	0.623050673	0.12413442	5.0191614	5.19E-07	3.72E-06	MT-RNR2
26187.86826	0.937262836	0.15767966	5.9440946	2.78E-09	2.75E-08	MT-ND1
16232.96396	0.751055795	0.12762913	5.8846737	3.99E-09	3.86E-08	MT-ND2
39969.77165	0.653236485	0.13060999	5.0014282	5.69E-07	4.04E-06	MT-CO1
21633.35928	0.581196004	0.14493883	4.00994	6.07E-05	0.000302107	MT-CO2
11492.67871	0.85824888	0.15276393	5.6181384	1.93E-08	1.71E-07	MT-ATP8
16158.79216	0.907292207	0.12814274	7.0803246	1.44E-12	2.05E-11	MT-ATP6
19313.00883	0.648289948	0.12177431	5.3237004	1.02E-07	8.14E-07	MT-CO3
2928.173187	0.493819948	0.09525042	5.1844384	2.17E-07	1.65E-06	MT-ND3
11107.96176	0.688144501	0.16342654	4.2107268	2.55E-05	0.000135745	MT-ND4L
47189.33659	0.711508242	0.14532213	4.8960764	9.78E-07	6.68E-06	MT-ND4
34362.26565	0.580892459	0.11439305	5.0780396	3.81E-07	2.79E-06	MT-ND5

23021.53263	0.728482863	0.1455588	5.0047326	5.59E-07	3.98E-06	MT-CYB
